# Author Correction: Nanomolar inhibitor of the galectin-8 N-terminal domain binds via a non-canonical cation-π interaction

**DOI:** 10.1038/s42004-025-01498-y

**Published:** 2025-04-01

**Authors:** Edvin Purić, Mujtaba Hassan, Fredrik Sjövall, Tihomir Tomašič, Mojca Pevec, Jurij Lah, Jaume Adrover Forteza, Anders Sundin, Hakon Leffler, Ulf J. Nilsson, Derek T. Logan, Marko Anderluh

**Affiliations:** 1 Department of Pharmaceutical Chemistry, Faculty of Pharmacy, University of Ljubljana, Ljubljana, Slovenia; 2 Department of Chemistry, Lund University, Lund, Sweden; 3 Department for Physical Chemistry, Faculty of Chemistry and Chemical Technology, University of Ljubljana, Ljubljana, Slovenia; 4 Department of Bio-engineering Sciences, Structural Biology Brussels, Vrije Universiteit Brussel, Brussels, Belgium; 5 Department of Chemistry, Biochemistry and Structural Biology, Centre for Molecular Protein Science, Lund University, Lund, Sweden; 6 Department of Laboratory Medicine, Section MIG, Lund University, Lund, Sweden

**Keywords:** Carbohydrates, Structure-based drug design, X-ray crystallography, Lead optimization

Correction to: *Communications Chemistry* 10.1038/s42004-025-01458-6, published online 24 February 2025

In the version of this article initially published, the diagram shown in Fig. 5c was an earlier, incorrect panel which has now been replaced; in the first sentence of the Methods “X-ray data collection and structure determination” section, a reference was missing and has now been cited (Ursby, T. et al. BioMAX – the first macromolecular crystallography beamline at MAX IV Laboratory. *J. Synchrotron Radiat*. **27**, 1415–1429 (2020)); and the Acknowledgements section omitted the following text: “We acknowledge the MAX IV Laboratory for beamtime on the BioMAX beamline under proposal 20220263. Research conducted at MAX IV, a Swedish national user facility, is supported by Vetenskapsrådet (Swedish Research Council, VR) under contract 2018-07152, Vinnova (Swedish Governmental Agency for Innovation Systems) under contract 2018-04969 and Formas under contract 2019-02496.” The figure and text have been amended in the HTML and PDF versions of the article.

