# Supplementary material for: Nanomolar inhibitor of the galectin-8 N-terminal domain binds via a non-canonical cation-π interaction
Source: Commun Chem. 2025 Feb 24;8:59. doi: 10.1038/s42004-025-01458-6 (PMC11850616; doi:10.1038/s42004-025-01458-6)
Supplement: Supplementary file 2 — Supplementary Information [file 42004_2025_1458_MOESM2_ESM.pdf]

Supplementary information for the manuscript:

Nanomolar inhibitor of the galectin-8 N-terminal domain binds via a non-canonical cation-π interaction

Edvin Purić[1], Mujtaba Hassan[2], Fredrik Sjövall[2], Tihomir Tomašič[1], Mojca Pevec[3,4], Jurij Lah[3], Jaume Adrover Forteza[5], Anders Sundin[2], Hakon Leffler[6], Ulf J. Nilsson[2], Derek T. Logan[5*], & Marko Anderluh[1,*]

[1]Department of Pharmaceutical Chemistry, Faculty of Pharmacy, University of Ljubljana, Aškerčeva cesta 7, 1000 Ljubljana, Slovenia

[2]Department of Chemistry, Lund University, Box 124, SE-221 00, Lund, Sweden

[3]Department for Physical Chemistry, Faculty of Chemistry and Chemical Technology, University of Ljubljana, Večna pot 13, 1000 Ljubljana, Slovenia

[4]Structural Biology Brussels, Department of Bio-engineering Sciences, Vrije Universiteit Brussel, Pleinlaan 2, 1050, Brussels, Belgium

[5]Centre for Molecular Protein Science, Department of Biochemistry and Structural Biology, Lund University, Box 124, 221 00 Lund, Sweden

[6]Department of Laboratory Medicine, Section MIG, Lund University BMC-C1228b, Klinikgatan 28, 221 84, Lund, Sweden

*Corresponding author: marko.anderluh@ffa.uni-lj.si; derek.logan@biochemistry.lu.se

Contents

Synthesis 6
Supplementary scheme 1 7
Supplementary scheme 2 9
Supplementary scheme 3 18
Supplementary scheme 4 20
Supplementary scheme 5 21
Supplementary scheme 6 25
Supplementary scheme 7 25
Supplementary scheme 8 31
Supplementary scheme 9 33
^{1}H, ^{13}C, ^{19}F NMR and HPLC chromatograms 34
Supplementary Fig. 1 34
Supplementary Fig. 2 35
Supplementary Fig. 3 35
Supplementary Fig. 4 36
Supplementary Fig. 5 36
Supplementary Fig. 6 37
Supplementary Fig. 7 37
Supplementary Fig. 8 38
Supplementary Fig. 9 38
Supplementary Fig. 10 39
Supplementary Fig. 11 39
Supplementary Fig. 12 40
Supplementary Fig. 13 40
Supplementary Fig. 14 41
Supplementary Fig. 15 41
Supplementary Fig. 16 42
Supplementary Fig. 17 42
Supplementary Fig. 18 43
Supplementary Fig. 19 43
Supplementary Fig. 20 44
Supplementary Fig. 21 44
Supplementary Fig. 22 45
Supplementary Fig. 23 46

Supplementary Fig. 24......46
Supplementary Fig. 25......47
Supplementary Fig. 26......47
Supplementary Fig. 27......48
Supplementary Fig. 28......49
Supplementary Fig. 29......50
Supplementary Fig. 30......50
Supplementary Fig. 31......51
Supplementary Fig. 32......51
Supplementary Fig. 33......52
Supplementary Fig. 34......52
Supplementary Fig. 35......53
Supplementary Fig. 36......53
Supplementary Fig. 37......54
Supplementary Fig. 38......54
Supplementary Fig. 39......55
Supplementary Fig. 40......55
Supplementary Fig. 41......56
Supplementary Fig. 42......56
Supplementary Fig. 43......57
Supplementary Fig. 44......58
Supplementary Fig. 45......59
Supplementary Fig. 46......59
Supplementary Fig. 47......60
Supplementary Fig. 48......60
Supplementary Fig. 49......61
Supplementary Fig. 50......61
Supplementary Fig. 51......62
Supplementary Fig. 52......62
Supplementary Fig. 53......63
Supplementary Fig. 54......63
Supplementary Fig. 55......64
Supplementary Fig. 56......64
Supplementary Fig. 57......65
Supplementary Fig. 58......65
Supplementary Fig. 59......66

Supplementary Fig. 60......66
Supplementary Fig. 61......67
Supplementary Fig. 62......67
Supplementary Fig. 63......68
Supplementary Fig. 64......68
Supplementary Fig. 65......69
Supplementary Fig. 66......70
Supplementary Fig. 67......70
Supplementary Fig. 68......71
Supplementary Fig. 69......72
Supplementary Fig. 70......72
Supplementary Fig. 71......73
Supplementary Fig. 72......73
Supplementary Fig. 73......74
Supplementary Fig. 74......74
Supplementary Fig. 75......75
Supplementary Fig. 76......75
Supplementary Fig. 77......76
Supplementary Fig. 78......76
Supplementary Fig. 79......77
Supplementary Fig. 80......77
Supplementary Fig. 81......78
Supplementary Fig. 82......78
Supplementary Fig. 83......79
Supplementary Fig. 84......79
Supplementary Fig. 85......80
Supplementary Fig. 86......81
Supplementary Fig. 87......81
Supplementary Fig. 88......82
Supplementary Fig. 89......82
Supplementary Fig. 90......83
Supplementary Fig. 91......83
Supplementary Fig. 92......84
Supplementary Fig. 93......84
Supplementary Fig. 94......85
Supplementary Fig. 95......85

Protein purification 86
Supplementary Fig. 96 86
X-ray crystallography data 87
Supplementary table 1 87
ITC data 88
Supplementary Fig. 97 88
Supplementary table 2 88
Supplementary table 3. 89
Competitive fluorescence polarisation assay data 89
Supplementary table 4 89
Ligand efficiency (LE) and ligand-lipophilicity efficiency (LLE) calculations 90
Supplementary table 5 90
MD simulation data 91
Supplementary Fig. 98. 91
Supplementary Fig. 99. 91
Supplementary Fig. 100. 93
Supplementary Fig. 101 93
Supplementary Fig. 102. 94
Supplementary Fig. 103. 96
Supplementary Fig. 104. 97
Supplementary Fig. 105. 99
Supplementary Fig. 106. 99
Supplementary Fig. 107. 100
Energy Decomposition Analysis 102
Supplementary Fig. 109 102
Crystal structure 102
Supplementary Fig. 110 102
LNnT fluorescent probe binding data 103
Supplementary Fig. 111 103
Supplementary References 103

Synthesis

The synthesis of compounds **7a**-**f** and **8a**-**f** began with a previously reported[1], methyl ester-protected compound **4**, which was initially protected by benzylidene acetal to give the 4,6-*O*-benzylidene acetal-protected compound **5** as the core compound (Supplementary scheme 1). 2-*O*-Alkylation of **5** with suitable benzyl bromides/chlorides gave **6a**-**f**. Benzylidene acetals of **6a**-**f** were removed with *p*-toluenesulfonic acid monohydrate in methanol/water to obtain esters **7a**-**f**. Alkaline hydrolysis of the methyl esters **7a**-**f** yielded the carboxybenzimidazoles **8a**-**f**.

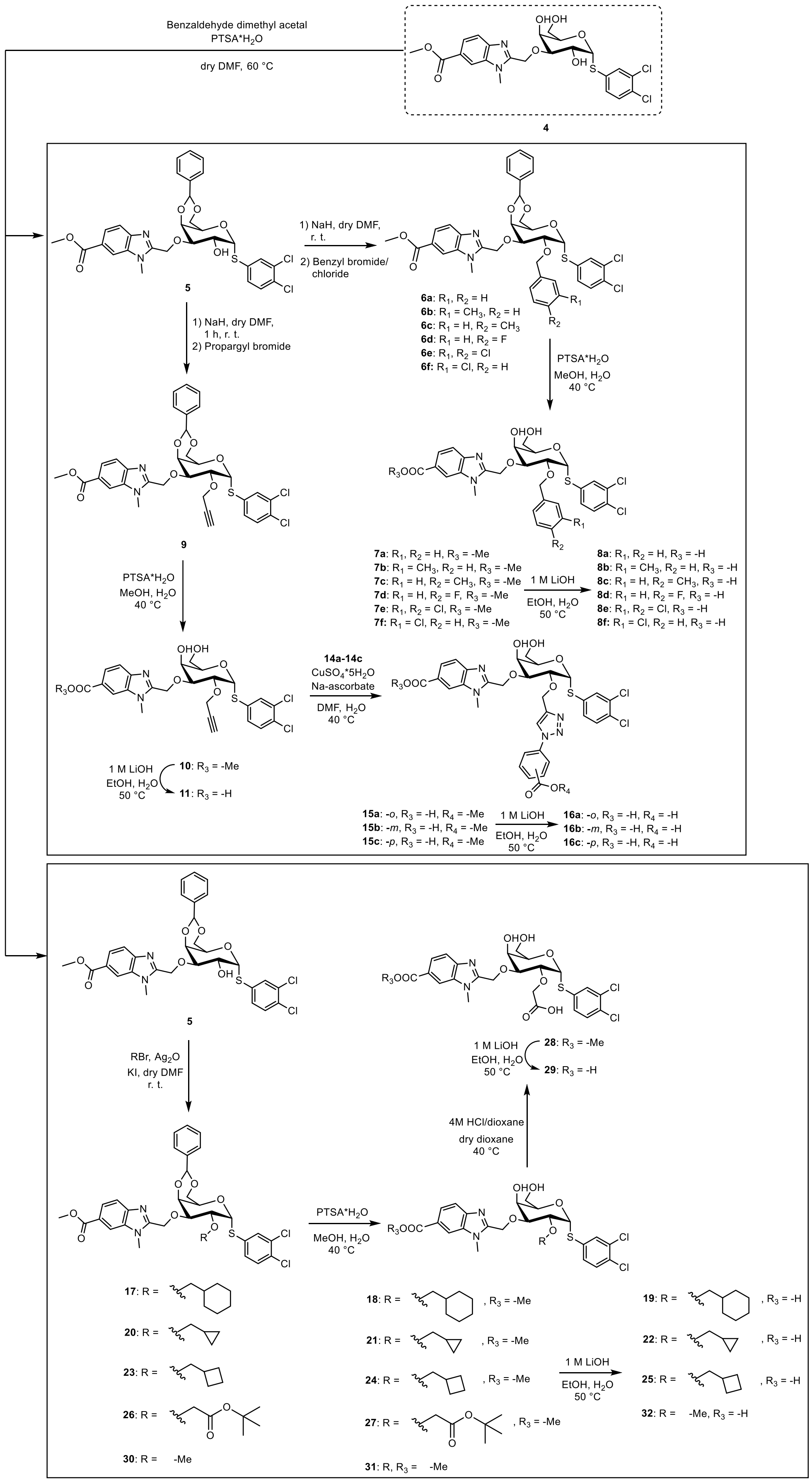

***Supplementary scheme 1**. Chemical synthesis of galectin-8N inhibitors. PTSA*H_2O: p-toluenesulfonic acid monohydrate.*

Using a similar strategy, we synthesised galactosides **10** and **11** starting from compound **5**. 2-*O*-alkylation with propargyl bromide yielded **9**. The benzylidene acetal of compound **9** was removed with *p*-toluenesulfonic acid monohydrate in methanol/water to obtain **10**. Alkaline hydrolysis of the methyl ester **10** gave the carboxybenzimidazole **11**. In addition, the 2-*O*-propargyl derivative **11** was further used in a copper-catalysed alkyne-azide click reaction with the corresponding methyl azidobenzoates **14a**-**c** (prepared as reported by Hassan *et al.*[1]) to give compounds **15a**-**c**. Alkaline hydrolysis of esters **15a**-**c** yielded carboxybenzimidazoles **16a**-**c**.

The 2-*O*-alkylation using silver oxide, potassium iodide, and an appropriate (bromomethyl)cycloalkane in dry DMF proceeded from compound **5**, which yielded **17**, **20**, and **23**. Removal of the benzylidene acetal in the next step gave compounds **18**, **21**, and **24**. Further alkaline hydrolysis of the methyl esters **18**, **21**, and **24** yielded **19**, **22**, and **25**.

The synthesis of **29** started with a similar synthetic strategy for 2-*O*-alkylation using Ag_2O, KI, and *tert*-butyl bromoacetate to yield **26**. Further removal of benzylidene acetal gave compound **27**, where *tert*-butyl ester was removed in the next step by acidolysis using trifluoroacetic acid in dry DCM to yield compound **28**. Finally, alkaline hydrolysis of **28** gave the free carboxylate **29**. To introduce simple 2-*O*-methyl substituent as a reference structure, a 2-*O*-methylation **5** with methyl iodide was performed to yield **30**. Benzylidene acetal removal with *p*-toluenesulfonic acid monohydrate in methanol/H_2O gave compound **31** and further alkaline hydrolysis yielded compound **32**.

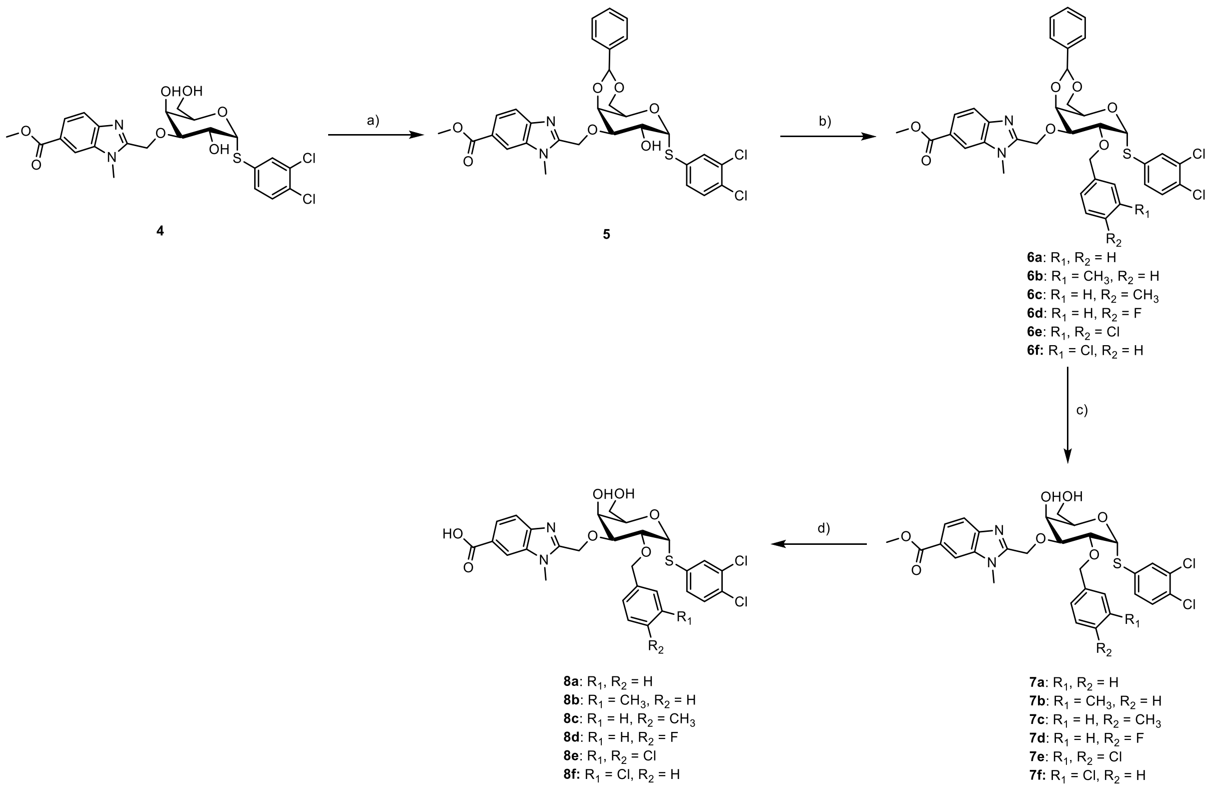

Supplementary scheme 2*. a) Benzaldehyde dimethyl acetal, PTSA*H_2O, dry DMF, 60 °C, 4h (55 %); b) i. NaH, dry DMF, rt, 1h; ii. appropriate benzyl chloride/bromide, rt, 4h (43-79 %); c) PTSA*H_2O, MeOH, H_2O, 40 °C, overnight (54-63 %); d) 1 M LiOH, EtOH/H_2O = 4/1, 50 °C, overnight (81-91 %).*

Compound **4** (500 mg, 0.920 mmol, 1 equiv.) and benzylidene dimethyl acetal (1.12 g, 7.359 mmol, 8 equiv.) were dissolved in dry DMF (7 mL). Then, *p*-toluenesulfonic acid monohydrate (70 mg, 0.368 mmol, 0.40 equiv.) was added to the reaction mixture. The flask was attached to the rotary evaporator, lowered in a water bath, the pressure was set to 200 mbar and the reaction was left stirring at 60 °C for 4 hours. After TLC showed complete consumption of starting material, the mixture was concentrated *in vacuo*. It was redissolved in EtOAc (80 mL) and the organic phase was washed with $NaHCO_{3\,(sat.)}$ (3x40 mL), dried over anhydrous Na_2SO_4, filtered and the solvent was evaporated *in vacuo*. The crude material was purified by a flash column chromatography (mobile phase: n-hexane/EtOAc = 1/3) to obtain **5** as a pale-yellow solid in 55 % yield (288 mg). ^1H NMR (400 MHz, $CDCl_3$) δ 7.98 (dd, J = 8.5, 1.5 Hz, 1H), 7.85 (d, J = 1.0 Hz, 1H), 7.74 (d, J = 8.5 Hz, 1H), 7.58 (d, J = 2.0 Hz, 1H), 7.37 – 7.27 (m, 5H), 7.22 (dd, J = 8.0, 1.6 Hz, 2H), 5.93 (d, J = 5.4 Hz, 1H), 5.08 – 4.98 (m, J = 14.1 Hz, 3H), 4.65 (dd, J = 10.1, 5.4 Hz, 1H), 4.37 (dd, J = 3.6, 1.2 Hz, 1H), 4.15 (dd, 1H), 4.08 (dd, J = 2.1, 1.2 Hz, 1H), 4.01 (dd, 1H), 3.98 (s, 3H), 3.87 (dd, J = 10.1, 3.5 Hz, 1H), 3.64 (s, 3H).

General method for the preparation of compounds **6a**-**f**

Compound **5** (1 equiv.) was dissolved in dry DMF (1-2 mL). NaH (1.50 equiv., 60 %) was added into dry DMF in a separate flask, flushed with argon, and dissolved using ultrasonic sonicator. Afterwards, NaH solution in dry DMF was added to the reaction mixture and left stirring for 1 h at room temperature. Then the appropriate benzyl chloride/bromide (1.70 equiv.) was added to the mixture and left stirring for 4 h at room temperature. After TLC showed almost all starting material was consumed, the mixture was quenched with MeOH (2 mL) and diluted with EtOAc (50 mL). The organic phase was washed with $NaHCO_{3\,(sat.)}$ (3x25 mL), dried over anhydrous Na_2SO_4, filtered and the solvent was evaporated *in vacuo*. The crude material was purified by a flash column chromatography (mobile phase: n-hexane/EtOAc = 1/2) to obtain compounds **6a**-**f**.

a) Compound **6a**

Following the general procedure, the reaction was performed with **5** (200 mg, 0.317 mmol, 1 equiv.), NaH (19 mg, 0.475 mmol, 1.50 equiv., 60 %) and benzyl chloride (62 µL, 68 mg, 0.538 mmol, 1.70 equiv.). Compound **6a** was obtained in 53 % yield (120 mg). ^{1}H NMR (400 MHz, $CDCl_3$) δ 7.98 (dd, 1H), 7.84 (d, J = 1.0 Hz, 1H), 7.76 – 7.72 (m, 1H), 7.48 (d, J = 2.1 Hz, 1H), 7.38 – 7.27 (m, 10H), 7.20 (ddd, J = 8.9, 4.6, 2.1 Hz, 3H), 5.80 (d, J = 5.4 Hz, 1H), 5.10 – 5.03 (m, 2H), 4.95 (d, 1H), 4.76 – 4.68 (m, 2H), 4.37 (d, J = 2.2 Hz, 1H), 4.35 (dd, 1H), 4.08 (dd, J = 12.5, 1.2 Hz, 1H), 4.00 (s, 1H), 3.98 (s, 3H), 3.97 – 3.88 (m, 2H), 3.67 (s, 3H).

b) Compound **6b**

Following the general procedure, the reaction was performed with **5** (200 mg, 0.317 mmol, 1 equiv.), NaH (19 mg, 0.475 mmol, 1.50 equiv., 60 %) and 3-methylbenzyl chloride (71 µL, 75 mg, 0.538 mmol, 1.70 equiv.). Compound **6b** was obtained in 60 % yield (140 mg). ^{1}H NMR (400 MHz, $CDCl_3$) δ 7.98 (dd, J = 8.4, 1.5 Hz, 1H), 7.85 (dd, 1H), 7.75 (d, J = 8.5 Hz, 1H), 7.47 (d, J = 2.1 Hz, 1H), 7.33 – 7.28 (m, 4H), 7.23 – 7.09 (m, 7H), 5.77 (d, J = 5.4 Hz, 1H), 5.09 (d, J = 13.5 Hz, 1H), 5.02 (s, 1H), 4.96 (d, J = 13.6 Hz, 1H), 4.69 (s, 2H), 4.37 (dd, 1H), 4.34 (dd, J = 10.1, 5.4 Hz, 1H), 4.08 (dd, J = 11.2 Hz, 1H), 4.00 (dd, 1H), 3.98 (s, 3H), 3.95 (d, 1H), 3.90 (dd, J = 10.0, 3.7 Hz, 1H), 3.69 (s, 2H), 2.30 (s, 2H).

c) Compound **6c**

Following the general procedure, the reaction was performed with **5** (200 mg, 0.317 mmol, 1 equiv.), NaH (19 mg, 0.475 mmol, 1.50 equiv., 60 %) and 4-methylbenzyl bromide (103 mg, 0.538 mmol, 1.70 equiv.). Compound **6c** was obtained in 56 % yield (130 mg). ^{1}H NMR (400

MHz, $CDCl_3$) δ 7.98 (dd, J = 8.5, 1.5 Hz, 1H), 7.86 (d, J = 1.0 Hz, 1H), 7.75 (d, J = 8.5 Hz, 1H), 7.46 (d, J = 2.1 Hz, 1H), 7.33 – 7.30 (m, 2H), 7.30 – 7.27 (m, 2H), 7.23 (d, J = 7.8 Hz, 4H), 7.19 (dd, J = 8.4, 2.1 Hz, 2H), 7.10 (d, J = 7.8 Hz, 2H), 5.71 (d, J = 5.4 Hz, 1H), 5.10 – 5.03 (m, 2H), 4.95 (d, J = 13.6 Hz, 1H), 4.68 (d, J = 4.0 Hz, 2H), 4.37 (d, J = 3.2 Hz, 1H), 4.33 (dd, J = 10.1, 5.4 Hz, 1H), 4.07 (dd, J = 12.4, 1.2 Hz, 1H), 3.99 (s, 1H), 3.98 (s, 3H), 3.97 – 3.92 (m, 1H), 3.88 (dd, J = 10.1, 3.6 Hz, 1H), 3.69 (s, 3H), 2.34 (s, 3H).

d) Compound **6d**

Following the general procedure, the reaction was performed with **5** (200 mg, 0.317 mmol, 1 equiv.), NaH (19 mg, 0.475 mmol, 1.50 equiv., 60 %) and 4-fluorobenzyl bromide (67 µL, 102 mg, 0.538 mmol, 1.70 equiv.). Compound **6d** was obtained in 43 % yield (100 mg). ^{1}H NMR (400 MHz, $CDCl_3$) δ 7.98 (dd, J = 8.5, 1.5 Hz, 1H), 7.84 (d, J = 1.0 Hz, 1H), 7.75 (d, J = 8.5 Hz, 1H), 7.49 (d, J = 2.1 Hz, 1H), 7.36 – 7.30 (m, 4H), 7.30 – 7.25 (m, 3H), 7.21 (dd, J = 8.4, 2.0 Hz, 3H), 6.99 (td, J = 9.0, 2.4 Hz, 2H), 5.84 (d, J = 5.4 Hz, 1H), 5.07 (d, J = 13.8 Hz, 1H), 5.04 (d, J = 5.7 Hz, 1H), 4.94 (d, J = 13.6 Hz, 1H), 4.67 (q, J = 11.4 Hz, 2H), 4.37 (d, J = 3.3 Hz, 1H), 4.34 (dd, J = 10.0, 5.4 Hz, 1H), 4.10 (dd, J = 1.2 Hz, 1H), 4.00 (d, 2H), 3.98 (s, 3H), 3.91 (dd, J = 10.0, 3.5 Hz, 1H), 3.66 (s, 3H).

e) Compound **6e**

Following the general procedure, the reaction was performed with **5** (200 mg, 0.317 mmol, 1 equiv.), NaH (19 mg, 0.475 mmol, 1.50 equiv., 60 %) and 3,4-dichlorobenzyl chloride (74 µL, 105 mg, 0.538 mmol, 1.70 equiv.). Compound **6e** was obtained in 79 % yield (198 mg). ^{1}H NMR (400 MHz, $CDCl_3$) δ 7.99 (dd, J = 8.5, 1.6 Hz, 1H), 7.85 (d, J = 0.9 Hz, 1H), 7.75 (d, J = 9.0 Hz, 1H), 7.50 (dd, J = 10.9, 2.1 Hz, 2H), 7.38 – 7.27 (m, 5H), 7.23 (dd, J = 8.5, 2.2 Hz, 1H), 7.17 (dd, 2H), 7.14 (dd, J = 8.2, 2.0 Hz, 1H), 5.86 (d, J = 5.4 Hz, 1H), 5.09 (d, J = 13.7 Hz, 1H), 5.02 – 4.92 (m, 2H), 4.66 (ABq, J = 12.1 Hz, 2H), 4.37 (d, J = 3.0 Hz, 1H), 4.33 (dd, J = 10.1, 5.4 Hz, 1H), 4.09 (dd, J = 8.3 Hz, 1H), 4.02 (d, 1H), 3.98 (s, 3H), 3.93 (dd, J = 10.1, 3.6 Hz, 1H), 3.70 (s, 2H).

f) Compound **6f**

Following the general procedure, the reaction was performed with **5** (200 mg, 0.317 mmol, 1 equiv.), NaH (19 mg, 0.475 mmol, 1.50 equiv., 60 %) and 3-chlorobenzyl bromide (71 µL, 111 mg, 0.538 mmol, 1.70 equiv.). Compound **6f** was obtained in 59 % yield (142 mg). ^{1}H NMR (400 MHz, $CDCl_3$) δ 7.98 (dd, J = 8.5, 1.6 Hz, 1H), 7.84 (d, J = 1.0 Hz, 1H), 7.75 (dd, J = 8.5,

0.4 Hz, 1H), 7.51 (d, J = 2.1 Hz, 1H), 7.40 (t, 1H), 7.34 – 7.27 (m, 4H), 7.27 – 7.26 (d, 1H), 7.24 (d, J = 2.2 Hz, 1H), 7.23 – 7.15 (m, 4H), 5.84 (d, J = 5.4 Hz, 1H), 5.09 (d, J = 13.7 Hz, 1H), 5.01 – 4.90 (m, 2H), 4.69 (q, J = 12.0 Hz, 2H), 4.38 (d, J = 3.1 Hz, 1H), 4.33 (dd, J = 10.0, 5.4 Hz, 1H), 4.09 (dd, J = 12.5, 1.3 Hz, 1H), 4.01 (s, 1H), 3.98 (s, 3H), 3.95 (dd, J = 1.7 Hz, 1H), 3.92 (dd, J = 10.0, 3.6 Hz, 1H), 3.69 (s, 3H).

General procedure for compounds **7a**-**f**

Compounds **6a-f** (1 equiv.) were dissolved in MeOH (1 mL/10 mg). para toluene sulfonic acid monohydrate (1.4 equiv.) was added to the reaction mixture, followed by addition of water (1-2 mL). The mixture was left stirring overnight at 40 °C. After all starting material was consumed, MeOH was evaporated, and the crude material was redissolved in EtOAc (50 mL). Afterwards, the organic phase was washed with $NaHCO_{3\,(sat.)}$ (3x30 mL), dried over anhydrous Na_2SO_4, filtered and the solvent was evaporated *in vacuo*. Compounds **7a-f** were obtained via flash column chromatography (mobile phase: DCM/MeOH = 19/1).

a) Compound **7a**

Following the general procedure, the reaction was performed with **6a** (120 mg, 0.169 mmol, 1 equiv.), 12 mL of MeOH and para toluene sulfonic acid monohydrate (44 mg, 0.233 mmol, 1.4 equiv.). Compound **7a** was obtained with 57 % yield (60 mg). ^{1}H NMR (400 MHz, $CDCl_3$) δ 8.03 (d, J = 1.1 Hz, 1H), 7.98 (dd, J = 8.5, 1.5 Hz, 1H), 7.72 (d, J = 8.5 Hz, 1H), 7.53 (d, J = 2.1 Hz, 1H), 7.37 – 7.29 (m, 6H), 7.26 (dd, J = 8.4, 2.1 Hz, 1H), 5.73 (d, J = 5.5 Hz, 1H), 5.13 (d, J = 14.1 Hz, 1H), 4.87 (d, J = 14.1 Hz, 1H), 4.69 (ABq, J = 11.4 Hz, 2H), 4.35 (dd, J = 9.8, 5.5 Hz, 1H), 4.30 (d, J = 2.0 Hz, 1H), 4.26 (t, J = 4.7 Hz, 1H), 3.97 (s, 3H), 3.94 (dd, J = 11.8, 4.9 Hz, 1H), 3.83 (dd, J = 11.1, 3.9 Hz, 1H), 3.78 (dd, J = 9.9, 3.1 Hz, 1H), 3.71 (s, 3H). ^{13}C NMR (101 MHz, $CDCl_3$) δ 167.3, 153.8, 144.9, 137.6, 135.5, 134.1, 133.0, 132.8, 131.5, 130.8, 130.6, 128.5, 128.1, 128.1, 125.1, 123.9, 119.3, 111.8, 87.1, 79.9, 77.2, 75.2, 72.9, 70.5, 67.8, 64.3, 62.9, 52.3, 30.1. HRMS calcd $C_{30}H_{31}O_7N_2Cl_2S$ + H^+ $(M+H)^+$: 633.1224, found: 633.1211. HPLC purity: 97.1 %.

b) Compound **7b**

Following the general procedure, the reaction was performed with **6b** (190 mg, 0.258 mmol, 1 equiv.), 19 mL of MeOH and para toluene sulfonic acid monohydrate (69 mg, 0.362 mmol, 1.4 equiv.). Compound **7b** was obtained with 63 % yield (105 mg). ^{1}H NMR (400 MHz, $CDCl_3$) δ 8.03 (d, J = 1.0 Hz, 1H), 7.98 (dd, J = 8.5, 1.5 Hz, 1H), 7.71 (d, J = 8.5 Hz, 1H), 7.51 (d, J =

2.1 Hz, 1H), 7.33 (d, J = 8.4 Hz, 1H), 7.27 (dd, J = 2.2 Hz, 1H), 7.25 (dd, J = 2.1 Hz, 1H), 7.23 – 7.17 (m, 1H), 7.14 (d, J = 6.6 Hz, 2H), 7.10 (d, J = 7.3 Hz, 1H), 5.70 (d, J = 5.5 Hz, 1H), 5.13 (d, J = 13.9 Hz, 1H), 4.88 (d, J = 14.0 Hz, 1H), 4.65 (ABq, J = 11.4 Hz, 2H), 4.33 (dd, J = 8.8, 4.4 Hz, 1H), 4.31 (s, 1H), 4.27 (t, J = 4.8 Hz, 1H), 3.97 (s, 3H), 3.93 (dd, J = 11.7, 5.0 Hz, 1H), 3.83 (dd, J = 11.8, 4.7 Hz, 1H), 3.77 (dd, J = 9.9, 3.1 Hz, 1H), 3.72 (s, 3H), 2.30 (s, 3H). ^{13}C NMR (101 MHz, $CDCl_3$) δ 167.3, 153.8, 144.9, 138.2, 137.5, 135.5, 134.2, 132.9, 132.8, 131.5, 130.8, 130.6, 128.9, 128.4, 125.2, 125.1, 123.9, 119.3, 111.8, 87.3, 79.8, 77.2, 75.2, 73.1, 70.5, 67.8, 64.3, 62.9, 52.3, 30.1, 21.4. HRMS calcd $C_{31}H_{33}O_7N_2Cl_2S$ + H^+ $(M+H)^+$: 647.1380, found: 647.1373. HPLC purity: 97.7 %.

c) Compound **7c**

Following the general procedure, the reaction was performed with **6c** (130 mg, 0.177 mmol, 1 equiv.), 13 mL of MeOH and para toluene sulfonic acid monohydrate (47 mg, 0.247 mmol, 1.4 equiv.). Compound **7c** was obtained with 62 % yield (71 mg). ^{1}H NMR (400 MHz, $CDCl_3$) δ 8.07 (d, J = 1.1 Hz, 1H), 8.00 (dd, J = 8.5, 1.5 Hz, 1H), 7.74 (d, J = 8.5 Hz, 1H), 7.50 (d, J = 2.1 Hz, 1H), 7.33 (d, J = 8.4 Hz, 1H), 7.26 – 7.22 (m, 3H), 7.11 (d, J = 7.8 Hz, 2H), 5.64 (d, J = 5.5 Hz, 1H), 5.16 (d, J = 14.2 Hz, 1H), 4.87 (d, J = 14.2 Hz, 1H), 4.71 – 4.61 (m, 2H), 4.32 (dd, J = 9.8, 5.5 Hz, 1H), 4.26 (dd, J = 11.6, 3.6 Hz, 2H), 3.97 (s, 3H), 3.92 (dd, 1H), 3.82 (dd, J = 8.0, 4.1 Hz, 1H), 3.77 (dd, J = 9.9, 3.1 Hz, 1H), 3.75 (s, 3H), 2.33 (s, 3H). ^{13}C NMR (101 MHz, $CDCl_3$) δ 167.3, 153.9, 148.2, 145.0, 138.0, 135.6, 134.5, 134.2, 132.9, 132.8, 131.5, 130.8, 130.6, 129.2, 128.3, 125.1, 124.0, 119.4, 111.8, 87.4, 79.9, 77.2, 75.0, 73.0, 70.4, 68.1, 64.4, 63.3, 52.3, 30.1, 21.2. HRMS calcd $C_{31}H_{33}O_7N_2Cl_2S$ + H^+ $(M+H)^+$: 647.1380, found: 647.1375. HPLC purity: 97.0 %.

d) Compound **7d**

Following the general procedure, the reaction was performed with **6d** (100 mg, 0.135 mmol, 1 equiv.), 10 mL of MeOH and para toluene sulfonic acid monohydrate (36 mg, 0.189 mmol, 1.4 equiv.). Compound **7d** was obtained with 62 % yield (55 mg). ^{1}H NMR (400 MHz, $CDCl_3$) δ 8.06 (d, J = 1.0 Hz, 1H), 8.00 (dd, J = 8.5, 1.5 Hz, 1H), 7.74 (d, J = 8.5 Hz, 1H), 7.54 (d, J = 2.1 Hz, 1H), 7.35 (d, J = 8.4 Hz, 1H), 7.34 – 7.31 (m, 1H), 7.31 (d, J = 5.4 Hz, 1H), 7.29 (d, J = 2.1 Hz, 1H), 7.05 – 6.94 (m, 2H), 5.77 (d, J = 5.5 Hz, 1H), 5.47 (s, 1H), 5.17 (d, J = 14.5 Hz, 1H), 4.86 (d, J = 14.4 Hz, 1H), 4.66 (ABq, J = 11.3 Hz, 2H), 4.34 (dd, J = 9.8, 5.5 Hz, 1H), 4.30 – 4.23 (m, 2H), 3.97 (s, 3H), 3.93 (d, J = 4.8 Hz, 1H), 3.85 – 3.77 (m, 2H), 3.75 (s, 3H). ^{13}C NMR (101 MHz, $CDCl_3$) δ 167.3, 153.9, 145.0, 135.5, 134.0, 133.4, 132.94, 132.91, 131.6,

130.7, 130.7, 129.8, 129.7, 125.2, 124.0, 119.4, 115.5, 115.3, 111.8, 87.0, 80.2, 77.2, 74.9, 72.1, 70.4, 68.0, 64.3, 63.3, 52.3, 30.1. ^{19}F NMR (376 MHz, $CDCl_3$) δ -112.96 (tt, J = 8.5, 5.2 Hz). HRMS calcd $C_{30}H_{30}O_7N_2Cl_2FS$ + H^+ $(M+H)^+$: 651.1129, found: 651.1122. HPLC purity: 99.73 %.

e) Compound **7e**

Following the general procedure, the reaction was performed with **6e** (198 mg, 0.250 mmol, 1 equiv.), 20 mL of MeOH and para toluene sulfonic acid monohydrate (67 mg, 0.351 mmol, 1.4 equiv.). Compound **7e** was obtained with 59 % yield (104 mg). ^{1}H NMR (400 MHz, $CDCl_3$) δ 8.09 (d, J = 0.9 Hz, 1H), 8.01 (dd, J = 8.5, 1.5 Hz, 1H), 7.75 (d, J = 8.5 Hz, 1H), 7.57 (d, J = 2.1 Hz, 1H), 7.49 (d, J = 1.9 Hz, 1H), 7.37 (dd, J = 8.3, 1.3 Hz, 2H), 7.30 (dd, J = 8.4, 2.1 Hz, 1H), 7.15 (dd, J = 8.2, 2.0 Hz, 1H), 5.80 (d, J = 5.5 Hz, 1H), 5.19 (d, J = 14.7 Hz, 1H), 4.85 (d, J = 14.7 Hz, 1H), 4.66 (ABq, J = 12.0 Hz, 2H), 4.34 (dd, J = 9.8, 5.5 Hz, 1H), 4.26 (dd, J = 3.9 Hz, 2H), 3.97 (s, 3H), 3.95 (d, J = 4.8 Hz, 1H), 3.82 (dd, J = 9.8, 3.0 Hz, 1H), 3.78 (s, 3H), 2.81 (s, 1H). ^{13}C NMR (101 MHz, $CDCl_3$) δ 167.3, 154.0, 138.0, 135.5, 133.8, 132.99, 132.97, 132.6, 131.9, 131.8, 130.8, 130.7, 130.4, 129.6, 126.8, 125.2, 124.1, 119.4, 111.8, 86.9, 80.6, 77.2, 75.3, 71.5, 70.4, 67.9, 64.2, 63.4, 52.3, 30.1. HRMS calcd $C_{30}H_{29}O_7N_2Cl_4S$ + H^+ $(M+H)^+$: 701.04441, found: 701.04161. HPLC purity: 97.3 %.

f) Compound **7f**

Following the general procedure, the reaction was performed with **6f** (111 mg, 0.147 mmol, 1 equiv.), 11 mL of MeOH and para toluene sulfonic acid monohydrate (39 mg, 0.205 mmol, 1.4 equiv.). Compound **7f** was obtained with 54 % yield (54 mg). ^{1}H NMR (400 MHz, $CDCl_3$) δ 8.09 (d, J = 1.0 Hz, 1H), 8.01 (dd, J = 8.5, 1.5 Hz, 1H), 7.75 (d, J = 8.5 Hz, 1H), 7.57 (d, J = 2.1 Hz, 1H), 7.41 (s, 1H), 7.36 (d, J = 8.4 Hz, 1H), 7.30 (dd, J = 8.4, 2.1 Hz, 1H), 7.27 (d, J = 5.3 Hz, 1H), 7.25 – 7.19 (m, J = 11.3, 7.0 Hz, 2H), 5.77 (d, J = 5.5 Hz, 1H), 5.58 (s, 1H), 5.20 (d, J = 14.6 Hz, 1H), 4.85 (d, J = 14.6 Hz, 1H), 4.69 (ABq, J = 11.9 Hz, 2H), 4.34 (dd, J = 9.8, 5.5 Hz, 1H), 4.27 (dd, 2H), 3.97 (s, 3H), 3.95 (d, 1H), 3.82 (dd, J = 9.8, 3.0 Hz, 2H), 3.78 (s, 3H). ^{13}C NMR (101 MHz, $CDCl_3$) δ 154.0, 151.0, 139.8, 134.4, 133.9, 133.1, 132.8, 131.7, 130.9, 130.7, 129.8, 128.1, 127.9, 125.8, 125.2, 124.7, 124.1, 119.4, 111.8, 87.1, 80.5, 77.2, 75.3, 72.2, 70.4, 68.0, 64.3, 63.4, 52.3, 30.1. HRMS calcd $C_{30}H_{30}O_7N_2Cl_3S$ + H^+ $(M+H)^+$: 667.0834, found: 667.0804. HPLC purity: 99.1 %.

General procedure for compounds **8a-f**

Compounds **7a-f** (1 equiv.) were dissolved in EtOH/H_2O = 4/1. Then $LiOH_{(aq.)}$ (1 M, 6 equiv.) was added to the reaction mixture. The mixture was left stirring overnight at 50 °C. After all starting material was consumed, the solvent was evaporated *in vacuo* and the crude was redissolved into EtOAc (10 ml) and 10 mL of water was added. The mixture was neutralized with 1 M HCl (to pH = 3) and the water phase was washed with EtOAc (3x20 ml). Combined organic phases were dried over anhydrous Na_2SO_4, filtered and the solvent was evaporated in vacuo.

a) Compound **8a**

Following the general procedure, the reaction was performed with **7a** (25 mg, 0.039 mmol, 1 equiv.) and 1M $LiOH_{(aq.)}$ (6 mg, 0.237 mmol, 6 equiv.) to obtain compound **8a** in 90 % yield (22 mg). ^{1}H NMR (400 MHz, DMSO-d_6) δ 12.44 (s, 1H), 8.15 (d, J = 1.0 Hz, 1H), 7.84 (dd, J = 8.4, 1.5 Hz, 1H), 7.77 (d, J = 2.1 Hz, 1H), 7.68 (d, J = 8.4 Hz, 1H), 7.56 (d, J = 8.4 Hz, 1H), 7.46 (dd, J = 8.5, 2.1 Hz, 1H), 7.35 (dd, J = 7.7, 1.7 Hz, 2H), 7.32 – 7.26 (m, 3H), 6.07 (d, J = 5.4 Hz, 1H), 5.21 (s, 1H), 4.98 (ABq, J = 12.8 Hz, 2H), 4.74 (d, J = 11.8 Hz, 1H), 4.57 (d, J = 11.8 Hz, 1H), 4.14 (dd, J = 10.0, 5.5 Hz, 1H), 4.11 (s, 1H), 4.00 (t, J = 6.3 Hz, 1H), 3.84 (s, 3H), 3.63 (dd, J = 10.0, 3.0 Hz, 1H), 3.56 (dd, J = 10.9, 6.1 Hz, 1H), 3.37 (dd, J = 10.8, 6.4 Hz, 1H, this signal overlaps with water). ^{13}C NMR (101 MHz, DMSO-d_6) δ 168.4, 154.6, 145.3, 138.6, 136.2, 136.0, 132.9, 131.8, 131.8, 131.1, 129.9, 128.6, 128.2, 128.0, 125.8, 123.4, 123.4, 119.2, 112.5, 86.5, 78.7, 75.1, 72.8, 71.5, 65.2, 64.0, 60.5, 30.6, 21.6. HRMS calcd $C_{29}H_{29}O_7N_2Cl_2S + H^+$ $(M+H)^+$: 619.1067, found: 619.1065. HPLC purity: 99.29 %.

b) Compound **8b**

Following the general procedure, the reaction was performed with **7b** (84 mg, 0.130 mmol, 1 equiv.) and 1 M $LiOH_{(aq.)}$ (10 mg, 0.778 mmol, 6 equiv.) to obtain compound **8b** in 85 % yield (70 mg). ^{1}H NMR (400 MHz, DMSO-d_6) δ 8.15 (d, J = 1.0 Hz, 1H), 7.83 (dd, J = 8.4, 1.5 Hz, 1H), 7.76 (d, J = 2.1 Hz, 1H), 7.68 (d, J = 8.5 Hz, 1H), 7.57 (d, J = 8.4 Hz, 1H), 7.46 (dd, J = 8.4, 2.1 Hz, 1H), 7.21 – 7.15 (m, 2H), 7.13 (d, J = 7.6 Hz, 1H), 7.07 (d, J = 7.3 Hz, 1H), 6.04 (d, J = 5.4 Hz, 1H), 5.19 (s, 1H), 4.98 (ABq, J = 12.7 Hz, 2H), 4.68 (d, J = 11.8 Hz, 1H), 4.53 (d, J = 11.8 Hz, 1H), 4.12 (dd, J = 9.9, 5.5 Hz, 2H), 4.00 (t, J = 6.3 Hz, 1H), 3.85 (s, 3H), 3.63 (dd, J = 10.0, 3.0 Hz, 1H), 3.56 (dd, J = 10.8, 6.1 Hz, 1H), 3.38 (dd, J = 10.9, 6.3 Hz, 1H, this signal overlaps with water), 2.21 (s, 3H). ^{13}C NMR (101 MHz, DMSO-d_6) δ 168.3, 154.6, 145.4, 138.5, 137.7, 136.2, 136.0, 132.8, 131.8, 131.8, 131.1, 129.9, 128.8, 128.6, 128.5, 125.3,

123.3, 119.2, 112.6, 86.6, 78.7, 75.1, 72.9, 71.6, 65.3, 64.0, 60.5, 30.6, 21.6, 21.4. HRMS calcd $C_{30}H_{31}O_7N_2Cl_2S + H^+$ $(M+H)^+$: 633.1224, found: 633.1220. HPLC purity: 99.54 %.

c) Compound **8c**

Following the general procedure, the reaction was performed with **7c** (34 mg, 0.053 mmol, 1 equiv.) and 1 M $LiOH_{(aq.)}$ (7.5 mg, 0.315 mmol, 6 equiv.) to obtain compound **8c** in 81 % yield (27 mg). ^{1}H NMR (400 MHz, DMSO-d_6) δ 12.85 (s, 1H), 8.15 (d, J = 1.0 Hz, 1H), 7.84 (dd, J = 8.4, 1.6 Hz, 1H), 7.75 (d, J = 2.1 Hz, 1H), 7.69 (d, J = 8.4 Hz, 1H), 7.56 (d, J = 8.4 Hz, 1H), 7.45 (dd, J = 8.5, 2.1 Hz, 1H), 7.22 (d, J = 8.0 Hz, 2H), 7.08 (d, J = 7.8 Hz, 2H), 6.00 (d, J = 5.4 Hz, 1H), 5.17 (d, J = 5.1 Hz, 1H), 4.96 (ABq, J = 12.8 Hz, 2H), 4.69 – 4.64 (m, 1H), 4.52 (d, J = 11.6 Hz, 1H), 4.11 (dd, J = 10.0, 5.4 Hz, 2H), 3.99 (t, J = 6.3 Hz, 1H), 3.84 (s, 3H), 3.61 (dd, J = 10.0, 3.0 Hz, 1H), 3.54 (dd, J = 11.0, 5.9 Hz, 1H), 2.27 (s, 3H). ^{13}C NMR (101 MHz, DMSO-d_6) δ 168.2, 154.7, 145.4, 137.1, 136.2, 136.0, 135.5, 133.2, 132.8, 131.8, 131.7, 131.0, 129.9, 129.2, 128.4, 125.3, 123.3, 119.2, 112.6, 86.6, 78.7, 74.9, 72.8, 71.4, 65.3, 64.0, 60.5, 39.3, 30.6, 21.2. HRMS calcd $C_{30}H_{31}O_7N_2Cl_2S + H^+$ $(M+H)^+$: 633.1224, found: 633.1219. HPLC purity: 99.05 %.

d) Compound **8d**

Following the general procedure, the reaction was performed with **7d** (28 mg, 0.043 mmol, 1 equiv.) and 1 M $LiOH_{(aq.)}$ (6 mg, 0.258 mmol, 6 equiv.) to obtain compound **8d** in 88 % yield (24 mg). ^{1}H NMR (400 MHz, DMSO-d_6) δ 12.85 (s, 1H), 8.15 (d, J = 1.0 Hz, 1H), 7.83 (dd, J = 8.4, 1.5 Hz, 1H), 7.78 (d, J = 2.1 Hz, 1H), 7.68 (d, J = 8.5 Hz, 1H), 7.57 (d, J = 8.4 Hz, 1H), 7.47 (dd, J = 8.5, 2.1 Hz, 1H), 7.38 (dd, J = 8.6, 5.7 Hz, 2H), 7.10 (t, J = 8.9 Hz, 2H), 6.08 (d, J = 5.4 Hz, 1H), 5.20 (d, J = 4.9 Hz, 1H), 4.96 (ABq, J = 12.8 Hz, 2H), 4.71 (d, J = 11.7 Hz, 1H), 4.55 (d, J = 11.7 Hz, 1H), 4.13 (dd, J = 10.0, 5.4 Hz, 2H), 4.00 (t, J = 6.3 Hz, 1H), 3.84 (s, 3H), 3.62 (dd, J = 10.0, 3.0 Hz, 1H), 3.59 – 3.52 (m, 1H). ^{13}C NMR (101 MHz, DMSO-d_6) δ 163.2, 154.6, 145.3, 136.2, 135.9, 134.8, 134.8, 132.9, 131.9, 131.8, 131.1, 130.3, 130.2, 130.0, 123.4, 119.2, 115.5, 115.3, 112.6, 86.4, 79.8, 79.1, 78.7, 75.0, 72.8, 70.7, 65.2, 63.9, 60.5, 30.6. ^{19}F NMR (376 MHz, DMSO-d_6) δ -114.87, -114.88, -114.89, -114.91, -114.92, -114.93, -114.95. HRMS calcd $C_{29}H_{28}O_7N_2Cl_2FS + H^+$ $(M+H)^+$: 637.0973, found: 637.0967. HPLC purity: 98.3 %.

e) Compound **8e**

Following the general procedure, the reaction was performed with **7e** (70 mg, 0.100 mmol, 1 equiv.) and 1 M $LiOH_{(aq.)}$ (14 mg, 0.597 mmol, 6 equiv.). to obtain compound **8e** in 88 % yield (60 mg). ^{1}H NMR (400 MHz, DMSO-d_6) δ 8.16 (d, J = 1.0 Hz, 1H), 7.84 (dd, J = 8.4, 1.5 Hz, 1H), 7.78 (d, J = 2.1 Hz, 1H), 7.69 (d, J = 8.5 Hz, 1H), 7.60 (d, J = 1.8 Hz, 1H), 7.57 (d, J = 8.4 Hz, 1H), 7.53 – 7.50 (m, 1H), 7.47 (dd, J = 8.4, 2.1 Hz, 1H), 7.32 (dd, J = 8.3, 1.9 Hz, 1H), 6.10 (d, J = 5.4 Hz, 1H), 4.97 (ABq, J = 12.7 Hz, 2H), 4.73 (d, J = 12.7 Hz, 1H), 4.61 (d, J = 12.7 Hz, 1H), 4.16 – 4.10 (m, J = 10.1, 5.4 Hz, 2H), 4.02 (t, J = 6.3 Hz, 1H), 3.87 (s, 3H), 3.65 (dd, J = 10.0, 3.0 Hz, 1H), 3.57 (dd, J = 10.9, 6.1 Hz, 1H), 3.39 (dd, J = 10.9, 6.4 Hz, 1H, this signal overlaps with water). ^{13}C NMR (101 MHz, DMSO-d_6) δ 168.4, 154.5, 145.3, 140.0, 136.2, 135.9, 132.9, 131.8, 131.8, 131.3, 131.1, 130.8, 130.4, 130.0, 129.8, 128.1, 123.4, 119.2, 112.6, 86.4, 78.6, 75.2, 72.9, 69.8, 65.1, 63.7, 60.5, 30.5, 21.6. HRMS calcd $C_{29}H_{27}O_7N_2Cl_4S$ + H^+ $(M+H)^+$: 687.0288, found: 687.0279. HPLC purity: 99.2 %.

f) Compound **8f**

Following the general procedure, the reaction was performed with **7f** (65 mg, 0.097 mmol, 1 equiv.) and 1 M $LiOH_{(aq.)}$ (14 mg, 0.584 mmol, 6 equiv.) to obtain compound **8f** in 91 % yield (58 mg). ^{1}H NMR (400 MHz, DMSO-d_6) δ 8.14 (d, J = 1.0 Hz, 1H), 7.83 (dd, J = 8.4, 1.5 Hz, 1H), 7.78 (d, J = 2.1 Hz, 1H), 7.68 (d, J = 8.5 Hz, 1H), 7.57 (d, J = 8.4 Hz, 1H), 7.47 (dd, J = 8.5, 2.1 Hz, 1H), 7.42 (s, 1H), 7.34 – 7.28 (m, 3H), 6.09 (d, J = 5.4 Hz, 1H), 4.97 (ABq, J = 12.7 Hz, 2H), 4.74 (d, J = 12.4 Hz, 1H), 4.60 (d, J = 12.4 Hz, 1H), 4.13 (dd, J = 9.9, 5.5 Hz, 2H), 4.01 (t, J = 6.3 Hz, 1H), 3.86 (s, 3H), 3.65 (dd, J = 10.0, 3.0 Hz, 1H), 3.57 (dd, J = 10.9, 6.1 Hz, 1H), 3.38 (dd, J = 10.9, 6.4 Hz, 1H, this signal overlaps with water). ^{13}C NMR (101 MHz, DMSO-d_6) δ 172.5, 154.5, 145.3, 141.3, 136.2, 135.9, 133.4, 132.9, 131.9, 131.8, 131.1, 130.5, 130.0, 127.8, 127.7, 126.5, 123.4, 119.1, 112.5, 86.5, 78.6, 75.2, 72.9, 70.5, 65.1, 63.8, 60.5, 30.5, 21.6. HRMS calcd $C_{30}H_{31}O_7N_2Cl_2S$ + H^+ $(M+H)^+$: 653.0677, found: 653.0670. HPLC purity: 99.59 %.

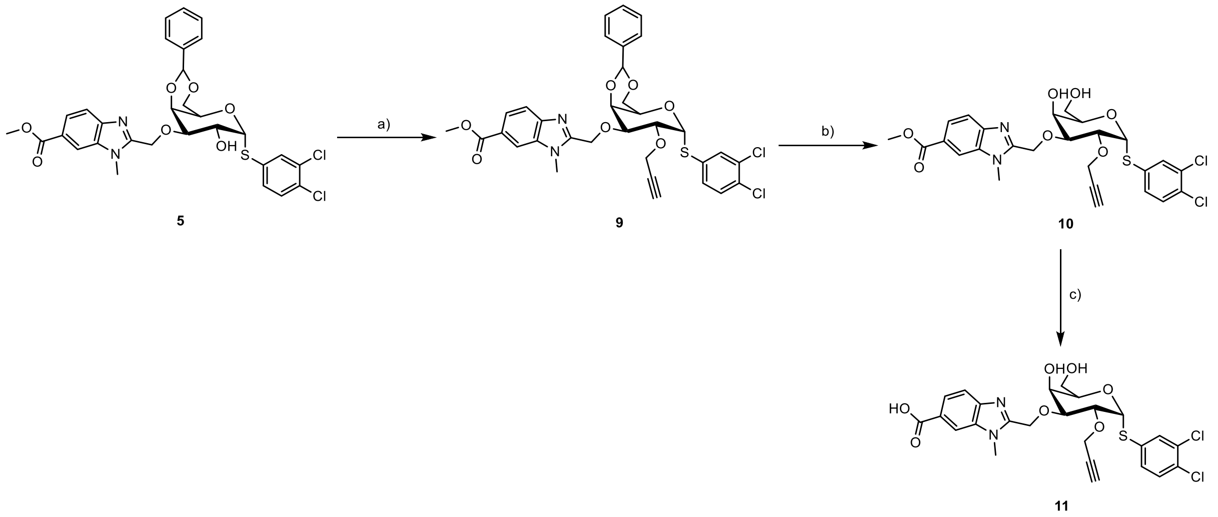

Supplementary scheme 3. a) i. NaH, dry DMF, rt, 1h; ii. Propargyl bromide, rt, 4 h (71 %); b) PTSAH_2O, MeOH, H_2O, 40 °C, overnight (77 %); c) 1 M LiOH, EtOH/ H_2O = 4/1, 50 °C, overnight (82 %).*

Synthesis of compound **9**

Compound **5** (382 mg, 0.605 mmol, 1 equiv.) was dissolved in dry DMF (3-4 mL). NaH (36 mg, 0.907 mmol, 1.5 equiv., 60 %) was added into dry DMF in a separate flask, flushed with argon, and dissolved using ultrasonic sonicator. Afterwards, NaH solution in dry DMF was added to the reaction mixture and left stirring for 1 h at room temperature. Propargyl bromide (110 µL, 153 mg, 1.03 mmol, 1.7 equiv., 80 % wt in toluene) was added to the reaction mixture and it was left stirring for 4 h at room temperature. After TLC showed all starting material was consumed, the mixture was quenched with MeOH (2 mL) and diluted with EtOAc (80 mL). The organic phase was washed with $NaHCO_{3\ (sat.)}$ (3x40 mL), dried over anhydrous Na_2SO_4, filtered and concentrated in vacuo. The crude material was purified via flash column chromatography (mobile phase: n-hexane/EtOAc =1/2) to obtain **9** as an oil in 71 % yield (287 mg). ^1H NMR (400 MHz, $CDCl_3$) δ 7.99 (dd, J = 8.5, 1.5 Hz, 1H), 7.88 (d, J = 1.0 Hz, 1H), 7.75 (d, J = 8.5 Hz, 1H), 7.56 (d, J = 2.1 Hz, 1H), 7.35 – 7.24 (m, 5H), 7.18 (dd, J = 8.0, 1.5 Hz, 2H), 5.99 (d, J = 5.4 Hz, 1H), 5.11 (d, J = 13.6 Hz, 1H), 4.93 (d, J = 12.4 Hz, 2H), 4.49 (dd, J = 10.1, 5.4 Hz, 1H), 4.40 (dd, J = 2.4, 0.9 Hz, 2H), 4.36 (d, J = 2.9 Hz, 1H), 4.10 (dd, J = 3.4, 1.4 Hz, 1H, this signal overlaps with EtOAc), 4.03 (s, 1H), 3.98 (s, 3H), 3.96 (dd, 1H), 3.89 (dd, J = 10.1, 3.6 Hz, 1H), 3.78 (s, 3H), 2.47 (t, J = 2.4 Hz, 1H).

Synthesis of compound **10**

Compound **9** (269 mg, 0.402 mmol, 1 equiv.) was dissolved in MeOH (20 mL). para toluene sulfonic acid (107 mg, 0.562 mmol, 1.4 equiv.) was added to the reaction mixture, followed by addition of water (2 mL). The mixture was left stirring overnight at 40 °C. After all starting

material was consumed, MeOH was evaporated, and the crude material was redissolved in EtOAc (50 mL). The organic phase was washed with $NaHCO_3$ (sat.) (3x30 mL), dried over anhydrous Na_2SO_4, filtered and concentrated in vacuo. The crude material was purified via flash column chromatography (mobile phase: DCM/MeOH = 19/1) to obtain compound **10** in 77 % yield (180 mg). ^{1}H NMR (400 MHz, $CDCl_3$) δ 8.11 (s, 1H), 8.00 (dd, J = 8.5, 1.4 Hz, 1H), 7.74 (d, J = 8.5 Hz, 1H), 7.60 (d, J = 1.8 Hz, 1H), 7.36 (d, J = 8.5 Hz, 1H), 7.33 (dd, J = 8.5, 1.8 Hz, 1H), 5.92 (d, J = 5.5 Hz, 1H), 5.55 (s, 1H), 5.20 (d, J = 14.2 Hz, 1H), 4.86 (d, J = 14.2 Hz, 1H), 4.50 (dd, J = 9.9, 5.5 Hz, 1H), 4.38 (d, J = 2.4 Hz, 2H), 4.31 – 4.25 (m, 2H), 3.97 (s, 3H), 3.96 – 3.94 (m, 1H), 3.86 (s, 3H), 3.77 (dd, J = 9.9, 2.9 Hz, 1H), 2.99 (s, 1H), 2.47 (t, J = 2.4 Hz, 1H). ^{13}C NMR (101 MHz, $CDCl_3$) δ 167.3, 153.9, 144.9, 133.9, 133.0, 132.9, 131.7, 130.8, 130.7, 125.2, 124.0, 119.4, 111.8, 86.9, 80.2, 79.0, 75.6, 74.1, 70.5, 68.0, 64.3, 63.2, 58.1, 52.3, 30.3, 29.7. HRMS calcd $C_{26}H_{27}O_7N_2Cl_2S + H^+$ $(M+H)^+$: 581.0911, found: 581.0909. HPLC purity: 99.6 %.

Synthesis of compound **11**

Compound **10** (150 mg, 0.258 mmol, 1 equiv.) were dissolved in EtOH/H_2O = 4/1. Then $LiOH_{(aq.)}$ (1.55 mL, 37 mg, 1 M, 6 equiv.) was added to the reaction mixture. The mixture was left stirring overnight at 50 °C. After all starting material was consumed, the solvent was evaporated *in vacuo* and the crude was redissolved into EtOAc (20 ml) and 15 mL of water was added. The mixture was neutralised with 1 M HCl (to pH = 2-3) and water phase was washed with EtOAc (3x20 ml). Combined organic phases were dried over anhydrous Na_2SO_4, filtered and the solvent was evaporated *in vacuo*. Compound **11** was obtained without any further purification in 82 % yield (120 mg). ^{1}H NMR (400 MHz, DMSO-d_6) δ 8.19 (d, J = 0.9 Hz, 1H), 7.84 (dd, J = 8.4, 1.5 Hz, 1H), 7.79 (d, J = 2.1 Hz, 1H), 7.67 (d, J = 8.5 Hz, 1H), 7.56 (d, J = 8.4 Hz, 1H), 7.47 (dd, J = 8.5, 2.1 Hz, 1H), 6.02 (d, J = 5.4 Hz, 1H), 4.96 (ABq, J = 12.8 Hz, 2H), 4.38 – 4.27 (m, 2H), 4.22 (dd, J = 10.0, 5.4 Hz, 1H), 4.11 (s, 1H), 4.00 (t, J = 6.2 Hz, 1H), 3.93 (s, 3H), 3.58 (dd, 2H), 3.49 (t, J = 2.4 Hz, 1H), 3.39 (dd, J = 10.9, 6.4 Hz, 1H, this signal overlaps with water). ^{13}C NMR (101 MHz, DMSO-d_6) δ 154.5, 145.2, 142.4, 136.2, 135.8, 132.9, 131.8, 131.8, 131.1, 130.0, 123.4, 119.1, 112.5, 86.3, 80.5, 78.6, 78.1, 74.3, 72.9, 65.2, 63.9, 60.5, 57.4, 40.5, 30.7. HRMS calcd $C_{25}H_{25}O_7N_2Cl_2S + H^+$ $(M+H)^+$: 567.0754, found: 567.0755. HPLC purity: 98.90 %.

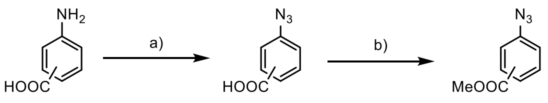

***Supplementary scheme 4**. a) $TMSN_3$, t-BuONO, CH_3CN or acetone, rt, 1h (75-81 %); b) H_2SO_4 (cat.), dry MeOH, 60 °C, overnight (66-74 %).*

General method for the preparation of azides **13a-c**

Aminobenzoic acids **12a-c** (1 equiv.) were dissolved in acetonitrile or acetone (4 mL). At 0°C, *t*-butyl nitrite (1.5 equiv.) was added, followed by the dropwise addition of trimethylsilyl azide (1.2 equiv.). The reaction mixture was cooled to room temperature and left stirring for 1 h. After all starting material was consumed, the solvent was evaporated *in vacuo*. The crude material was purified via flash column chromatography (mobile phase: n-hexane/EtOAc = 2/1 + 0.3 % glacial AcOH) to obtain azides **13a-c**. Products were stored in the dark at 4 °C due to the instability of aromatic azides.

a) Compound **13a**

Following the general procedure, the reaction was performed with **12a** (100 mg, 0.729 mmol, 1 equiv.), *t*-butyl nitrite (113 mg, 1.09 mmol, 1.5 equiv.), and of trimethylsilyl azide (101 mg, 0.875 mmol, 1.2 equiv.) in acetonitrile (4 mL) to obtain compound **13a** in 82 % yield (97 mg). ^{1}H NMR (400 MHz, $CDCl_3$) δ 8.14 (dd, J = 7.8, 1.4 Hz, 1H), 7.62 (ddd, J = 8.1, 7.4, 1.6 Hz, 1H), 7.31 – 7.24 (m, 2H).

b) Compound **13b**

Following the general procedure, the reaction was performed with **12b** (100 mg, 0.729 mmol, 1 equiv.), *t*-butyl nitrite (113 mg, 1.09 mmol, 1.5 equiv.), and of trimethylsilyl azide (101 mg, 0.875 mmol, 1.2 equiv.) in acetone to obtain compound **13b** in 76 % yield (90 mg). ^{1}H NMR (400 MHz, $CDCl_3$) δ 7.93 – 7.86 (m, 1H), 7.81 – 7.75 (m, 1H), 7.47 (t, J= 7.9 Hz, 1H), 7.29 – 7.24 (m, 1H).

c) Compound **13c**

Following the general procedure, the reaction was performed with **12c** (100 mg, 0.729 mmol, 1 equiv.), *t*-butyl nitrite (113 mg, 1.09 mmol, 1.5 equiv.), and of trimethylsilyl azide (101 mg, 0.875 mmol, 1.2 equiv.) in acetone to obtain compound **13c** in 76 % yield (90 mg). ^{1}H NMR (400 MHz, $CDCl_3$) d 8.11 (d, J = 8.8 Hz, 2H), 7.11 (d, J = 8.8 Hz, 2H).

General method for the preparation of methyl benzoates **14a-c**

Azides **13a-c** (1 equiv.) were dissolved in dry MeOH (2-3 mL). A catalytic amount of sulfuric acid was added, and the reaction mixture was left stirring overnight at 60 °C. After TLC showed that all starting material is consumed, reaction was quenched with saturated sodium bicarbonate solution (few drops). Afterwards, the mixture was extracted with EtOAc (40 mL) and organic phase was washed with water (3x20 mL), dried over anhydrous Na_2SO_4, filtered and the solvent was evaporated *in vacuo* to obtain **14a-c**. Products were stored in the dark at 4 °C due to the instability of aromatic azides.

a) Compound **14a**

Following the general procedure, the reaction was performed with **13a** (97 mg, 0.595 mmol, 1 equiv.) to give a compound **14a** as a solid in 66 % yield (70 mg). ^{1}H NMR (400 MHz, $CDCl_3$) δ 7.86 (dd, J = 7.8, 1.6 Hz, 1H), 7.53 (ddd, J = 8.1, 7.4, 1.6 Hz, 1H), 7.24 (dd, J = 8.1, 0.8 Hz, 1H), 7.18 (td, J = 7.8, 1.1 Hz, 1H), 3.91 (s, 3H).

b) Compound **14b**

Following the general procedure, the reaction was performed with **13b** (90 mg, 0.552 mmol, 1 equiv.) to give a compound **14b** as a solid in 70 % yield (68 mg). ^{1}H NMR (400 MHz, $CDCl_3$) δ 7.83 – 7.80 (m, 1H), 7.72 – 7.70 (m, 1H), 7.43 (t, J = 7.9 Hz, 1H), 7.20 (ddd, J = 8.0, 2.4, 1.0 Hz, 1H), 3.93 (s, 3H).

c) Compound **14c**

Following the general procedure, the reaction was performed with **13c** (120 mg, 0.735 mmol, 1 equiv.) to give a compound **14c** as a solid in 74 % yield (97 mg). ^{1}H NMR (400 MHz, $CDCl_3$) δ 8.06 – 8.00 (m, 2H), 7.11 – 7.04 (m, 2H), 3.91 (s, 3H)

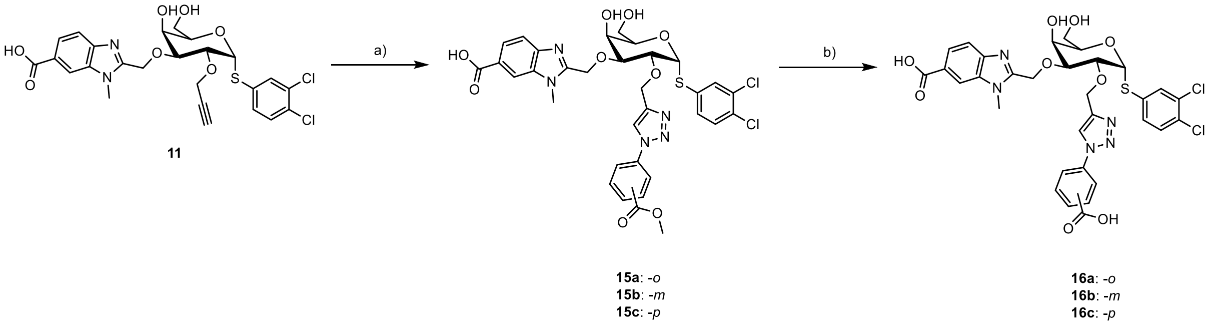

Supplementary scheme 5*. a)* ***14a-c****, $CuSO_4$*5H_2O, sodium ascorbate, MeOH/H_2O = 3/1, 40 °C, overnight (65-78 %); b) 1 M LiOH, EtOH/H_2O = 4/1, 50 °C, overnight (61-68 %).*

General method for the preparation of methyl benzoate – triazoles **15a-c**

Compound **11** (1 equiv.), methyl benzoates **14a-c** (1.2 equiv.), $CuSO_4*5H_2O$ (0.2 equiv.), and sodium ascorbate (0.4 equiv.) were suspended in $MeOH/H_2O$ = 3/1 (4-6 mL). The reaction mixture was left stirring overnight at 40 °C. After all starting material was consumed, the mixture was concentrated *in vacuo*. The crude material was purified via flash column chromatography (mobile phase: DCM/MeOH = 40/1) and reversed-phase Isolera to obtain compound **15a-c**.

a) Compound **15a**

Following the general procedure, the reaction was performed with compound **11** (70 mg, 0.123 mmol, 1 equiv.), **14a** (24 mg, 0.148 mmol, 1.2 equiv.), $CuSO_4*5H_2O$ (5 mg, 0.028 mmol, 0.2 equiv.), and sodium ascorbate (10 mg, 0.050 mmol, 0.4 equiv.) to obtain compound **15a** in 78 % yield (72 mg). ^{1}H NMR (400 MHz, Acetone-d_6) δ 8.42 (d, J = 2.6 Hz, 2H), 8.15 (dd, J = 8.6, 1.1 Hz, 1H), 8.10 (d, J = 8.6 Hz, 1H), 7.95 (dd, J = 7.7, 1.4 Hz, 1H), 7.83 – 7.76 (m, 2H), 7.71 (td, J = 7.6, 1.2 Hz, 1H), 7.61 (dd, J = 7.8, 0.9 Hz, 1H), 7.55 (dd, J = 8.4, 2.1 Hz, 1H), 7.49 (d, J = 8.4 Hz, 1H), 6.15 (d, J = 5.5 Hz, 1H), 5.64 (d, J = 15.1 Hz, 1H, this signal overlaps with water), 5.46 (d, J = 15.1 Hz, 1H), 5.08 (d, J = 12.4 Hz, 1H), 4.93 (d, J = 12.4 Hz, 1H), 4.51 (dd, J = 9.9, 5.5 Hz, 1H), 4.42 (d, J = 2.2 Hz, 1H), 4.29 (t, J = 5.7 Hz, 1H), 4.20 (s, 3H), 3.96 (dd, J = 9.9, 2.9 Hz, 1H), 3.77 (ddd, J = 17.5, 11.2, 5.8 Hz, 2H), 3.65 (s, 3H).^{13}C NMR (101 MHz, Acetone-d_6) δ 166.1, 165.5, 154.2, 144.2, 135.9, 135.6, 133.7, 133.1, 132.8, 131.9, 131.7, 130.8, 130.6, 130.4, 129.9, 127.8, 127.6, 126.4, 125.3, 116.0, 113.8, 86.6, 79.9, 75.5, 72.2, 67.0, 63.4, 62.6, 61.3, 51.9, 31.1, 29.7, 29.6. HRMS calcd $C_{33}H_{32}O_9N_5Cl_2S + H^+$ $(M+H)^+$: 744.1292, found: 744.1281. HPLC purity: 99.9 %.

b) Compound **15b**

Following the general procedure, the reaction was performed with compound **11** (75 mg, 0.132 mmol, 1 equiv.), **14b** (28 mg, 0.158 mmol, 1.2 equiv.), $CuSO_4*5H_2O$ (5 mg, 0.028 mmol, 0.2 equiv.), and sodium ascorbate (10.5 mg, 0.053 mmol, 0.4 equiv.) to obtain compound **15b** in 76 % yield (75 mg). ^{1}H NMR (400 MHz, Acetone-d_6) δ 8.63 (s, 1H), 8.24 (s, 1H), 8.21 (t, J = 1.8 Hz, 1H), 7.97 – 7.90 (m, 3H), 7.86 (d, J = 8.5 Hz, 1H), 7.63 (d, J = 2.0 Hz, 1H), 7.57 (t, J = 7.9 Hz, 1H), 7.39 (dd, J = 8.4, 2.1 Hz, 1H), 7.34 (d, J = 8.4 Hz, 1H), 5.97 (d, J = 5.5 Hz, 1H), 5.41 (d, J = 14.7 Hz, 1H), 5.22 (d, J = 14.7 Hz, 1H), 4.91 (d, J = 12.5 Hz, 1H), 4.76 (d, J = 12.5 Hz, 1H), 4.37 (dd, J = 9.9, 5.5 Hz, 1H, this signal overlaps with water), 4.23 (d, J = 2.2 Hz, 1H, this signal overlaps with water), 4.13 (t, 1H), 4.03 (s, 3H), 3.81 (s, 3H), 3.78 (dd, J = 9.9, 3.0

Hz, 1H), 3.68 – 3.58 (m, J = 11.3, 5.8 Hz, 2H). ^{13}C NMR (101 MHz, DMSO-d$_6$) δ 168.1, 165.7, 154.6, 145.7, 137.2, 136.0, 135.9, 132.6, 131.8, 131.7, 131.6, 131.02, 131.00, 129.9, 129.5, 125.6, 124.8, 123.7, 123.0, 120.6, 118.8, 112.8, 86.6, 78.8, 75.4, 72.9, 65.5, 64.0, 63.1, 60.5, 53.0, 39.5, 30.8. HRMS calcd $C_{33}H_{32}O_9N_5Cl_2S$ + H^+ $(M+H)^+$: 744.1292, found: 744.1280. HPLC purity: 96.3 %.

c) Compound **15c**

Following the general procedure, the reaction was performed with compound **11** (60 mg, 0.106 mmol, 1 equiv.), **14c** (22.5 mg, 0.127 mmol, 1.2 equiv.), $CuSO_4*5H_2O$ (4 mg, 0.023 mmol, 0.2 equiv.), and sodium ascorbate (8.5 mg, 0.043 mmol, 0.4 equiv.) to obtain compound **15c** in 65 % yield (51 mg). ^{1}H NMR (400 MHz, Acetone-d$_6$) δ 8.76 (s, 1H), 8.41 (s, 1H), 8.16 – 8.12 (m, 3H), 8.07 (d, J = 8.6 Hz, 1H), 7.96 (d, J = 8.8 Hz, 2H), 7.78 (d, J = 2.0 Hz, 1H), 7.54 (dd, J = 8.4, 2.0 Hz, 1H), 7.49 (d, J = 8.4 Hz, 1H), 6.14 (d, J = 5.4 Hz, 1H), 5.59 (d, J = 15.0 Hz, 1H), 5.42 (d, J = 14.9 Hz, 1H), 5.06 (d, J = 12.5 Hz, 1H), 4.91 (d, J = 12.6 Hz, 1H), 4.52 (dd, J = 9.9, 5.5 Hz, 1H), 4.40 (d, 1H), 4.27 (d, J = 5.7 Hz, 1H), 4.19 (s, 3H), 3.98 – 3.96 (m, J = 2.2 Hz, 1H), 3.95 (s, 3H), 3.84 – 3.72 (m, J = 17.4, 11.2, 5.7 Hz, 2H). ^{13}C NMR (101 MHz, Acetone-d$_6$) δ 166.8, 165.4, 154.6, 145.9, 143.0, 140.2, 135.9, 135.4, 132.9, 131.9, 131.5, 131.0, 130.5, 130.3, 129.9, 125.5, 124.1, 121.8, 119.7, 118.1, 112.6, 86.9, 79.4, 75.7, 72.2, 66.7, 64.2, 63.2, 61.2, 51.7, 30.2, 29.7, 29.6. HRMS calcd $C_{33}H_{32}O_9N_5Cl_2S$ + H^+ $(M+H)^+$: 744.1292, found: 744.1279. HPLC purity: 96.3 %.

General method for the preparation of **16a-c**

Compounds **15a-c** (1 equiv.) were dissolved in EtOH/H_2O = 4/1. Then $LiOH_{(aq.)}$ (1 M, 6 equiv.) was added to the reaction mixture. The mixture was left stirring overnight at 50 °C. After all the starting material was consumed, the mixture was concentrated *in vacuo*. The crude material was purified using reversed-phase Isolera (water/CH_3CN) to obtain compounds **16a-c**.

a) Compound **16a**

Following the general procedure, the reaction was performed with **15a** (30 mg, 0.040 mmol, 1 equiv.) and 1 M $LiOH_{(aq.)}$ (5.8 mg, 0.242 mmol, 6 equiv.) to obtain compound **16a** in 68 % yield (20 mg). ^{1}H NMR (400 MHz, CD_3CN) δ 8.11 (s, 1H), 7.96 (s, 1H), 7.80 (dd, J = 8.5, 1.2 Hz, 1H), 7.68 (d, J = 2.8 Hz, 1H), 7.66 (dd, 1H), 7.50 (d, J = 8.4 Hz, 2H), 7.45 – 7.38 (m, 3H), 7.22 (d, J = 7.4 Hz, 1H), 5.85 (d, J = 5.5 Hz, 1H), 4.93 – 4.85 (m, 2H), 4.83 (dd, 2H), 4.24 (dd, J = 7.8 Hz, 2H), 4.18 (t, J = 6.1 Hz, 1H), 3.75 (s, 3H), 3.71 – 3.61 (m, 3H). ^{13}C NMR (101

MHz, CD_3CN) δ 173.8, 172.8, 153.2, 144.3, 143.2, 136.6, 135.9, 135.2, 133.9, 133.8, 132.5, 132.4, 132.3, 131.3, 131.2, 130.3, 129.8, 129.6, 126.4, 125.7, 124.4, 118.2, 112.3, 87.3, 78.6, 75.5, 72.3, 66.3, 63.9, 63.5, 61.2, 30.6. HRMS calcd $C_{32}H_{30}O_9N_5Cl_2S + H^+$ $(M+H)^+$: 730.1136, found: 730.1124. HPLC purity: 98.4 %.

b) Compound **16b**

Following the general procedure, the reaction was performed with **15b** (25 mg, 0.034 mmol, 1 equiv.) and 1 M $LiOH_{(aq.)}$ (4.82 mg, 0.201 mmol, 6 equiv.) to obtain compound **16b** in 61 % yield (15 mg). ^1H NMR (400 MHz, CD_3CN) δ 8.36 (s, 1H), 8.15 – 8.13 (m, 1H), 7.97 (d, J = 0.9 Hz, 1H), 7.95 (dt, J = 7.8, 1.1 Hz, 1H), 7.79 (dd, J = 8.5, 1.5 Hz, 1H), 7.69 (t, J = 1.0 Hz, 1H), 7.64 (ddd, J = 8.0, 2.2, 1.0 Hz, 1H), 7.52 (d, J = 8.5 Hz, 1H), 7.48 (d, J = 7.9 Hz, 1H), 7.43 (d, 2H), 5.90 (d, J = 5.5 Hz, 1H), 4.95 – 4.84 (m, 3H), 4.77 (d, J = 12.6 Hz, 1H), 4.28 (dd, J = 10.1, 5.6 Hz, 1H), 4.26 (d, J = 2.5 Hz, 1H), 4.20 (t, J = 6.2 Hz, 1H), 3.76 (s, 3H), 3.71 (d, J = 3.1 Hz, 1H), 3.65 – 3.56 (m, 2H). ^{13}C NMR (101 MHz, CD_3CN) δ 173.1, 172.0, 153.5, 145.6, 143.6, 139.7, 136.7, 136.0, 135.3, 133.8, 132.5, 132.3, 131.5, 131.3, 131.2, 130.2, 130.0, 124.4, 123.2, 122.7, 121.4, 118.4, 112.3, 87.5, 78.6, 75.9, 72.3, 66.4, 64.0, 63.5, 61.3, 30.5. HRMS calcd $C_{32}H_{30}O_9N_5Cl_2S + H^+$ $(M+H)^+$: 730.1136, found: 730.1128. HPLC purity: 99.3 %.

c) Compound **16c**

Following the general procedure, the reaction was performed with **15c** (22 mg, 0.030 mmol, 1 equiv.) and 1 M $LiOH_{(aq.)}$ (4.25 mg, 0.177 mmol, 6 equiv.) to obtain compound **16c** in 65 % yield (14 mg). ^1H NMR (400 MHz, DMSO-d_6) δ 8.72 (s, 1H), 8.09 – 8.03 (m, 3H), 7.85 (d, J = 8.3 Hz, 1H), 7.79 (d, J = 2.0 Hz, 1H), 7.69 (d, J = 8.5 Hz, 2H), 7.55 (d, J = 8.4 Hz, 1H), 7.51 – 7.45 (m, 2H), 6.13 (d, J = 5.3 Hz, 1H), 5.48 (s, 1H), 4.98 (d, J = 12.3 Hz, 1H), 4.88 (d, J = 12.9 Hz, 2H), 4.77 (d, J = 12.7 Hz, 1H), 4.22 (dd, J = 9.9, 5.4 Hz, 1H), 4.17 (s, 1H), 4.01 (t, J = 6.3 Hz, 1H), 3.80 (s, 3H), 3.67 – 3.57 (m, 2H). ^{13}C NMR (101 MHz, DMSO-d_6) δ 168.1, 152.5, 145.5, 143.2, 141.4, 137.0, 136.1, 136.0, 132.8, 131.77, 131.75, 131.1, 131.0, 129.9, 126.5, 123.9, 122.6, 119.14, 119.1, 117.7, 111.5, 86.4, 78.5, 75.0, 74.96, 72.9, 65.0, 63.9, 63.1, 63.0, 60.4, 30.3. HRMS calcd $C_{32}H_{30}O_9N_5Cl_2S + H^+$ $(M+H)^+$: 730.1136, found: 730.1125. HPLC purity: 97.5 %.

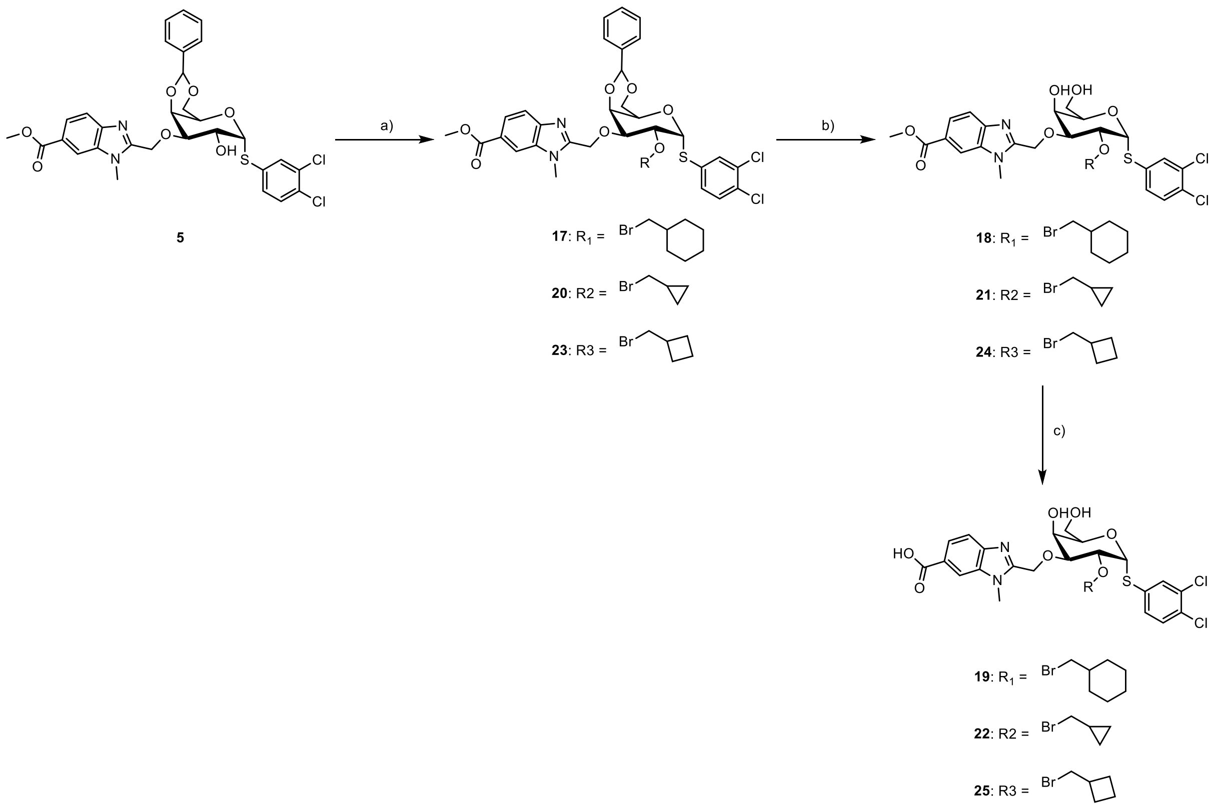

Supplementary scheme 6. *a) (bromomethyl)cyclohexane/(bromomethyl)cyclopropane/(bromomethyl)cyclobutane, Ag_2O, KI, dry DMF, rt, overnight (27-37 %); b) PTSA*H_2O, MeOH, H_2O, 40 °C, overnight (63-68 %); c) 1M LiOH, EtOH/H_2O = 4/1, 50 °C, overnight (78-80 %).*

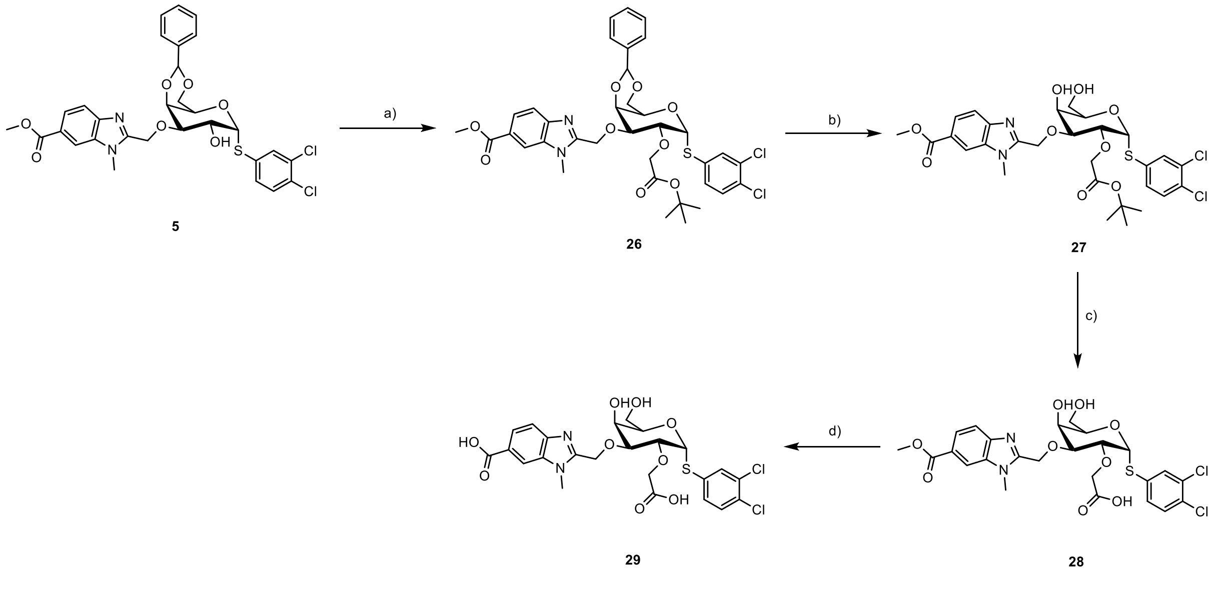

Supplementary scheme 7. *a) tert-butyl bromoacetate, Ag_2O, KI, dry DMF, rt, overnight (89 %); b) PTSA*H_2O, MeOH, H_2O, 40 °C, overnight (57 %); c) 4 M HCl/dioxane, dry dioxane, 40 °C, overnight (76 %); d) 1 M LiOH, EtOH/H_2O = 4/1, 50 °C, overnight (70 %).*

General method for preparation of compounds **17**, **20**, **23**, **26**

Compound **5** (1 equiv.) was dissolved in dry DMF (3-4 mL). Then, alkyl bromide (1.5 equiv.), Ag_2O (5 equiv.), and KI (2 equiv.) were added to the reaction mixture, which was left stirring

overnight at rt. The next day reaction mixture was diluted with EtOAc, filtered through Celite 545, and washed with EtOAc. Filtrate was washed with water (3x40 mL), dried over Na_2SO_4, filtered, and concentrated *in vacuo*. The crude material was purified via flash column chromatography (MP=n-hexane/EtOAc=1/2) to obtain compounds **17**, **20**, **23**, and **26**.

a) Compound **17**

Following the general procedure, the reaction was performed with **5** (206 mg, 0.326 mmol, 1 equiv.), (bromomethyl)cyclohexane (87 mg, 0.489 mmol, 68 µL, 1.5 equiv.), Ag_2O (378 mg, 1.631 mmol, 5 equiv.), and KI (108 mg, 0.652 mmol, 2 equiv.) to obtain compound **17** in 37 % yield (87 mg). ^1H NMR (400 MHz, $CDCl_3$) δ 7.99 (dd, 1H), 7.94 (d, J = 1.0 Hz, 1H), 7.76 (d, J = 8.4 Hz, 1H), 7.54 (d, J = 2.1 Hz, 1H), 7.34 – 7.22 (m, 7H), 5.94 (d, J = 5.4 Hz, 1H), 5.14 – 5.07 (m, 2H), 4.96 (d, J = 13.6 Hz, 1H), 4.32 (d, J = 3.5 Hz, 1H), 4.20 (dd, J = 10.0, 5.4 Hz, 1H), 4.09 (d, 1H), 3.97 (s, 3H), 3.96 (dd, 2H), 3.83 (dd, J = 10.1, 3.7 Hz, 1H), 3.81 (s, 3H), 3.45 (dd, J = 8.8, 6.9 Hz, 1H), 3.38 (dd, J = 8.8, 6.2 Hz, 1H), 1.88 – 1.63 (m, 6H), 1.62 – 1.53 (m, 1H), 1.22 – 1.12 (m, 2H), 0.97 – 0.88 (m, 2H).

b) Compound **20**

Following the general procedure, the reaction was performed with **5** (277 mg, 0.439 mmol, 1 equiv.), (bromomethyl)cyclopropane (64 µL, 88 mg, 0.658 mmol, 1.5 equiv.), Ag_2O (508 mg, 2.193 mmol, 5 equiv.), and KI (145 mg, 0.877 mmol, 2 equiv.) to obtain compound **20** in 36 % yield (108 mg). ^1H NMR (400 MHz, $CDCl_3$) δ 7.99 (d, J = 8.4 Hz, 1H), 7.93 (s, 1H), 7.75 (d, J = 8.5 Hz, 1H), 7.54 (s, 1H), 7.35 – 7.20 (m, 7H), 5.97 (d, J = 5.1 Hz, 1H), 5.12 (d, J = 13.2 Hz, 1H), 5.06 (s, 1H), 4.98 (d, J = 13.4 Hz, 1H), 4.37 (s, 1H), 4.32 – 4.25 (m, 1H), 4.10 (d, J = 12.6 Hz, 1H), 3.99 (dd, J = 9.7 Hz, 2H), 3.98 (s, 3H), 3.85 (dd, J = 13.7 Hz, 1H), 3.82 (s, 3H), 3.57 – 3.51 (m, 1H), 3.47 – 3.41 (m, 1H), 1.14 – 1.06 (m, 1H), 0.56 (d, J = 7.9 Hz, 2H), 0.27 – 0.23 (m, 2H).

c) Compound **23**

Following the general procedure, the reaction was performed with **5** (342 mg, 0.542 mmol, 1 equiv.), (bromomethyl)cyclobutane (68 µL, 121 mg, 0.812 mmol, 1.5 equiv.), Ag_2O (627 mg, 2.708 mmol, 5 equiv.), and KI (180 mg, 1.083 mmol, 2 equiv.) to obtain compound **23** in 27 % yield (104 mg). ^1H NMR (400 MHz, $CDCl_3$) δ 7.99 (dd, 1H), 7.95 (d, J = 1.0 Hz, 1H), 7.75 (d, J = 8.5 Hz, 1H), 7.54 (d, J = 2.1 Hz, 1H), 7.37 – 7.23 (m, 7H), 5.96 (d, J = 5.4 Hz, 1H), 5.10 (t, J = 6.8 Hz, 2H), 4.96 (d, J = 13.6 Hz, 1H), 4.34 (d, J = 3.3 Hz, 1H), 4.26 – 4.20 (m, 1H),

4.09 (dd, J = 8.1, 4.5 Hz, 1H), 3.97 (s, 3H), 3.97 (dd, 2H), 3.82 (dd, 1H), 3.81 (s, 3H), 3.66 (dd, J = 9.1, 6.9 Hz, 1H), 3.55 (dd, J = 9.1, 6.7 Hz, 1H), 2.65 – 2.52 (m, 1H), 2.10 – 2.01 (m, 1H), 1.97 – 1.71 (m, 5H).

d) Compound **26**

Following the general procedure, the reaction was performed with **2** (306 mg, 0.485 mmol, 1 equiv.), *tert*-butyl bromoacetate (107 µL, 142 mg, 0.727 mmol, 1.5 equiv.), Ag_2O (561 mg, 2.42 mmol, 5 equiv.), and KI (161 mg, 0.969 mmol, 2 equiv.) to obtain compound **26** in 89 % yield (320 mg). ^{1}H NMR (400 MHz, $CDCl_3$) δ 7.99 (dd, J = 8.5, 1.5 Hz, 1H), 7.91 (d, J = 1.0 Hz, 1H), 7.76 (d, J = 8.5 Hz, 1H), 7.58 (d, J = 1.9 Hz, 1H), 7.35 – 7.27 (m, 5H), 7.17 (dd, J = 8.1, 1.4 Hz, 2H), 6.12 (d, J = 5.4 Hz, 1H), 5.13 (d, J = 13.6 Hz, 1H), 4.96 (t, J = 6.8 Hz, 2H), 4.35 (d, J = 3.0 Hz, 1H), 4.25 (d, J = 2.3 Hz, 2H), 4.09 (dd, 2H), 4.02 (d, 1H), 3.98 (dd, 1H), 3.98 (s, 3H), 3.91 (dd, J = 10.0, 3.6 Hz, 1H), 3.78 (s, 3H), 1.47 (s, 9H).

General method for preparation of compounds **18**, **21**, **24, 27**

Compounds **17, 20, 23**, and **26** (1 equiv.) were dissolved in MeOH (1 mL/10 mg). *p*-toluenesulfonic acid monohydrate (2.1 equiv.) was added to the reaction mixture, followed by addition of water (1-2 mL). The mixture was left stirring overnight at 40 °C. After all starting material was consumed, MeOH was evaporated, and the crude material was redissolved in EtOAc (50 mL). Afterwards, the organic phase was washed with $NaHCO_{3(sat.)}$ (3 x 30 mL), dried over anhydrous Na_2SO_4, filtered, and concentrated *in vacuo*. The crude material was purified via flash column chromatography (mobile phase: DCM/MeOH = 19/1) to obtain compounds **18, 21, 24** and **27** as a white solid.

a) Compound **18**

Following the general procedure, the reaction was performed with **17** (87 mg, 0.120 mmol, 1 equiv.), 9 mL of MeOH, and *p*-toluenesulfonic acid monohydrate (48 mg, 0.251 mmol, 2.1 equiv.) to obtain compound **18** in 67 % yield (51 mg). ^{1}H NMR (400 MHz, $CDCl_3$) δ 8.09 (d, J = 1.0 Hz, 1H), 7.98 (dd, J = 8.5, 1.5 Hz, 1H), 7.73 (d, J = 8.5 Hz, 1H), 7.58 (d, J = 2.0 Hz, 1H), 7.35 (d, J = 8.4 Hz, 1H), 7.31 (dd, J = 8.4, 2.0 Hz, 1H), 5.87 (d, J = 5.5 Hz, 1H), 5.27 (s, 1H), 5.19 (d, J = 14.1 Hz, 1H), 4.88 (d, J = 14.1 Hz, 1H), 4.29 – 4.23 (m, 2H), 4.19 (dd, J = 9.8, 5.5 Hz, 1H), 3.97 (s, 3H), 3.92 (dd, J = 2.2 Hz, 1H), 3.84 (s, 3H), 3.73 (dd, J = 9.8, 3.1 Hz, 1H), 3.48 (dd, J = 8.9, 6.8 Hz, 1H), 3.35 (dd, J = 8.9, 6.3 Hz, 1H), 3.14 (d, J = 4.8 Hz, 1H), 1.83 – 1.64 (m, 6H), 1.30 – 1.12 (m, 3H), 1.00 – 0.87 (m, 2H). ^{13}C NMR (101 MHz, $CDCl_3$) δ

167.3, 154.1, 145.1, 135.6, 134.3, 132.9, 131.5, 130.7, 130.6, 125.1, 124.0, 119.4, 111.8, 86.8, 80.1, 77.2, 76.4, 75.3, 70.3, 68.2, 64.6, 63.4, 52.3, 38.4, 30.2, 30.1, 30.0, 26.5, 25.8, 25.8. HRMS calcd for $C_{30}H_{36}O_7N_2Cl_2S + H^+$ $(M+H)^+$: 639.1693, found: 639.1684. HPLC purity: 96.32 %.

b) Compound **21**

Following the general procedure, the reaction was performed with **20** (108 mg, 0.157 mmol, 1 equiv.), 11 mL of MeOH, and *p*-toluenesulfonic acid monohydrate (63 mg, 0.331 mmol, 2.10 equiv.) to obtain compound **21** in 63 % yield (59 mg). ^{1}H NMR (400 MHz, $CDCl_3$) δ 8.07 (d, J = 1.0 Hz, 1H), 7.97 (dd, J = 8.5, 1.5 Hz, 1H), 7.71 (d, J = 8.5 Hz, 1H), 7.58 (d, J = 1.9 Hz, 1H), 7.36 – 7.29 (m, 2H), 5.89 (d, J = 5.5 Hz, 1H), 5.42 (s, 1H), 5.19 (d, J = 13.9 Hz, 1H), 4.90 (d, J = 13.9 Hz, 1H), 4.32 (d, J = 1.6 Hz, 1H), 4.26 (dd, J = 9.8, 5.4 Hz, 2H), 3.97 (s, 3H), 3.92 (dd, J = 3.8 Hz, 1H), 3.85 (s, 3H), 3.74 (dd, J = 9.8, 3.1 Hz, 1H), 3.53 – 3.39 (m, 3H), 1.14 – 1.06 (m, 1H), 0.61 – 0.48 (m, 2H), 0.31 – 0.19 (m, 2H).^{13}C NMR (101 MHz, $CDCl_3$) δ 167.3, 153.9, 144.9, 135.5, 134.3, 132.8, 132.8, 131.4, 130.6, 125.1, 123.9, 119.3, 111.8, 86.9, 79.7, 77.2, 75.2, 74.9, 70.5, 67.8, 64.4, 62.9, 52.3, 30.2, 10.9, 3.6, 3.1. HRMS calcd for $C_{27}H_{30}O_7N_2Cl_2S + H^+$ $(M+H)^+$: 597.1224, found: 597.1210. HPLC purity: 99.59 %.

c) Compound **24**

Following the general procedure, the reaction was performed with **23** (108 mg, 0.154 mmol, 1 equiv.), 11 mL of MeOH, and *p*-toluenesulfonic acid monohydrate (62 mg, 0.324 mmol, 2.1 equiv.) to obtain compound **24** in 68 % yield (64 mg). ^{1}H NMR (400 MHz, $CDCl_3$) δ 8.05 (d, J = 1.0 Hz, 1H), 7.96 (dd, 1H), 7.69 (dd, J = 8.5 Hz, 1H), 7.57 (d, J = 1.8 Hz, 1H), 7.34 (d, J = 8.3 Hz, 1H), 7.30 (dd, J = 8.4, 1.9 Hz, 1H), 5.88 (d, J = 5.5 Hz, 1H), 5.36 (s, 1H), 5.14 (d, J = 13.7 Hz, 1H), 4.89 (d, J = 13.8 Hz, 1H), 4.32 (d, J = 1.9 Hz, 1H), 4.28 – 4.19 (m, J = 10.1, 5.2 Hz, 2H), 3.96 (s, 3H), 3.93 (dd, J = 11.5, 4.8 Hz, 1H), 3.82 (s, 3H), 3.72 – 3.66 (m, 3H), 3.50 (dd, J = 9.1, 6.8 Hz, 1H), 2.64 – 2.52 (m, 1H), 2.10 – 1.99 (m, 2H), 1.95 – 1.84 (m, 2H), 1.82 – 1.73 (m, 2H). ^{13}C NMR (101 MHz, $CDCl_3$) δ 167.3, 153.8, 144.9, 135.4, 134.4, 132.8, 132.8, 131.3, 130.6, 130.6, 125.0, 123.9, 119.2, 111.8, 86.8, 79.4, 77.2, 75.4, 74.7, 70.6, 67.6, 64.4, 62.5, 52.3, 35.1, 30.2, 25.0, 18.6. HRMS calcd for $C_{28}H_{33}O_7N_2Cl_2S + H^+$ $(M+H)^+$: 611.1380, found: 611.1365. HPLC purity: 98.84 %.

d) Compound **27**

Following the general procedure, the reaction was performed with **26** (320 mg, 0.429 mmol, 1 equiv.), 20 mL of MeOH, and *p*-toluenesulfonic acid monohydrate (171 mg, 0.901 mmol, 2.1 equiv.) to obtain compound **27** in 57 % yield (160 mg). ^{1}H NMR (400 MHz, $CDCl_3$) δ 8.03 (d, J = 1.0 Hz, 1H), 7.93 (dd, J = 8.5, 1.5 Hz, 1H), 7.67 (d, J = 8.5 Hz, 1H), 7.60 (d, J = 1.8 Hz, 1H), 7.36 – 7.29 (m, 2H), 6.00 (d, J = 5.5 Hz, 1H), 5.82 (s, 1H), 5.22 (d, J = 13.8 Hz, 1H), 4.89 (d, J = 13.9 Hz, 1H), 4.37 (d, J = 2.0 Hz, 1H), 4.32 – 4.28 (m, 2H), 4.26 (d, J = 10.0 Hz, 1H), 4.14 (d, J = 16.4 Hz, 1H), 3.96 (s, 3H), 3.92 (dd, J = 11.5, 5.1 Hz, 2H), 3.82 (s, 3H), 3.77 (dd, J = 9.8, 3.0 Hz, 1H), 1.43 (s, 9H). ^{13}C NMR (101 MHz, $CDCl_3$) δ 169.1, 167.3, 154.0, 144.8, 135.4, 134.2, 132.8, 132.8, 131.4, 130.7, 130.6, 125.0, 124.0, 119.2, 111.8, 87.2, 82.0, 80.7, 77.3, 76.4, 70.8, 68.9, 67.3, 64.0, 62.4, 53.5, 52.3, 30.2, 28.1. HRMS calcd for $C_{29}H_{34}O_9N_2Cl_2S$ + H+ $(M+H)^+$: 657.1435, found: 657.1421. HPLC purity: 99.60 %.

Synthesis of Compound **28**

Compound **27** (140 mg, 0.213 mmol, 1 equiv.) was dissolved in dry dioxane (8 mL). 4 M HCl in dioxane (4.52 mL, 18.10 mmol, 85 equiv.) was added to the reaction mixture, which was left stirring overnight at 40 °C. After TLC showed complete consumption of starting material, the mixture was co-evaporated with Et_2O and concentrated *in vacuo*. The crude material was purified using reversed-phase Isolera to obtain compound **28** as a white solid in 76 % yield (97 mg). ^{1}H NMR (400 MHz, DMSO-d_6) δ 8.20 (s, 1H), 7.85 (d, J = 8.4 Hz, 1H), 7.77 (d, J = 17.3 Hz, 2H), 7.54 (d, J = 8.5 Hz, 1H), 7.46 (d, J = 8.2 Hz, 1H), 6.18 (d, J = 4.6 Hz, 1H), 5.37 (s, 1H), 5.07 (d, J = 12.0 Hz, 1H), 4.99 (d, J = 13.1 Hz, 1H), 4.17 (dd, J = 3.1, 1.1 Hz, 2H), 3.97 (dd, J = 14.6, 1.5 Hz, 2H), 3.92 (s, 3H), 3.89 (s, 3H), 3.77 (dd, J = 16.2, 4.8 Hz, 1H), 3.60 (dd, J = 11.2, 3.3 Hz, 2H). ^{13}C NMR (101 MHz, DMSO-d_6) δ 167.1, 155.3, 145.3, 140.7, 136.6, 136.0, 132.2, 131.8, 131.2, 131.0, 129.7, 125.4, 124.2, 123.3, 119.5, 112.5, 79.5, 75.0, 73.0, 64.7, 60.4, 52.5, 40.7, 30.9, 30.7. HRMS calcd for $C_{25}H_{26}O_9N_2Cl_2S + H^+$ $(M+H)^+$: 601.0809, found: 601.0798. HPLC purity: 99.23 %.

General procedure for compounds **19**, **22**, **25, 29**

Compounds **18**, **21**, **24** and **28** (1 equiv.) were dissolved in EtOH/H_2O = 4/1. Then $LiOH_{(aq.)}$ (1 M, 6 equiv.) was added to the reaction mixture. The mixture was left stirring overnight at 50 °C. After all starting material was consumed, the mixture was concentrated *in vacuo* and the crude was redissolved into EtOAc and water was added. The mixture was neutralised with 1M HCl (to pH = 3) and water phase was washed with EtOAc (3x20 ml). Combined organic phases were dried over anhydrous Na_2SO_4, filtered and the solvent was evaporated in vacuo.

a) Compound **19**

Following the general procedure, the reaction was performed with **18** (32 mg, 0.050 mmol, 1 equiv.) and 1 M $LiOH_{(aq.)}$ (7.2 mg, 0.300 mmol, 6 equiv.) to obtain compound **19** in 80 % yield (25 mg). ^{1}H NMR (400 MHz, DMSO-d_6) δ 8.21 (d, J = 1.0 Hz, 1H), 7.83 (dd, J = 8.5, 1.6 Hz, 1H), 7.75 (d, J = 2.1 Hz, 1H), 7.69 (d, J = 8.5 Hz, 1H), 7.56 (d, J = 8.5 Hz, 1H), 7.44 (dd, J = 8.5, 2.1 Hz, 1H), 6.05 (d, J = 5.4 Hz, 1H), 5.12 (d, J = 4.8 Hz, 1H), 4.96 (dd, J = 26.7, 12.8 Hz, 2H), 4.66 (t, J = 5.2 Hz, 1H), 4.07 (d, 1H), 3.96 (dd, J = 8.7, 4.3 Hz, 2H), 3.93 (s, 3H), 3.55 (dd, J = 10.0, 2.9 Hz, 2H), 3.44 (dd, J = 9.0, 6.9 Hz, 1H), 3.23 (dd, J = 9.1, 6.1 Hz, 1H), 1.71 – 1.55 (m, 5H), 1.51 – 1.40 (m, 1H), 1.19 – 1.05 (m, 2H), 0.94 – 0.81 (m, 2H). ^{13}C NMR (101 MHz, DMSO-d_6) δ 168.2, 154.7, 145.4, 136.3, 136.2, 132.7, 131.7, 131.6, 131.0, 129.8, 125.3, 123.3, 119.2, 112.6, 86.3, 78.6, 75.7, 75.3, 72.8, 65.3, 64.1, 60.5, 38.3, 30.6, 30.0, 29.8, 26.6, 25.8, 25.7. HRMS calcd for $C_{29}H_{34}O_7N_2Cl_2S + H^+$ $(M+H)^+$: 625.1537, found: 625.1520. HPLC purity: 98.96 %.

b) Compound **22**

Following the general procedure, the reaction was performed with **21** (43 mg, 0.072 mmol, 1 equiv.) and 1 M $LiOH_{(aq.)}$ (10 mg, 0.432 mmol, 6 equiv.) to obtain compound **22** in 79 % yield (33 mg). ^{1}H NMR (400 MHz, DMSO-d_6) δ 12.84 (s, 1H), 8.21 (d, J = 1.0 Hz, 1H), 7.84 (dd, J = 8.5, 1.5 Hz, 1H), 7.77 (d, J = 2.1 Hz, 1H), 7.69 (d, J = 8.4 Hz, 1H), 7.56 (d, J = 8.4 Hz, 1H), 7.45 (dd, J = 8.5, 2.1 Hz, 1H), 6.06 (d, J = 5.4 Hz, 1H), 5.14 (s, 1H), 4.99 (q, J = 12.7 Hz, 2H), 4.67 (s, 1H), 4.09 (s, 1H), 4.04 (dd, J = 10.0, 5.5 Hz, 1H), 3.97 (dd, J = 7.1 Hz, 1H), 3.96 (s, 3H), 3.56 (dd, J = 9.9, 3.0 Hz, 2H), 3.45 – 3.37 (m, 3H), 1.07 – 0.91 (m, 1H), 0.51 – 0.39 (m, 2H), 0.23 – 0.14 (m, 2H). ^{13}C NMR (101 MHz, DMSO-d_6) δ 168.2, 154.7, 145.3, 136.3, 136.2, 132.7, 131.7, 131.6, 131.0, 129.8, 125.3, 123.4, 119.2, 112.7, 86.4, 78.7, 75.1, 74.1, 72.8, 65.2, 64.1, 60.5, 30.7, 11.2, 3.5, 3.4. HRMS calcd for $C_{26}H_{28}O_7N_2Cl_2S + H^+$ $(M+H)^+$: 583.1067, found: 583.1054. HPLC purity: 97.99 %.

c) Compound **25**

Following the general procedure, the reaction was performed with **24** (30 mg, 0.049 mmol, 1 equiv.) and 1 M $LiOH_{(aq.)}$ (7 mg, 0.294 mmol, 6 equiv.) to obtain compound **25** in 78 % yield (23 mg). ^{1}H NMR (400 MHz, DMSO-d_6) δ 12.84 (s, 1H), 8.22 (d, J = 1.0 Hz, 1H), 7.83 (dd, J = 8.5, 1.5 Hz, 1H), 7.76 (d, J = 2.1 Hz, 1H), 7.69 (d, J = 8.4 Hz, 1H), 7.56 (d, J = 8.5 Hz, 1H), 7.45 (dd, J = 8.5, 2.1 Hz, 1H), 6.08 (d, J = 5.4 Hz, 1H), 5.12 (s, 1H), 4.98 (q, J = 12.8 Hz, 2H), 4.66 (s, 1H), 4.07 (d, 1H), 4.00 (dd, J = 10.0, 5.5 Hz, 1H), 3.96 (d, 1H), 3.93 (s, 3H), 3.66 (dd,

J = 9.4, 6.8 Hz, 1H), 3.55 (dd, J = 9.9, 3.3 Hz, 2H), 3.44 – 3.38 (m, 2H), 1.98 – 1.68 (m, 7H). ^{13}C NMR (101 MHz, DMSO-d$_6$) δ 168.2, 154.7, 136.23, 136.17, 132.7, 131.8, 131.6, 131.0, 129.8, 125.4, 123.4, 119.2, 112.7, 86.33, 86.31, 78.6, 75.7, 73.9, 72.8, 65.3, 64.2, 60.5, 35.1, 30.6, 24.9, 24.8, 18.5. HRMS calcd for $C_{27}H_{30}O_7N_2Cl_2S$+ H^+ (M+H)+: 597.1224, found: 597.1209. HPLC purity: 98.87 %.

d) Compound **29**

Following the general procedure, the reaction was performed with **28** (50 mg, 0.083 mmol, 1 equiv.) and 1 M $LiOH_{(aq.)}$ (12 mg, 0.499 mmol, 6 equiv.). The mixture was concentrated *in vacuo* and the crude material was purified using reversed-phase Isolera (water/CH_3CN) to obtain compound **29** in 70 % yield (34 mg). ^{1}H NMR (400 MHz, D_2O) δ 7.87 (d, 1H), 7.70 (d, J = 8.5 Hz, 1H), 7.52 – 7.44 (m, 2H), 7.22 (d, J = 1.7 Hz, 2H), 5.78 (d, J = 5.1 Hz, 1H), 4.93 (t, 2H), 4.14 (t, 1H), 4.11 – 4.03 (m, 2H), 3.97 (dd, J = 10.0, 5.5 Hz, 1H), 3.91 – 3.85 (m, 1H), 3.75 (s, 3H), 3.69 (d, J = 10.0 Hz, 1H), 3.58 – 3.48 (m, 2H). ^{13}C NMR (101 MHz, D_2O) δ 177.1, 174.8, 152.3, 140.7, 134.8, 134.2, 132.3, 132.3, 131.9, 131.5, 130.5, 124.2, 117.1, 111.7, 86.7, 78.3, 75.2, 71.8, 69.4, 66.1, 62.9, 60.8, 38.7, 30.1. HRMS calcd for $C_{24}H_{24}O_9N_2Cl_2S$ + H^+ (M+H)+: 587.0652, found: 587.0642. HPLC purity: 99.69 %.

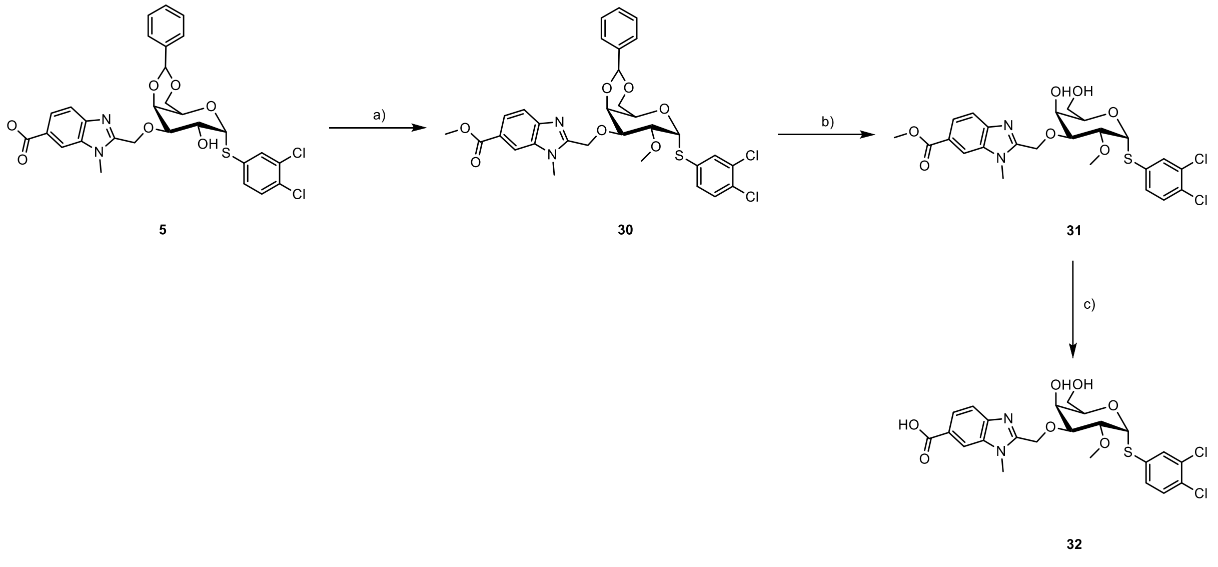

Supplementary scheme 8. a) MeI, NaH, 15-crown-5, dry DMF, rt, 4h (84 %); b) PTSAH_2O, MeOH, H_2O, 40 °C, overnight (79 %); c) 1 M LiOH, EtOH/H_2O = 4/1, 50 °C, overnight (72 %).*

Synthesis of Compound **30**

Compound **5** (340 mg, 0.559 mmol, 1 equiv.) was dissolved in dry THF (5 mL). NaH (36 mg, 0.894 mmol, 1.60 equiv., 60 %), 15-crown-5 (492 mg, 2.24 mmol, 4 equiv.), and methyl iodide (143 mg, 1.01 mmol, 1.80 equiv.) were added to the reaction mixture, which was left stirring

for 4 h at room temperature. After TLC showed all starting material was consumed, the mixture was quenched with MeOH (2 mL) and diluted with EtOAc (80 mL). The organic phase was washed with $NaHCO_{3\ (sat.)}$ (3x40 mL), dried over anhydrous Na_2SO_4, filtered and concentrated *in vacuo*. The crude material was purified via flash column chromatography (mobile phase: n-hexane/EtOAc =1/2) to obtain **30** as an oil in 84 % yield (304 mg). ^1H NMR (400 MHz, $CDCl_3$) δ 7.99 (dd, J = 8.5, 1.6 Hz, 1H), 7.88 (d, J = 1.0 Hz, 1H), 7.75 (d, J = 8.5 Hz, 1H), 7.56 (d, J = 2.1 Hz, 1H), 7.35 – 7.25 (m, 5H), 7.17 (dd, J = 8.1, 1.5 Hz, 2H), 5.98 (d, J = 5.4 Hz, 1H), 5.15 (d, J = 13.7 Hz, 1H), 4.94 (dd, J = 6.8 Hz, 2H), 4.35 (d, J = 2.9 Hz, 1H), 4.16 (dd, J = 10.0, 5.4 Hz, 1H), 4.09 (dd, J = 8.8, 4.2 Hz, 1H), 4.01 (d, 1H), 3.98 (s, 3H), 3.86 (dd, J = 10.0, 3.6 Hz, 1H), 3.78 (s, 3H), 3.54 (s, 3H).

Synthesis of Compound **31**

Compound **30** (304 mg, 0.471 mmol, 1 equiv.) was dissolved in MeOH (22 mL). *p*-toluenesulfonic acid monohydrate (188 mg, 0.989 mmol, 2.1 equiv.) was added to the reaction mixture, followed by addition of water (2 mL). The mixture was left stirring overnight at 40 °C. After all starting material was consumed, MeOH was evaporated, and the crude material was redissolved in EtOAc (50 mL). The organic phase was washed with $NaHCO_{3\ (sat.)}$ (3x30 mL), dried over anhydrous Na_2SO_4, filtered and concentrated *in vacuo*. The crude material was purified via flash column chromatography (mobile phase: DCM/MeOH = 19/1) to obtain compound **31** in 79 % yield (208 mg). ^1H NMR (400 MHz, $CDCl_3$) δ 8.11 (d, J = 1.0 Hz, 1H), 7.99 (dd, J = 8.5, 1.5 Hz, 1H), 7.73 (d, J = 8.5 Hz, 1H), 7.60 (d, J = 1.8 Hz, 1H), 7.39 – 7.29 (m, 2H), 5.90 (d, J = 5.4 Hz, 1H), 5.21 (d, J = 14.2 Hz, 1H), 4.86 (d, J = 14.3 Hz, 1H), 4.28 (dd, J = 6.7, 3.3 Hz, 2H), 4.16 (dd, J = 9.9, 5.4 Hz, 1H), 3.97 (s, 3H), 3.96 (dd, 1H), 3.85 (dd, 1H), 3.85 (s, 3H), 3.73 (dd, J = 9.9, 3.1 Hz, 1H), 3.53 (s, 3H). ^{13}C NMR (101 MHz, $CDCl_3$) δ 167.3, 153.9, 144.8, 135.5, 134.1, 132.9, 132.9, 131.6, 130.72, 130.65, 125.2, 124.0, 119.3, 111.8, 86.5, 80.1, 77.2, 70.6, 67.7, 64.3, 62.9, 58.1, 52.3, 30.1. HRMS calcd for $C_{24}H_{26}O_7N_2Cl_2S + H^+$ $(M+H)^+$: 557.0911, found: 557.0899. HPLC purity: 98.80 %.

Synthesis of Compound **32**

Compounds **31** (128 mg, 0.230 mmol, 1 equiv.) were dissolved in EtOH/H_2O = 4/1. Then 1 M $LiOH_{(aq.)}$ (33 mg, 1.38 mmol, 6 equiv.) was added to the reaction mixture. The mixture was left stirring overnight at 50 °C. After all starting material was consumed, the solvent was evaporated *in vacuo* and the crude was redissolved into EtOAc (40 ml) and 20 mL of water was added. The mixture was neutralized with 1M HCl (to pH = 3) and water phase was washed

with EtOAc (3x20 ml). Combined organic phases were dried over anhydrous Na_2SO_4, filtered, and the solvent was evaporated in vacuo. Compound **32** was obtained without any further purification in 72 % yield (90 mg). ^1H NMR (400 MHz, DMSO-d_6) δ 12.87 (s, 1H), 8.22 (d, J = 1.0 Hz, 1H), 7.85 (dd, J = 8.5, 1.5 Hz, 1H), 7.79 (d, J = 2.1 Hz, 1H), 7.70 (d, J = 8.7 Hz, 1H), 7.56 (d, J = 8.5 Hz, 1H), 7.47 (dd, J = 8.5, 2.1 Hz, 1H), 6.08 (d, J = 5.4 Hz, 1H), 5.17 (s, 1H), 4.97 (dd, J = 27.0, 12.9 Hz, 3H), 4.67 (s, 1H), 4.09 (d, J = 2.6 Hz, 1H), 3.98 (t, J = 6.3 Hz, 1H), 3.93 (s, 3H), 3.90 (dd, 1H), 3.59 – 3.53 (m, 2H), 3.37 (s, 3H). ^{13}C NMR (101 MHz, DMSO-d_6) δ 168.2, 154.7, 136.1, 136.0, 132.8, 131.8, 131.7, 131.1, 129.9, 125.5, 123.5, 119.1, 112.8, 86.1, 78.9, 77.0, 72.8, 65.3, 63.9, 60.5, 57.9, 30.9, 30.6. HRMS calcd for $C_{23}H_{24}O_7N_2Cl_2S + H^+$ $(M+H)^+$: 543.0754, found: 543.0742. HPLC purity: 99.54 %.

The Consortium for Functional Glycomics provided the tetrasaccharide (LNnT) for the synthesis of the Gal-8N probe. LNnT with 2-azidoethyl linker was converted to free amine in a Staudinger reaction and later coupled with 5-carboxyfluorescein. This probe is generally used in a competitive fluorescence polarisation assay to determine the binding affinities for Gal-8N inhibitors[1,2]. The structure and synthesis of the LNnT probe are shown below (Supplementary scheme 9).

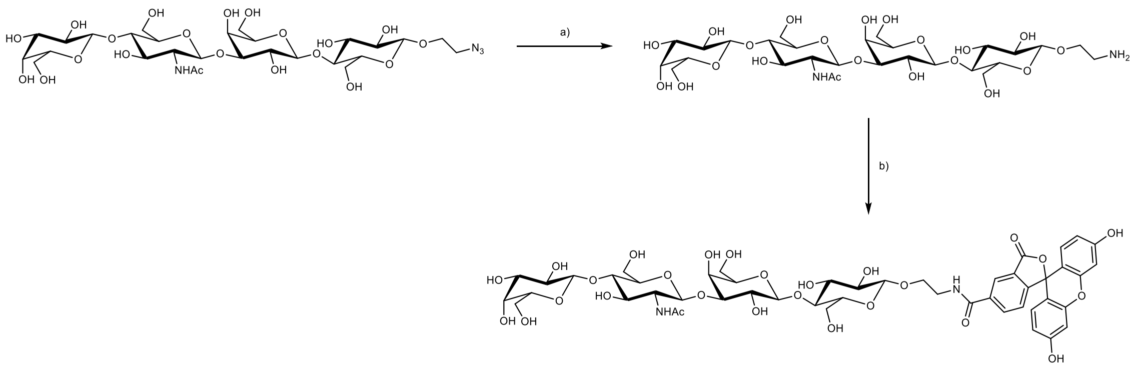

***Supplementary scheme 9**. Reagents and conditions: a) H_2, Pd/C, MeOH, rt, overnight; b) 5-carboxyfluorescein, BOP, HOBt, DIPEA, DMSO, rt, overnight[3].*

^1H, ^{13}C, ^{19}F NMR and HPLC chromatograms

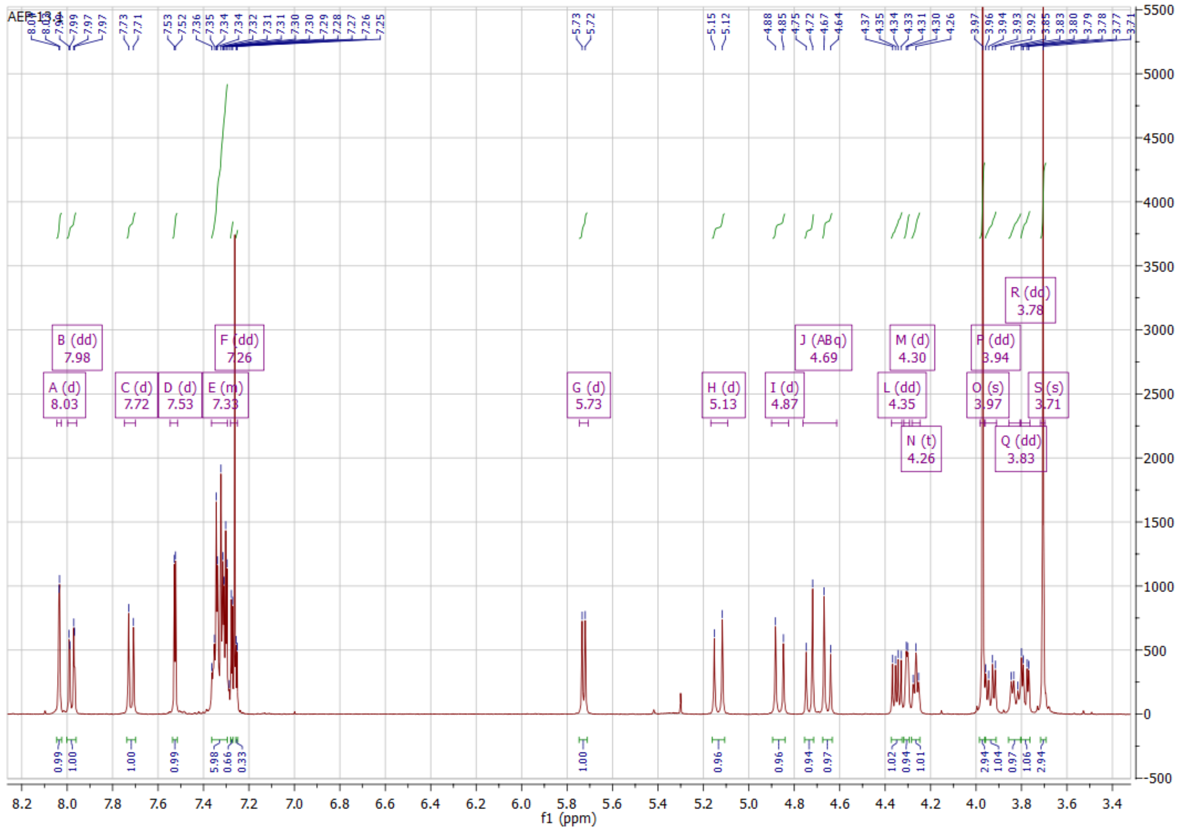

***Supplementary Fig. 1**. ^1H NMR (400 MHz, $CDCl_3$) of **7a**.*

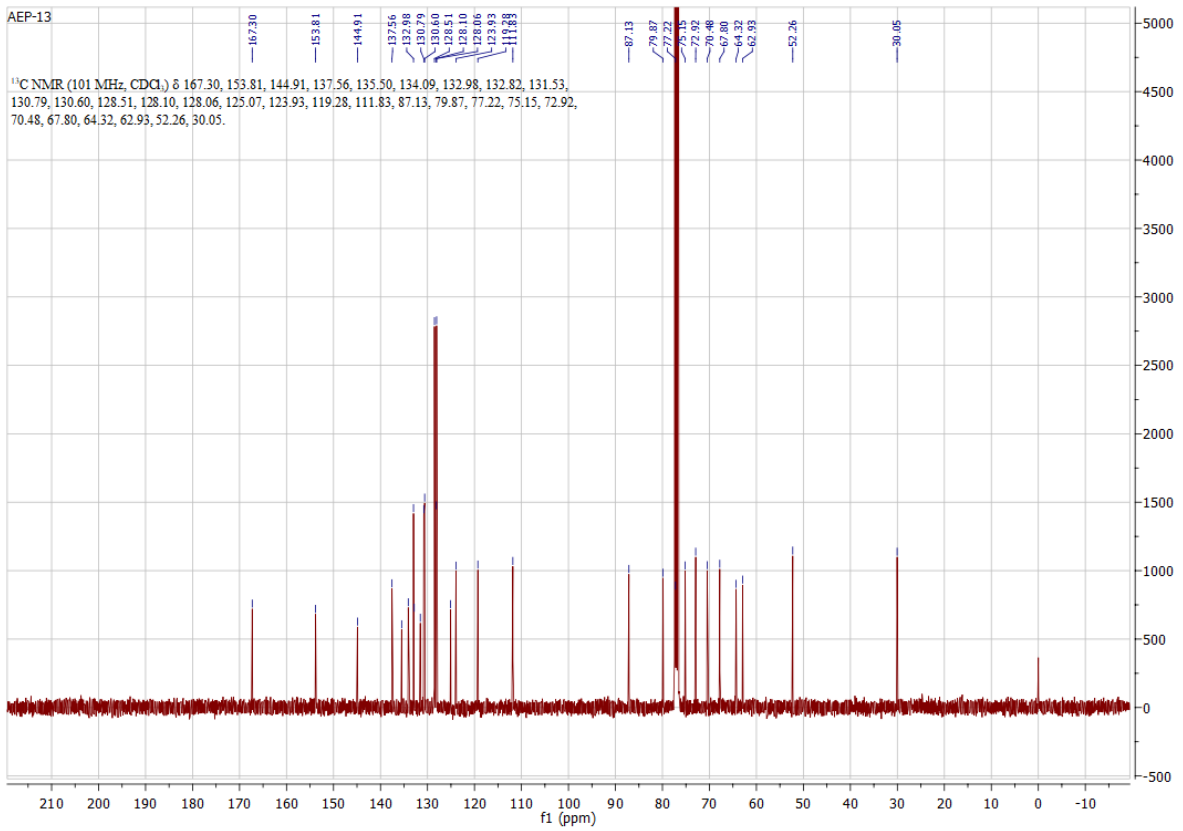

Supplementary Fig. 2. ^{13}C NMR (101 MHz, $CDCl_3$) of **7a**.

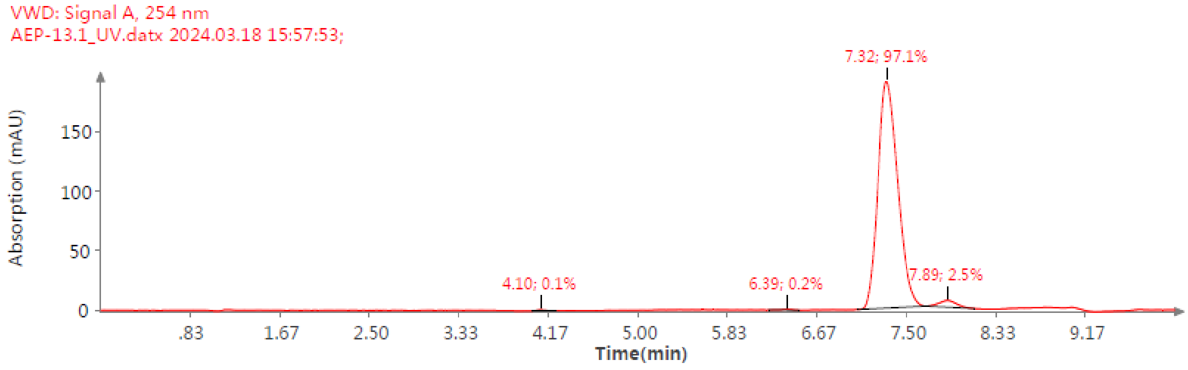

Supplementary Fig. 3. HPLC chromatogram of **7a**.

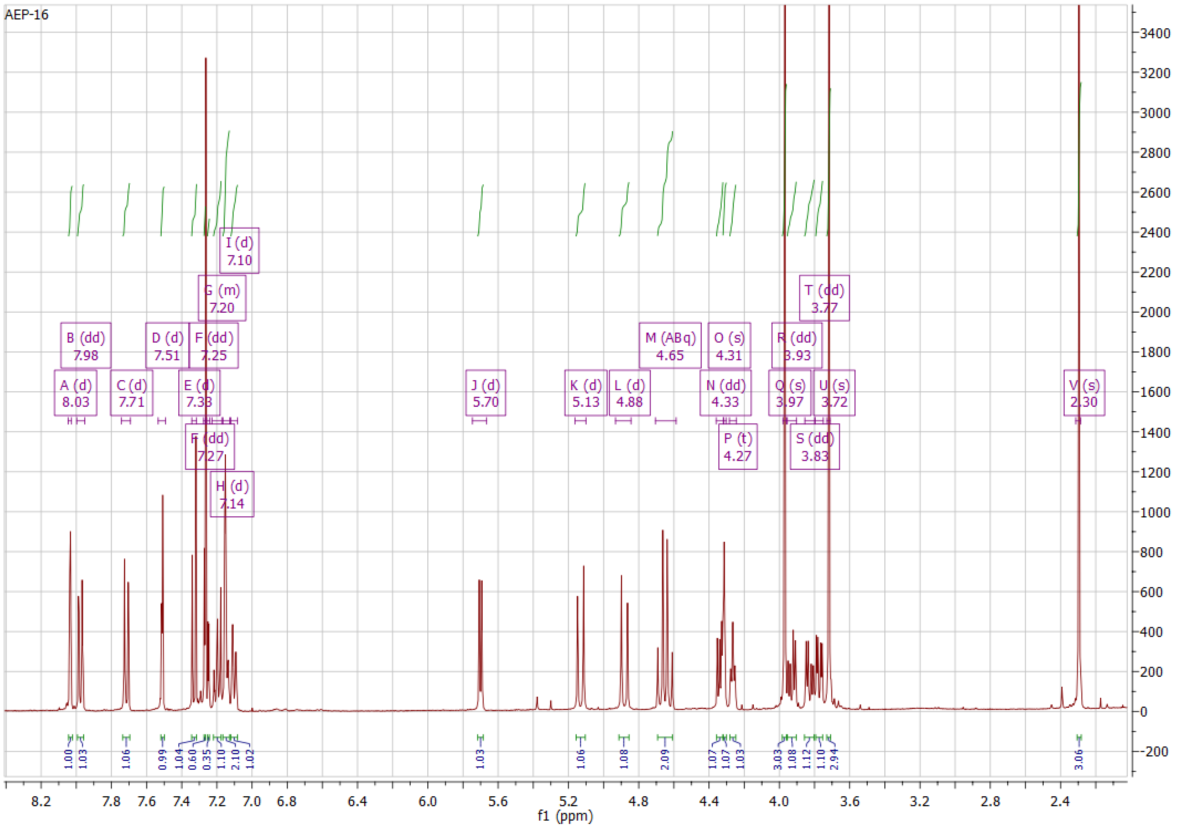

Supplementary Fig. 4. ^{1}H NMR (400 MHz, $CDCl_3$) of **7b**.

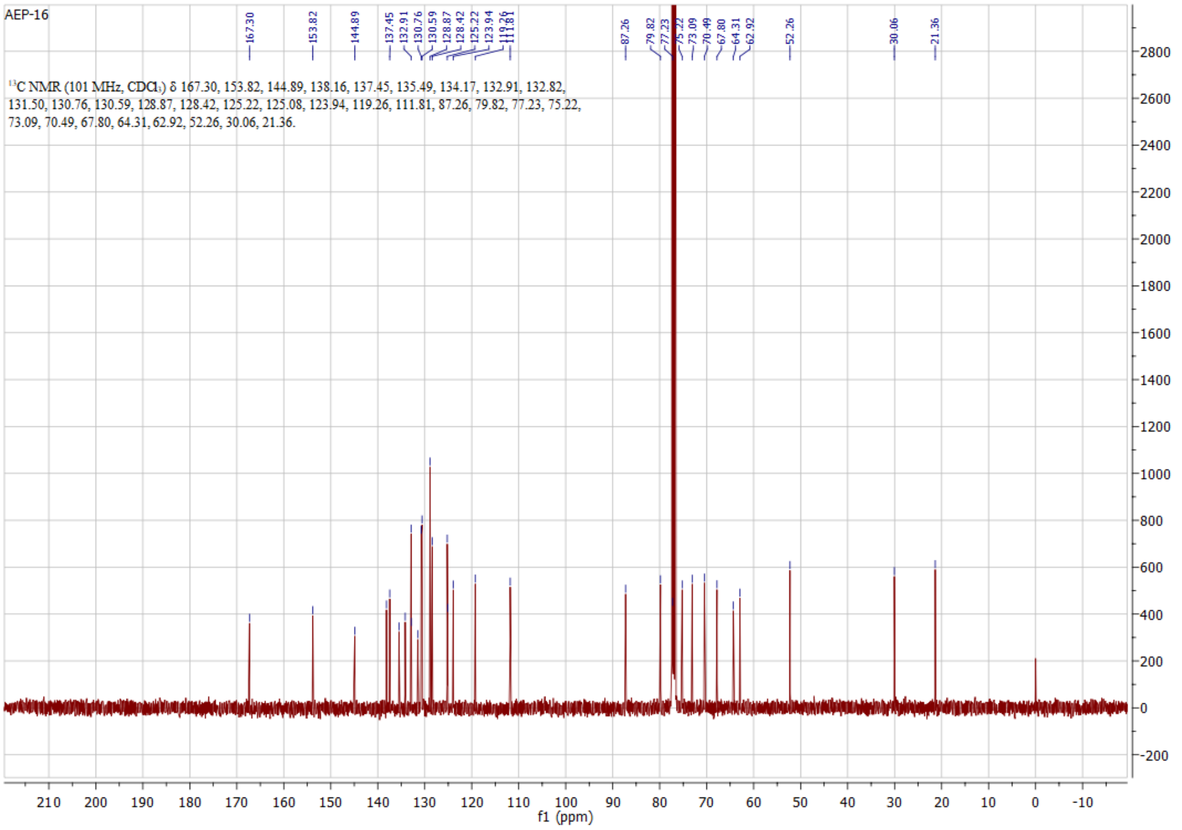

Supplementary Fig. 5. ^{13}C NMR (101 MHz, $CDCl_3$) of **7b**.

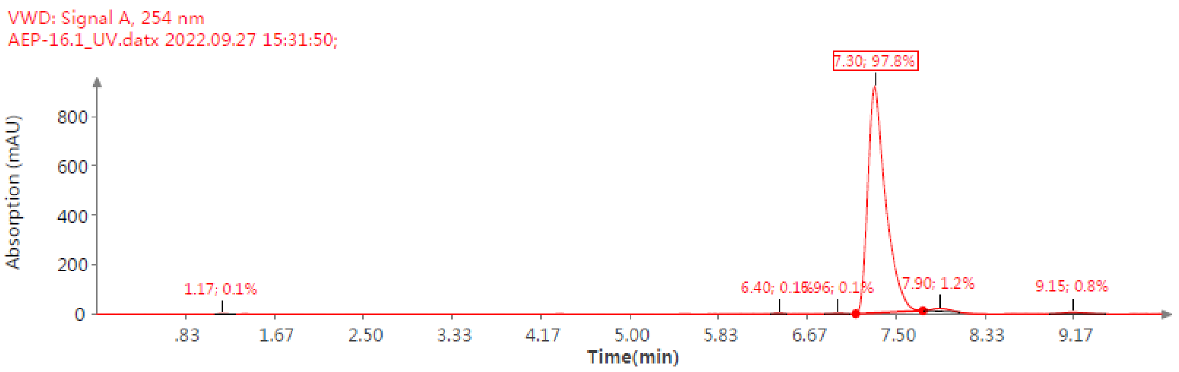

Supplementary Fig. 6. *HPLC chromatogram of* ***7b***.

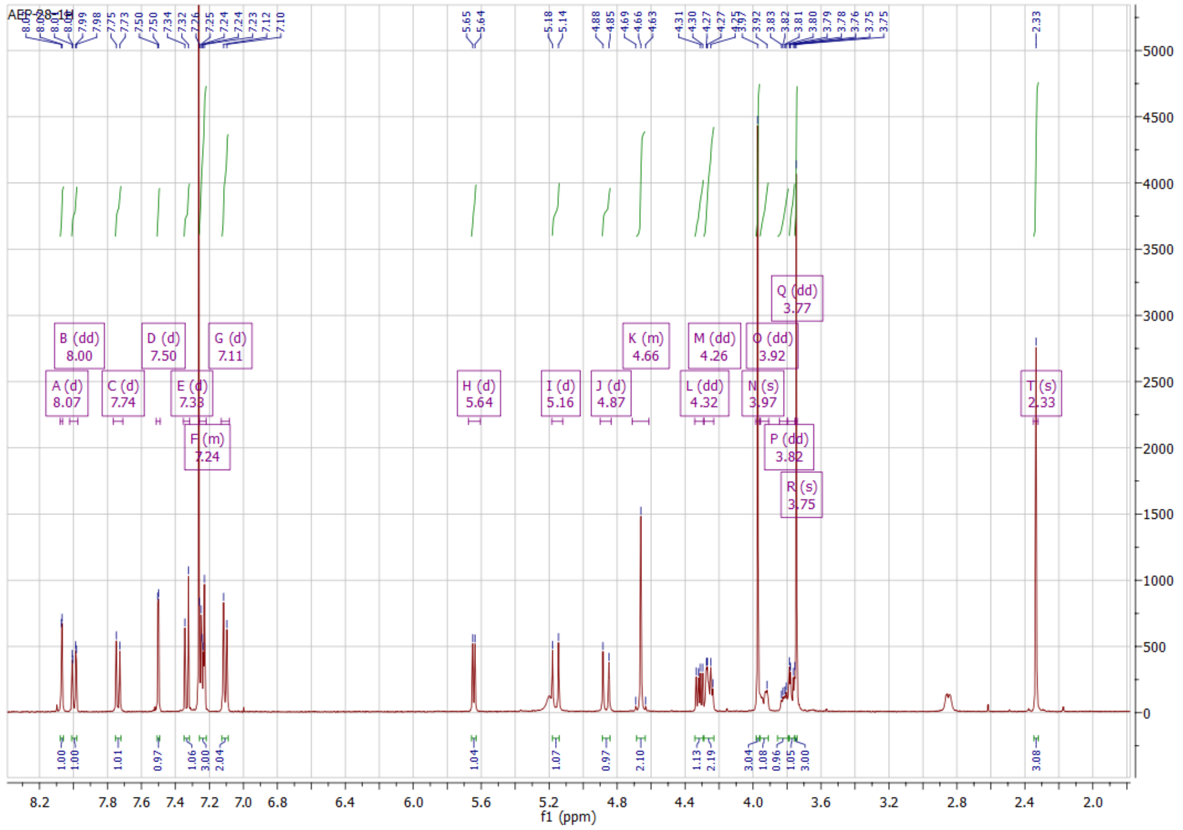

Supplementary Fig. 7. *^{1}H NMR (400 MHz, $CDCl_3$) of* ***7c***.

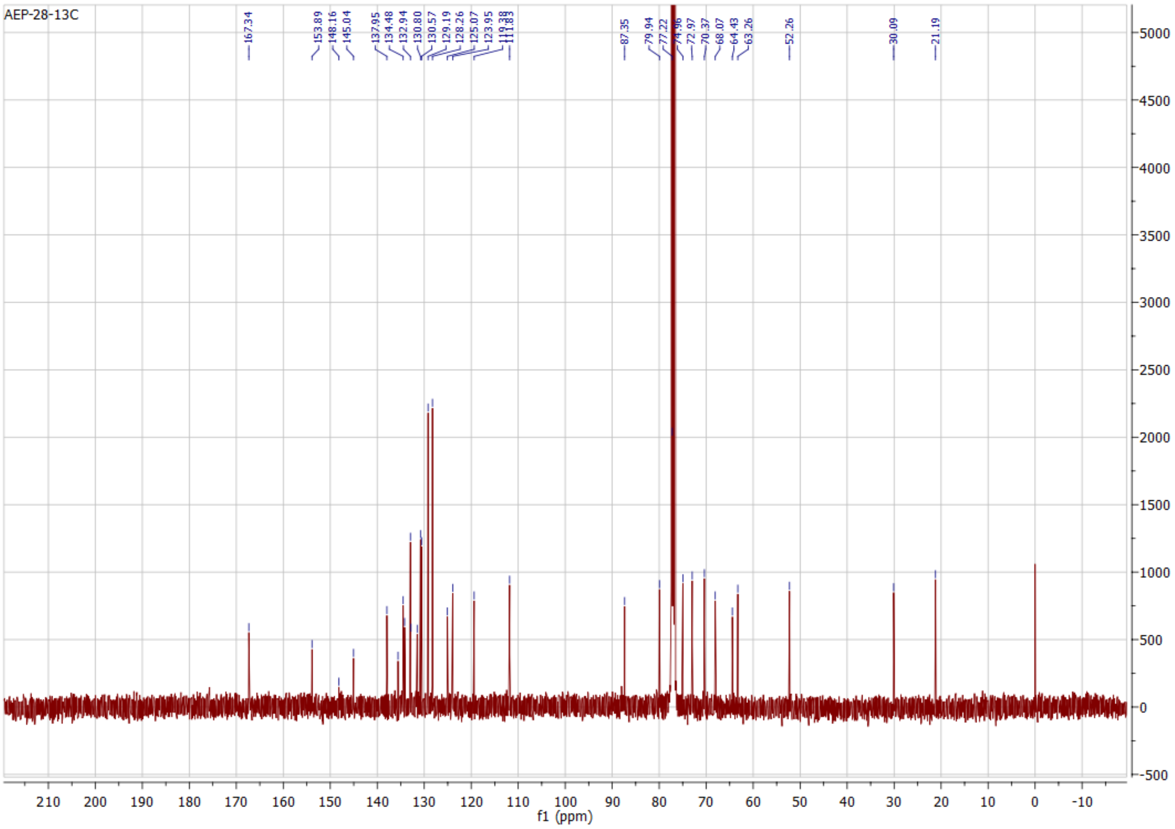

Supplementary Fig. 8. *^{13}C NMR (101 MHz, $CDCl_3$) of **7c**.*

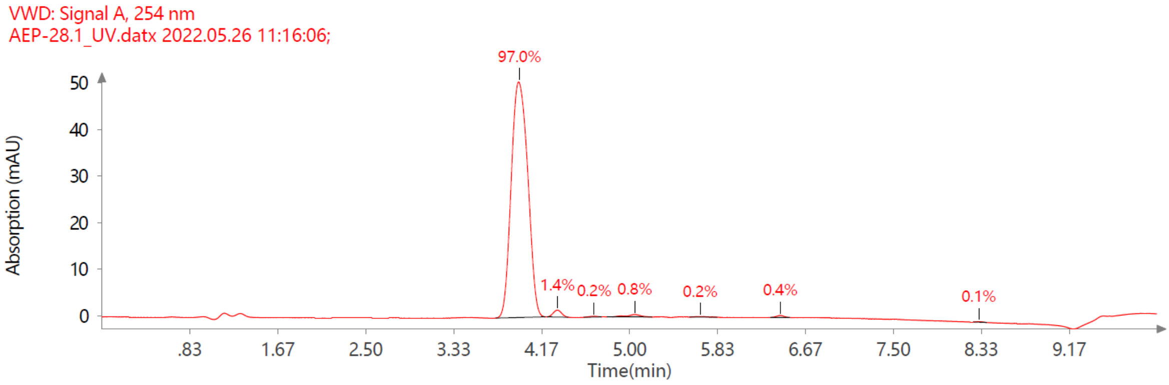

Supplementary Fig. 9. *HPLC chromatogram of **7c**.*

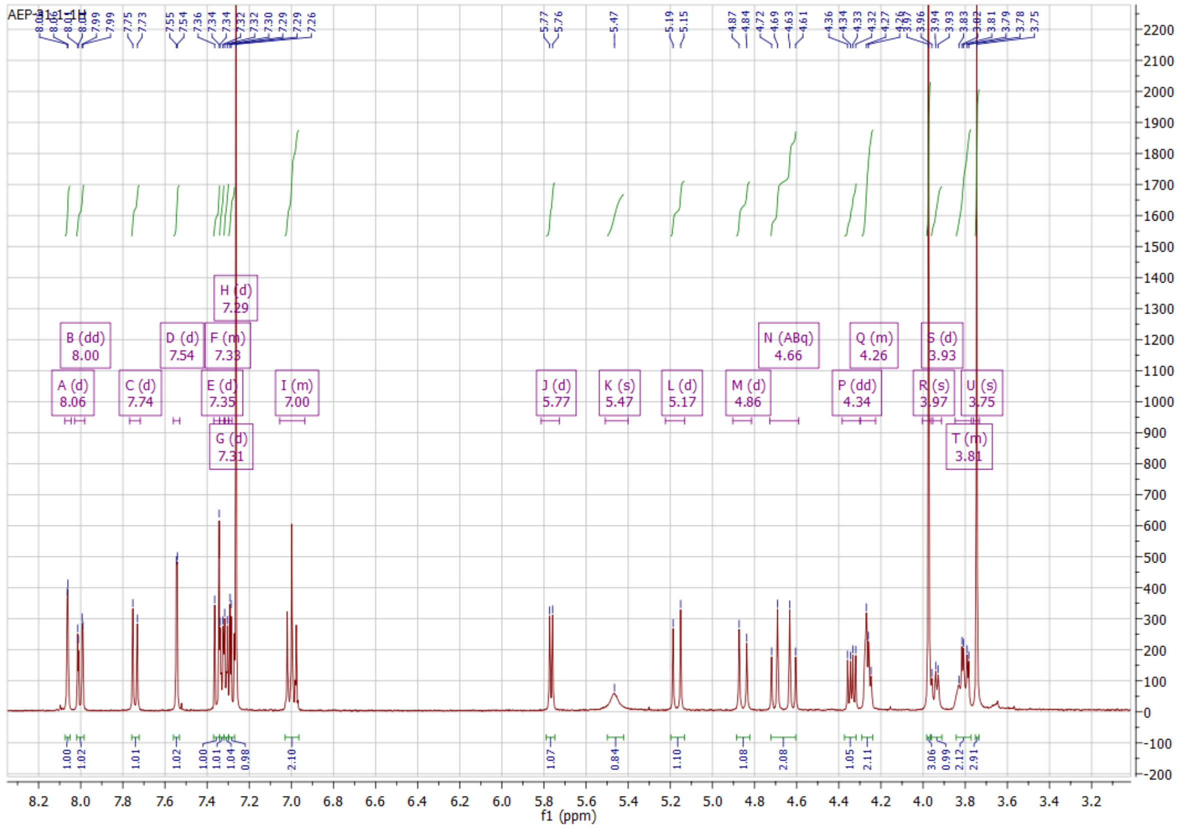

Supplementary Fig. 10. ^{1}H NMR (400 MHz, $CDCl_3$) of ***7d***.

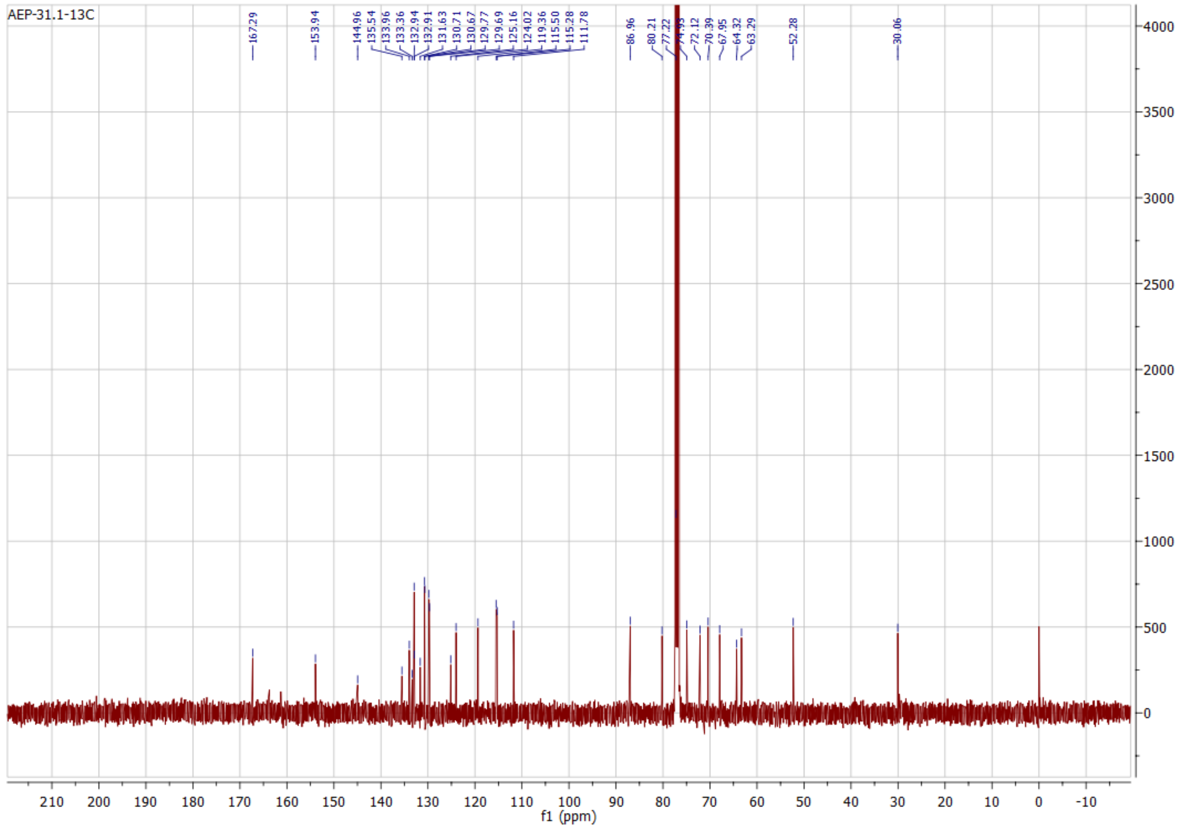

Supplementary Fig. 11. ^{13}C NMR (101 MHz, $CDCl_3$) of ***7d***.

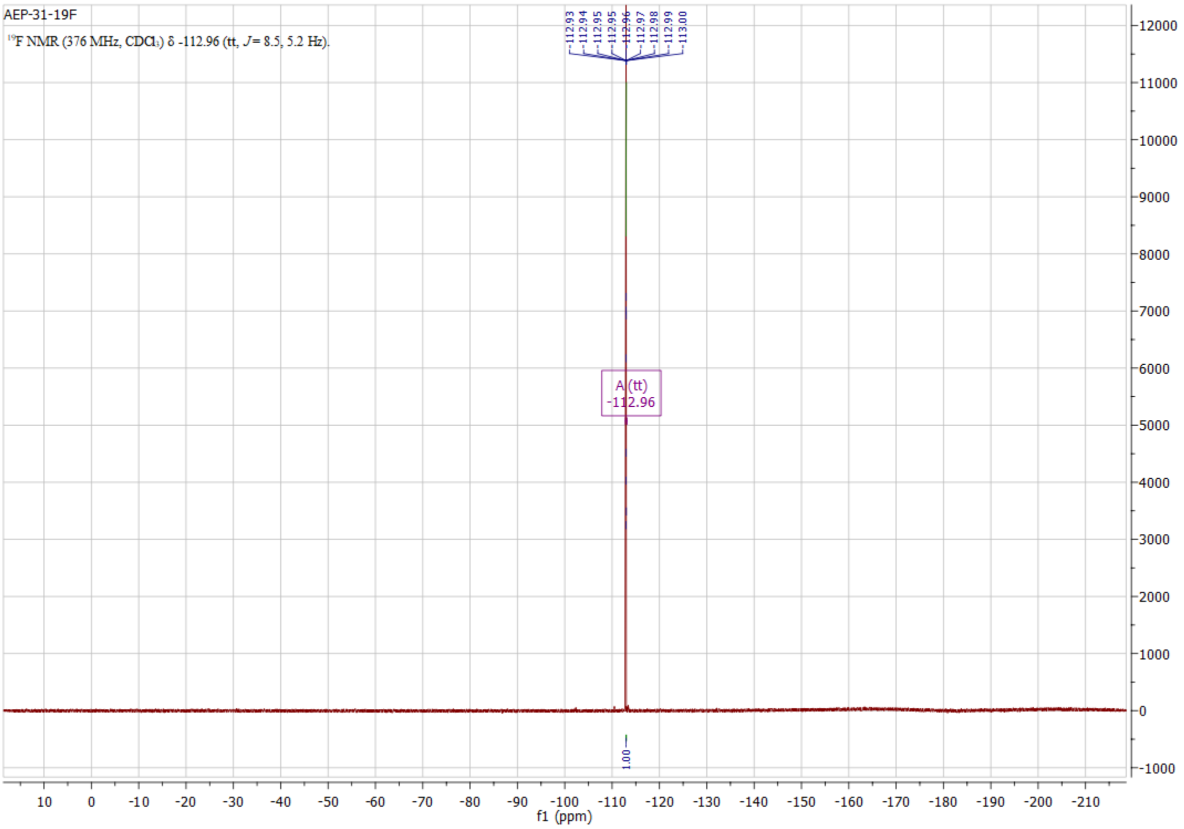

Supplementary Fig. 12. ^{19}F (376 MHz, $CDCl_3$) of ***7d***.

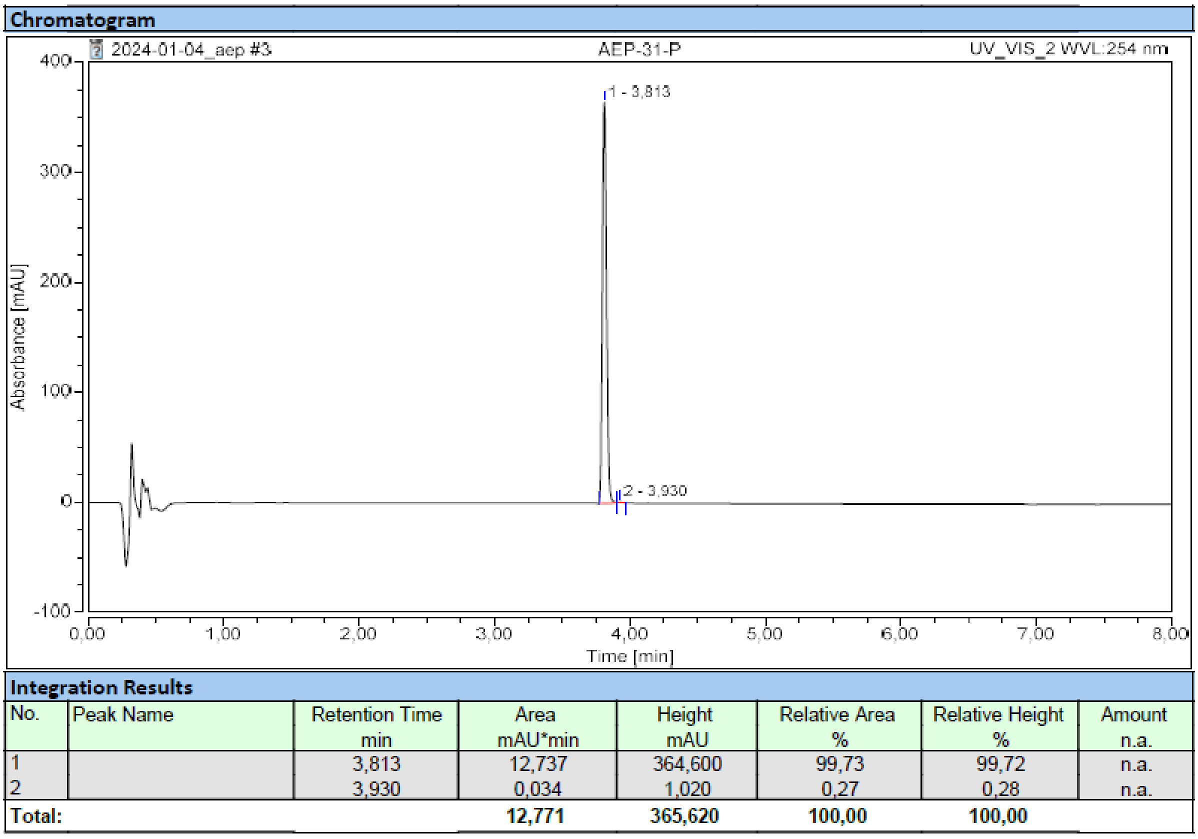

Integration Results

No.	Peak Name	Retention Time min	Area mAU*min	Height mAU	Relative Area %	Relative Height %	Amount n.a.
1		3,813	12,737	364,600	99,73	99,72	n.a.
2		3,930	0,034	1,020	0,27	0,28	n.a.
**Total:**			**12,771**	**365,620**	**100,00**	**100,00**	

Supplementary Fig. 13. HPLC chromatogram of ***7d***.

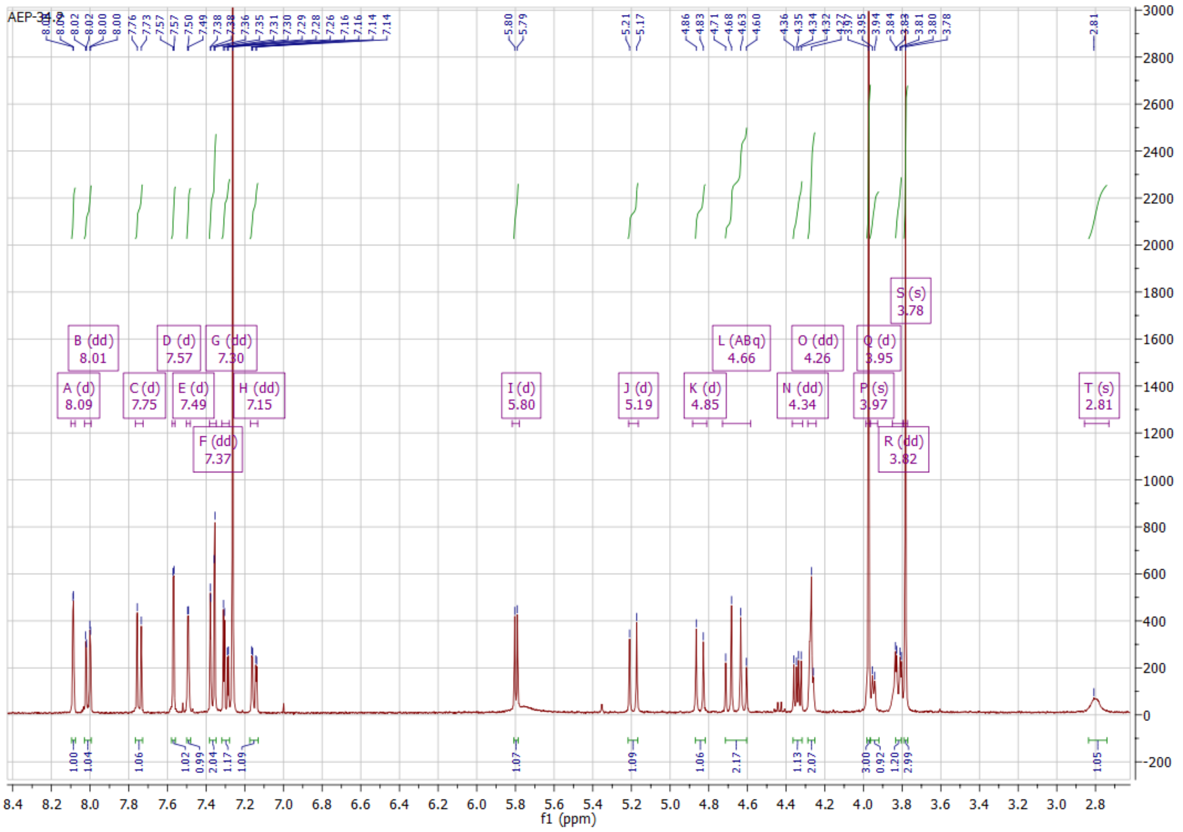

Supplementary Fig. 14. ^{1}H NMR (400 MHz, $CDCl_3$) of **7e**.

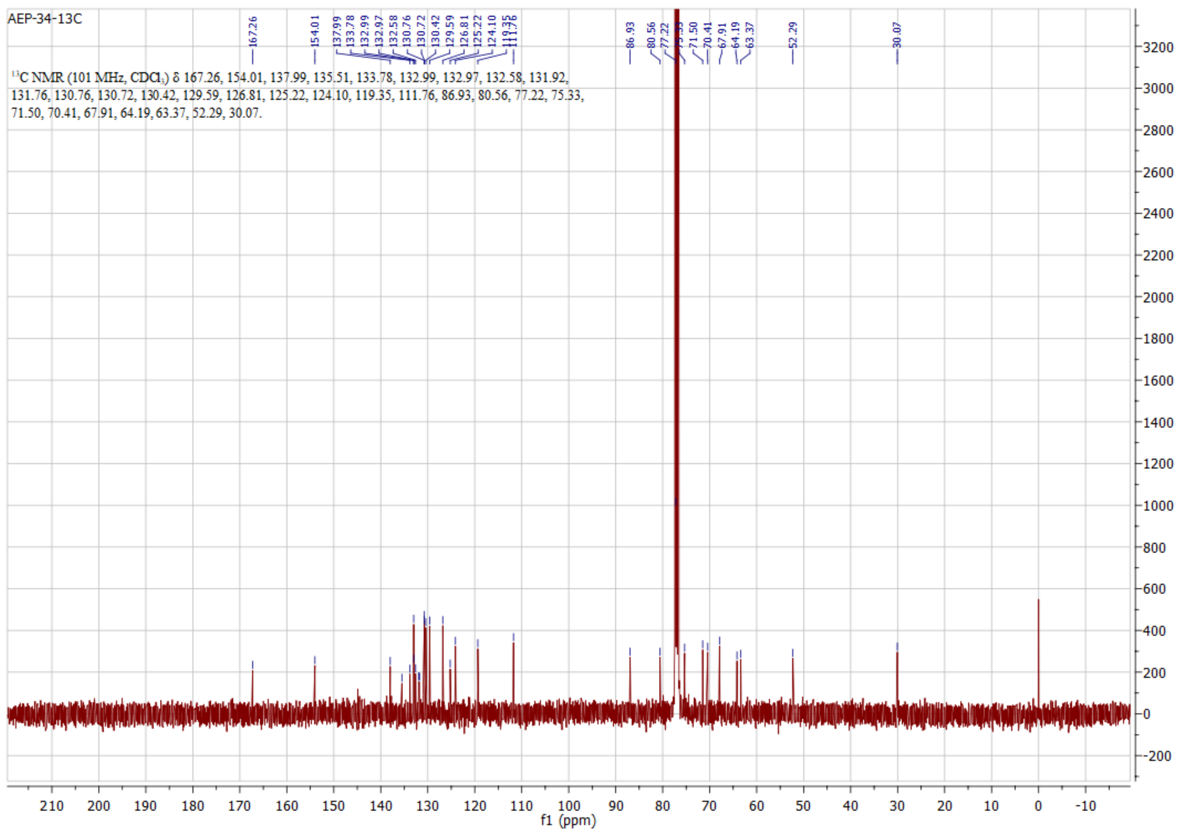

Supplementary Fig. 15. ^{13}C NMR (101 MHz, $CDCl_3$) of **7e**.

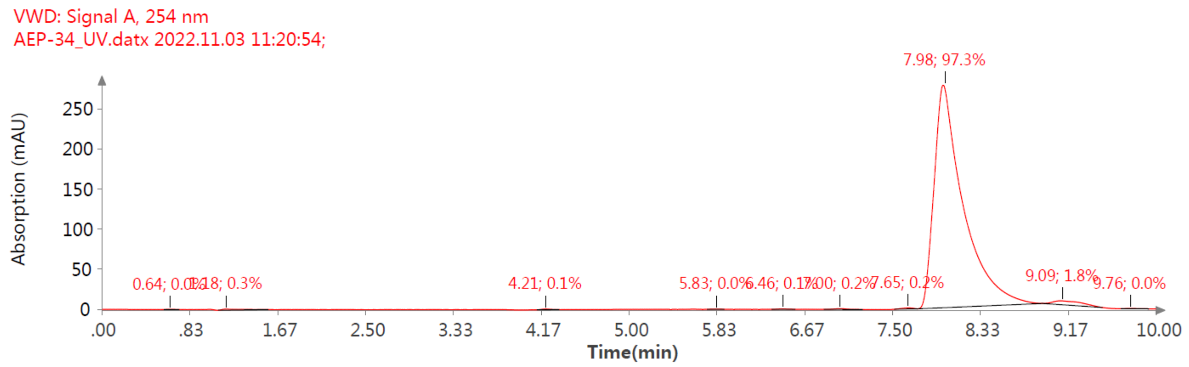

Supplementary Fig. 16. *HPLC chromatogram of **7e**.*

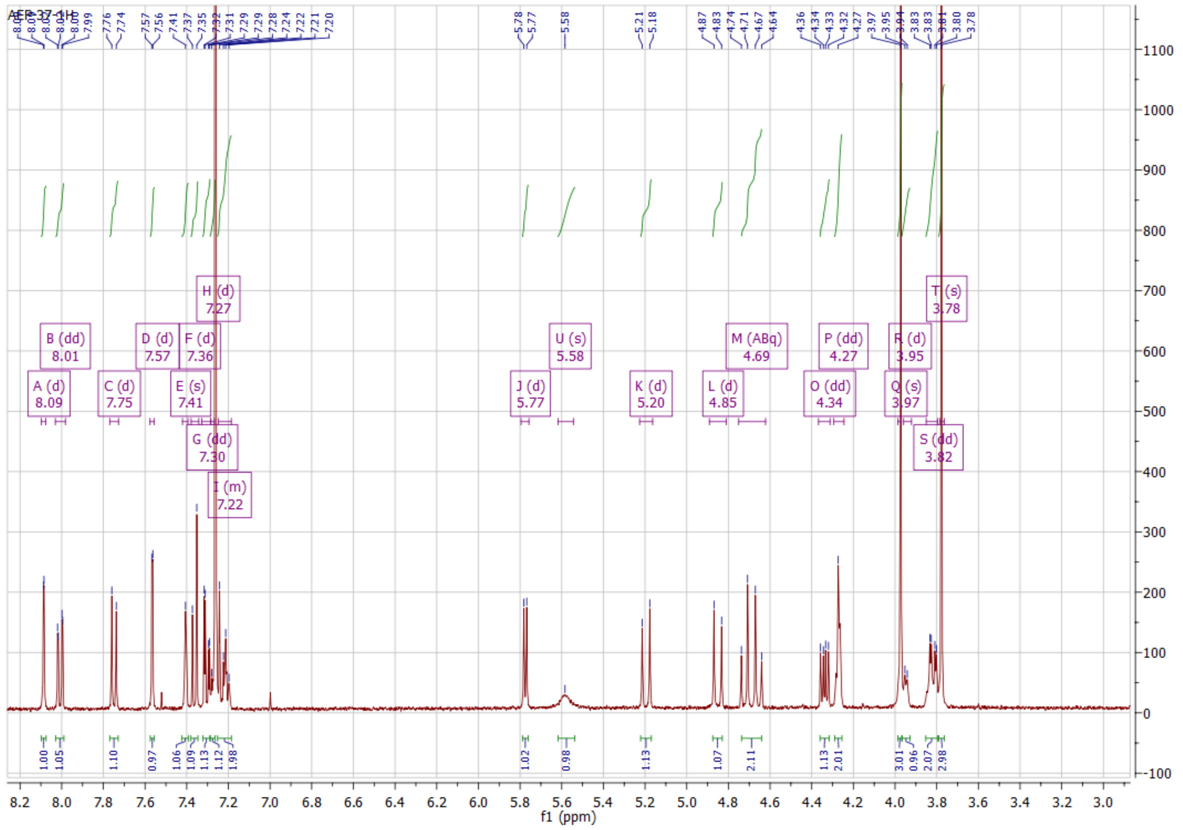

Supplementary Fig. 17. *^{1}H NMR (400 MHz, $CDCl_3$) of **7f**.*

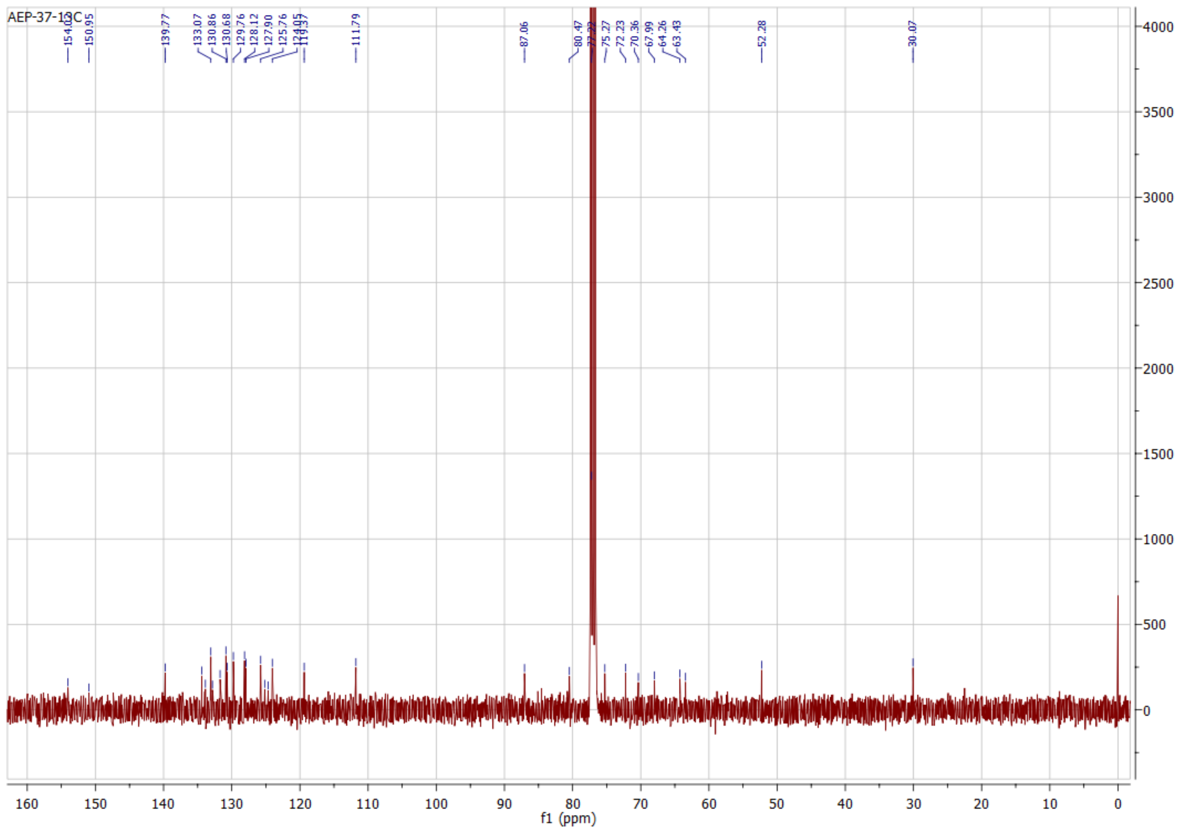

Supplementary Fig. 18*.* *^{13}C NMR (101 Mhz, $CDCl_3$) of* ***7f****.*

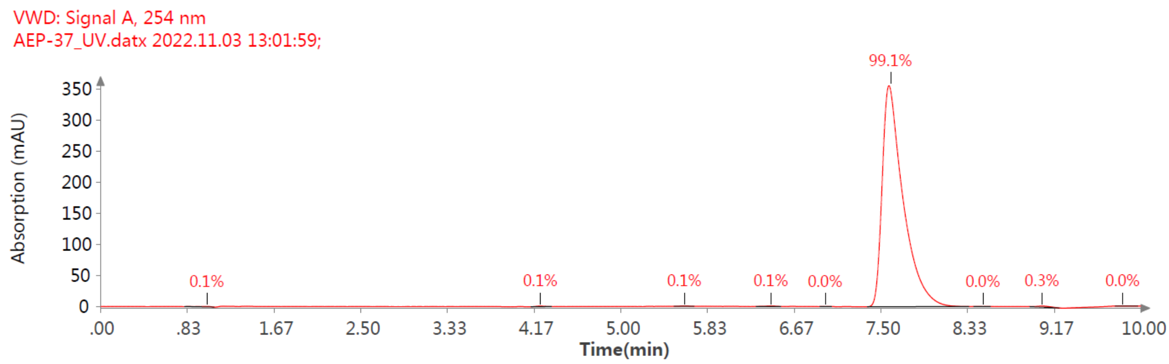

Supplementary Fig. 19*.* *HPLC chromatogram of* ***7f****.*

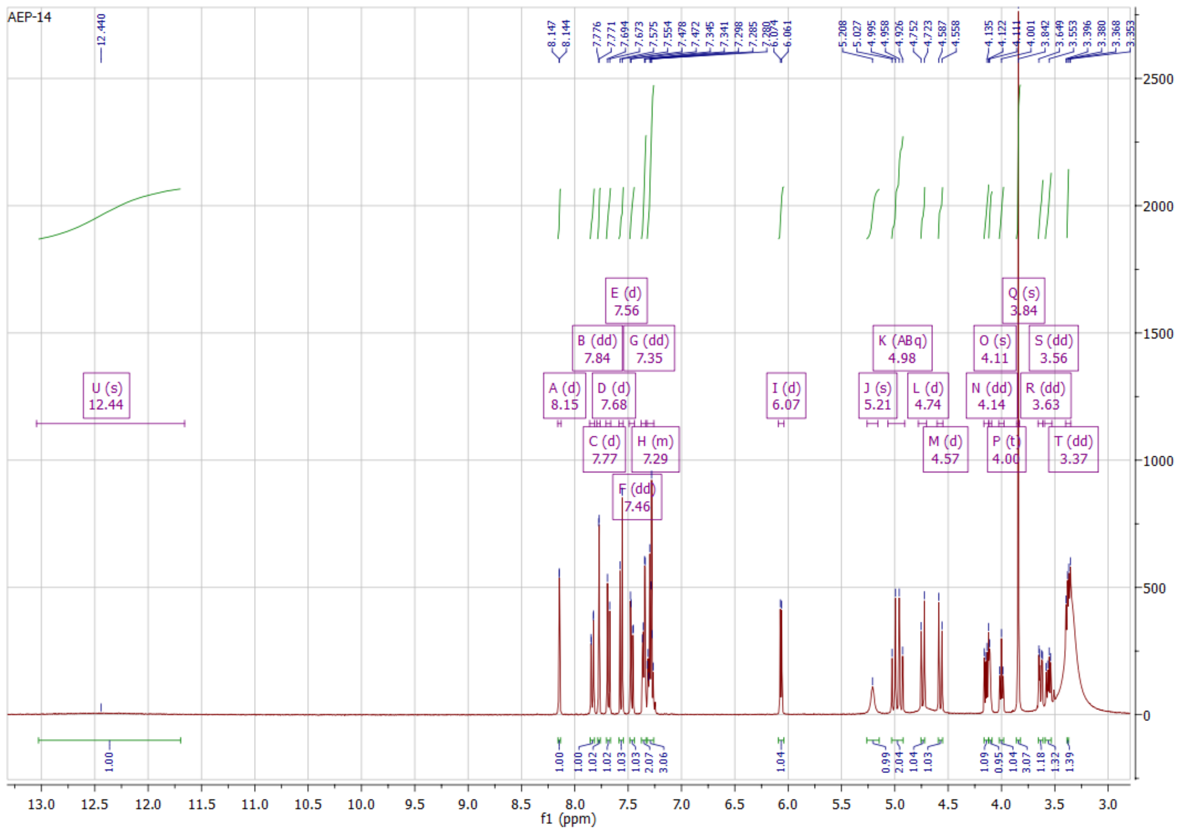

***Supplementary Fig. 20**. ^{1}H NMR (400 MHz, DMSO-d_6) of **8a**.*

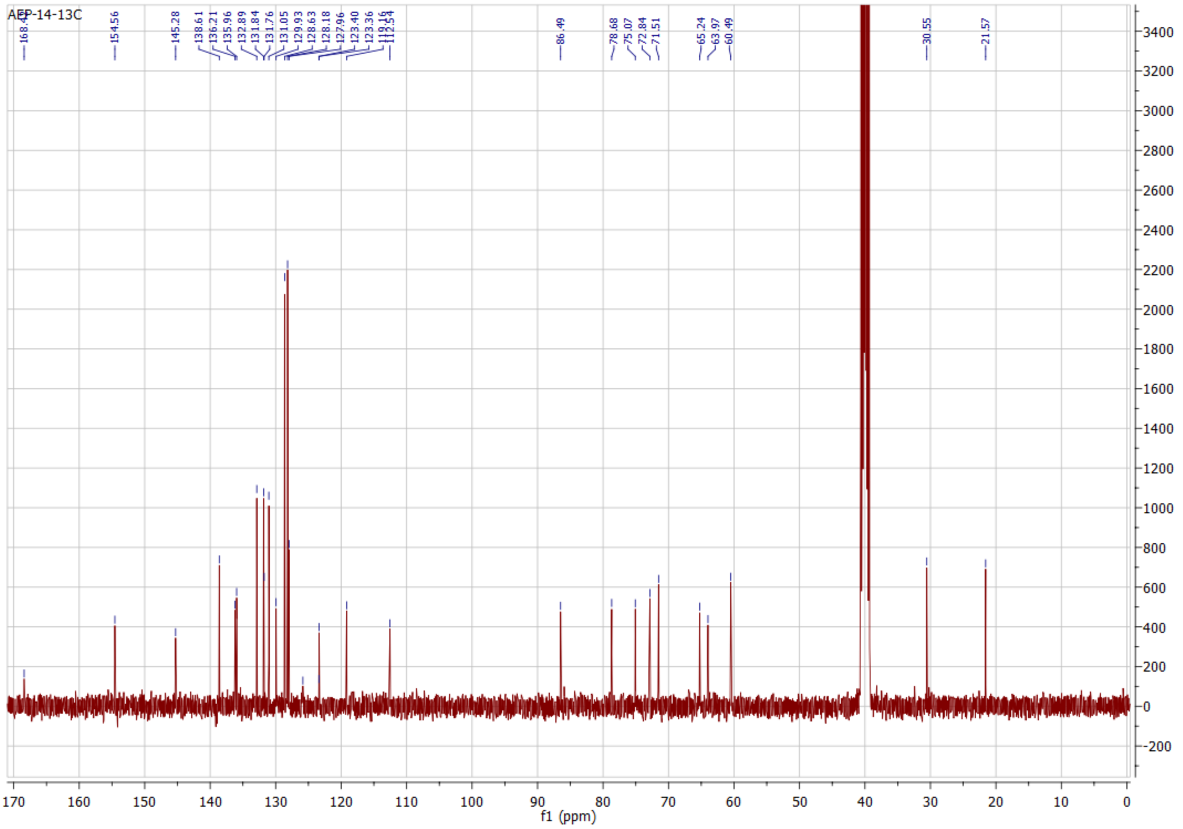

***Supplementary Fig. 21**. ^{13}C NMR (101 MHz, DMSO-d_6) of **8a**.*

Chromatogram

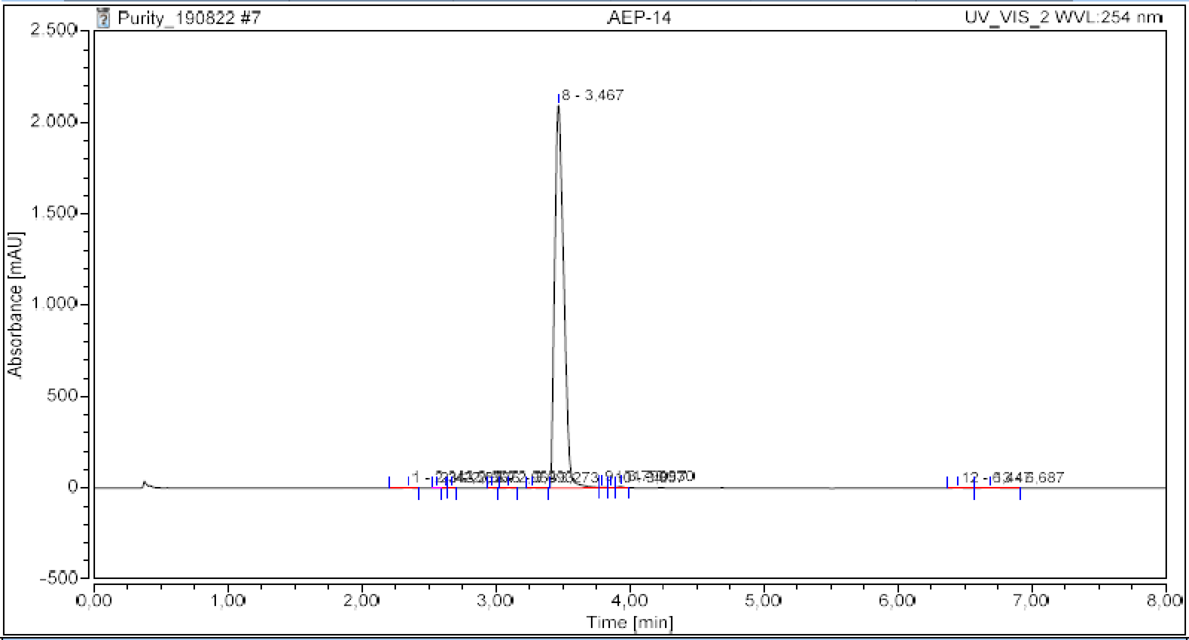

Integration Results

No.	Peak Name	Retention Time min	Area mAU*min	Height mAU	Relative Area %	Relative Height %	Amount n.a.
1		2,343	0,121	1,439	0,07	0,07	n.a.
2		2,557	0,006	0,177	0,00	0,01	n.a.
3		2,623	0,026	0,622	0,02	0,03	n.a.
4		2,667	0,066	1,730	0,04	0,08	n.a.
5		2,963	0,055	1,508	0,03	0,07	n.a.
6		3,093	0,080	1,561	0,05	0,07	n.a.
7		3,273	0,062	0,733	0,04	0,03	n.a.
8		3,467	163,020	2093,371	99,29	98,96	n.a.
9		3,790	0,190	3,278	0,12	0,15	n.a.
10		3,857	0,075	1,700	0,05	0,08	n.a.
11		3,930	0,349	7,910	0,21	0,37	n.a.
12		6,447	0,052	0,775	0,03	0,04	n.a.
13		6,687	0,079	0,529	0,05	0,03	n.a.
**Total:**			**164,180**	**2115,331**	**100,00**	**100,00**	

Supplementary Fig. 22. *HPLC chromatogram of **8a**.*

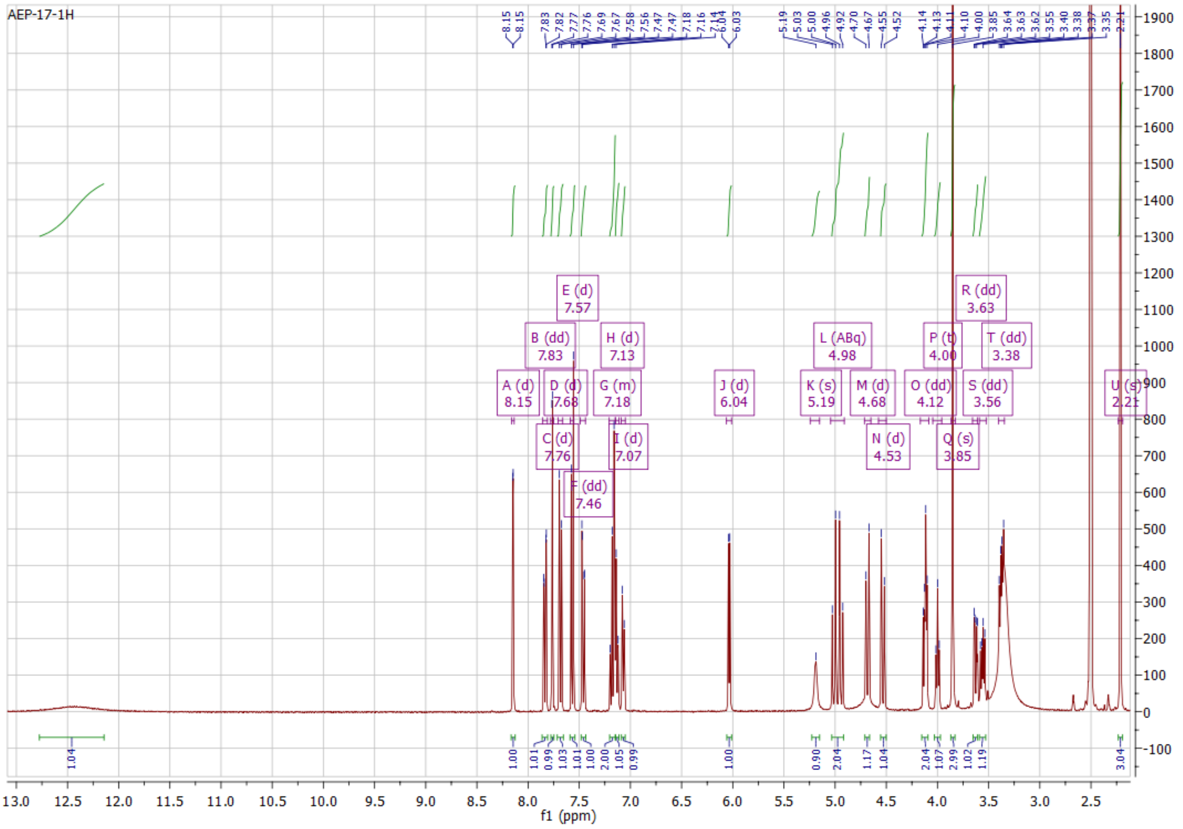

Supplementary Fig. 23. ^{1}H NMR (400 MHz, DMSO-d_6) of **8b**.

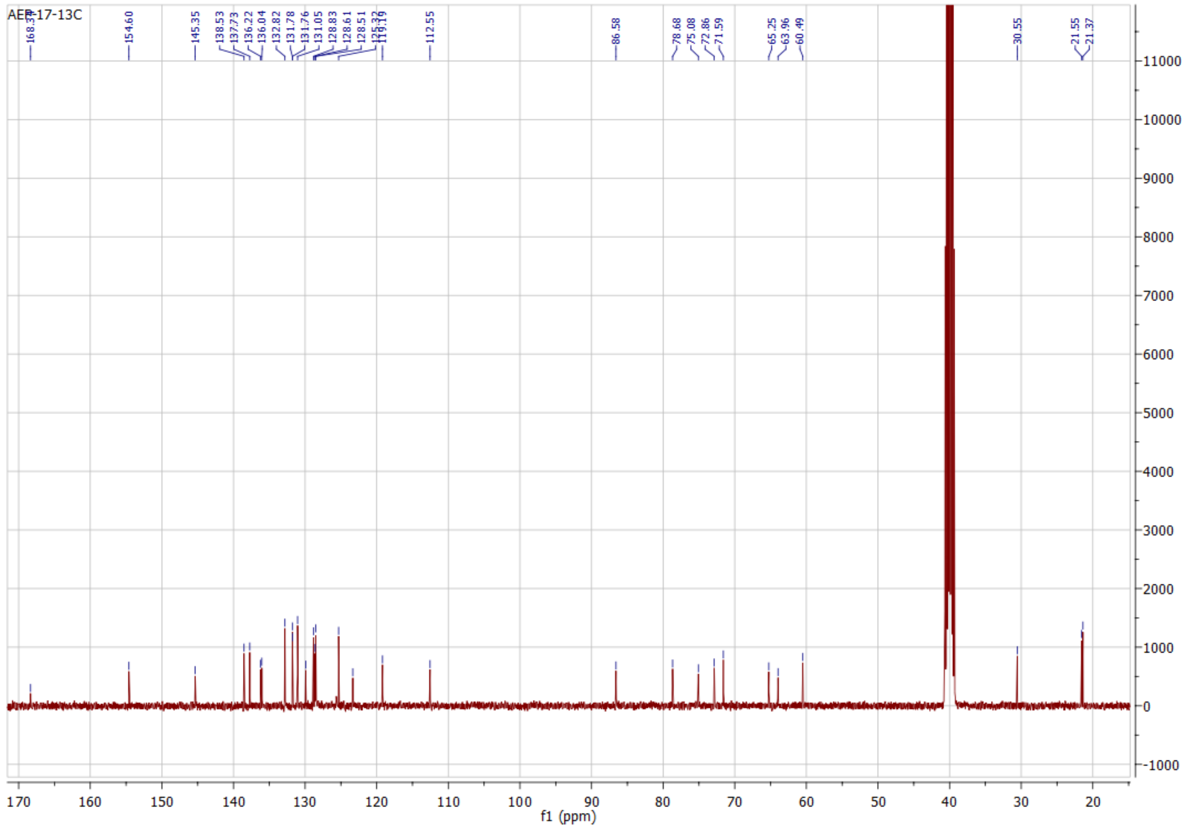

Supplementary Fig. 24. ^{13}C NMR (101 MHz, DMSO-d_6) of **8b**.

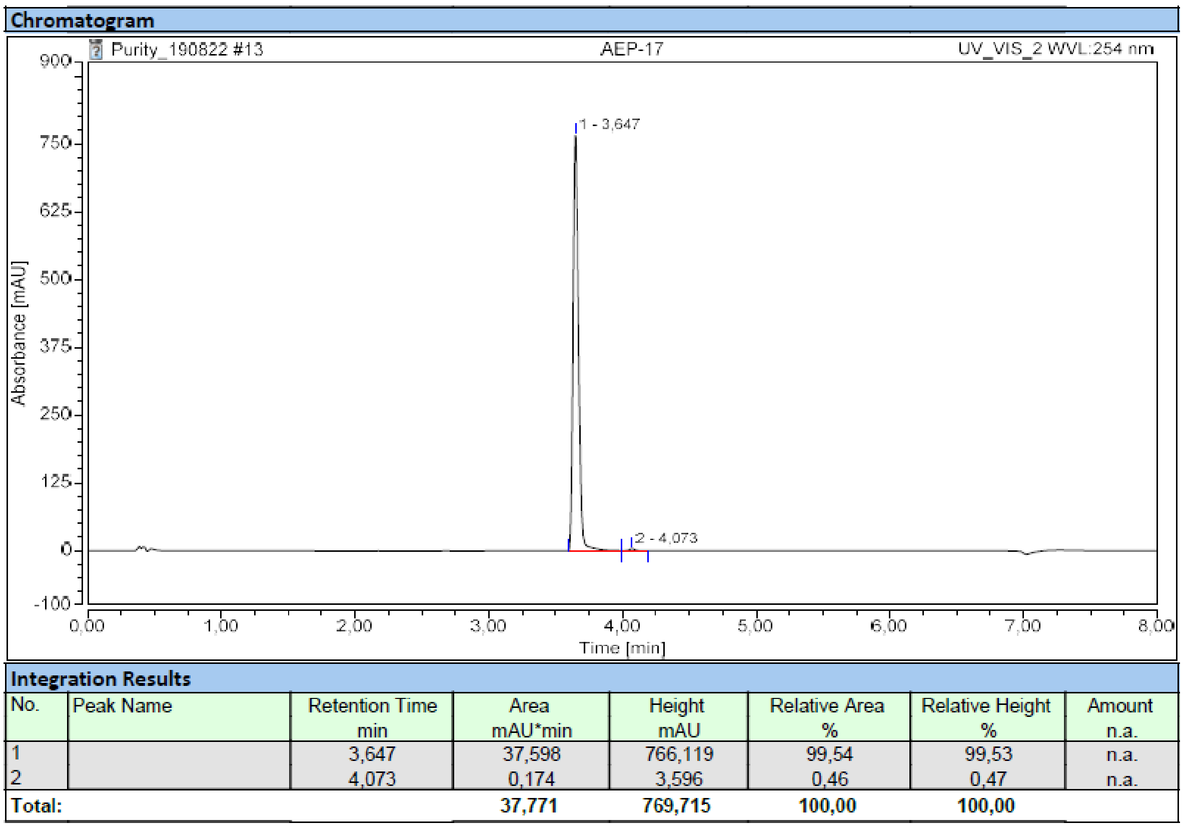

Integration Results

No.	Peak Name	Retention Time min	Area mAU*min	Height mAU	Relative Area %	Relative Height %	Amount n.a.
1		3,647	37,598	766,119	99,54	99,53	n.a.
2		4,073	0,174	3,596	0,46	0,47	n.a.
**Total:**			**37,771**	**769,715**	**100,00**	**100,00**	

Supplementary Fig. 25. *HPLC chromatogram of **8b**.*

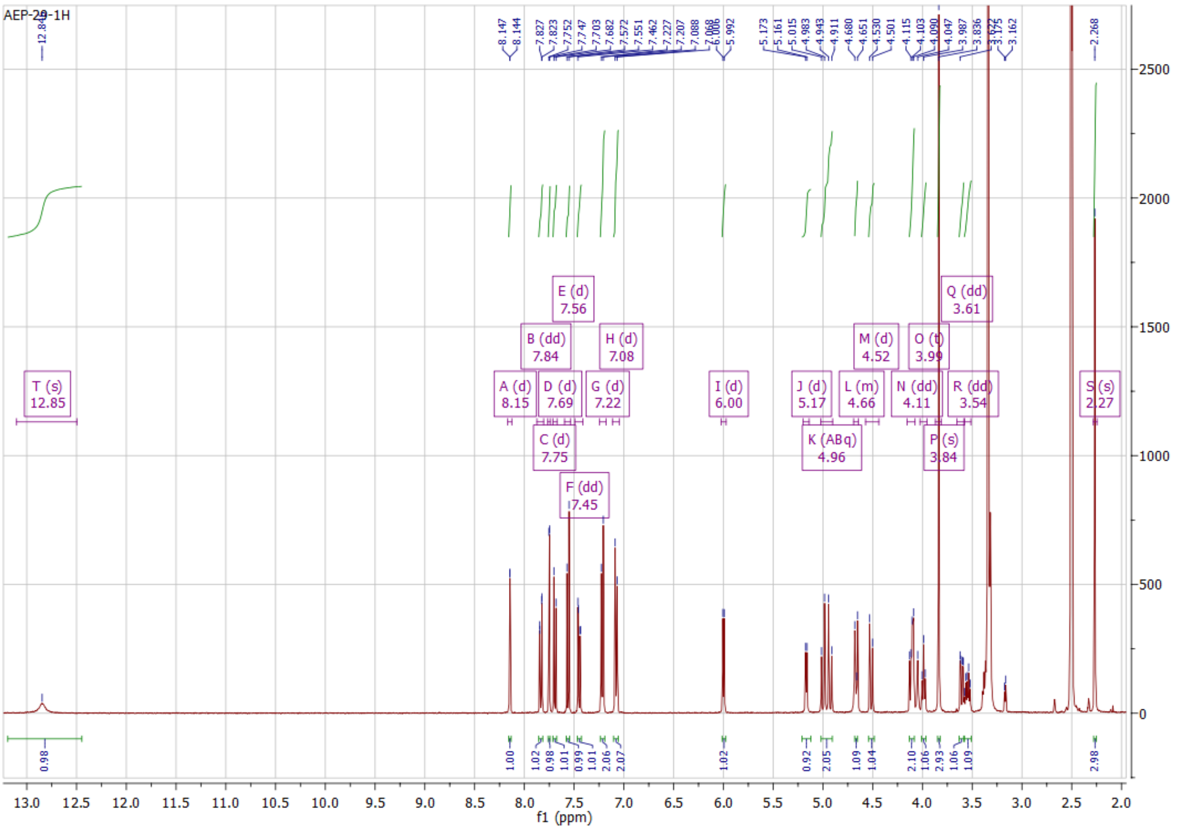

Supplementary Fig. 26. *^{1}H NMR (400 MHz, DMSO-d_6) of **8c**.*

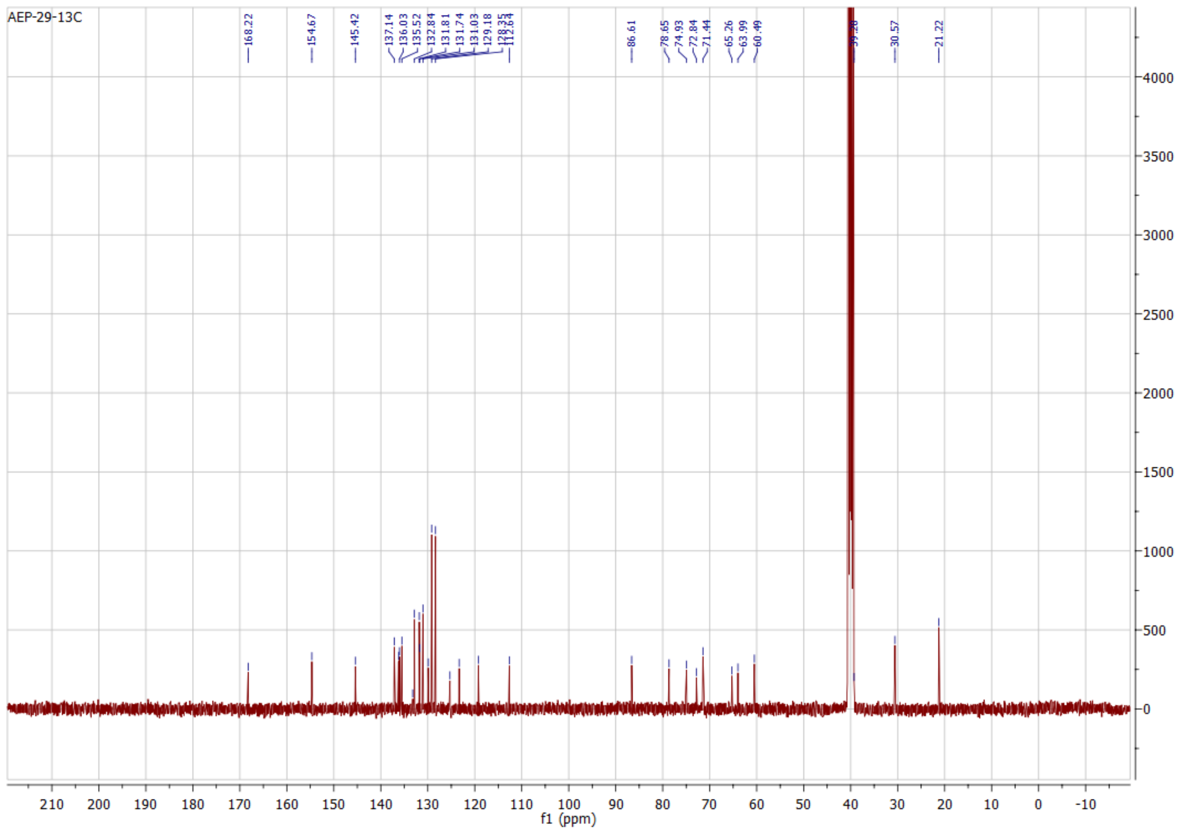

***Supplementary Fig. 27**. ^{13}C NMR (101 MHz, DMSO-d_6) of **8c**.*

Chromatogram

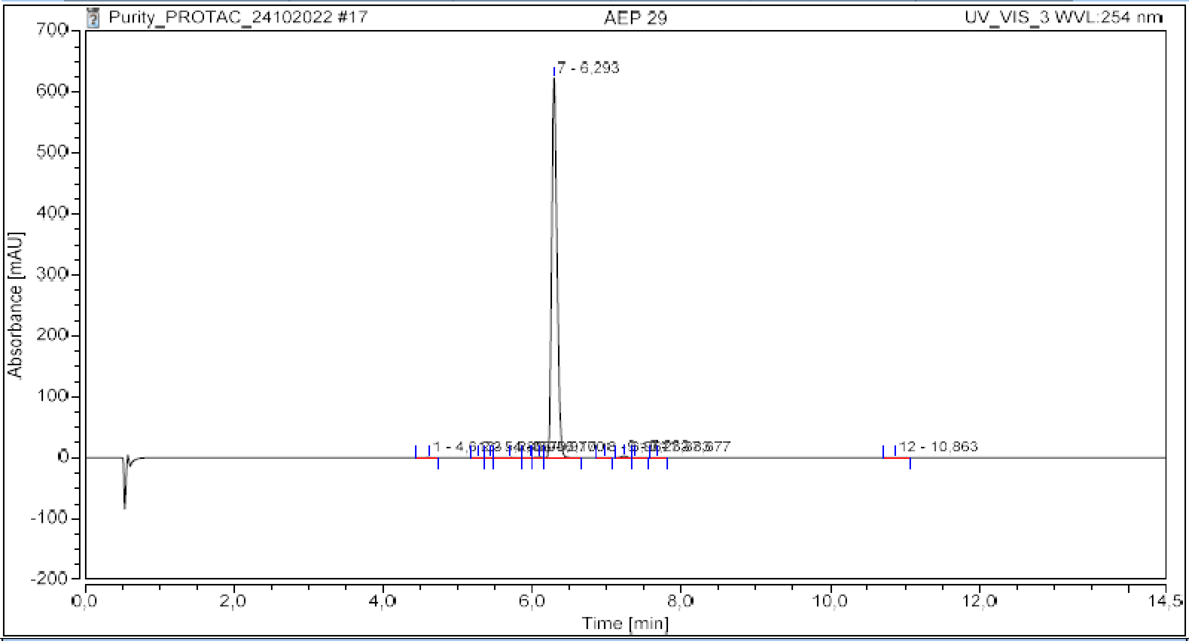

Integration Results

No.	Peak Name	Retention Time<br>min	Area<br>mAU*min	Height<br>mAU	Relative Area<br>%	Relative Height<br>%	Amount<br>n.a.
1		4,613	0,049	0,718	0,09	0,11	n.a.
2		5,287	0,006	0,062	0,01	0,01	n.a.
3		5,430	0,010	0,188	0,02	0,03	n.a.
4		5,707	0,047	0,356	0,09	0,06	n.a.
5		5,977	0,013	0,155	0,02	0,02	n.a.
6		6,100	0,085	1,027	0,17	0,16	n.a.
7		6,293	50,946	623,044	99,05	99,09	n.a.
8		6,967	0,013	0,156	0,02	0,02	n.a.
9		7,233	0,220	2,634	0,43	0,42	n.a.
10		7,383	0,020	0,175	0,04	0,03	n.a.
11		7,677	0,012	0,124	0,02	0,02	n.a.
12		10,863	0,016	0,103	0,03	0,02	n.a.
**Total:**			**51,437**	**628,741**	**100,00**	**100,00**	

Supplementary Fig. 28*. HPLC chromatogram of* ***8c****.*

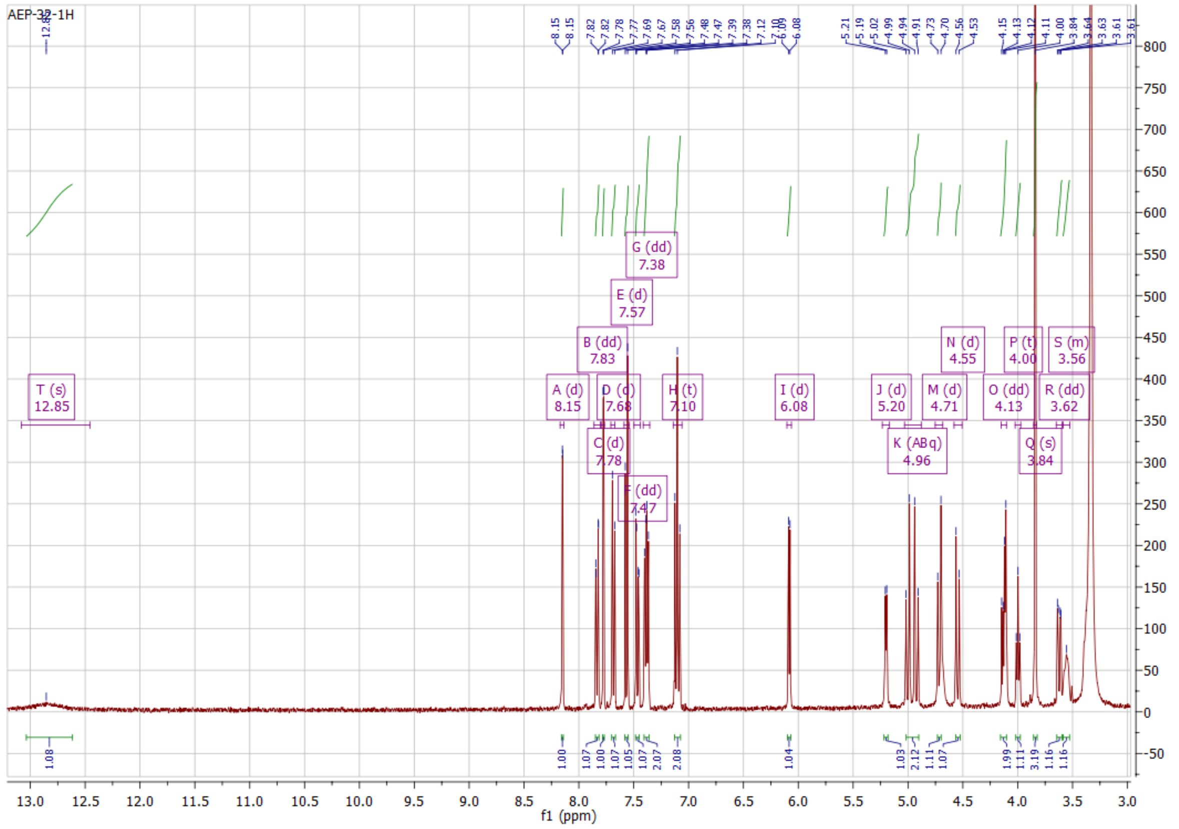

Supplementary Fig. 29. ^{1}H *NMR (400 MHz, DMSO-d$_6$) of* ***8d***.

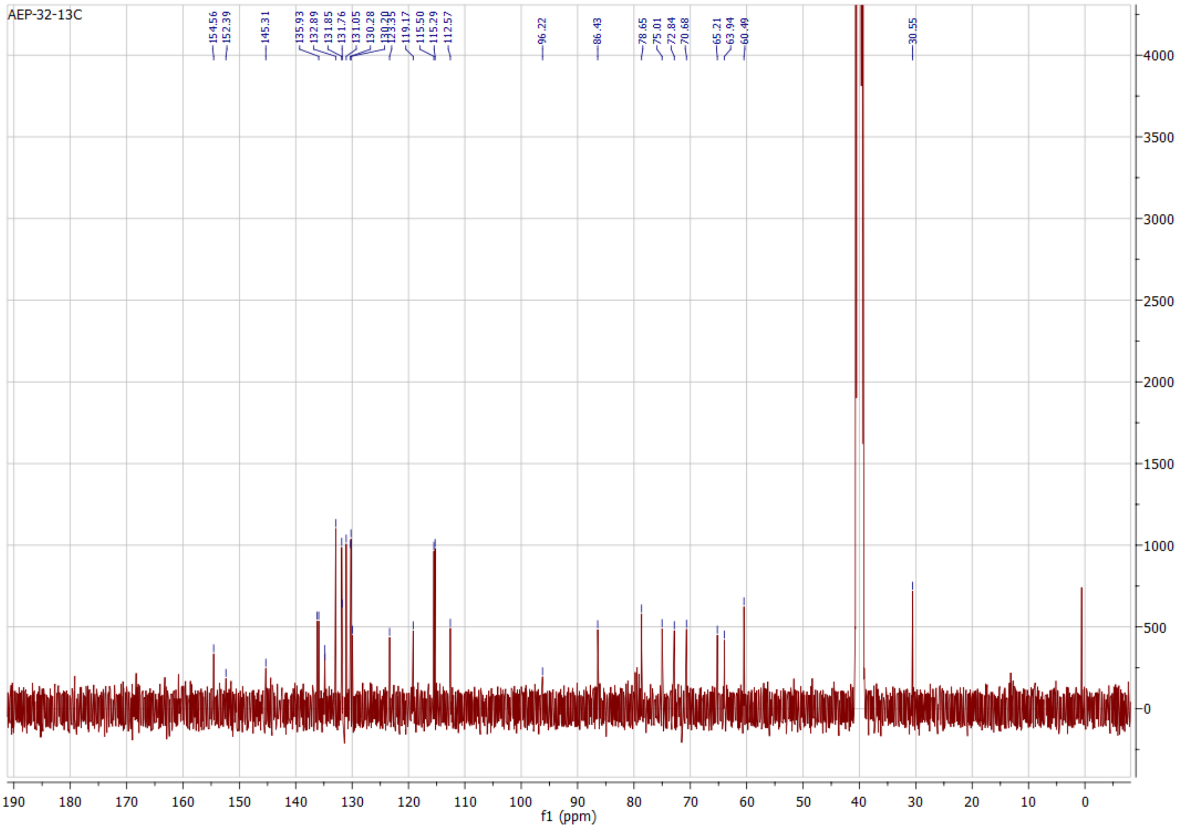

Supplementary Fig. 30. ^{13}C *NMR (101 MHz, DMSO-d$_6$) of* ***8d***.

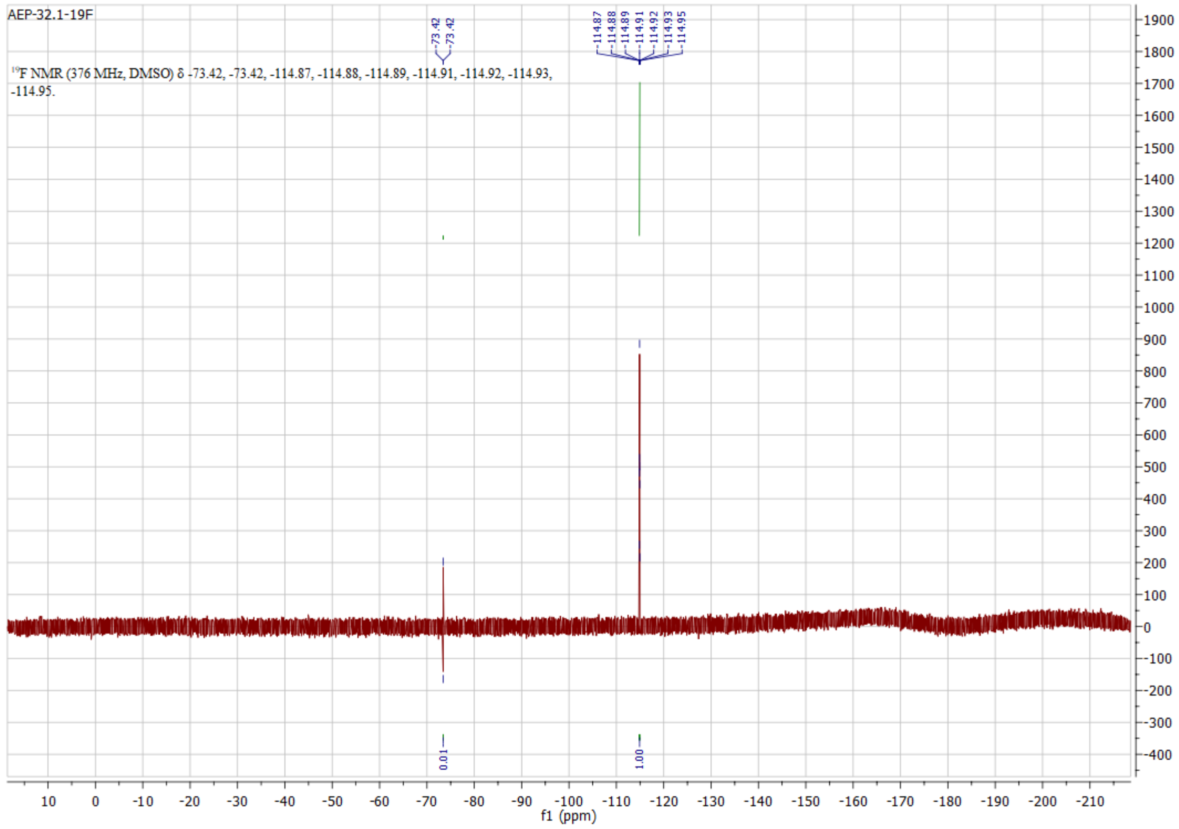

Supplementary Fig. 31. *^{19}F (376 MHz, DMSO-d_6) of **8d**.*

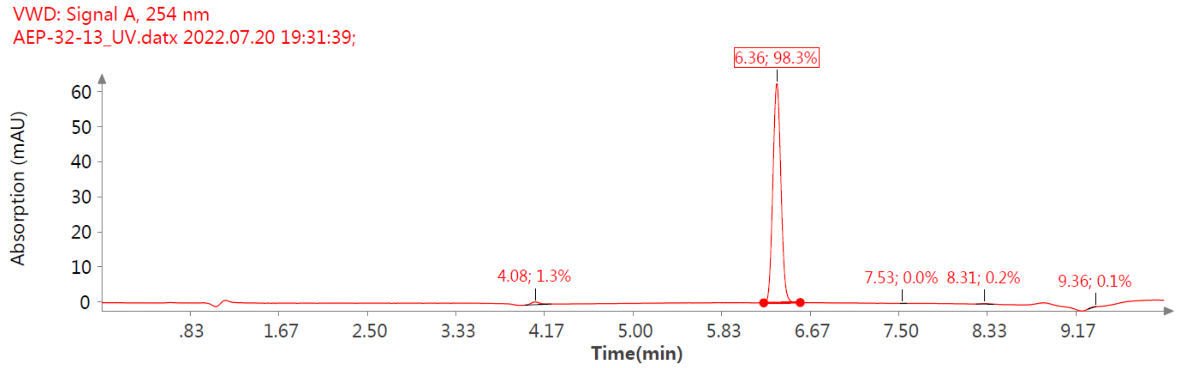

Supplementary Fig. 32. *HPLC chromatogram of **8d**.*

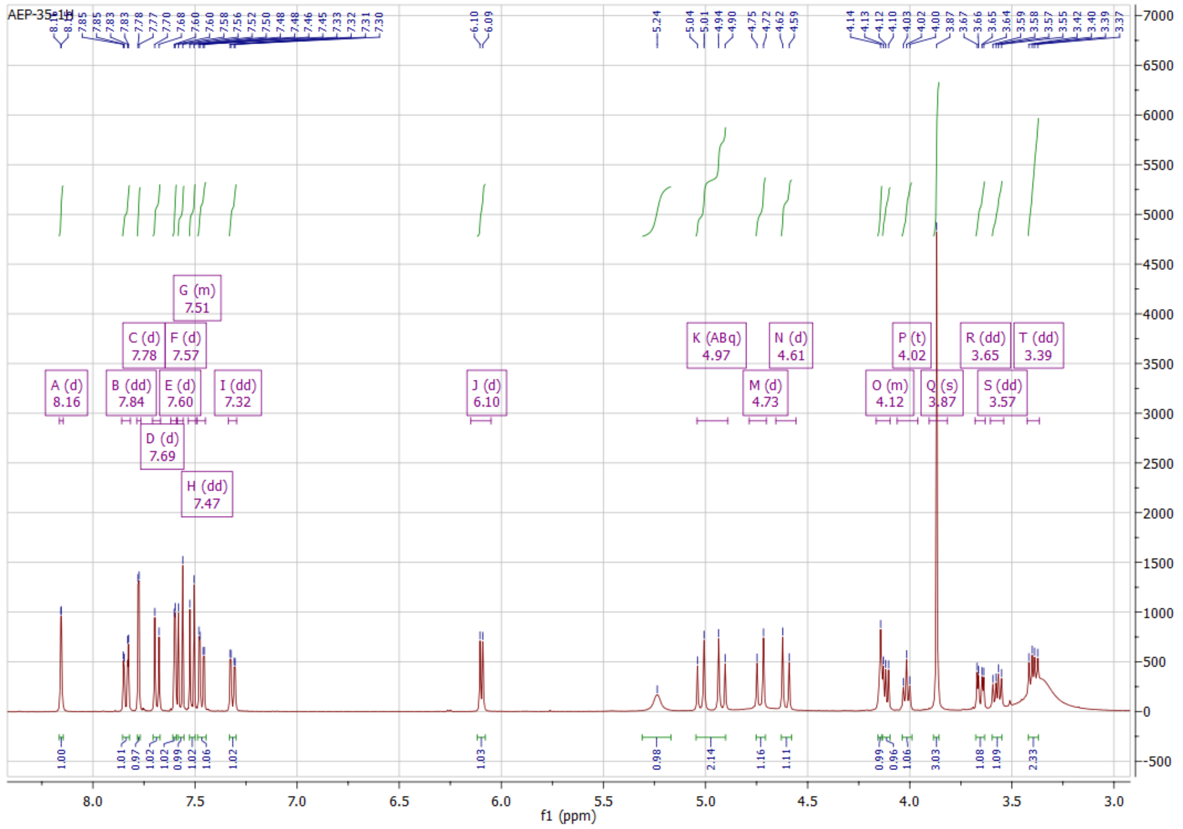

Supplementary Fig. 33. ^{1}H NMR (400 MHz, DMSO-d_6) of **8e**.

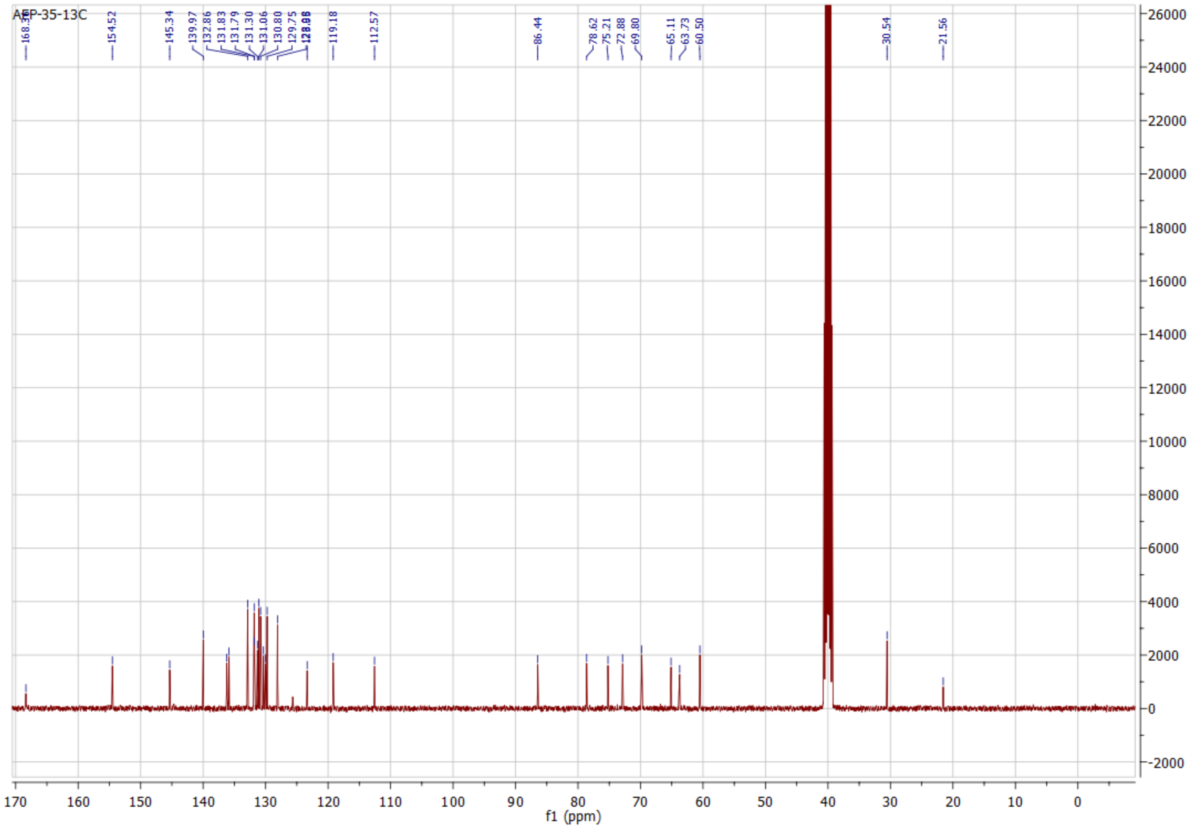

Supplementary Fig. 34. ^{13}C NMR (101 MHz, DMSO-d_6) of **8e**.

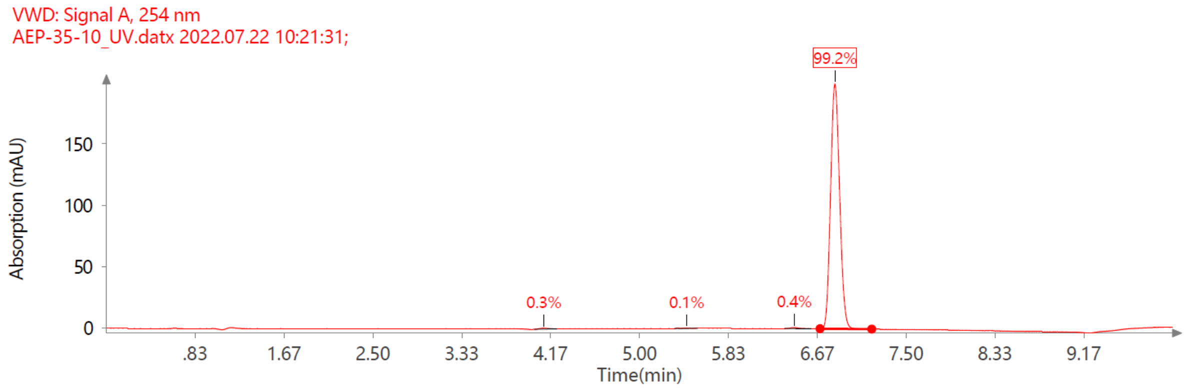

Supplementary Fig. 35. *HPLC chromatogram of* ***8e***.

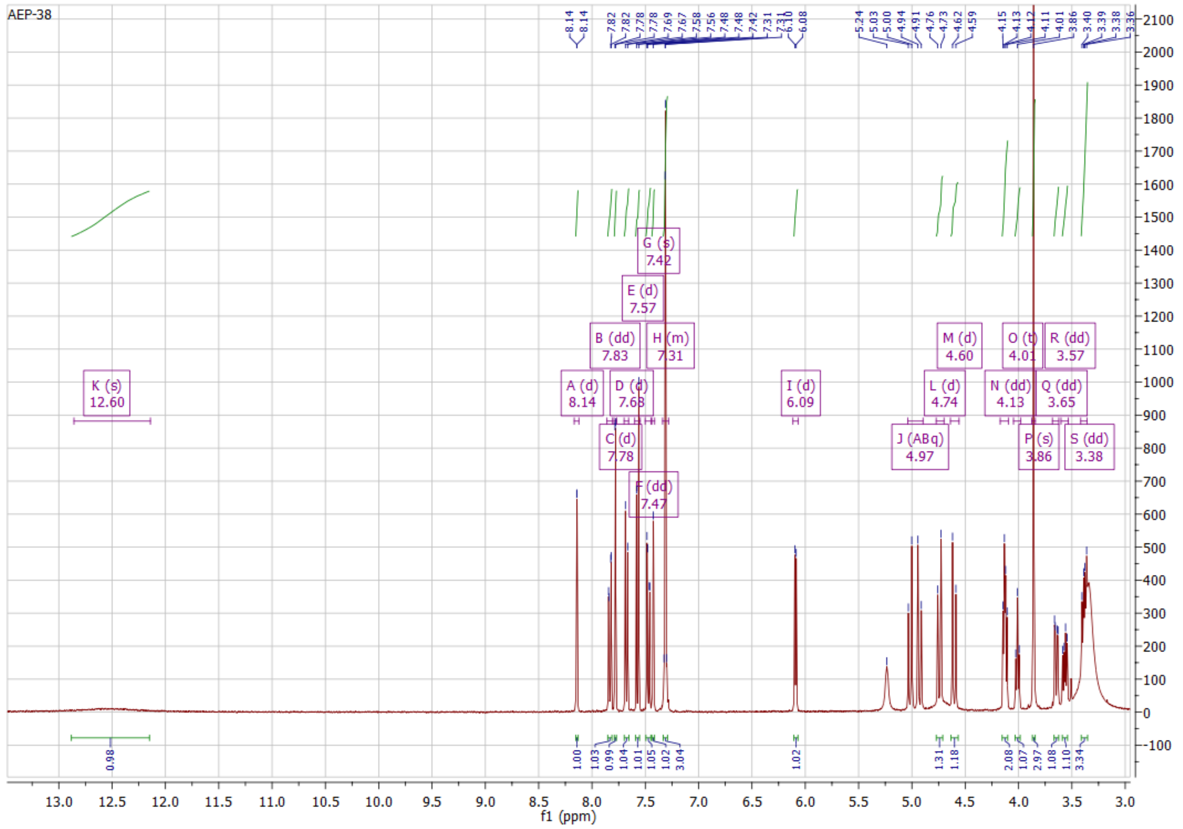

Supplementary Fig. 36. *^{1}H NMR (400 MHz, DMSO-d$_6$) of* ***8f***.

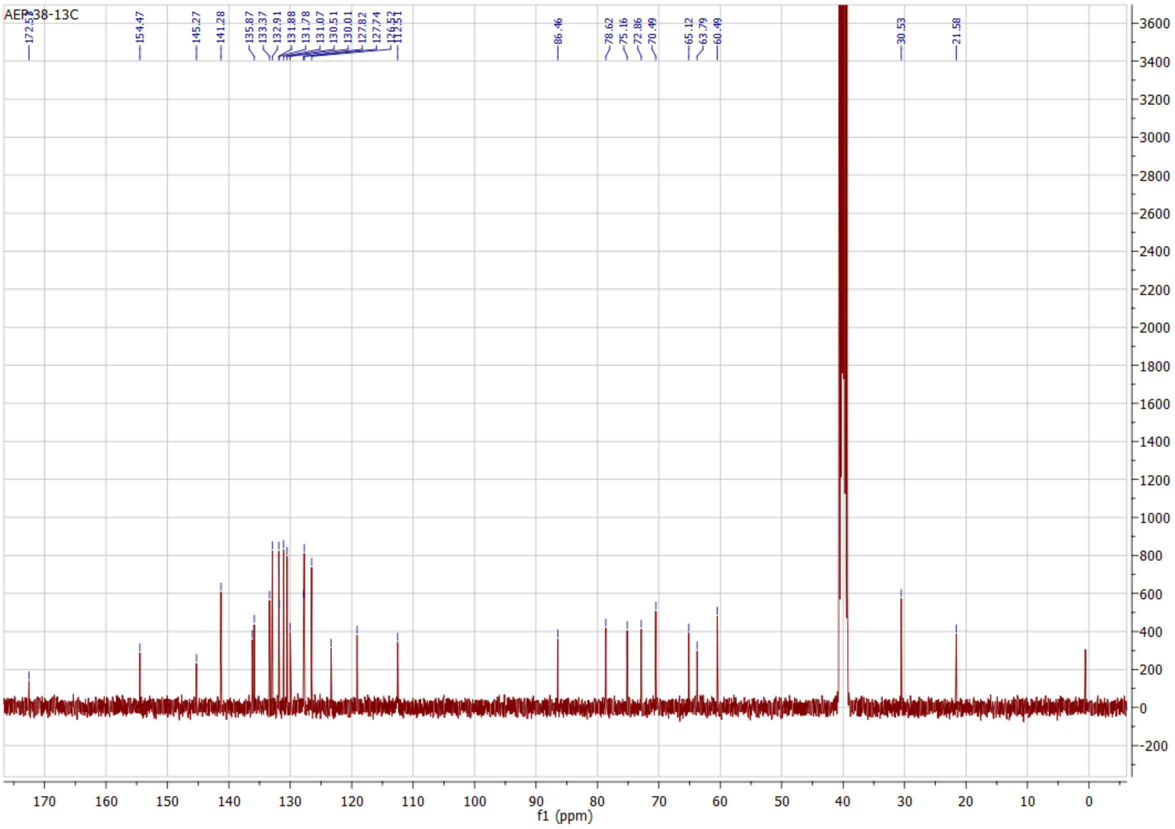

Supplementary Fig. 37. ^{13}C NMR (101 MHz, DMSO-d_6) of **8f**.

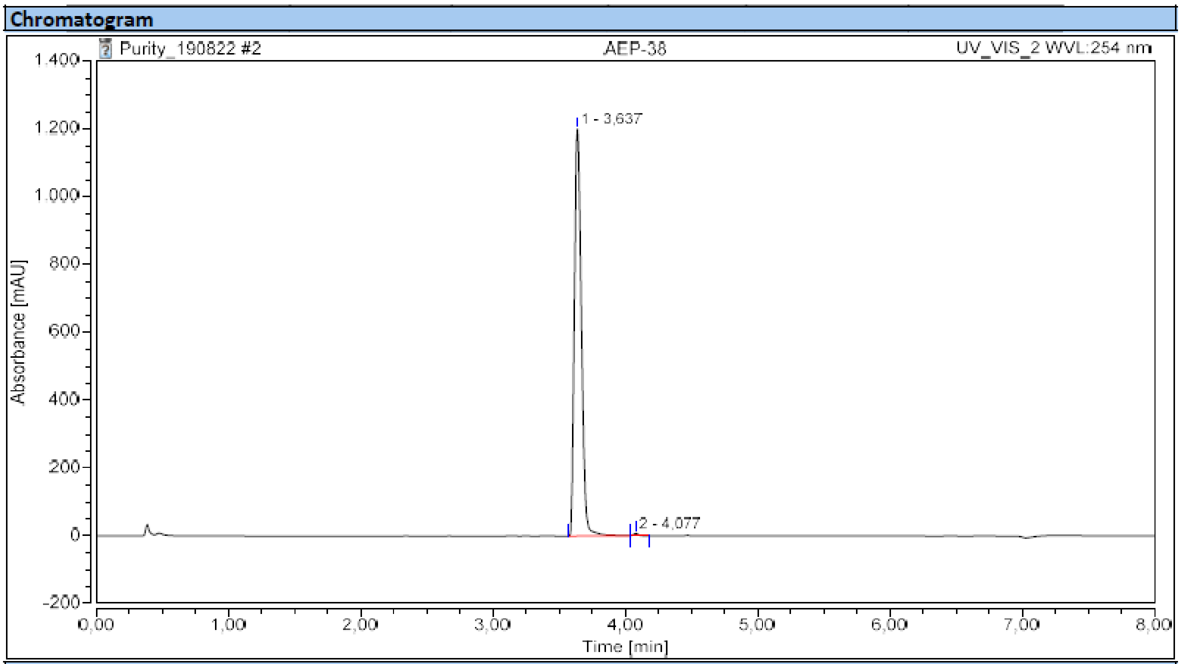

Integration Results

No.	Peak Name	Retention Time min	Area mAU*min	Height mAU	Relative Area %	Relative Height %	Amount n.a.
1		3,637	72,123	1201,095	99,59	99,40	n.a.
2		4,077	0,295	7,200	0,41	0,60	n.a.
**Total:**			**72,418**	**1208,296**	**100,00**	**100,00**	

Supplementary Fig. 38. HPLC chromatogram of **8f**.

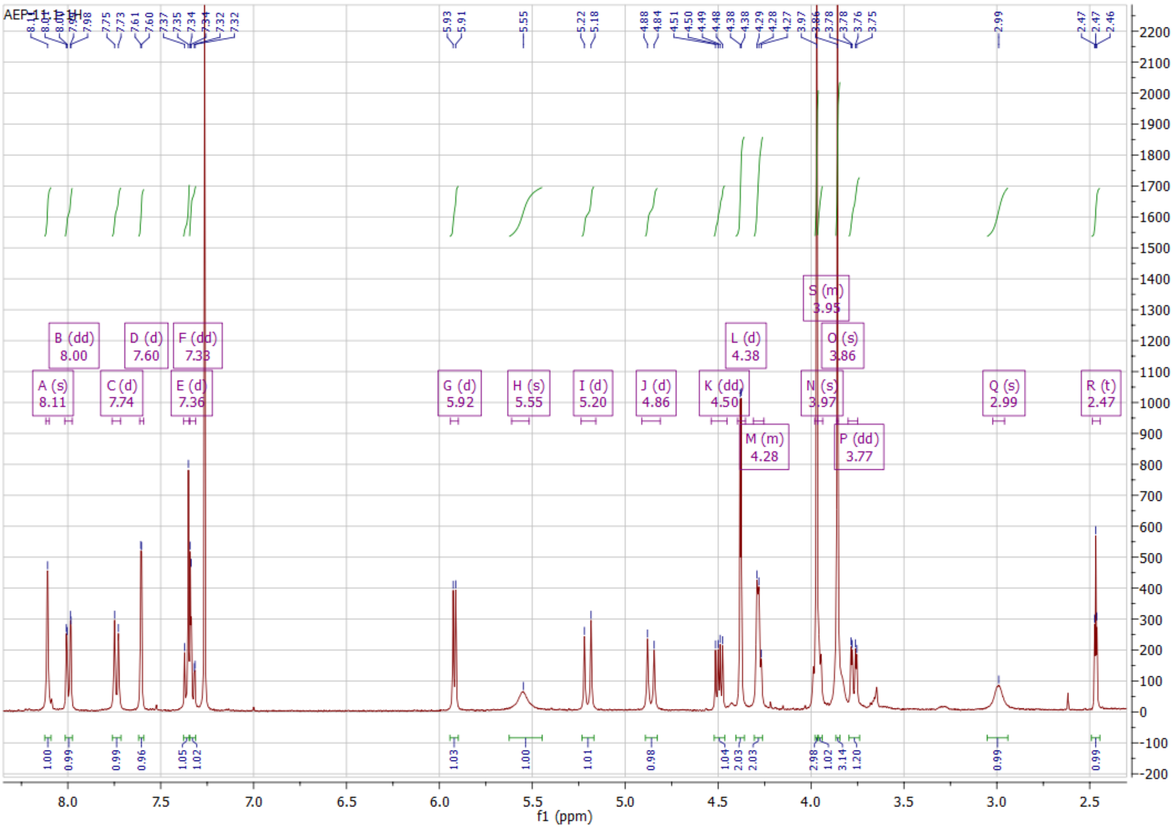

Supplementary Fig. 39. ^{1}H NMR (400 MHz, CDCl$_3$) of ***10****.*

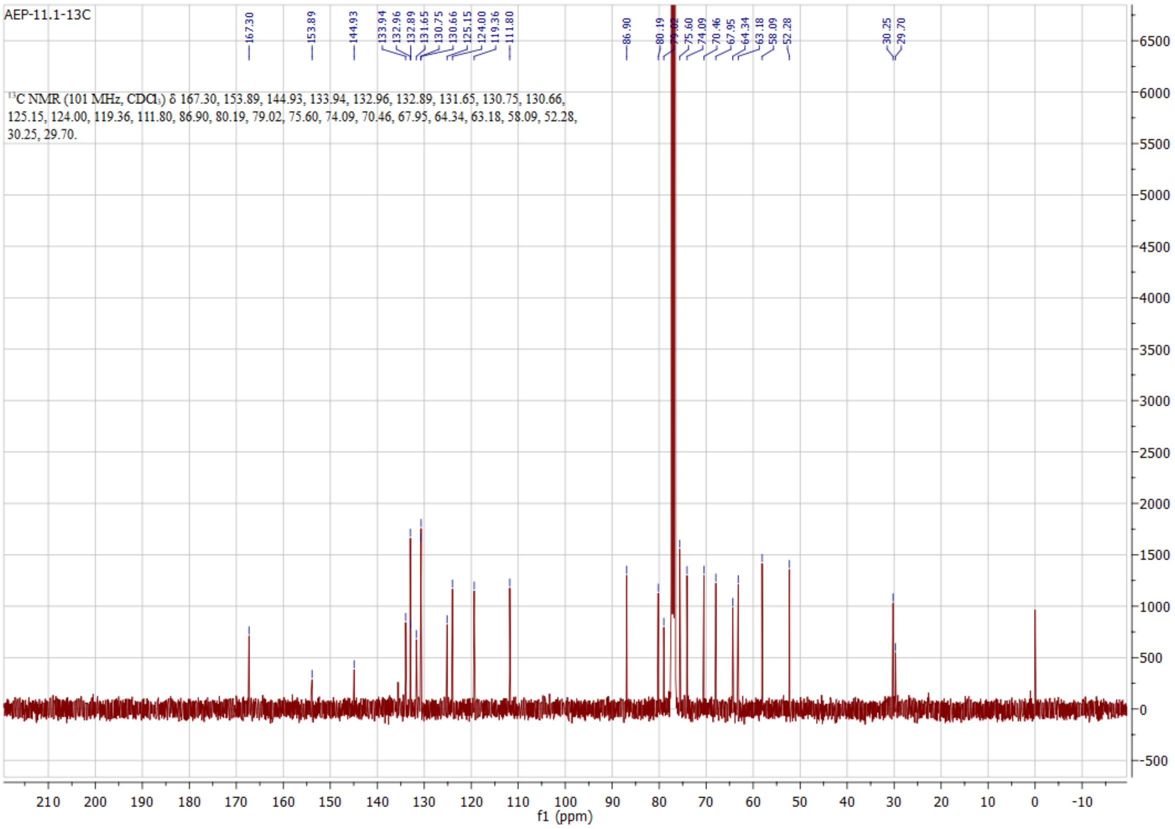

Supplementary Fig. 40. ^{13}C NMR (101 MHz, CDCl$_3$) of ***10****.*

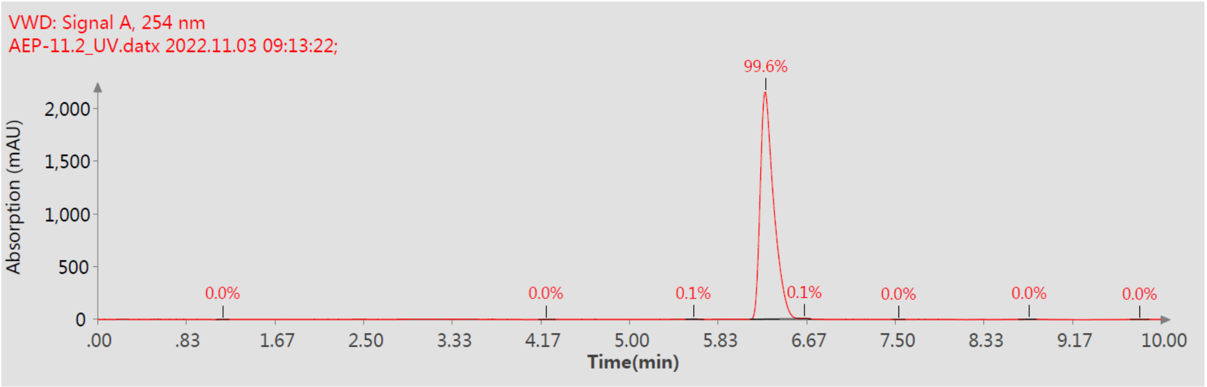

Supplementary Fig. 41. *HPLC chromatogram of **10**.*

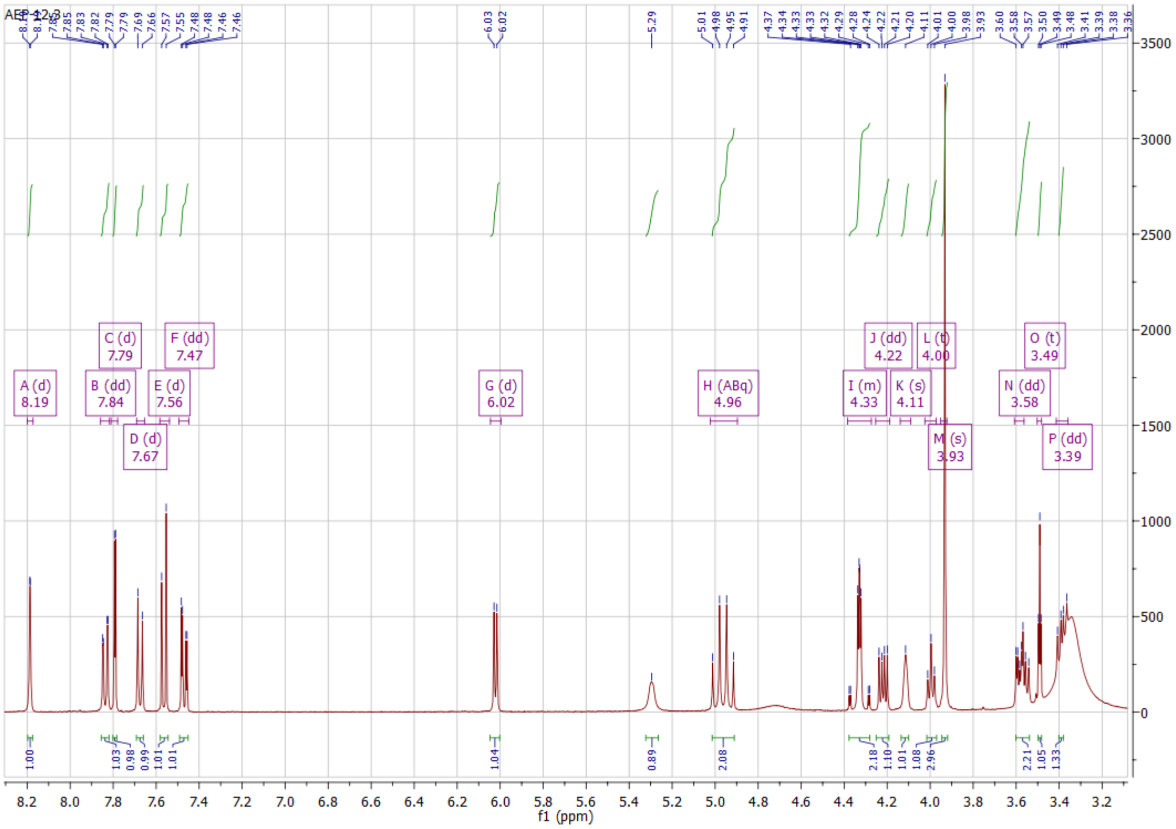

Supplementary Fig. 42. *^{1}H NMR (400 MHz, DMSO-d_6) of **11**.*

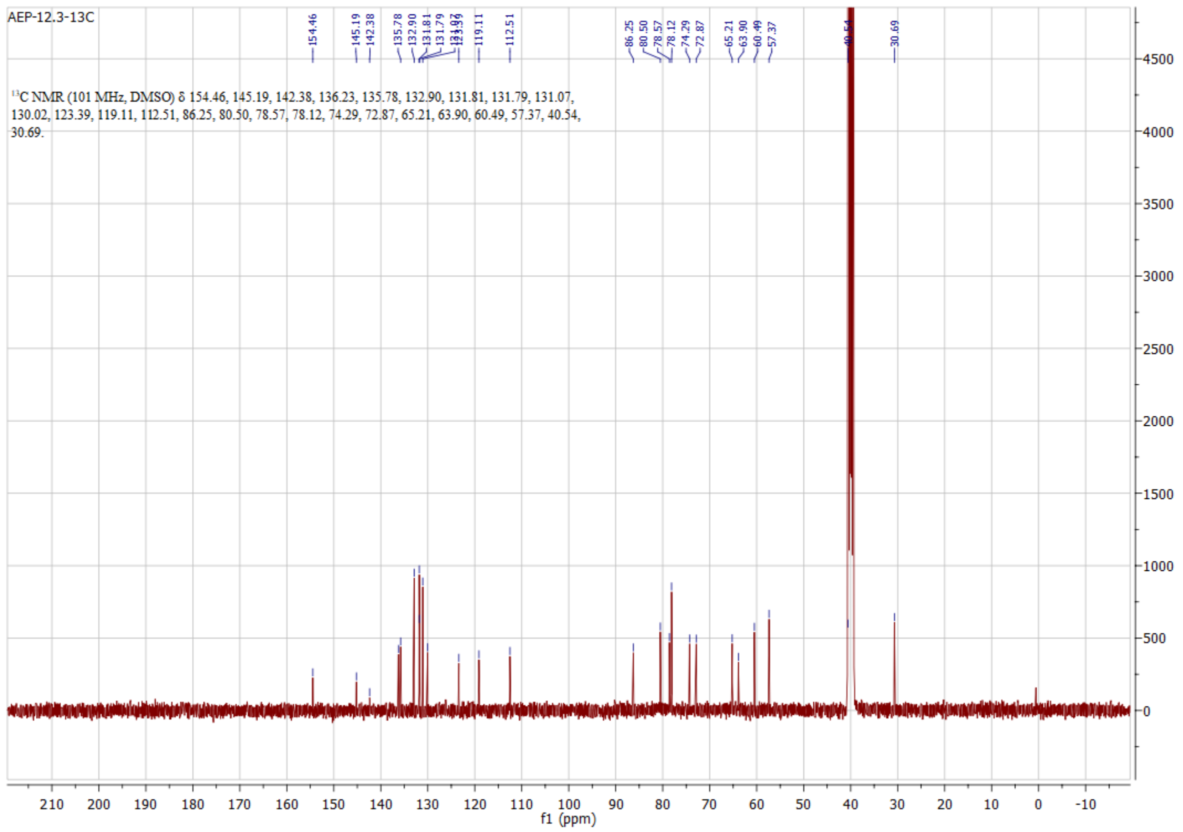

***Supplementary Fig. 43**. ^{13}C NMR (101 MHz, DMSO-d_6) of **11**.*

Chromatogram

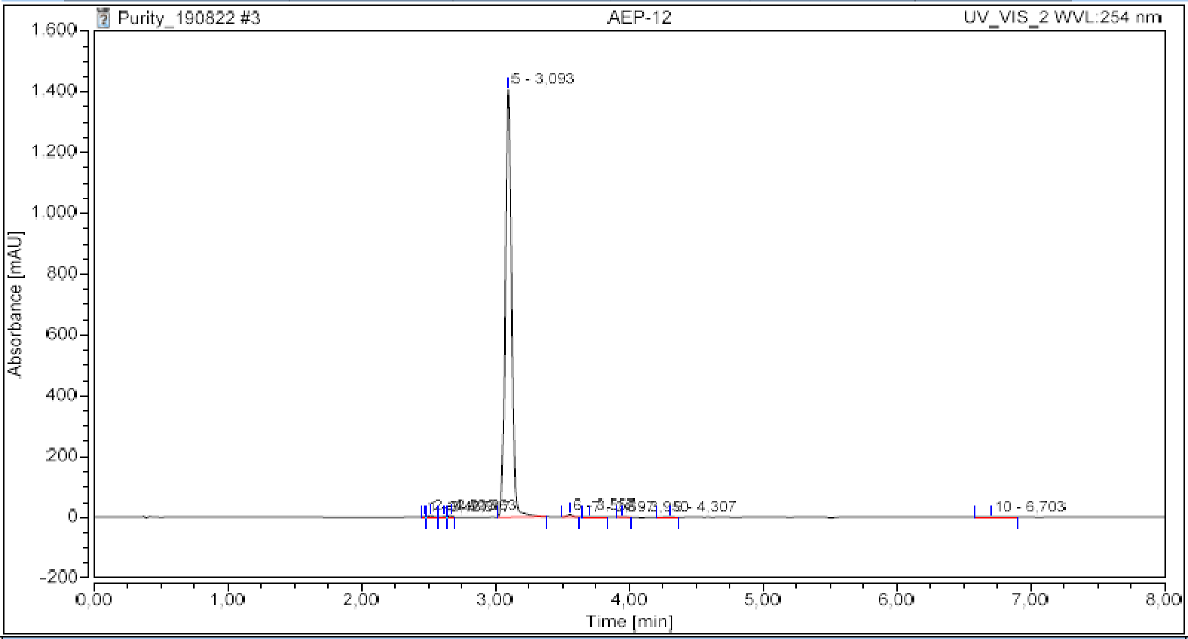

Integration Results

No.	Peak Name	Retention Time min	Area mAU*min	Height mAU	Relative Area %	Relative Height %	Amount n.a.
1		2,467	0,039	1,247	0,05	0,09	n.a.
2		2,513	0,170	4,817	0,21	0,34	n.a.
3		2,617	0,035	1,270	0,04	0,09	n.a.
4		2,663	0,161	4,752	0,20	0,33	n.a.
5		3,093	80,339	1407,881	98,90	98,38	n.a.
6		3,557	0,341	8,589	0,42	0,60	n.a.
7		3,697	0,049	0,850	0,06	0,06	n.a.
8		3,950	0,041	0,908	0,05	0,06	n.a.
9		4,307	0,028	0,496	0,03	0,03	n.a.
10		6,703	0,031	0,276	0,04	0,02	n.a.
**Total:**			**81,235**	**1431,087**	**100,00**	**100,00**	

Supplementary Fig. 44. *HPLC chromatogram of **11**.*

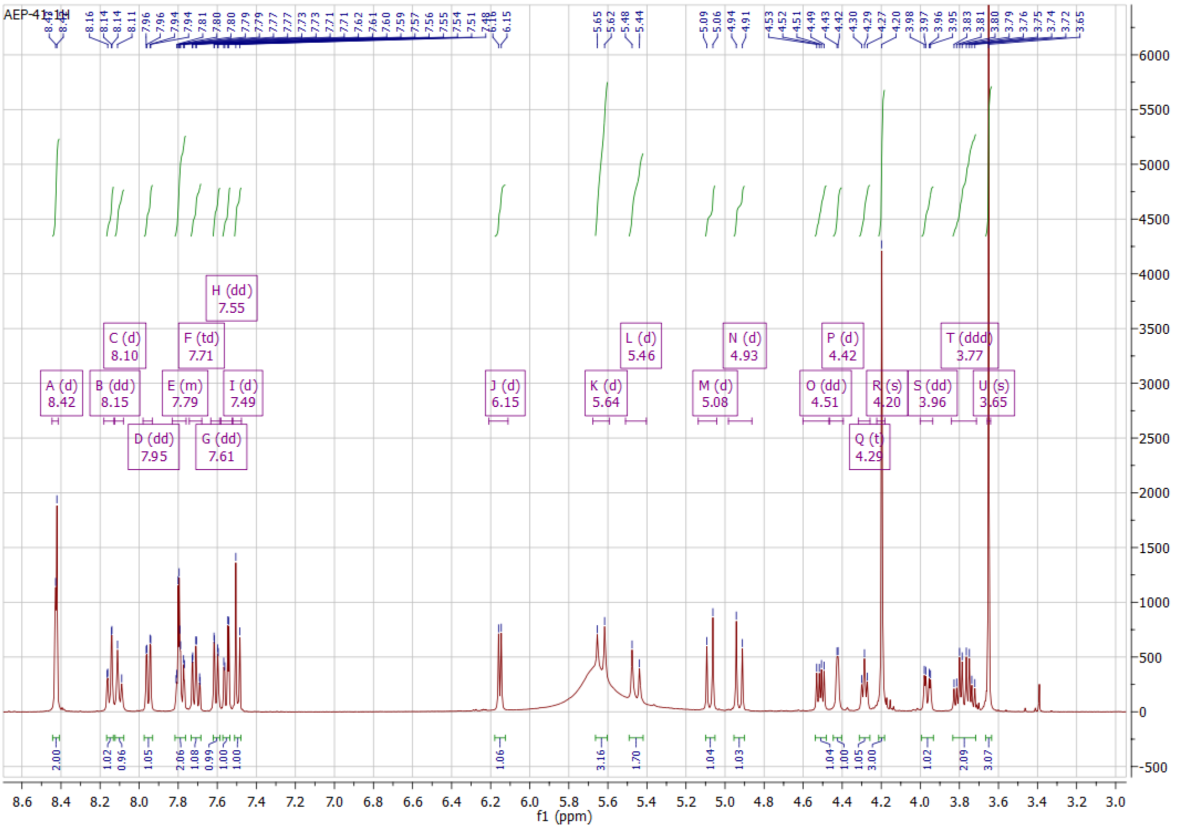

Supplementary Fig. 45. ^{1}H NMR (400 MHz, acetone-d_6) of **15a**.

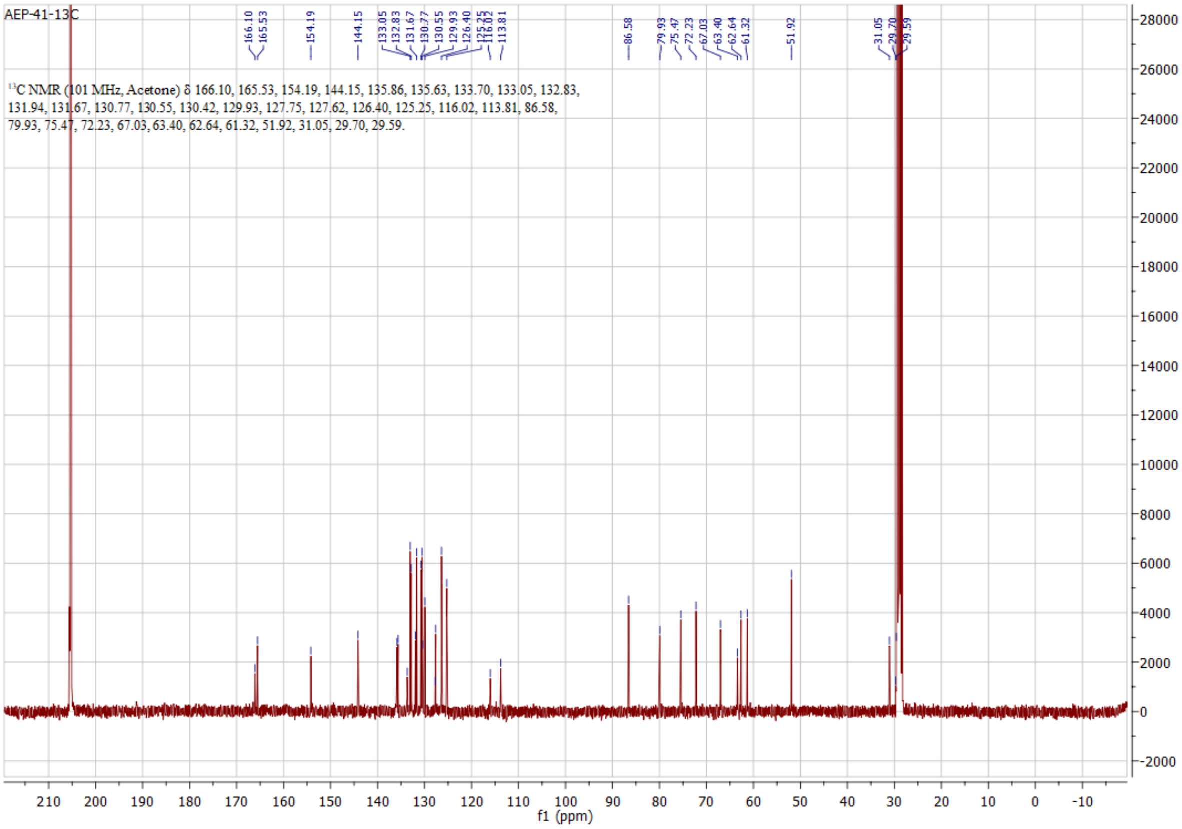

Supplementary Fig. 46. ^{13}C NMR (101 MHz, acetone-d_6) of **15a**.

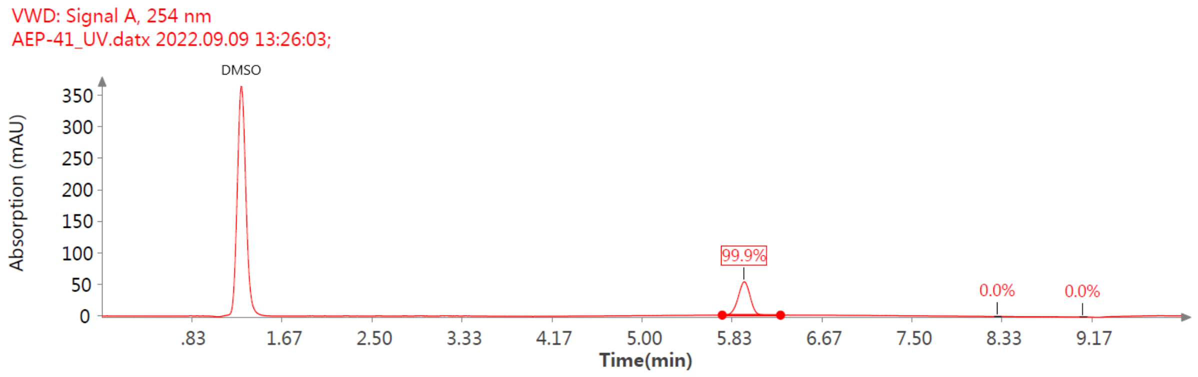

***Supplementary Fig. 47**. HPLC chromatogram of **15a**.*

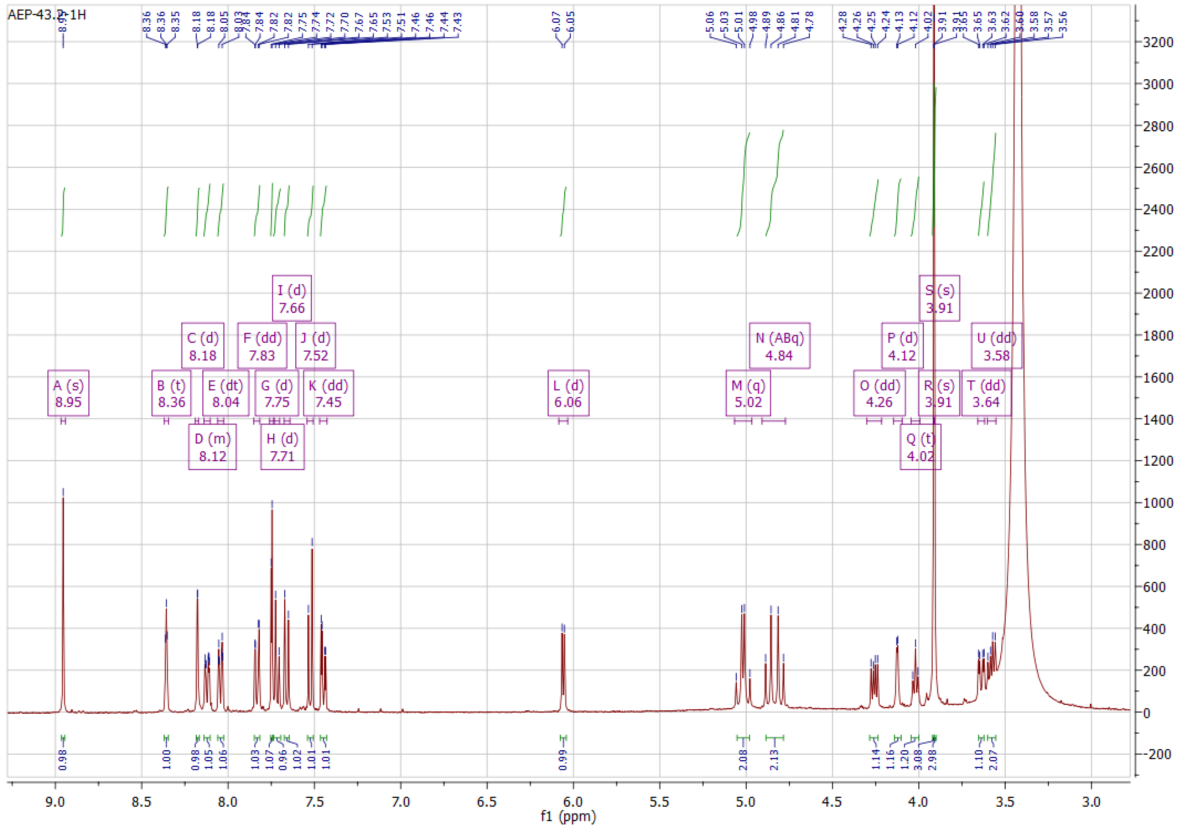

***Supplementary Fig. 48**. ^{1}H NMR (400 MHz, acetone-d_6) of **15b**.*

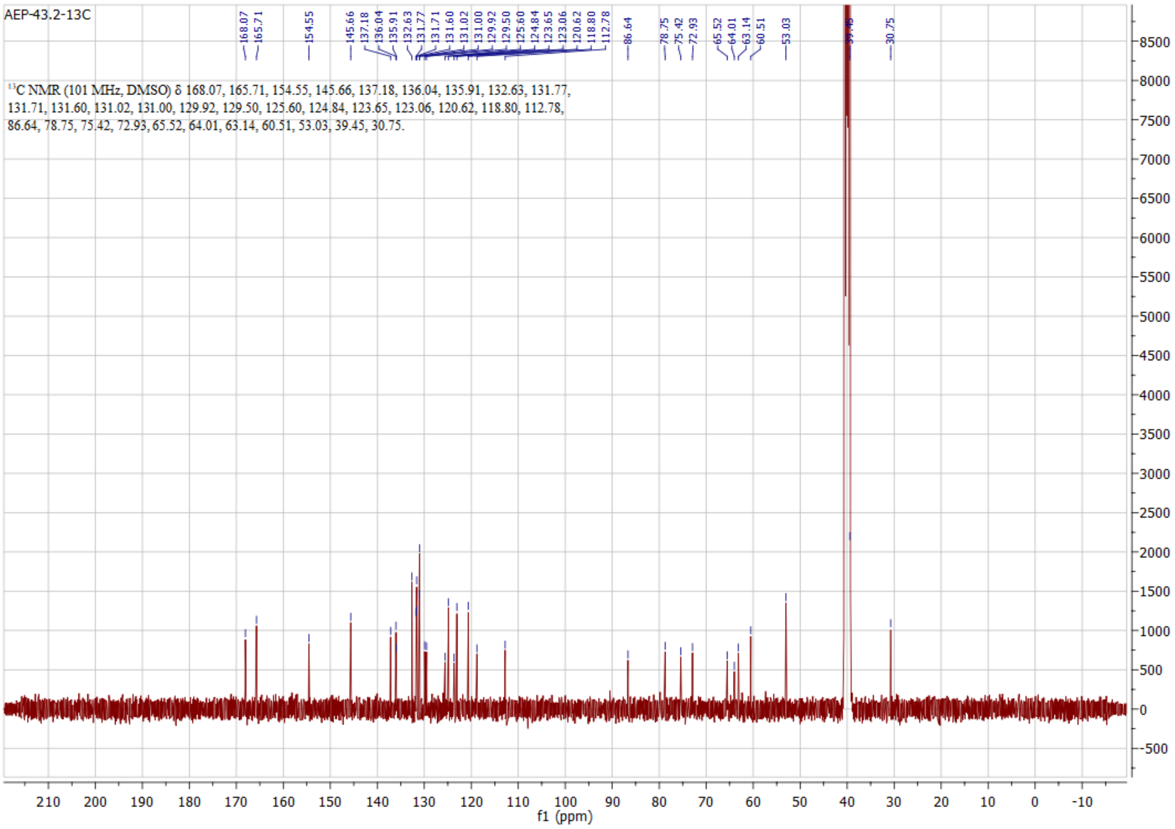

Supplementary Fig. 49. *^{13}C NMR (101 MHz, DMSO-d_6) of **15b***.

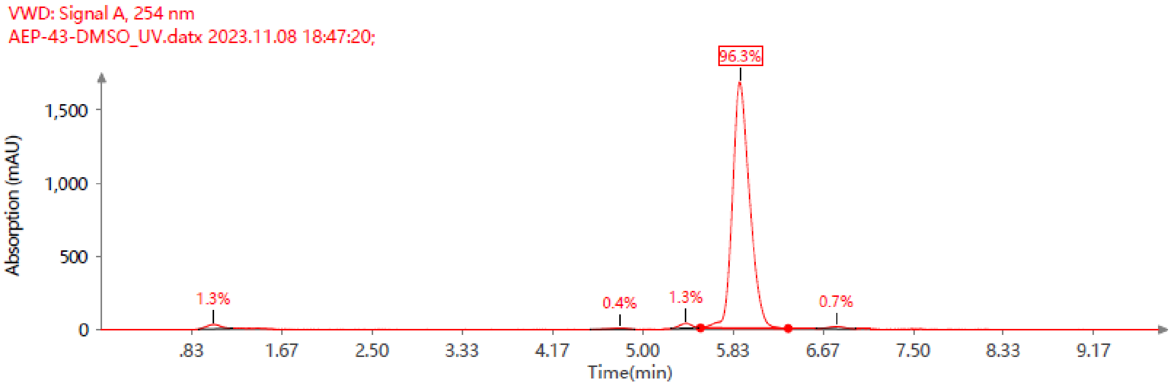

Supplementary Fig. 50. *HPLC chromatogram of **15b***.

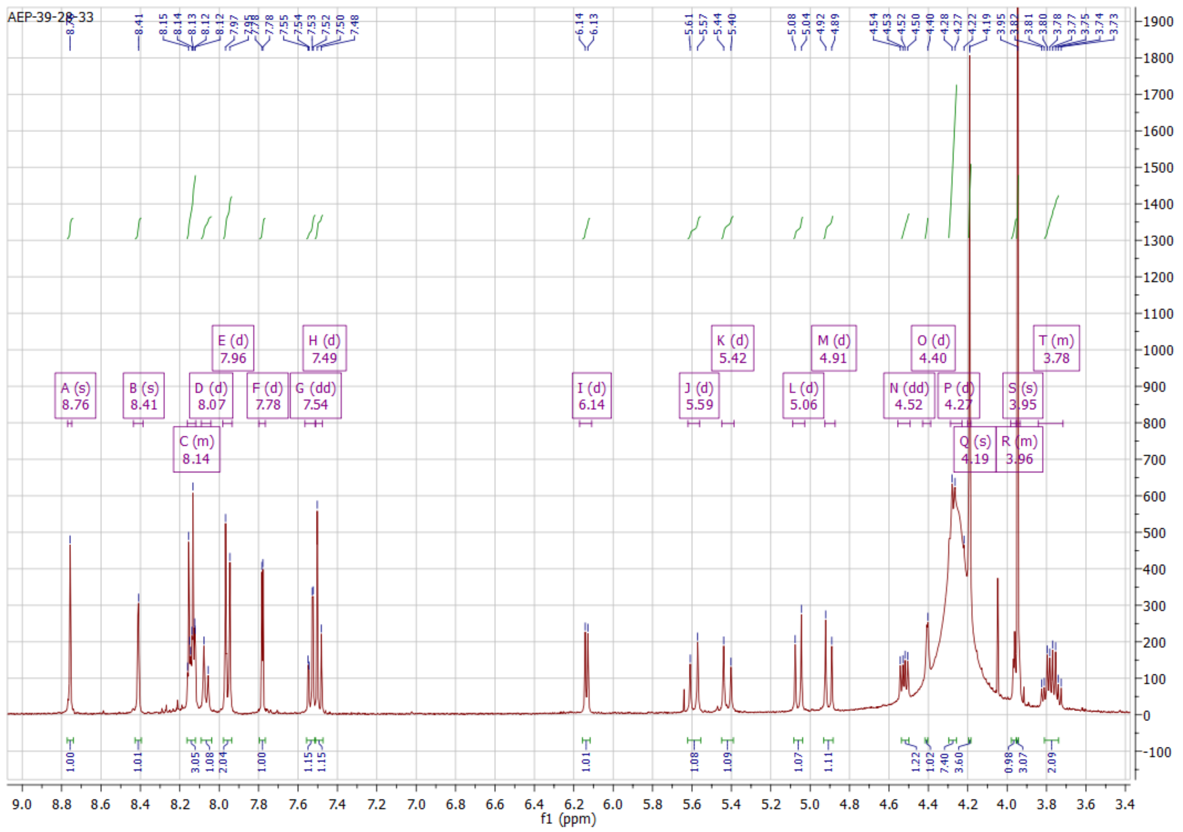

Supplementary Fig. 51. *^{1}H NMR (400 MHz, acetone-d$_6$) of **15c**.*

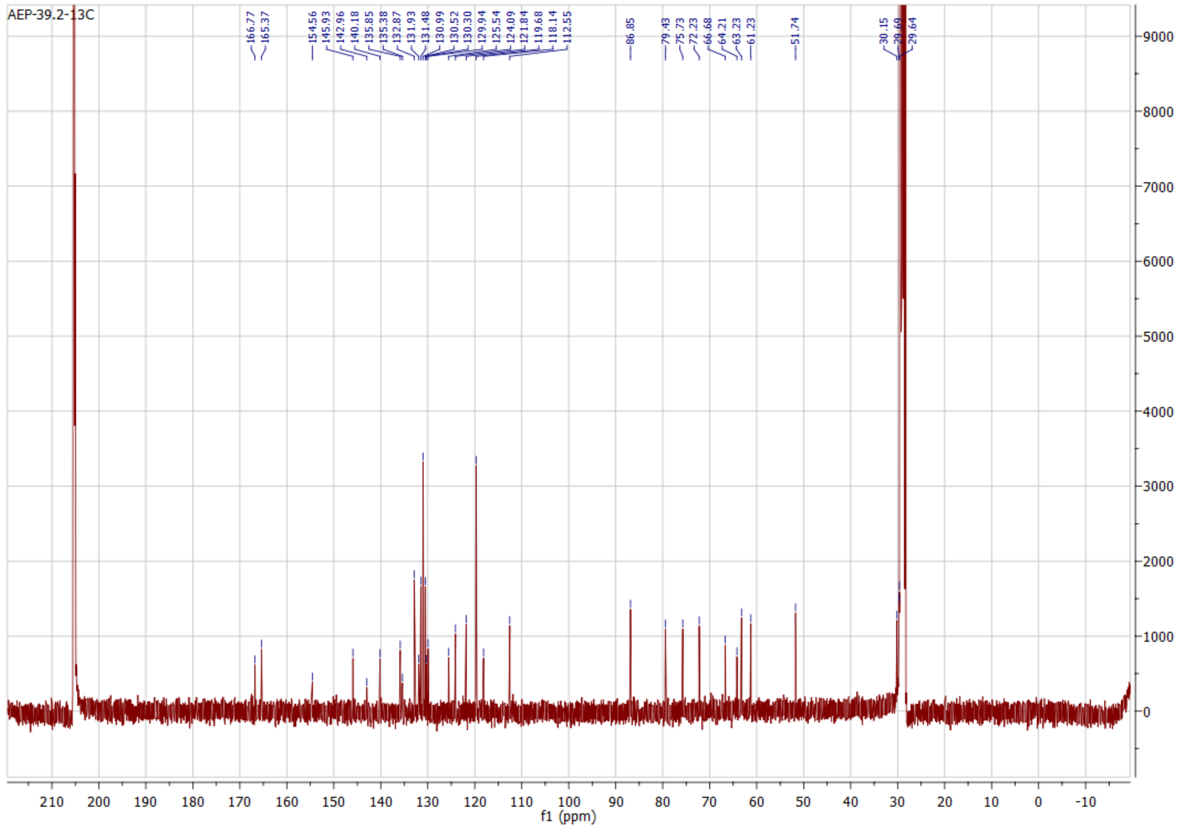

Supplementary Fig. 52. *^{13}C NMR (101 MHz, acetone-d$_6$) of **15c**.*

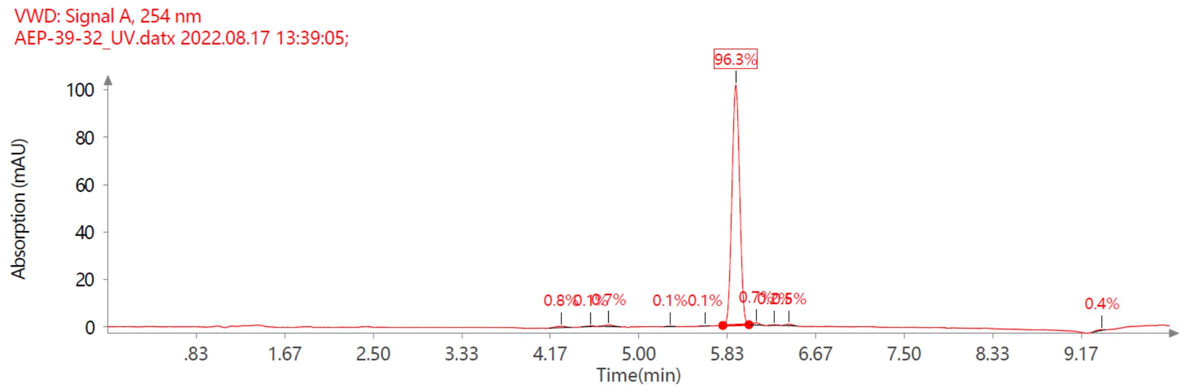

Supplementary Fig. 53. *HPLC chromatogram of **15c**.*

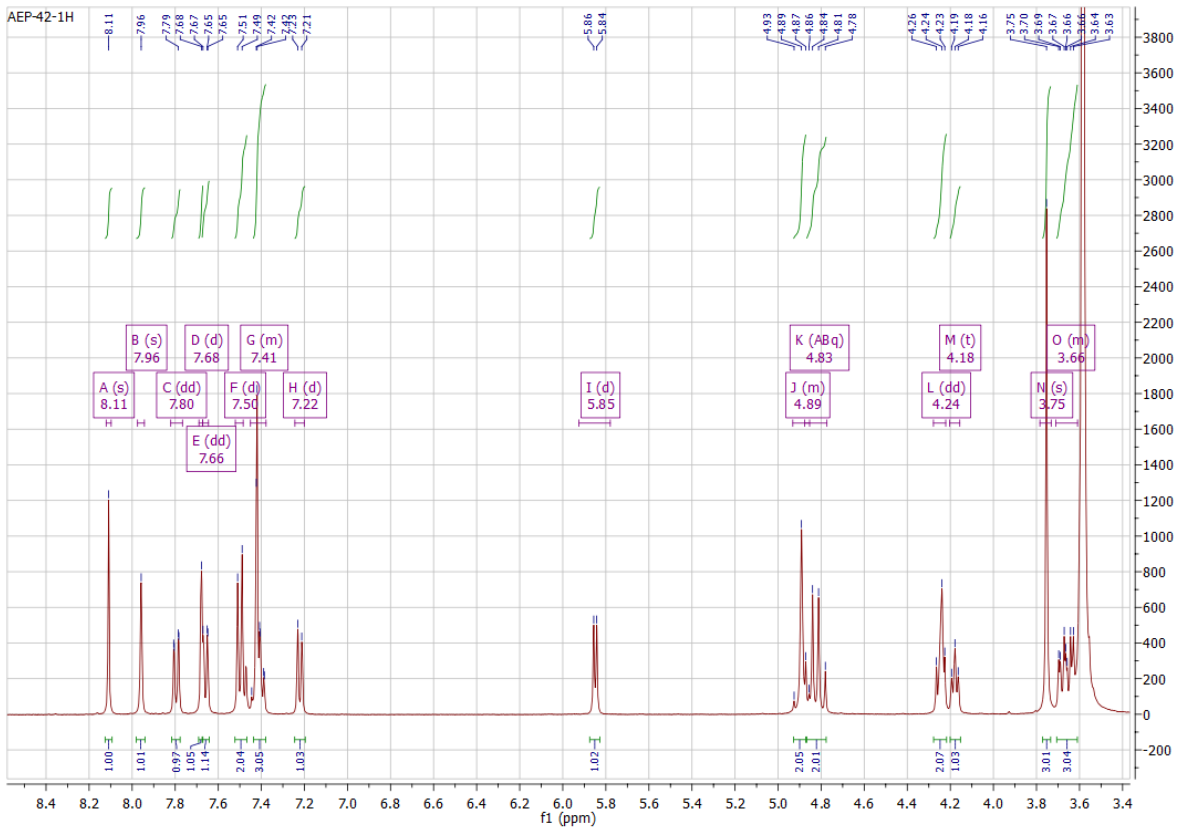

Supplementary Fig. 54. *^{1}H NMR (400 MHz, CD_3CN) of **16a**.*

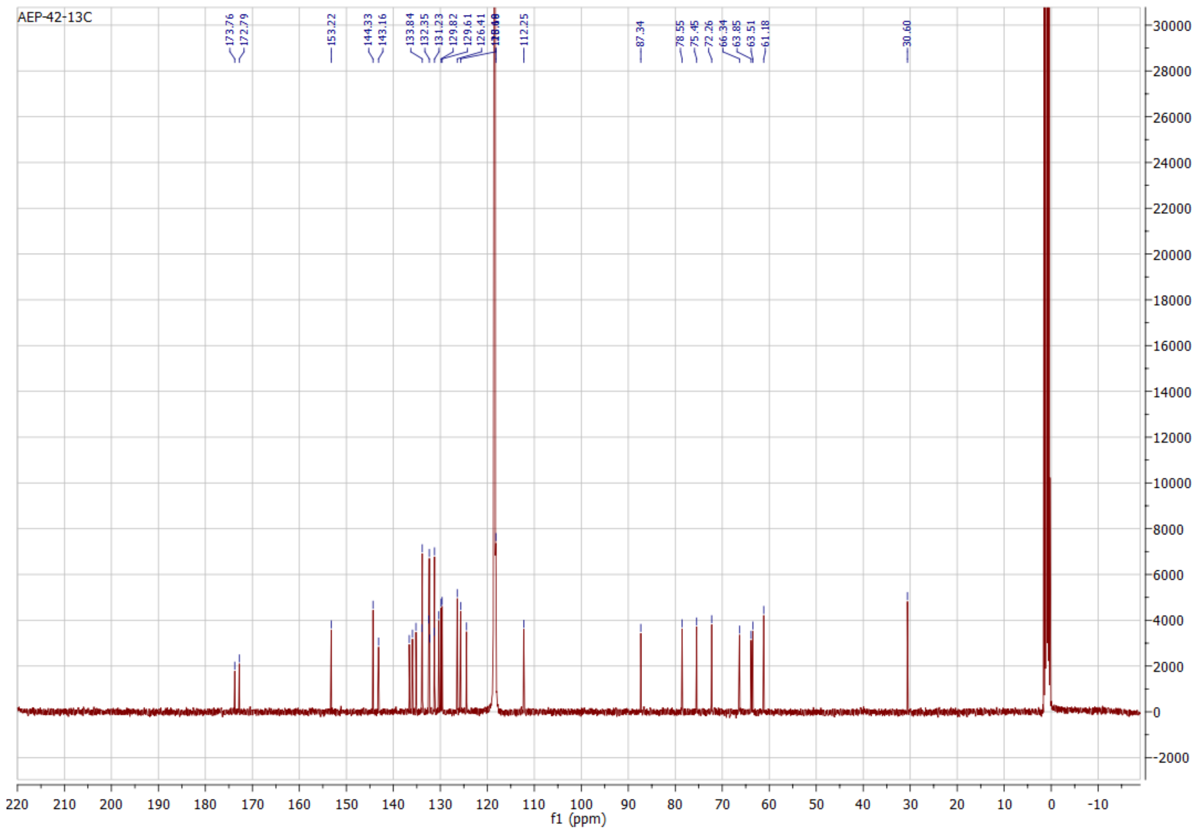

Supplementary Fig. 55. *^{13}C NMR (101 MHz, CD_3CN) of **16a**.*

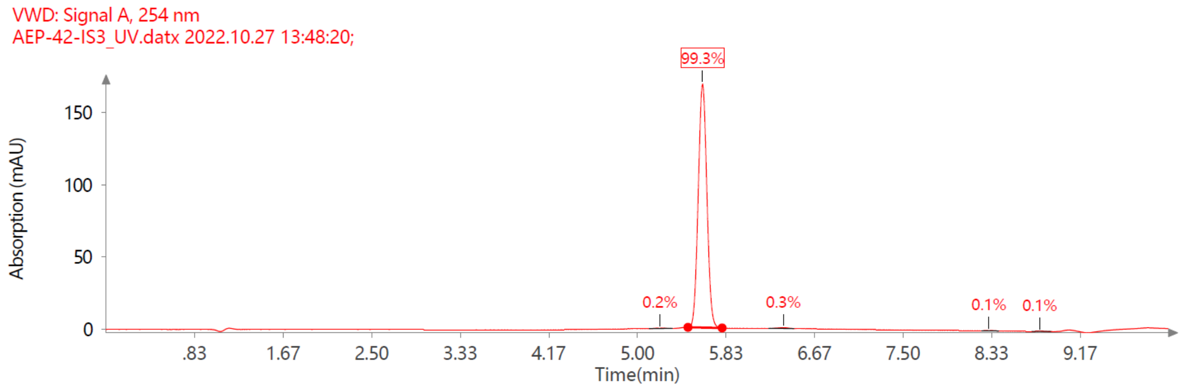

Supplementary Fig. 56. *HPLC chromatogram of **16a**.*

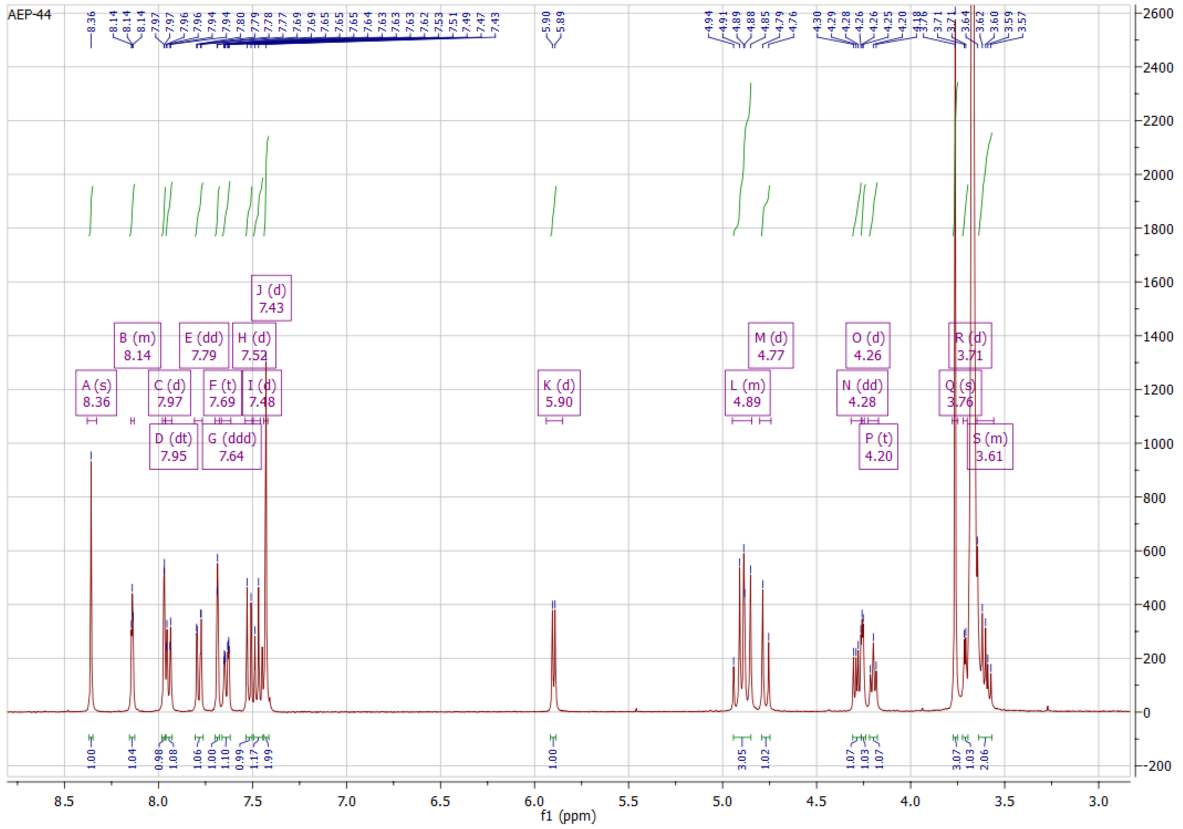

Supplementary Fig. 57. *^{1}H NMR (400 MHz, CD_3CN) of* ***16b***.

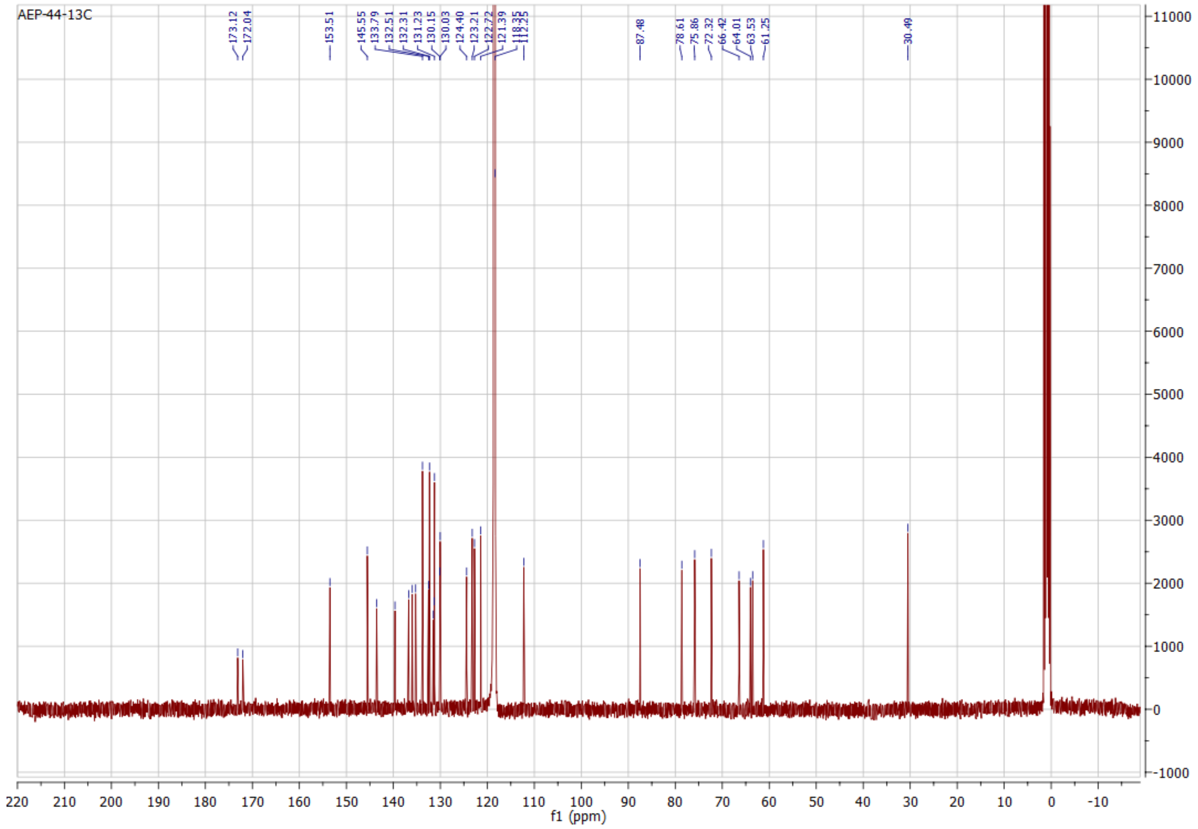

Supplementary Fig. 58. *^{13}C NMR (101 MHz, CD_3CN) of* ***16b***.

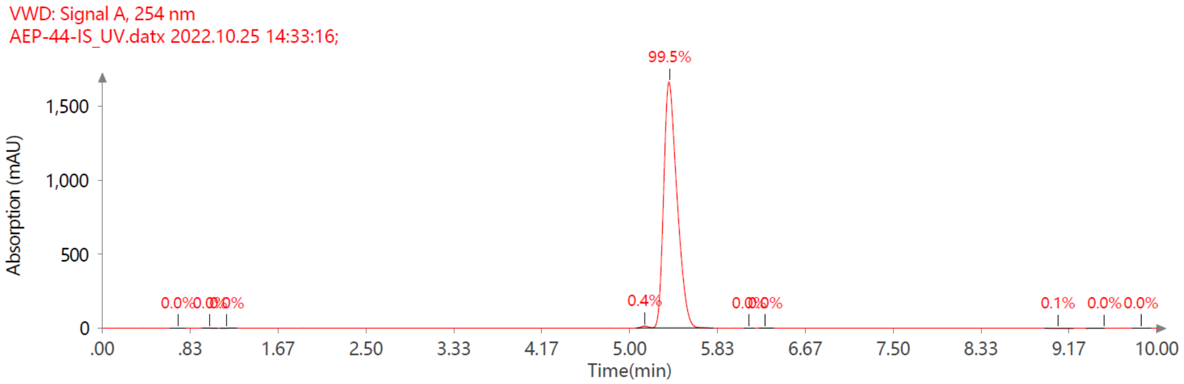

Supplementary Fig. 59. *HPLC chromatogram of* ***16b***.

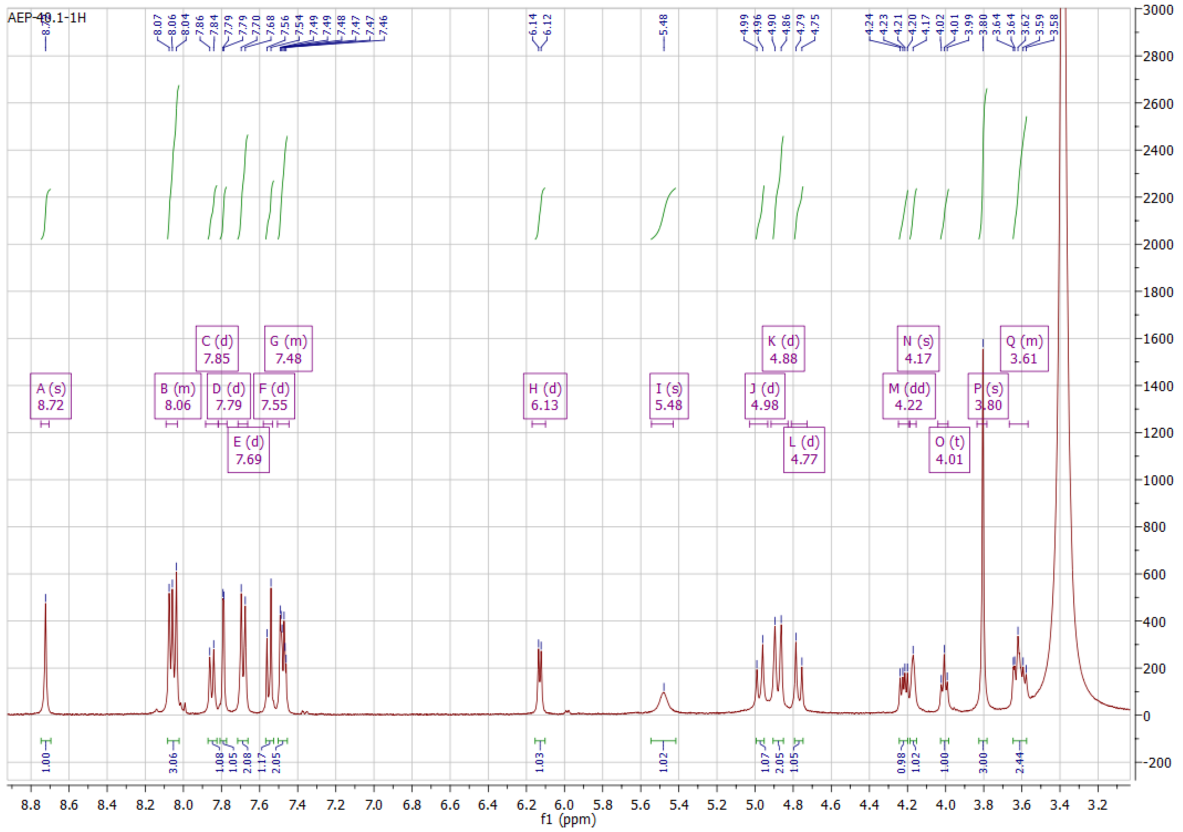

Supplementary Fig. 60. *^{1}H NMR (400 MHz, DMSO-d_6) of* ***16c***.

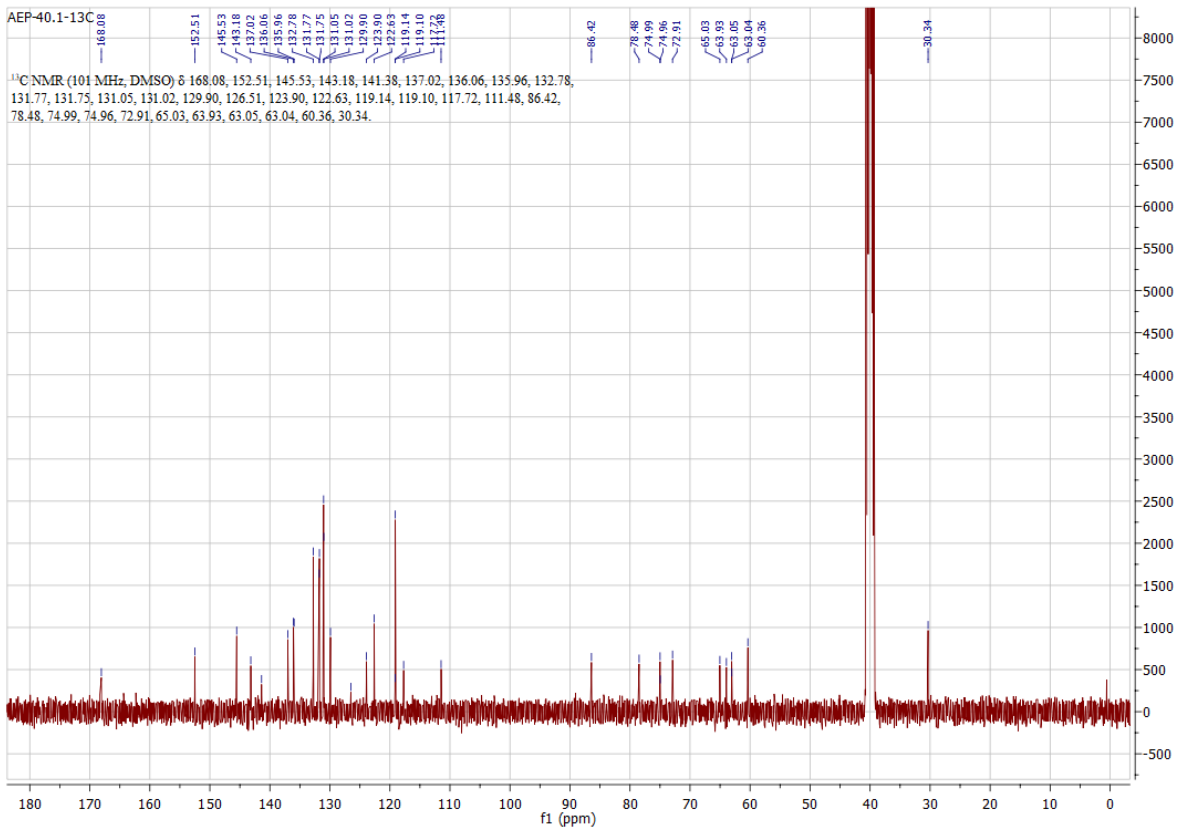

Supplementary Fig. 61. *^{13}C NMR (101 MHz, DMSO-d$_6$) of **16c**.*

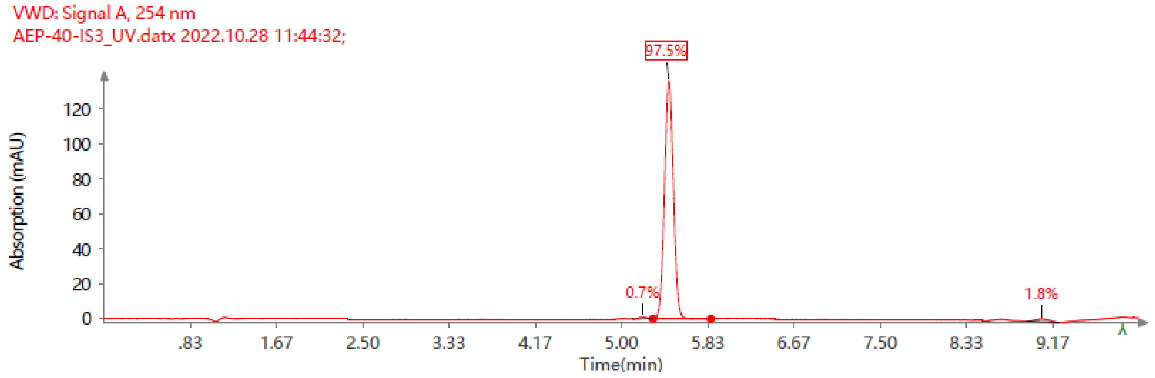

Supplementary Fig. 62. *HPLC chromatogram of **16c**.*

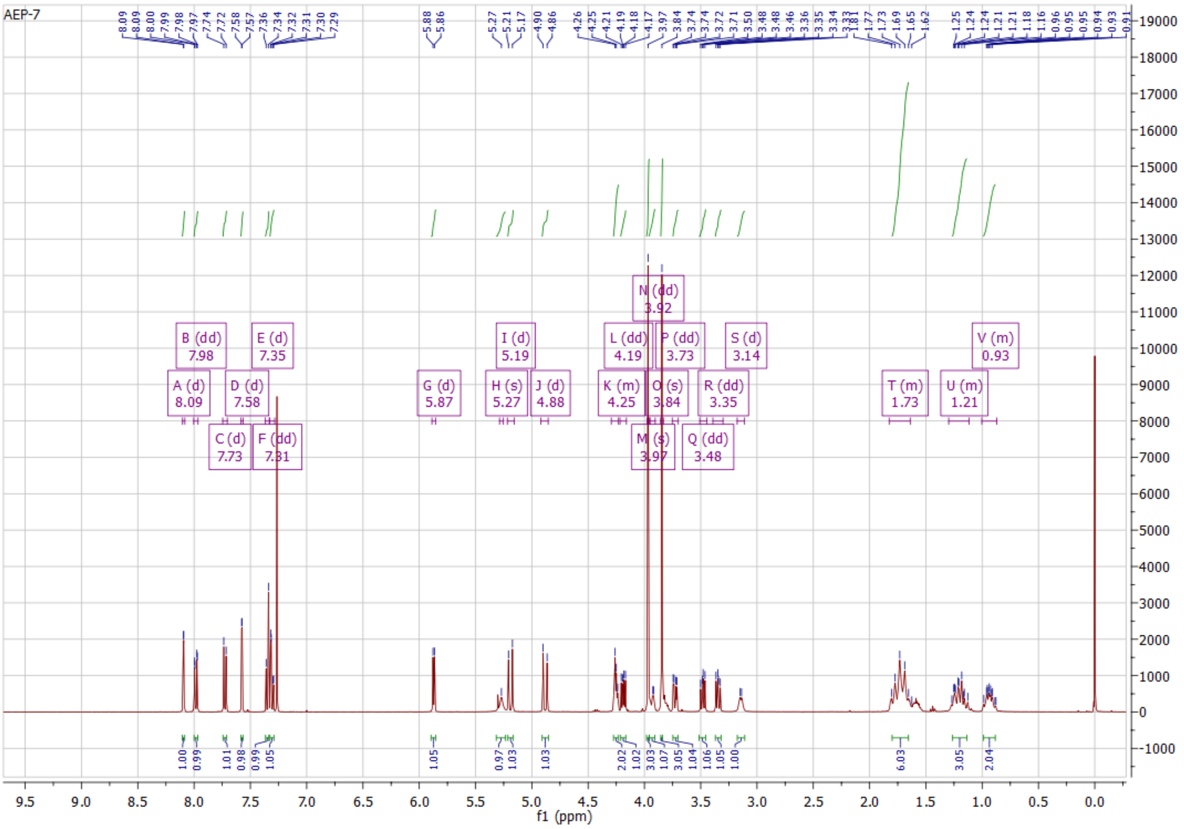

Supplementary Fig. 63. ^{1}H NMR (400 MHz, $CDCl_3$) of **18**.

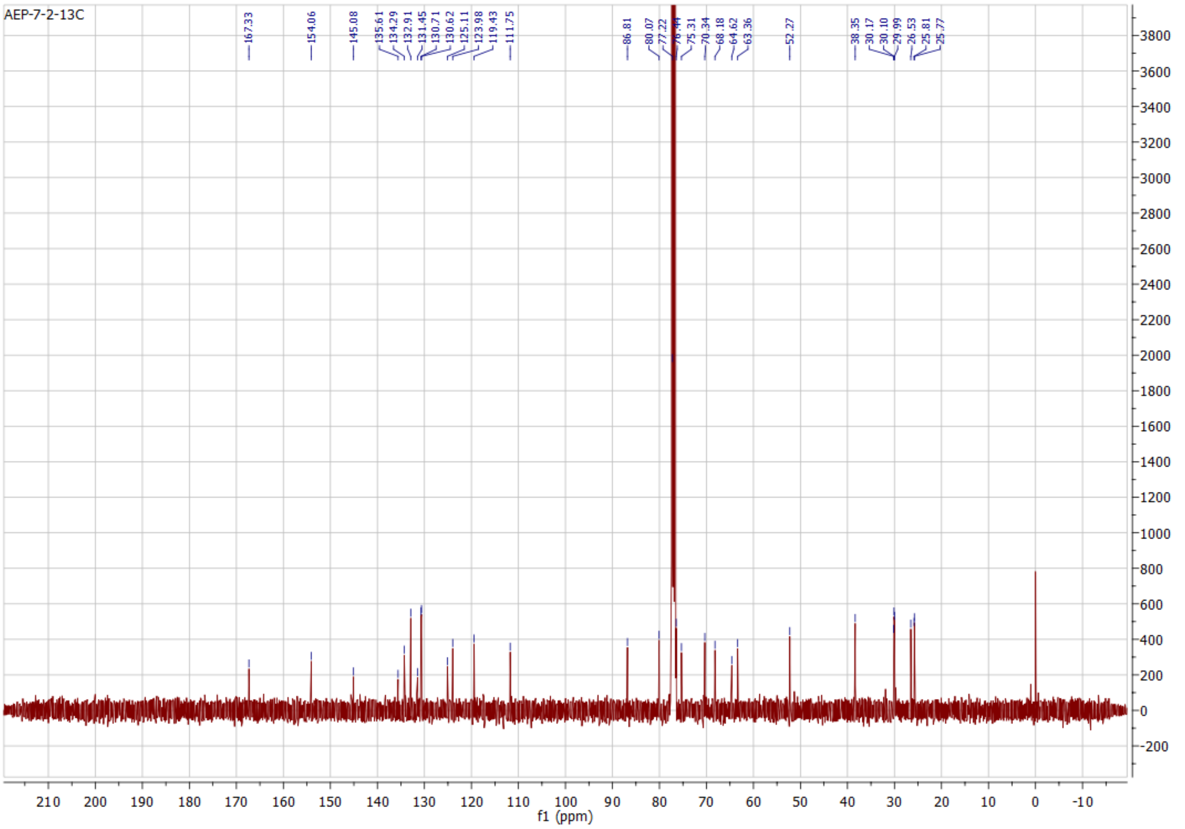

Supplementary Fig. 64. ^{13}C NMR (101 MHz, $CDCl_3$) of **18**.

Chromatogram

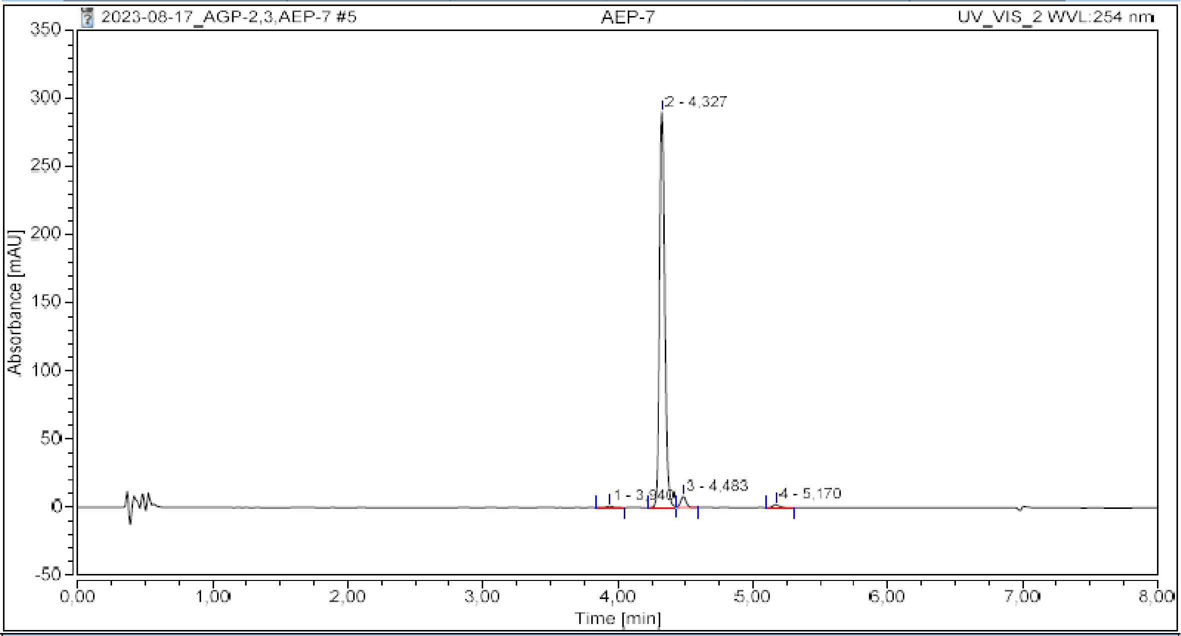

Integration Results

No.	Peak Name	Retention Time min	Area mAU*min	Height mAU	Relative Area %	Relative Height %	Amount n.a.
1		3,940	0,045	0,948	0,33	0,31	n.a.
2		4,327	13,249	290,511	96,32	96,24	n.a.
3		4,483	0,358	8,221	2,60	2,72	n.a.
4		5,170	0,103	2,181	0,75	0,72	n.a.
**Total:**			**13,755**	**301,860**	**100,00**	**100,00**	

Supplementary Fig. 65*. HPLC chromatogram of* ***18****.*

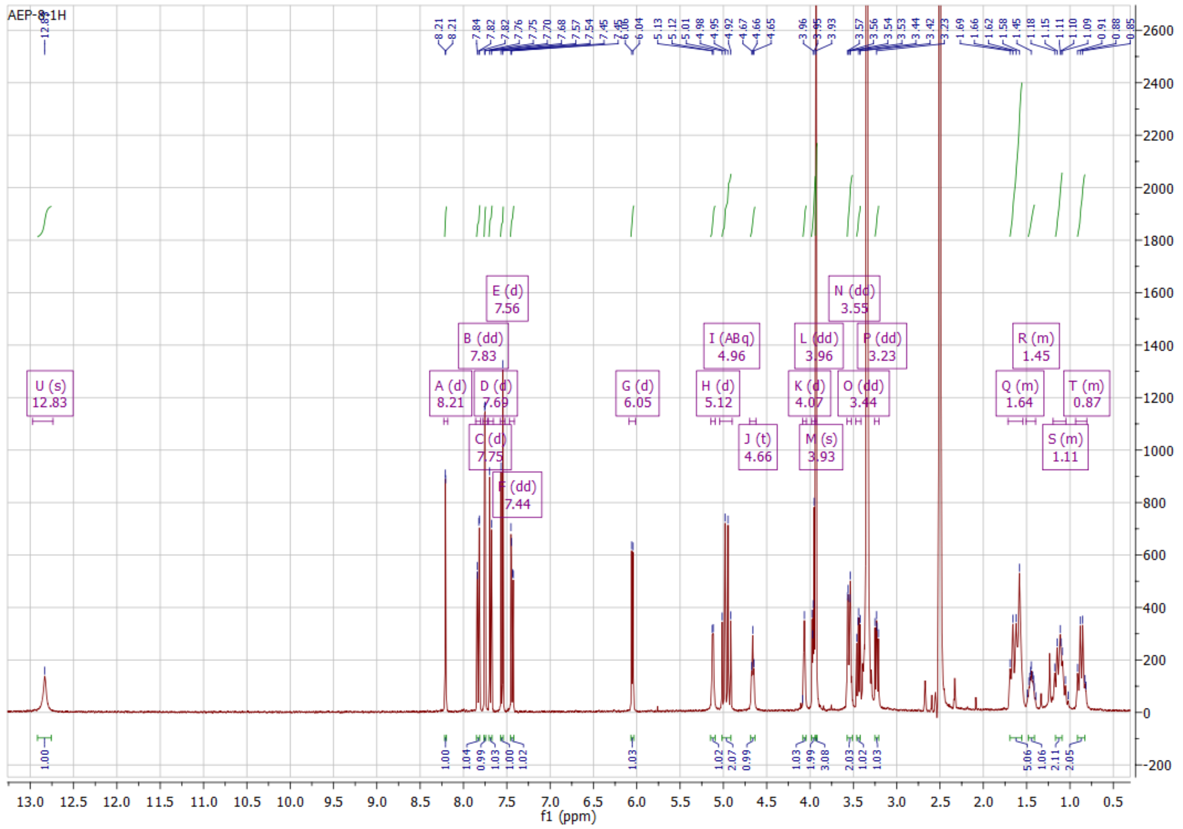

Supplementary Fig. 66. *^{1}H NMR (400 MHz, DMSO-d$_6$) of **19**.*

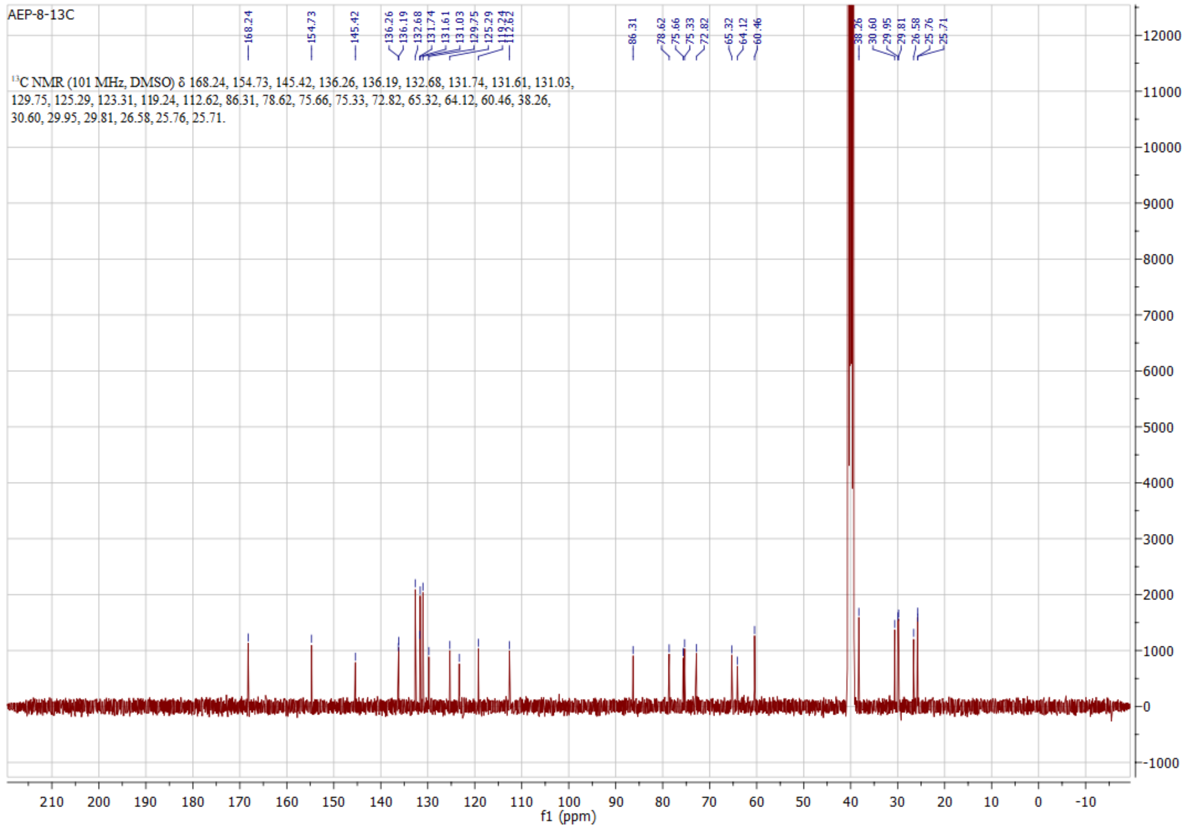

Supplementary Fig. 67. *^{13}C NMR (101 MHz, DMSO-d$_6$) of **19**.*

Chromatogram

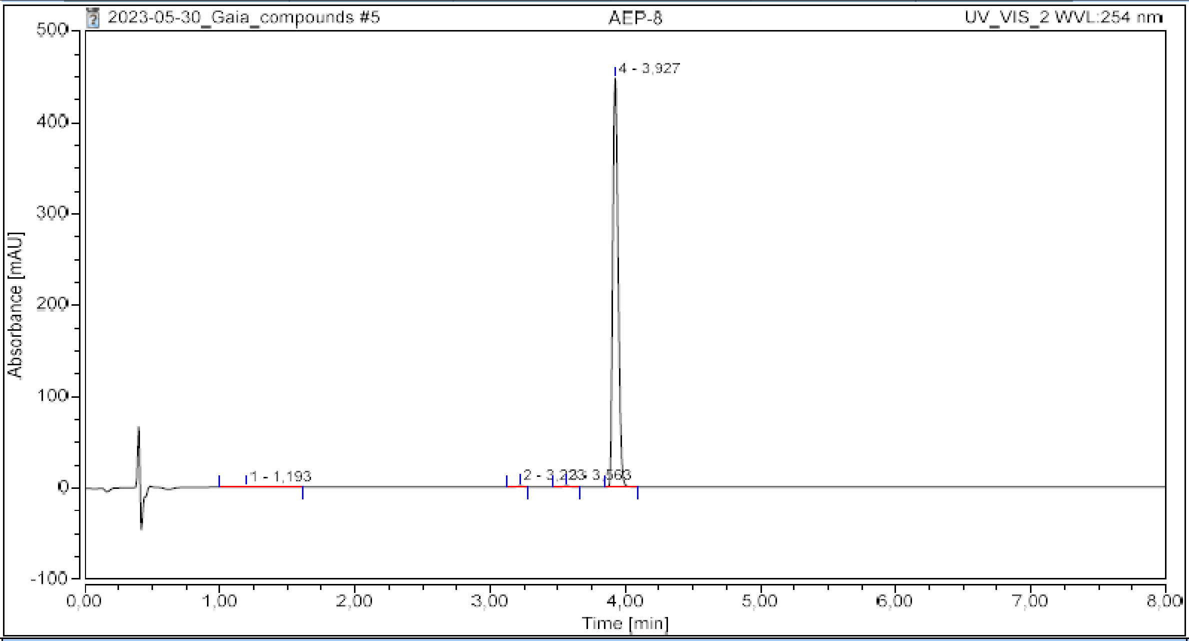

Integration Results

No.	Peak Name	Retention Time<br>min	Area<br>mAU*min	Height<br>mAU	Relative Area<br>%	Relative Height<br>%	Amount<br>n.a.
1		1,193	0,077	0,223	0,37	0,05	n.a.
2		3,223	0,067	1,243	0,32	0,28	n.a.
3		3,563	0,070	1,542	0,34	0,34	n.a.
4		3,927	20,456	446,763	98,96	99,33	n.a.
**Total:**			**20,671**	**449,771**	**100,00**	**100,00**	

***Supplementary Fig. 68**. HPLC chromatogram of **19**.*

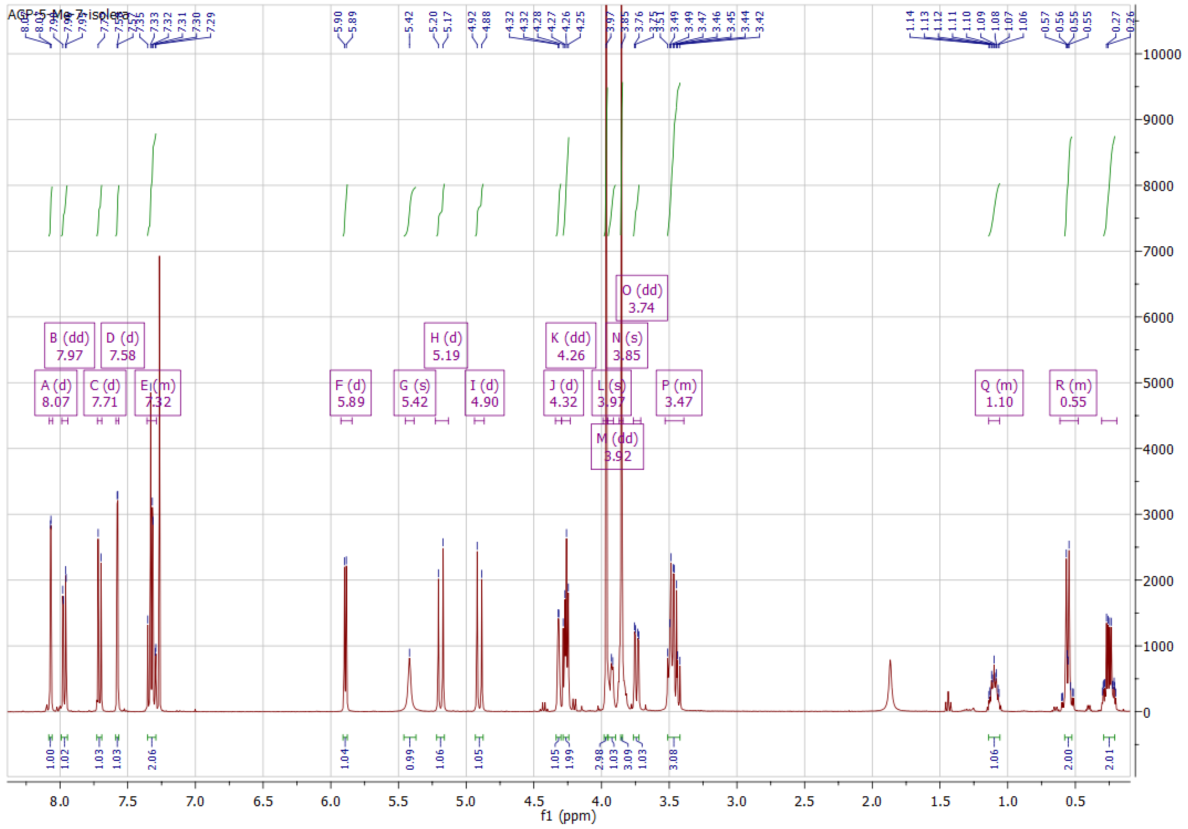

Supplementary Fig. 69. ^{1}H NMR (400 MHz, $CDCl_3$) of **21**.

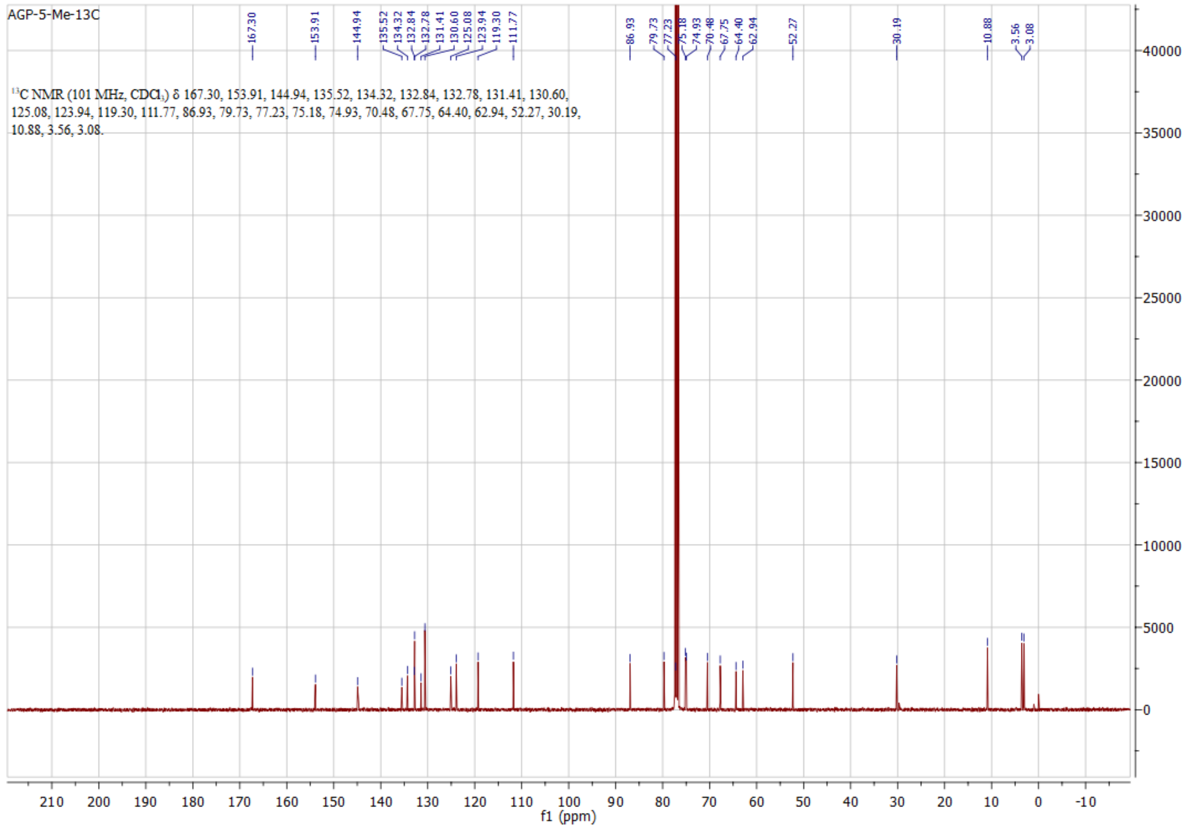

Supplementary Fig. 70. ^{13}C NMR (101 MHz, $CDCl_3$) of **21**.

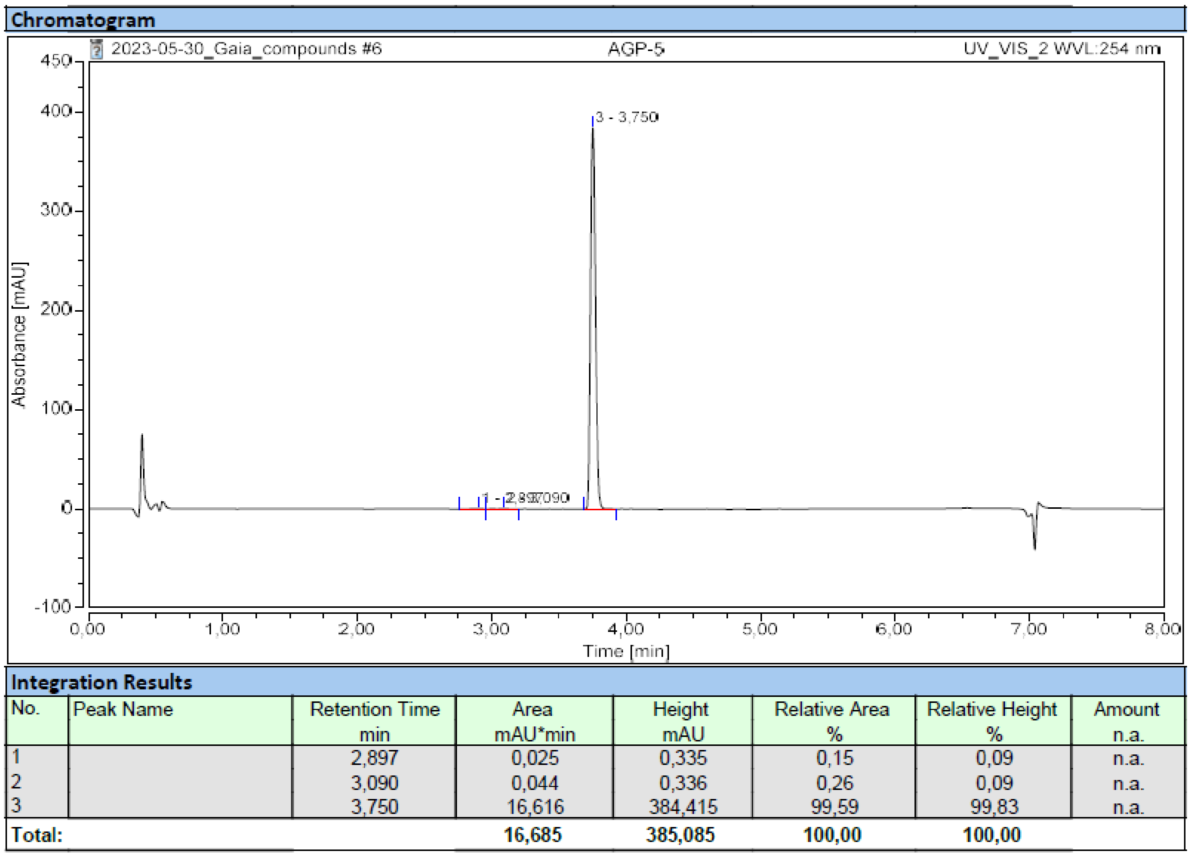

Integration Results

No.	Peak Name	Retention Time min	Area mAU*min	Height mAU	Relative Area %	Relative Height %	Amount n.a.
1		2,897	0,025	0,335	0,15	0,09	n.a.
2		3,090	0,044	0,336	0,26	0,09	n.a.
3		3,750	16,616	384,415	99,59	99,83	n.a.
**Total:**			**16,685**	**385,085**	**100,00**	**100,00**	

***Supplementary Fig. 71**. HPLC chromatogram of **21**.*

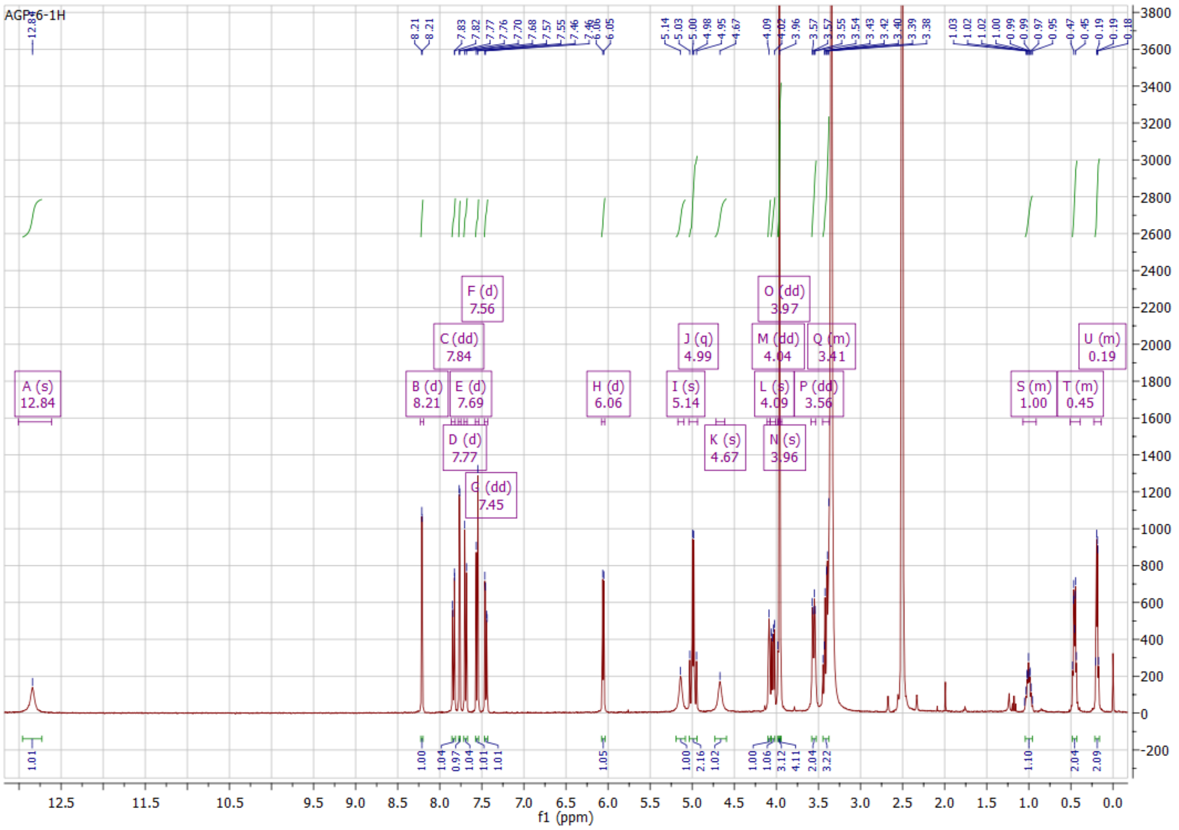

***Supplementary Fig. 72**. ^{1}H NMR (400 MHz, DMSO-d_6) of **22**.*

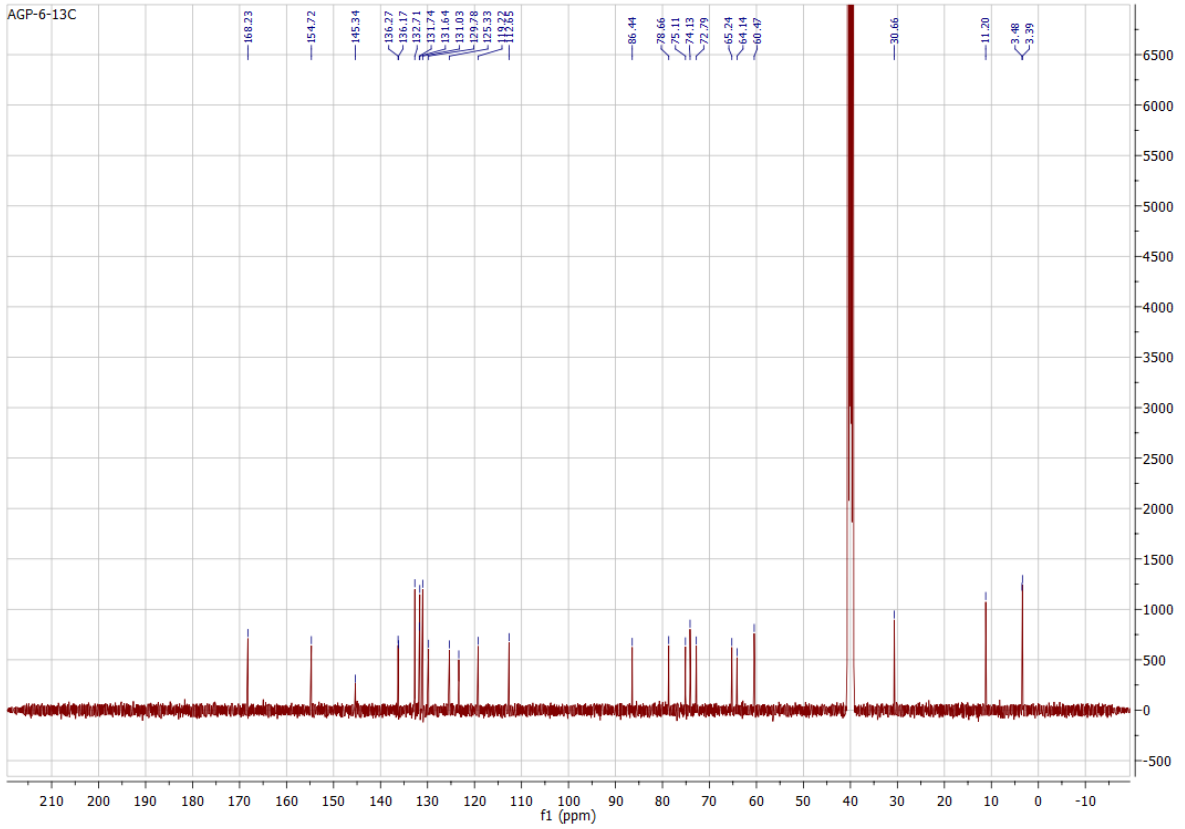

Supplementary Fig. 73. *^{13}C NMR (101 MHz, DMSO-d_6) of **22**.*

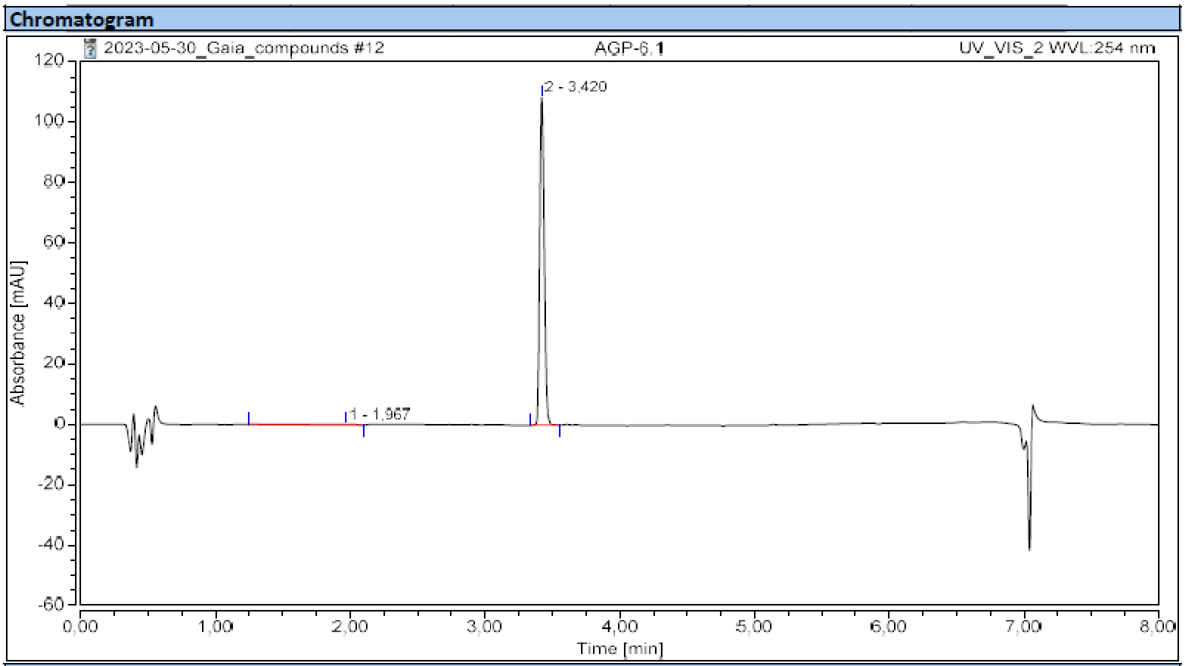

Integration Results

No.	Peak Name	Retention Time min	Area mAU*min	Height mAU	Relative Area %	Relative Height %	Amount n.a.
1		1,967	0,086	0,224	2,01	0,21	n.a.
2		3,420	4,190	108,392	97,99	99,79	n.a.
**Total:**			**4,276**	**108,616**	**100,00**	**100,00**	

Supplementary Fig. 74. *HPLC chromatogram of **22**.*

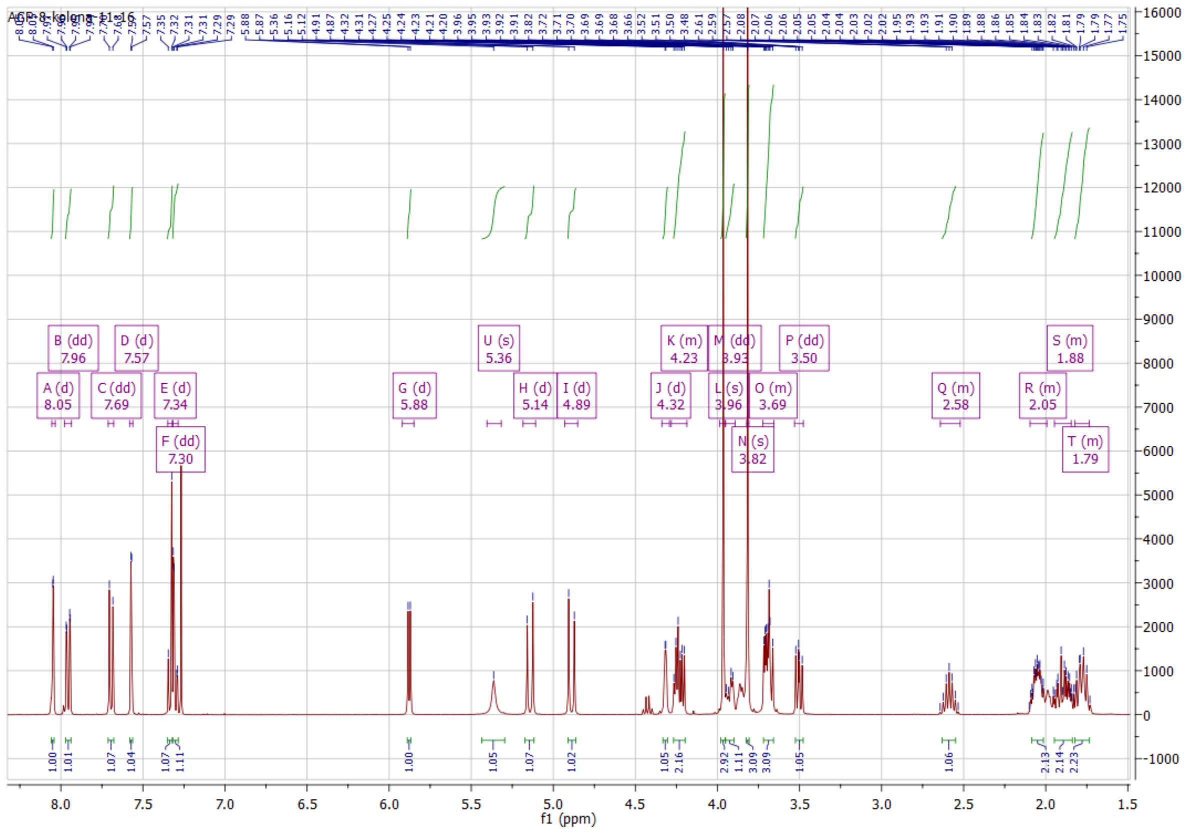

Supplementary Fig. 75. ^{1}H NMR (400 MHz, $CDCl_3$) of ***24****.*

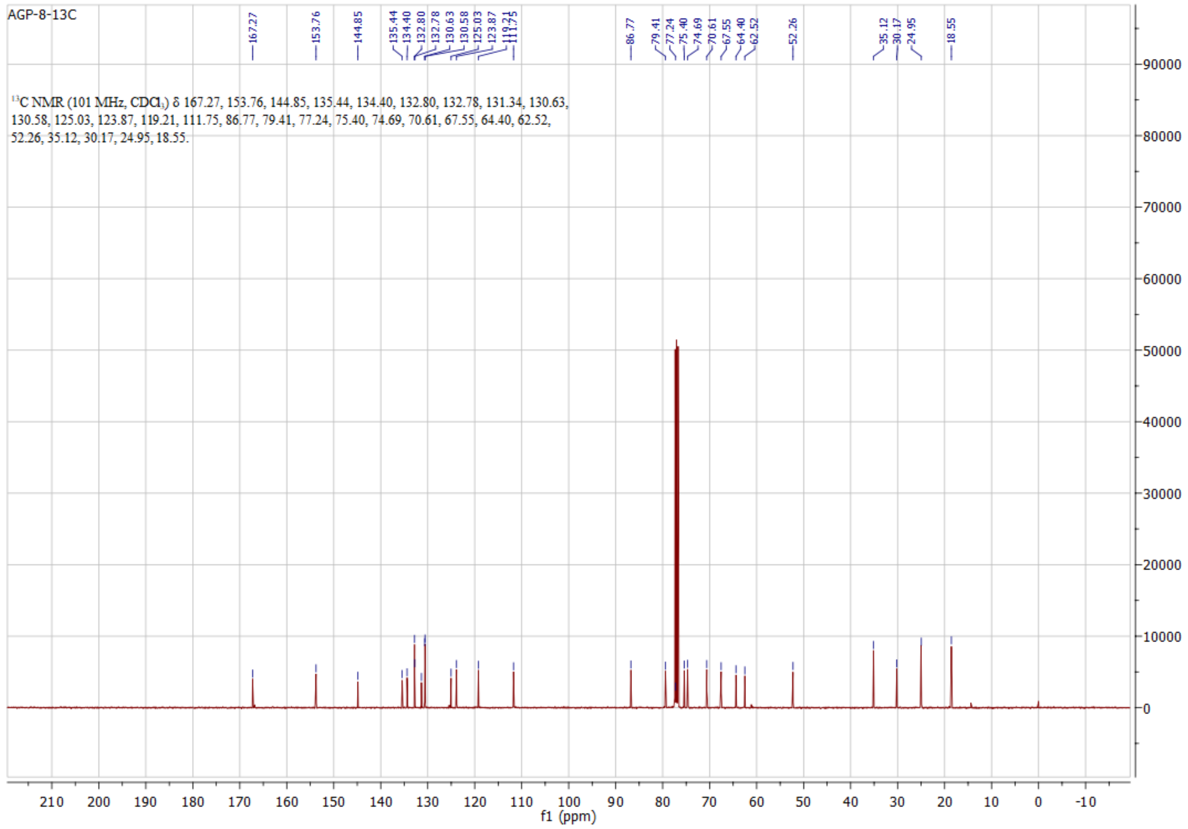

Supplementary Fig. 76. ^{13}C NMR (101 MHz, $CDCl_3$) of ***24****.*

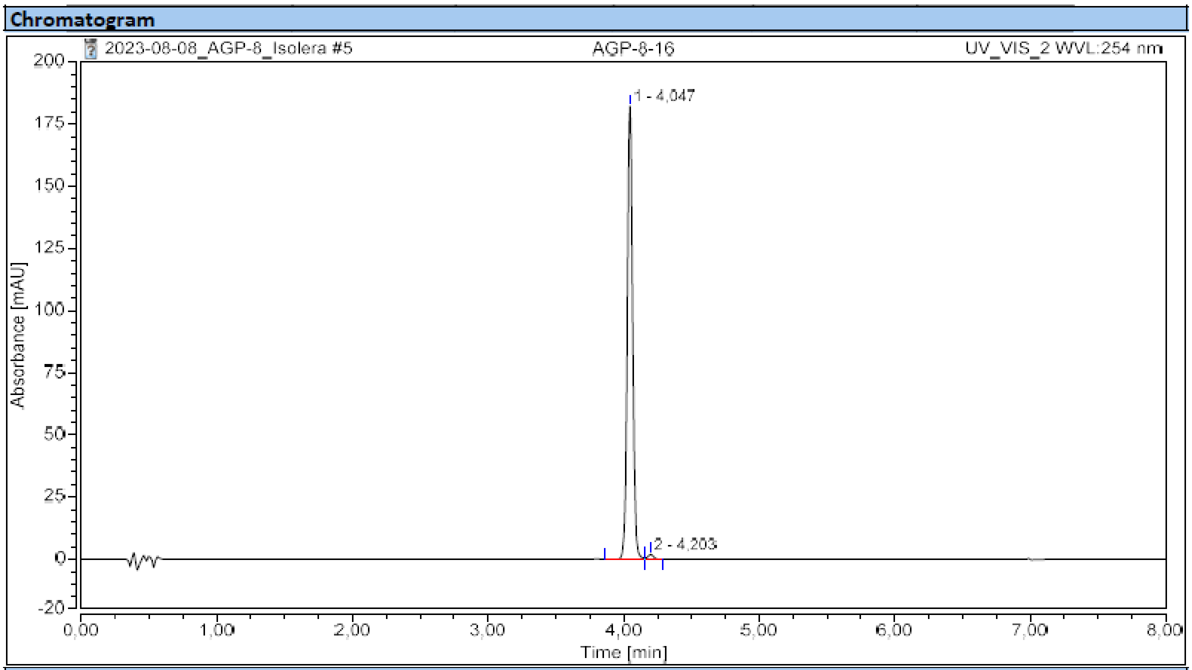

Integration Results

No.	Peak Name	Retention Time min	Area mAU*min	Height mAU	Relative Area %	Relative Height %	Amount n.a.
1		4,047	8,273	182,359	98,84	98,90	n.a.
2		4,203	0,097	2,019	1,16	1,10	n.a.
**Total:**			**8,370**	**184,378**	**100,00**	**100,00**	

Supplementary Fig. 77*. HPLC chromatogram of* ***24****.*

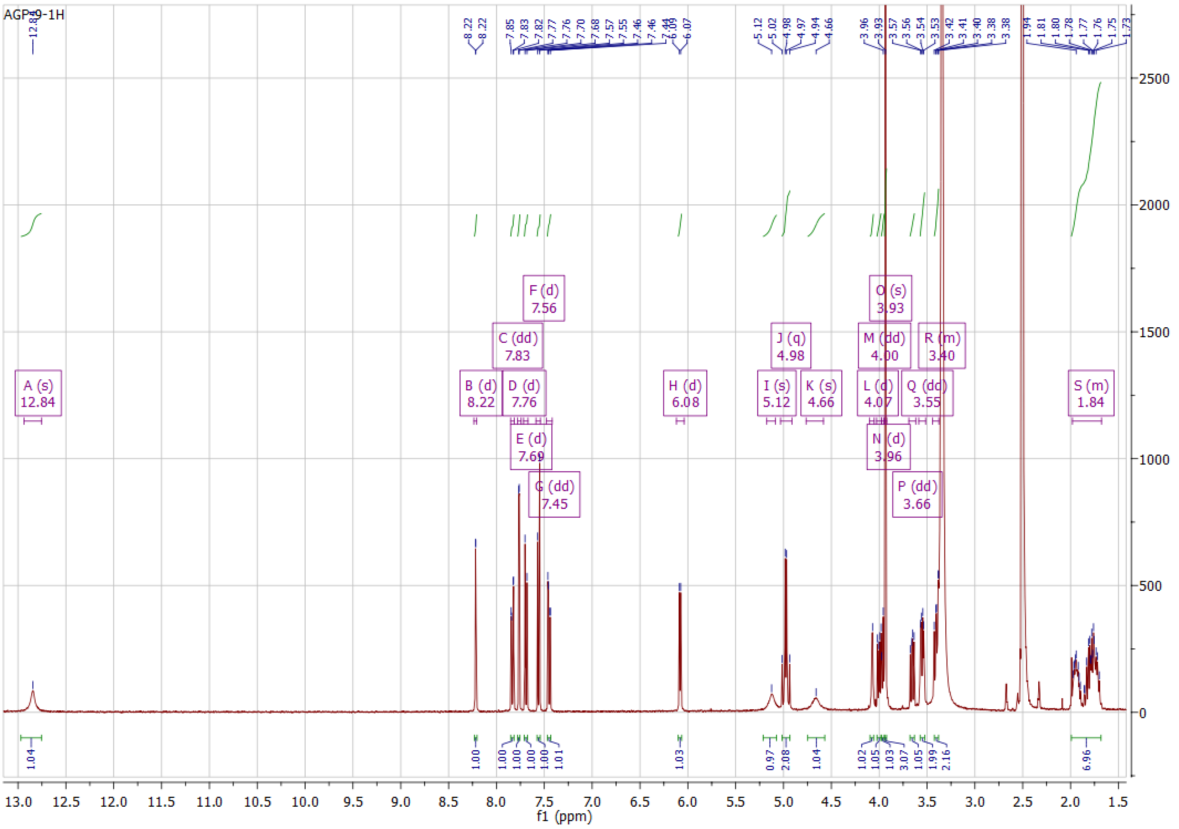

Supplementary Fig. 78*.* *^{1}H NMR (400 MHz, DMSO-d_6) of* ***25****.*

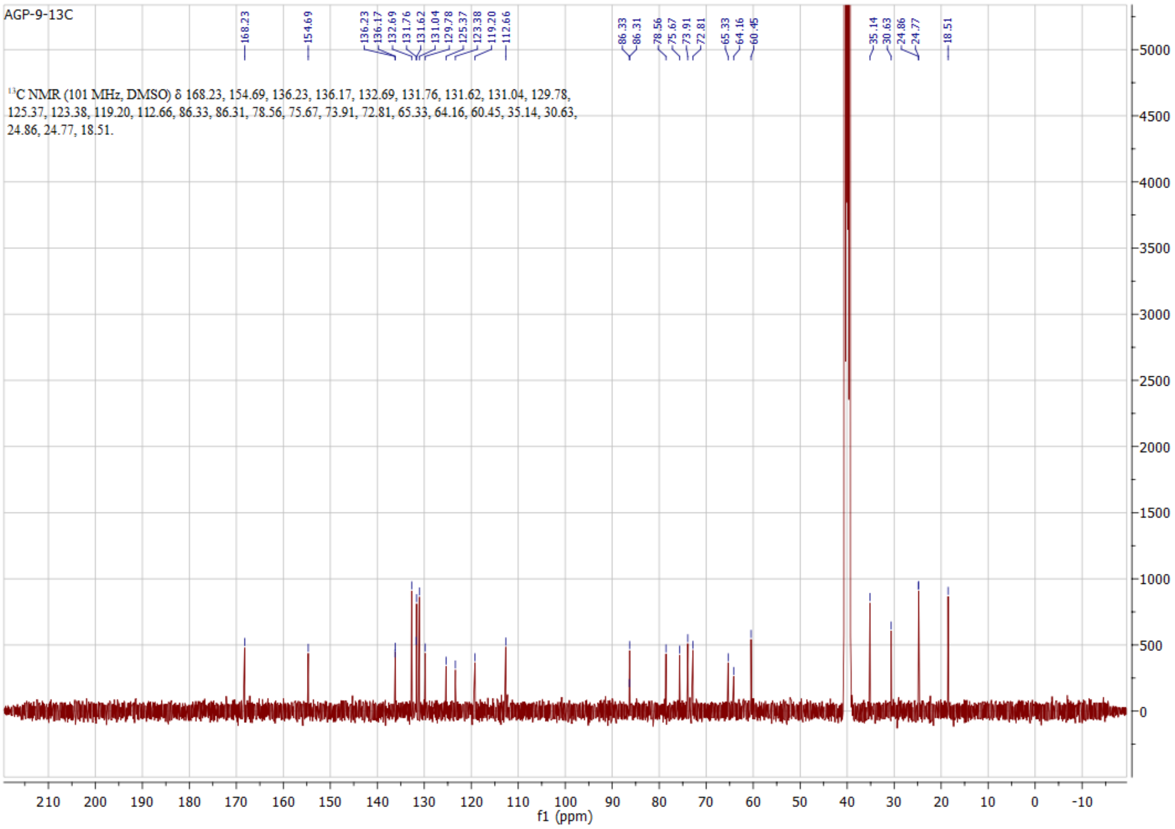

Supplementary Fig. 79. *^{13}C NMR (101 MHz, DMSO-d_6) of **25**.*

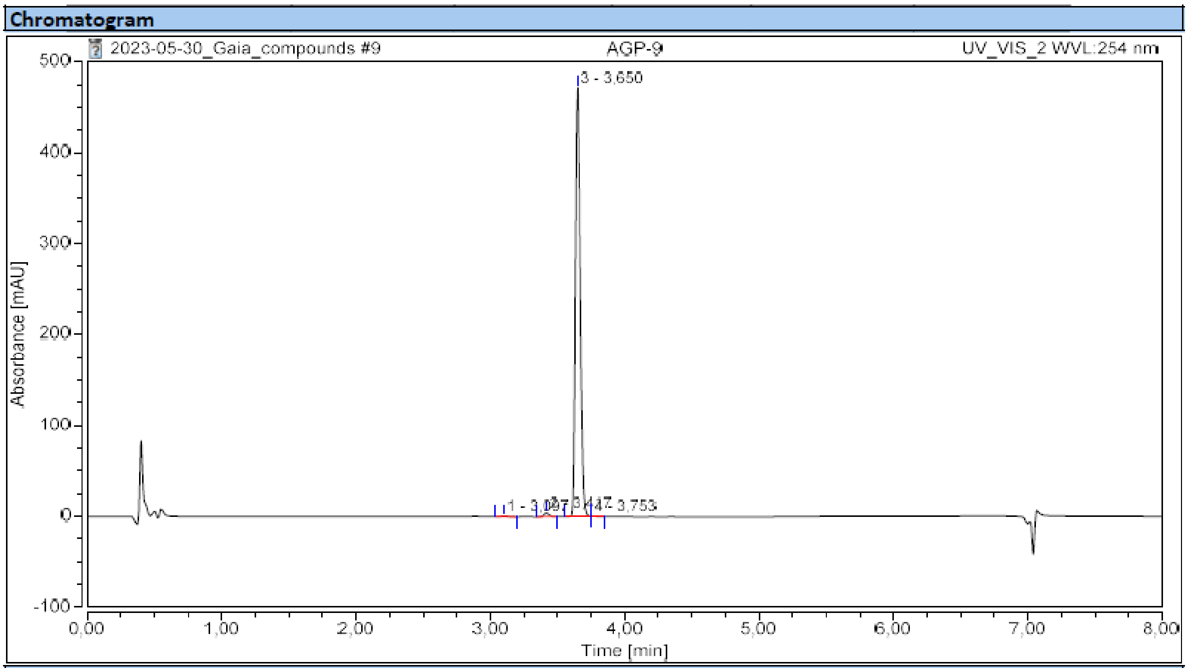

Integration Results

No.	Peak Name	Retention Time<br>min	Area<br>mAU*min	Height<br>mAU	Relative Area<br>%	Relative Height<br>%	Amount<br>n.a.
1		3,097	0,030	0,400	0,14	0,08	n.a.
2		3,417	0,174	3,754	0,83	0,79	n.a.
3		3,650	20,734	471,970	98,87	98,94	n.a.
4		3,753	0,034	0,897	0,16	0,19	n.a.
**Total:**			**20,972**	**477,021**	**100,00**	**100,00**	

Supplementary Fig. 80. *HPLC chromatogram of **25**.*

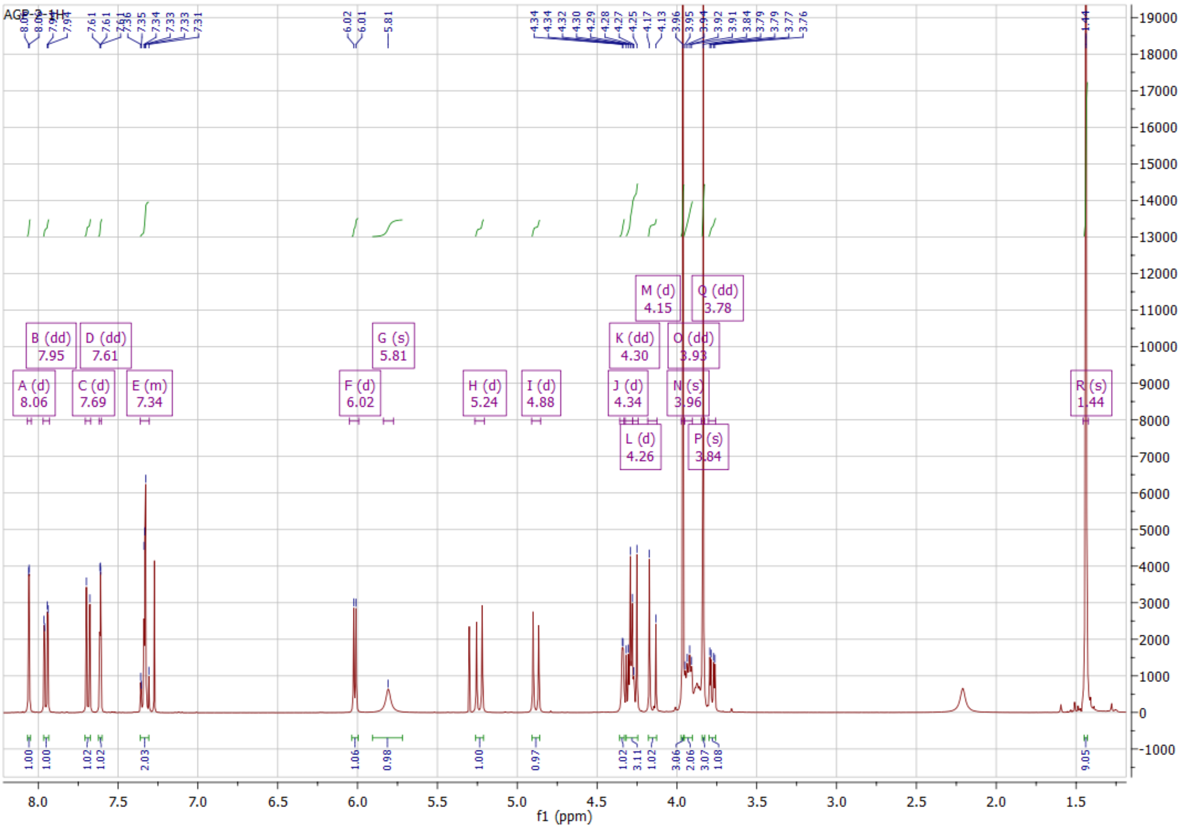

***Supplementary Fig. 81**. 1H NMR (400 MHz, $CDCl_3$) of **27**.*

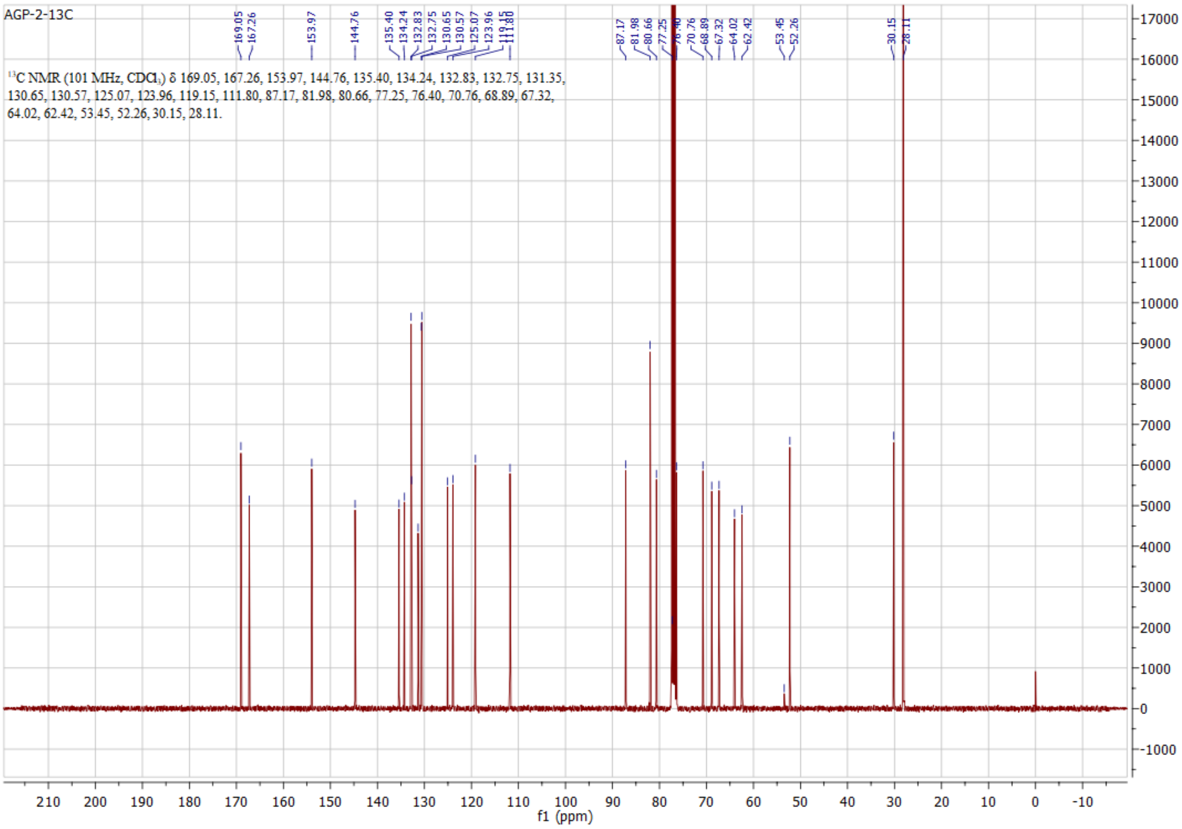

***Supplementary Fig. 82**. ^{13}C NMR (101 MHz, $CDCl_3$) of **27**.*

Chromatogram

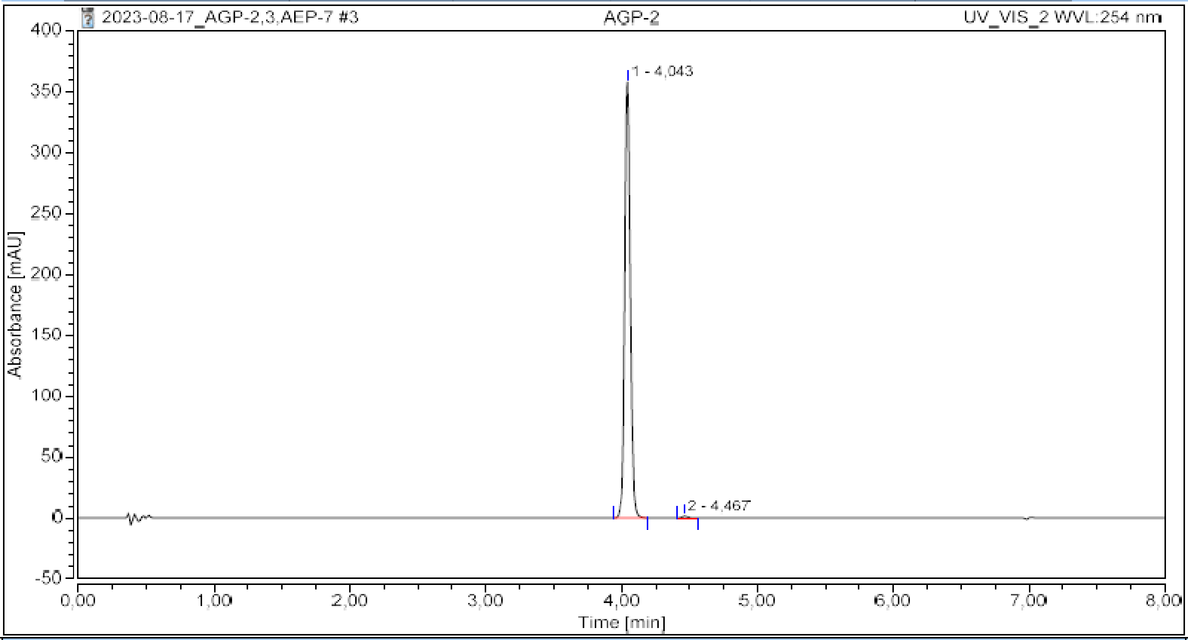

Integration Results

No.	Peak Name	Retention Time min	Area mAU*min	Height mAU	Relative Area %	Relative Height %	Amount n.a.
1		4,043	17,141	358,095	99,60	99,60	n.a.
2		4,467	0,068	1,451	0,40	0,40	n.a.
**Total:**			**17,210**	**359,545**	**100,00**	**100,00**	

Supplementary Fig. 83. *HPLC chromatogram of **27**.*

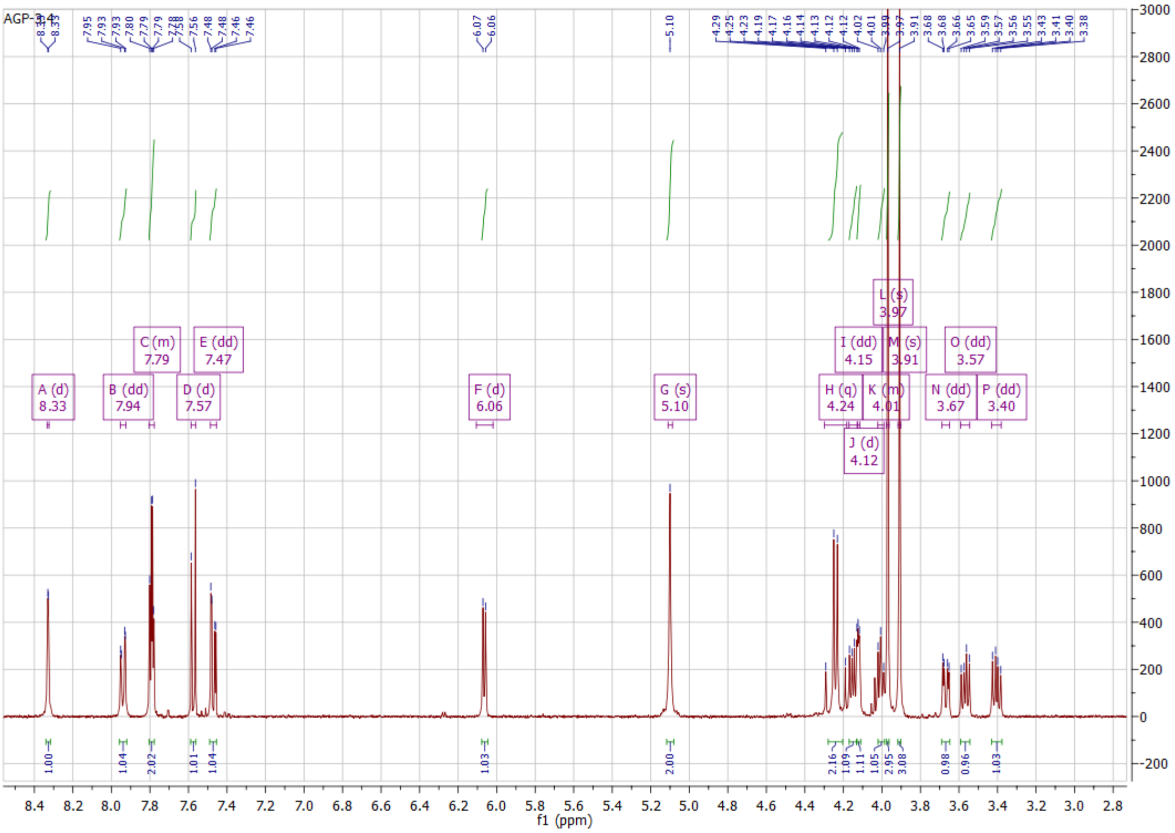

Supplementary Fig. 84. *^{1}H NMR (400 MHz, DMSO-d_6) of **28**.*

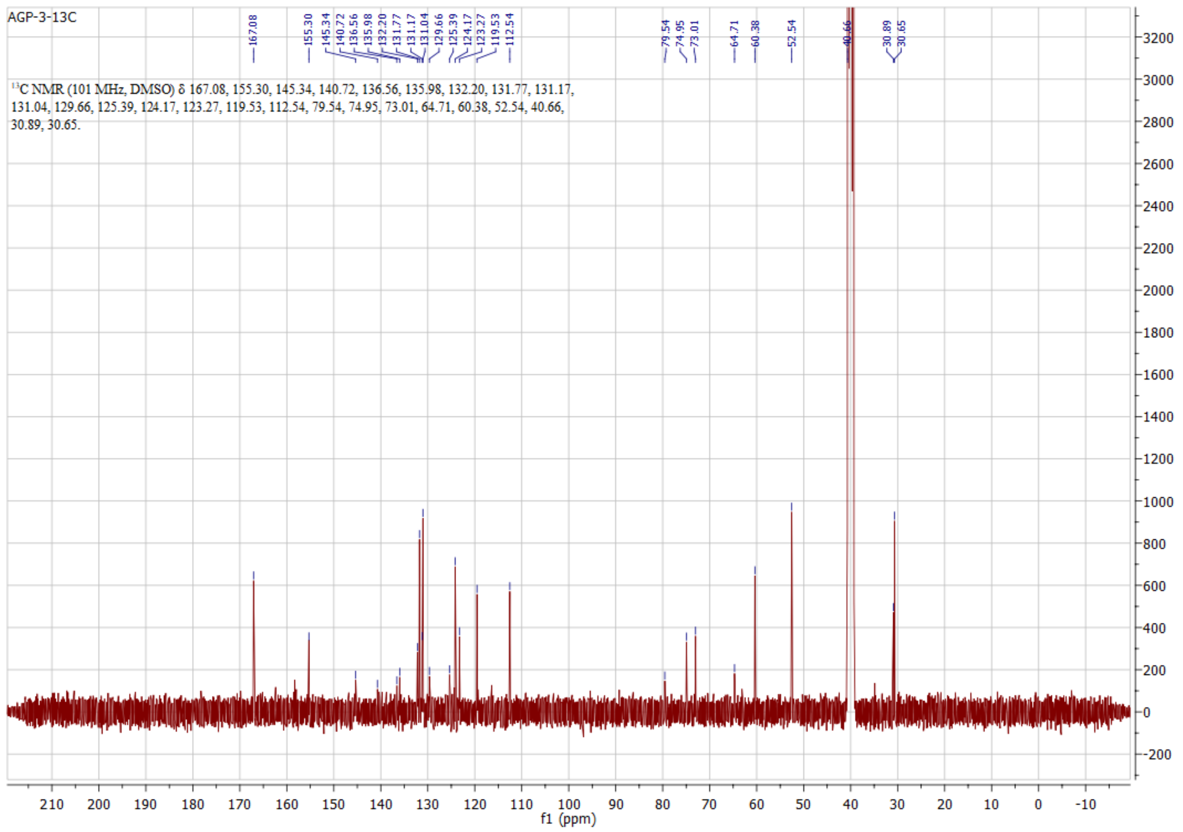

Supplementary Fig. 85. *^{13}C NMR (101 MHz, DMSO-d_6) of **28**.*

Chromatogram

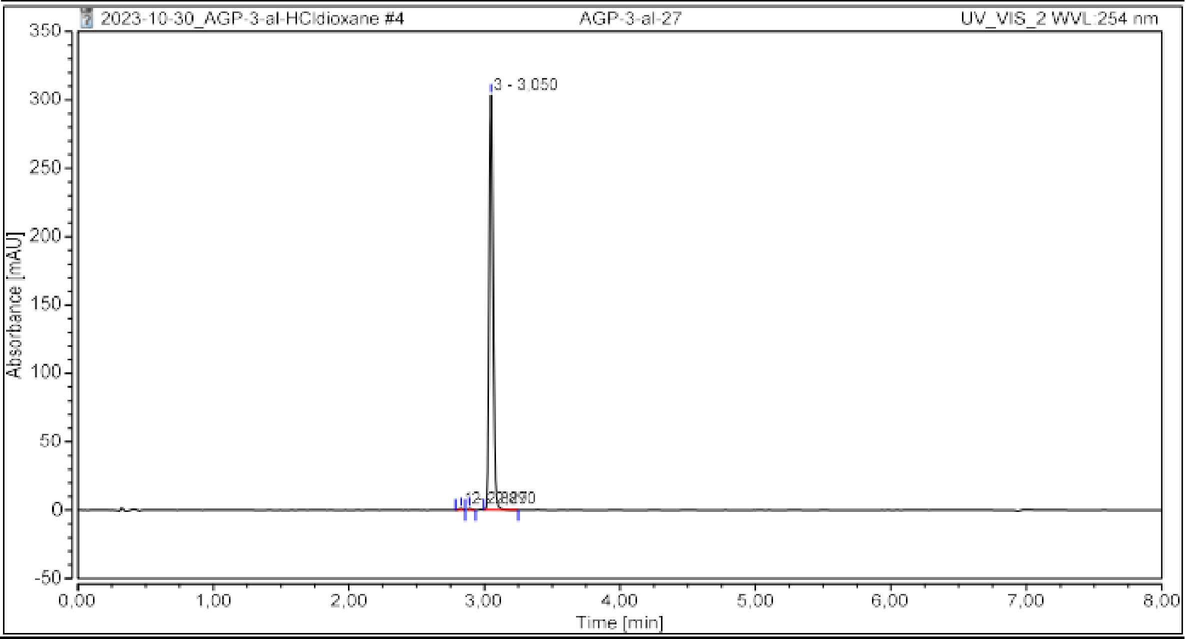

Integration Results

No.	Peak Name	Retention Time min	Area mAU*min	Height mAU	Relative Area %	Relative Height %	Amount n.a.
1		2,827	0,040	1,259	0,39	0,41	n.a.
2		2,890	0,039	1,191	0,38	0,39	n.a.
3		3,050	10,205	303,344	99,23	99,20	n.a.
**Total:**			**10,285**	**305,794**	**100,00**	**100,00**	

Supplementary Fig. 86. HPLC chromatogram of **28**.

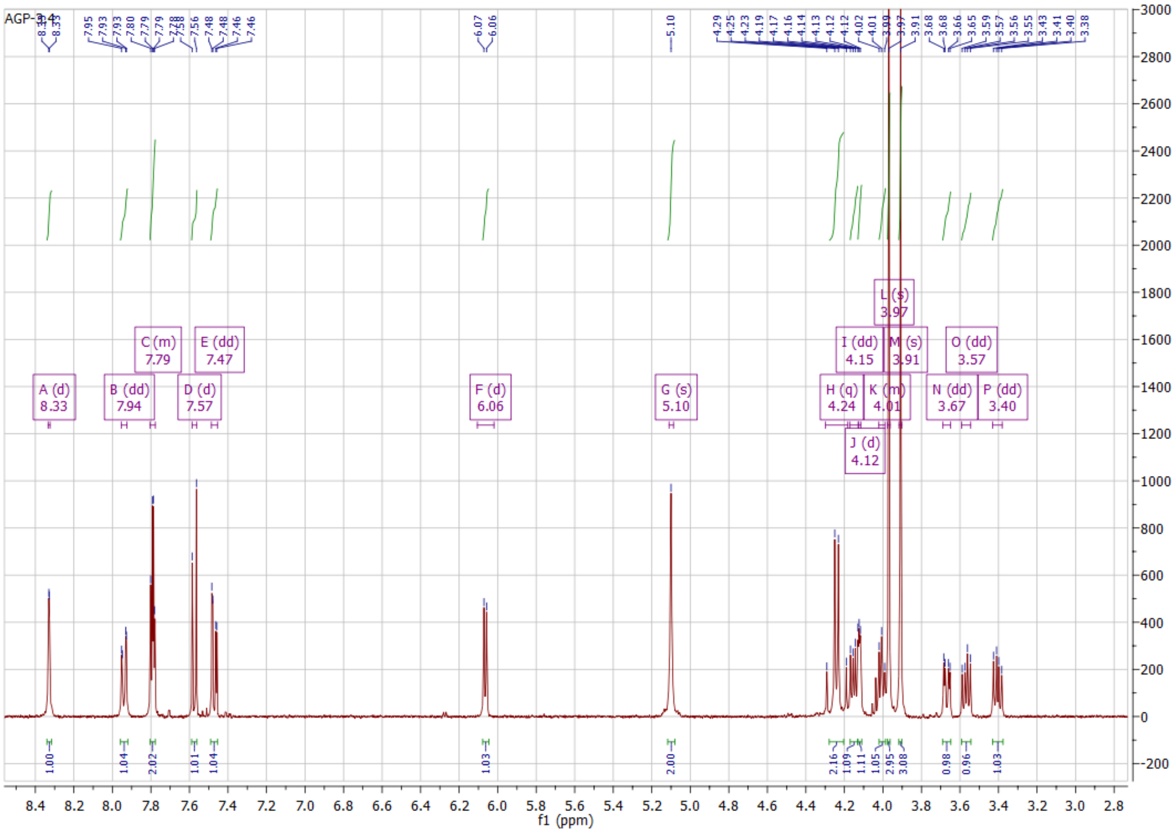

Supplementary Fig. 87. ^{1}H NMR (400 MHz, D_2O) of **29**.

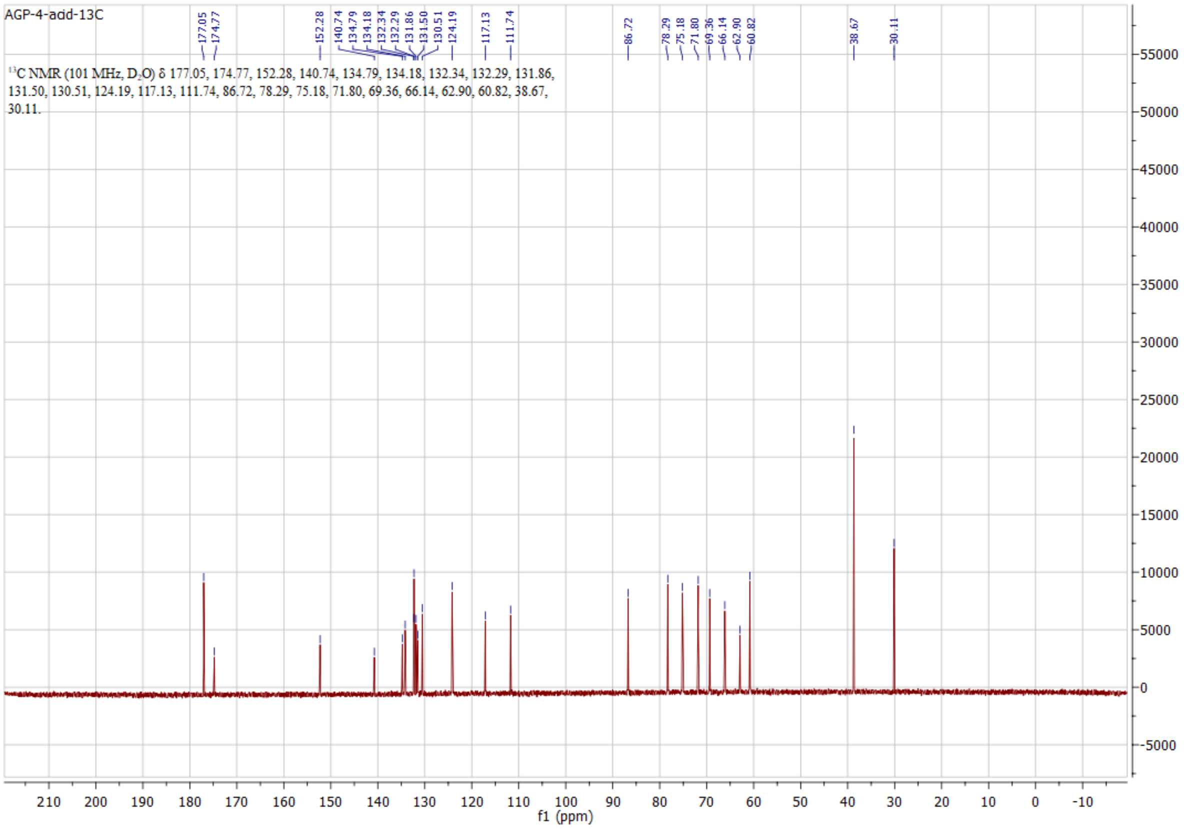

Supplementary Fig. 88. *^{13}C NMR (101 MHz, D_2O) of* ***29****.*

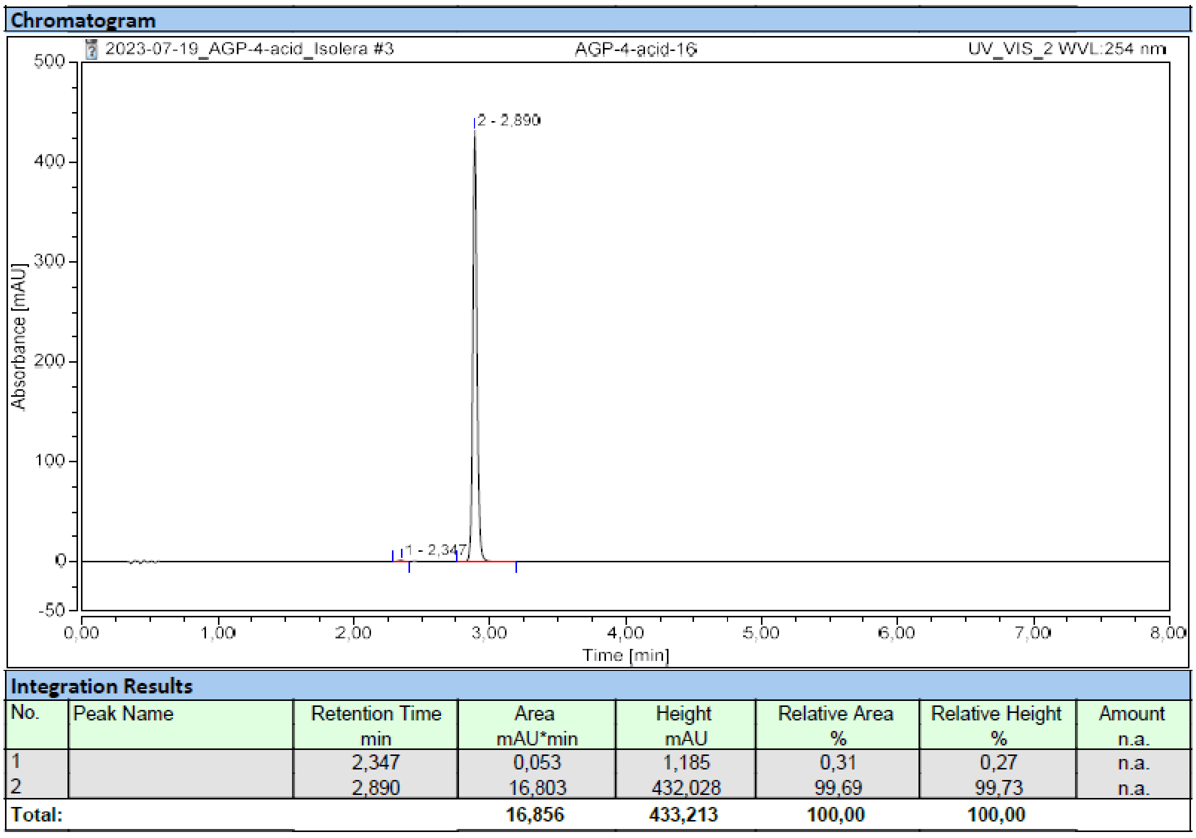

Integration Results

No.	Peak Name	Retention Time min	Area mAU*min	Height mAU	Relative Area %	Relative Height %	Amount n.a.
1		2,347	0,053	1,185	0,31	0,27	n.a.
2		2,890	16,803	432,028	99,69	99,73	n.a.
**Total:**			**16,856**	**433,213**	**100,00**	**100,00**	

Supplementary Fig. 89. *HPLC-UV chromatogram of* ***29****.*

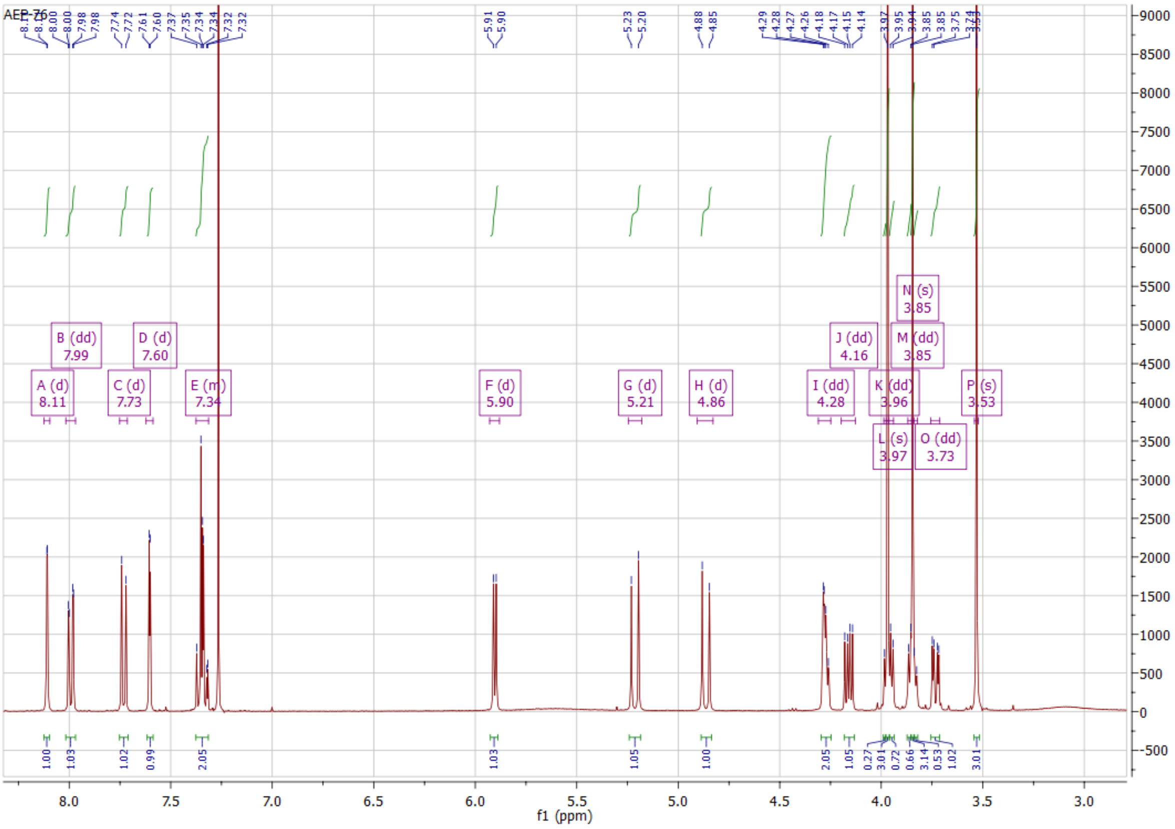

Supplementary Fig. 90*.* [1]*H NMR (400 MHz, CDCl*$_3$*) of* ***31****.*

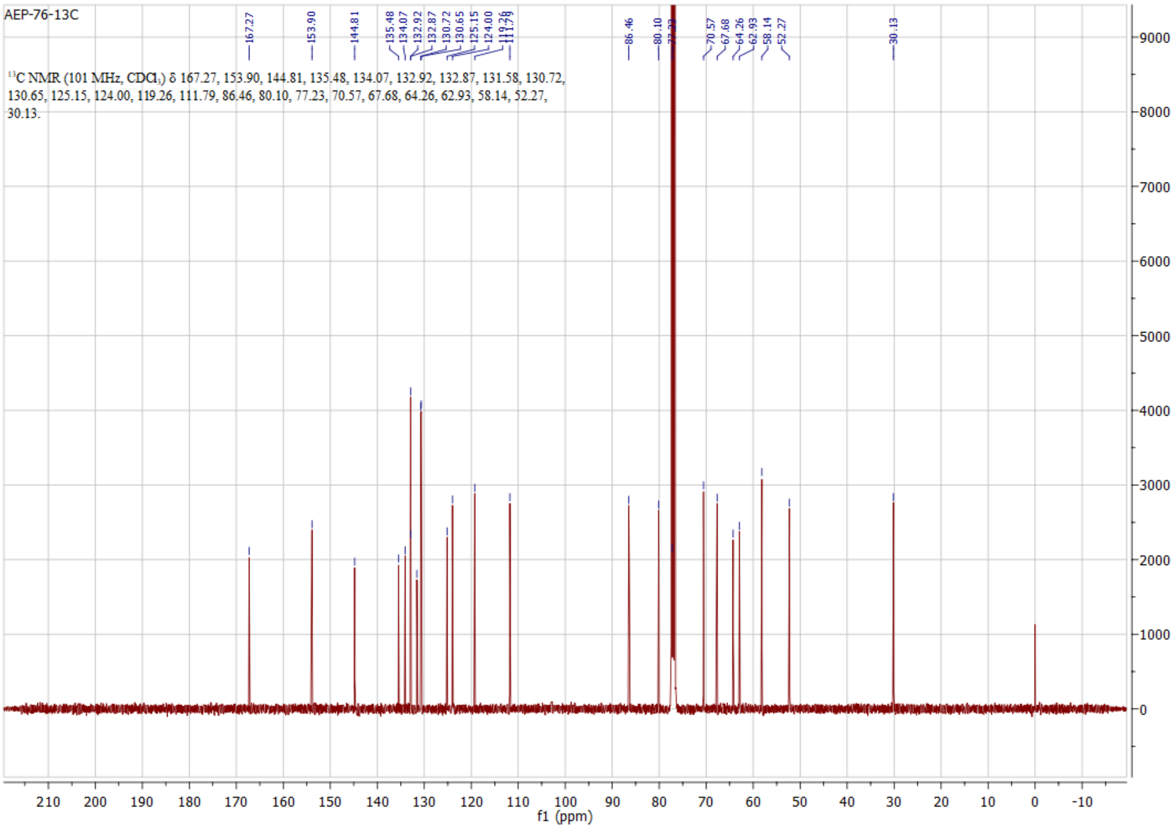

Supplementary Fig. 91*.* [13]*C NMR (101 MHz, CDCl*$_3$*) of* ***31****.*

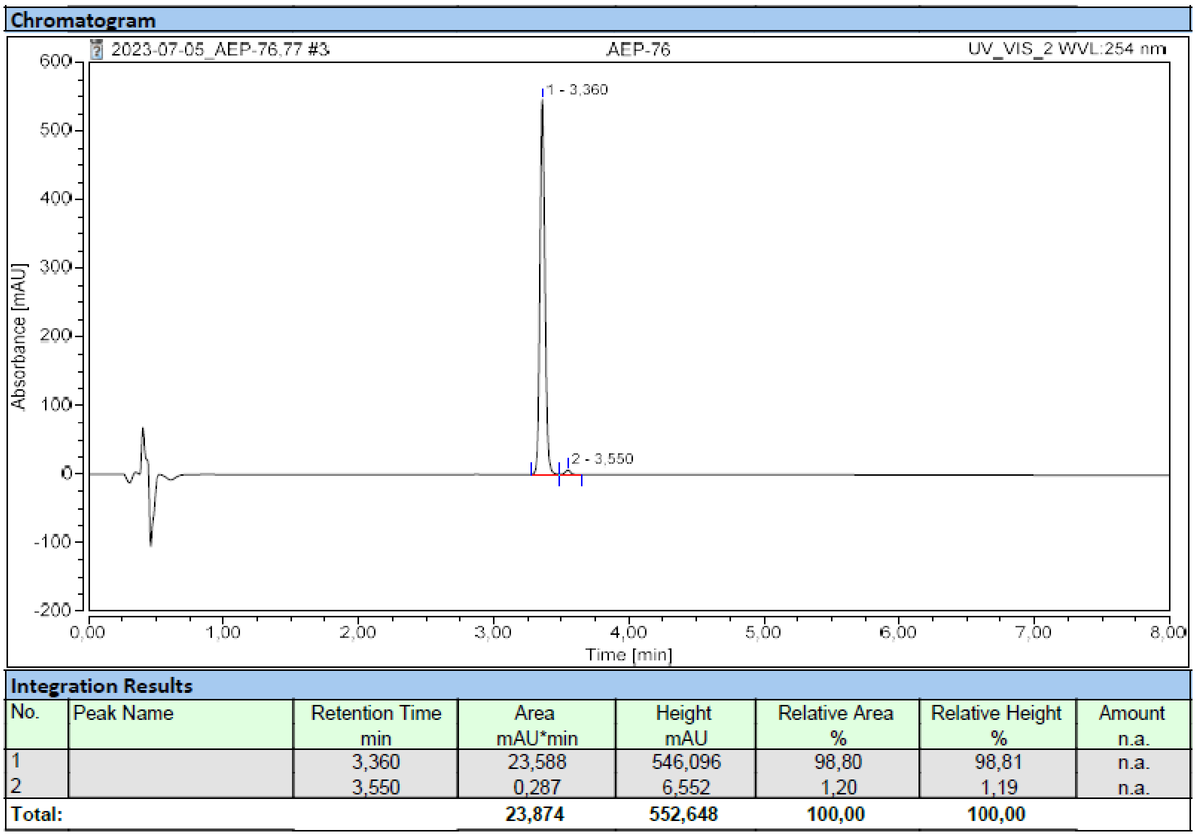

Integration Results

No.	Peak Name	Retention Time min	Area mAU*min	Height mAU	Relative Area %	Relative Height %	Amount n.a.
1		3,360	23,588	546,096	98,80	98,81	n.a.
2		3,550	0,287	6,552	1,20	1,19	n.a.
**Total:**			**23,874**	**552,648**	**100,00**	**100,00**	

Supplementary Fig. 92. *HPLC chromatogram of **31**.*

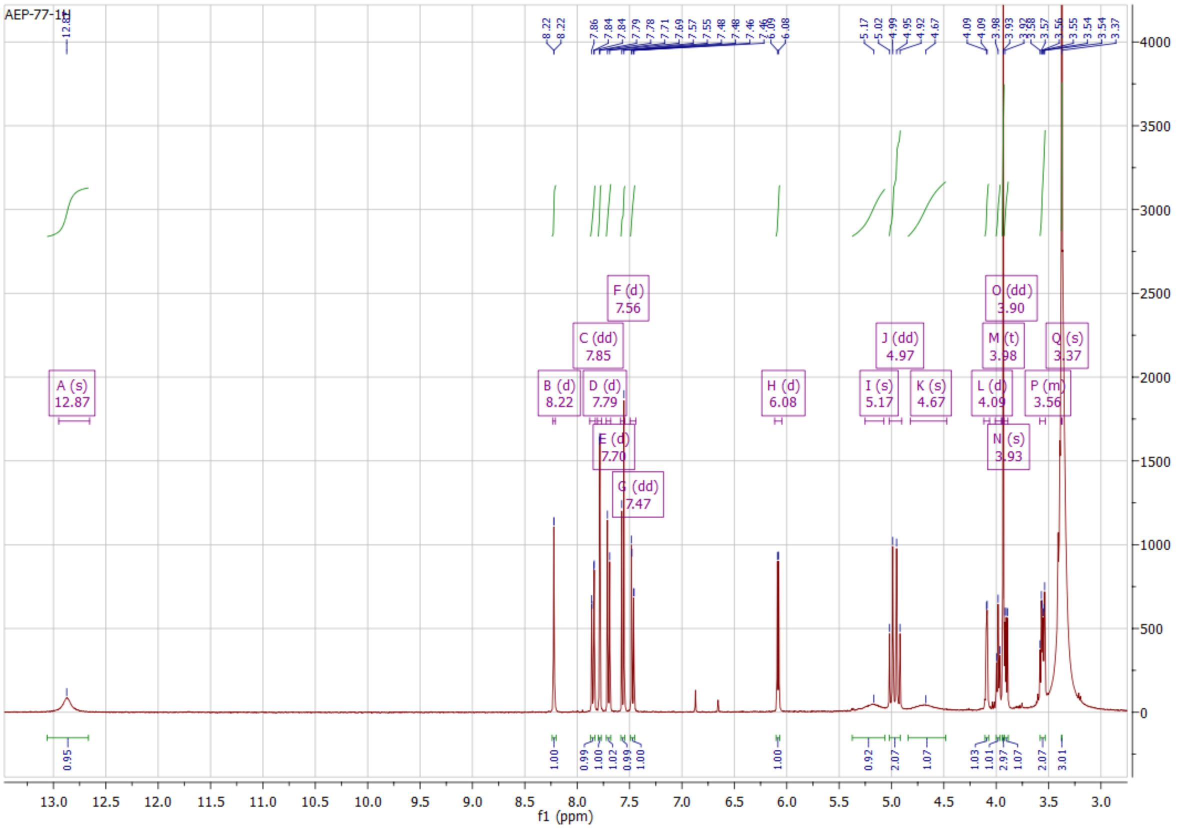

Supplementary Fig. 93. *^{1}H NMR (400 MHz, DMSO-d_6) of **32**.*

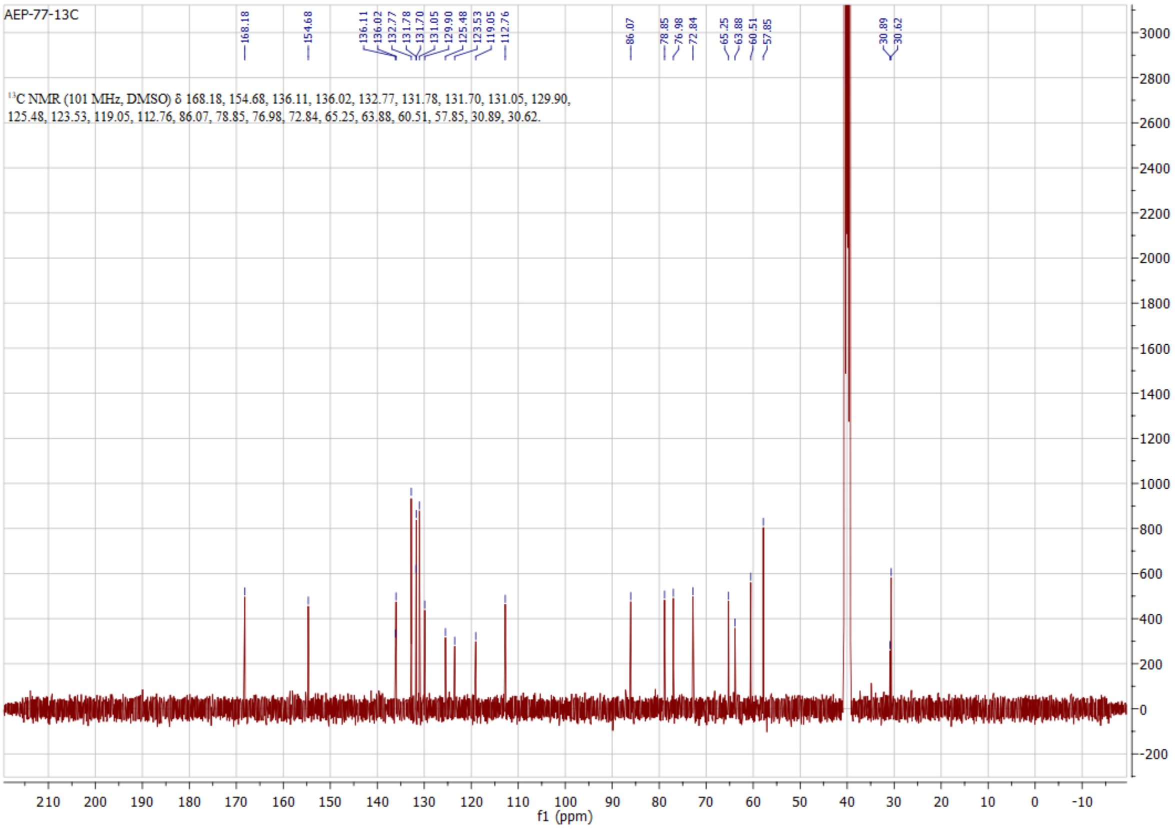

Supplementary Fig. 94. ^{13}C NMR (101 MHz, DMSO-d_6) of **32**.

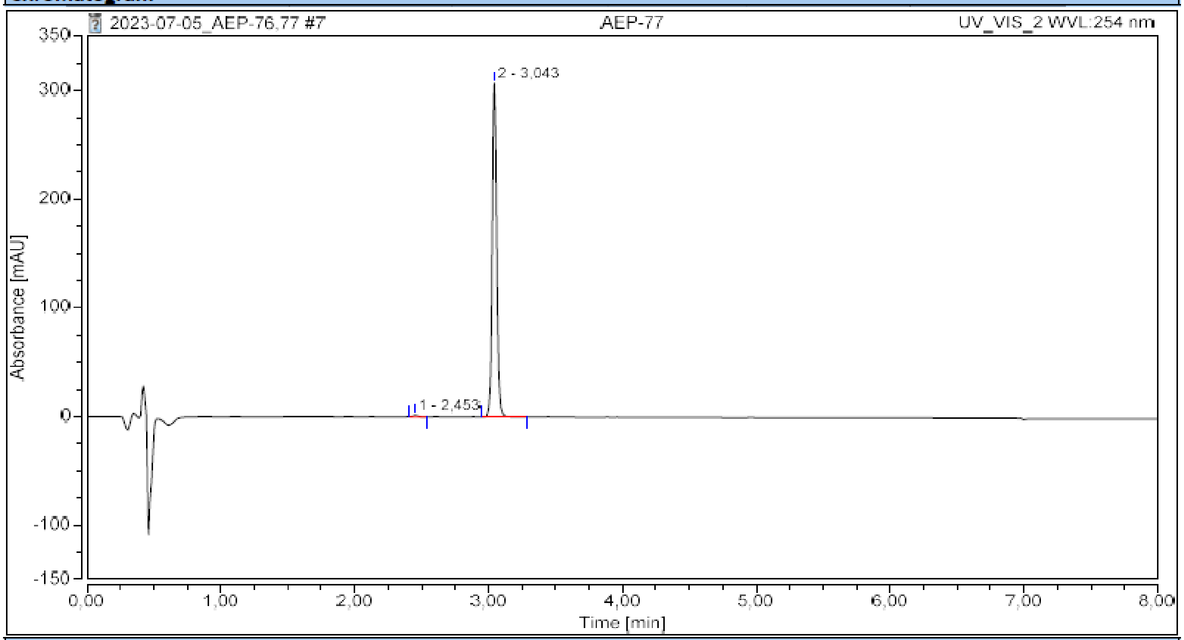

Integration Results

No.	Peak Name	Retention Time min	Area mAU*min	Height mAU	Relative Area %	Relative Height %	Amount n.a.
1		2,453	0,053	1,337	0,46	0,43	n.a.
2		3,043	11,582	306,945	99,54	99,57	n.a.
**Total:**			**11,635**	**308,282**	**100,00**	**100,00**	

Supplementary Fig. 95. HPLC-UV chromatogram of **32**.

Protein purification

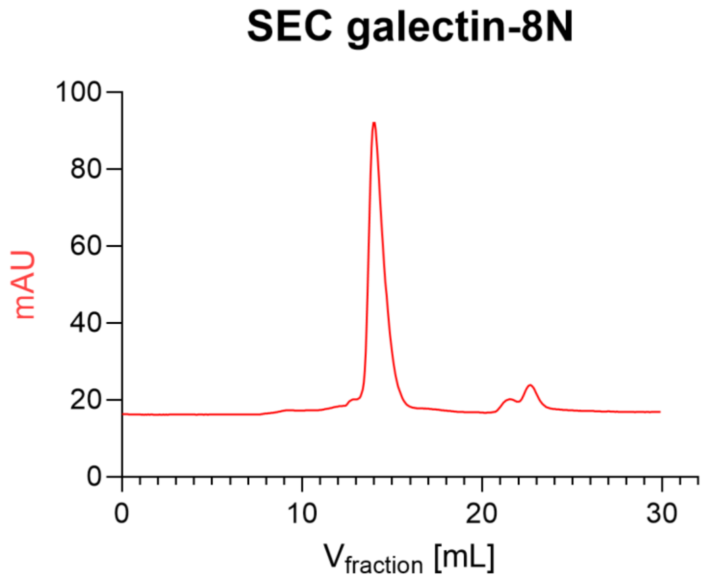

Supplementary Fig. 96. *UV spectra (254 nm) after Size-Exclusion Chromatography (SEC-FPLC).*

X-ray crystallography data

Supplementary table 1. *Data collection and refinement statistics.*

	overall	outer shell
**space group**	$P2_12_12_1$	
**unit cell dimensions (Å)**	a = 53.7, b = 62.3, c = 84.8	
**Data quality**		
**resolution limits (Å)**	45.4-1.08	1.12-1.08
**R_{merge} (I)**	0.054	1.465
**R_{meas} (I)**	0.056	1.573
**R_{pim} (I)**	0.015	0.554
**CC(1/2)**	0.999	0.559
**total number of observations**	1,480,031	75,497
**total number unique**	119,679	9,860
**completeness (%)**	98.0	83.8
**multiplicity**	12.4	7.7
**mean (I)/σ(I)**	18.4	1.2
**Refinement statistics**		
**resolution limits (Å)**	42.4-1.08	1.09-1.08
**No. of atoms protein/water/ligand**	2444 / 379 / 75	
**Mean B-factors protein/water/ligand (Å²)**	18.8 / 33.7 / 20.2	
**R_{model} (F)**	0.1327	0.289
**R_{free} (F)**	0.1506	0.294
**R_{free} test set size (%)**	5.0	
**R_{free} test set count**	5,966	160
**rms deviations from ideal geometry**		
**bonds**	0.009 Å	
**angles**	1.1 °	
**Ramachandran plot**		
**Favoured / allowed / outliers (%)**	98.6 / 1.4 / 0.0	
MolProbity clash score	1.41	

ITC data

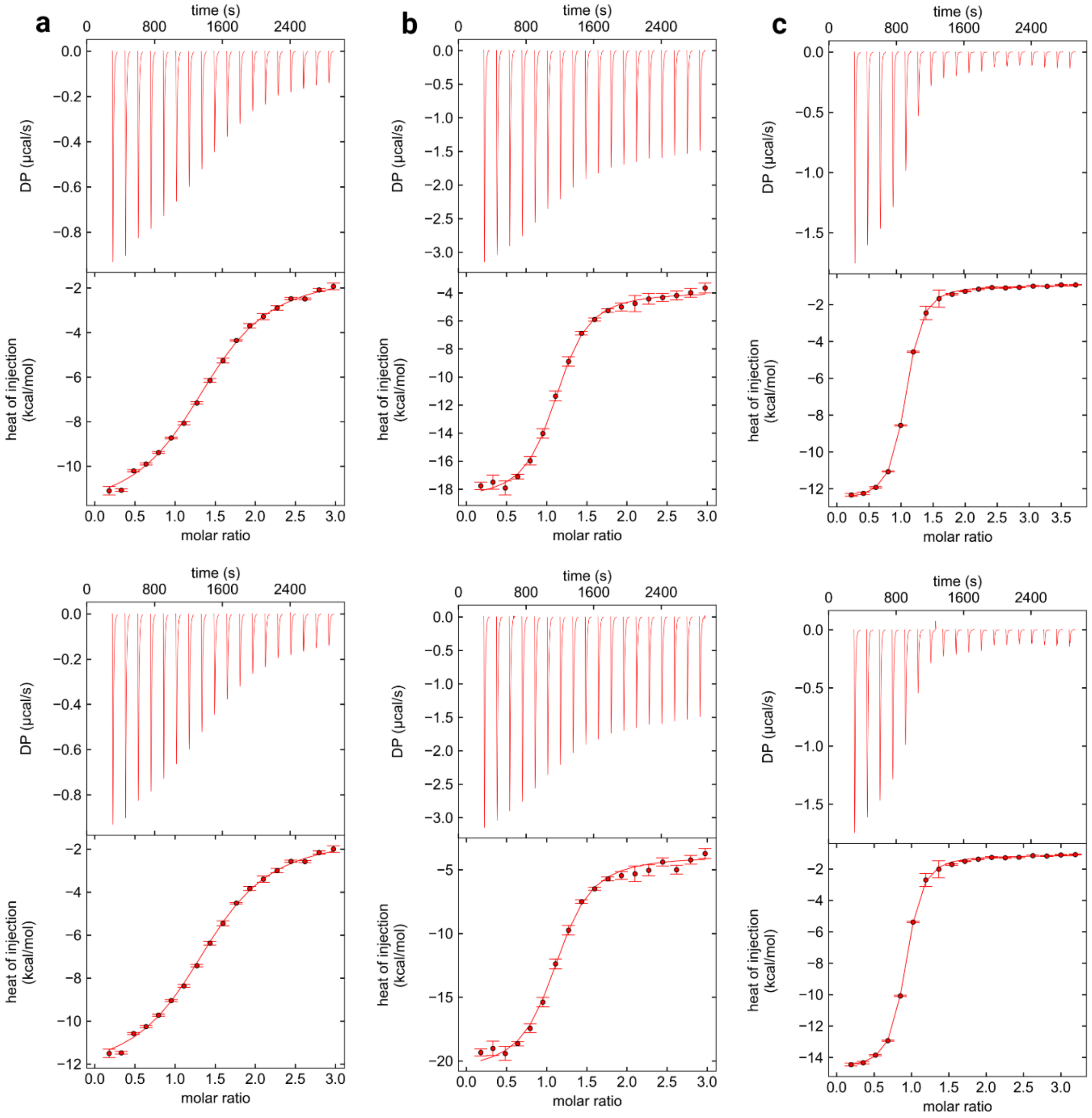

***Supplementary Fig. 97**. Titration curves and thermograms obtained by injections of **1** (**a**), **11** (**b**) and **29** (**c**) into galectin-8N solution. Number of replicate titrations: n=2.*

***Supplementary table 2**. Thermodynamic data of selected galectin-8N inhibitors, determined by ITC.*

Compound	ΔG [kJ/mol]	ΔH [kJ/mol]	-TΔS [kJ/mol]	K_d [µM]
**1**	-30.2 ± 0.6	-44 ± 12	14 ± 13	5 ± 1
**11**	-33.5 ± 0.5	-61 ± 8	28 ± 9	1.3 ± 0.3
**29**	-35.3 ± 0.4	-57 ± 3	22 ± 3	0.6 ± 0.1

Supplementary table 3. *Comparison of standard thermodynamic parameters of **1** in PBS and TRIS buffer.*

**Buffer**	K_d [µM]	**ΔH** [kJ/mol]	**-TΔS** [kJ/mol]	**ΔG** [kJ/mol]
TRIS	4.5	-50	19	-30.7
PBS	5	-44	14	-30.2

Competitive fluorescence polarisation assay data

Supplementary table 4. *K_d values (µM)[a,b] of ester compounds.*

**Compound**	**K_d (Galectin-3)**	**K_d (Galectin-8N)**
**7a**	n. s.	n. s.
**7b**	n. s.	n. s.
**7c**	n. s.	n. s.
**7d**	n. s.	n. s.
**7e**	n. b.	n. b.
**7f**	n. b.	n. b.
**10**	n. s.	275 ± 5
**18**	n. s.	n. s.
**21**	n. s.	n. b.
**24**	n. s.	n. s.
**27**	n. s.	201.7 ± 1.3
**28***	8.5 ± 1.5	7.7 ± 0.3
**31**	108.6 ± 31.0	n. s.

**K_d (galectin-1) = 44.7 ± 4.9*

[a]*n.s. = non-soluble at tested concentration*

[b]*n.b. = non-binding up to the highest tested concentration of 1500 µM.*

Ligand efficiency (LE) and ligand-lipophilicity efficiency (LLE) calculations

***Supplementary table 5**. clogP, LE and LLE calculations for **8a-f**, **11**, **15a-c**, **16a-c**, **19**, **22**, **25**, **29** and **32**.*

Compound	HA[a]	clogP	K_d [M]	LE [(kcal/mol)/heavy atom]	LLE
**8a**	41	5.44	$1.49*10^{-6}$	0.195	0.382
**8b**	42	5.94	$2.06*10^{-6}$	0.185	-0.257
**8c**	42	5.94	$2.07*10^{-6}$	0.185	-0.259
**8d**	42	5.59	$1.62*10^{-6}$	0.189	0.203
**8e**	43	6.75	$4.31*10^{-6}$	0.171	-1.38
**8f**	42	6.16	$1.84*10^{-6}$	0.187	-0.422
**11**	37	4.09	$0.800*10^{-6}$	0.226	2.01
**15a**	50	5.27	$3.50*10^{-6}$	0.149	0.182
**15b**	50	5.27	$4.51*10^{-6}$	0.146	0.0722
**15c**	50	5.27	$4.14*10^{-6}$	0.147	0.109
**16a**	49	5.07	$0.460*10^{-6}$	0.177	1.26
**16b**	49	5.07	$0.650*10^{-6}$	0.173	1.11
**16c**	49	5.07	$1.09*10^{-6}$	0.167	0.888
**19**	41	6.32	$4.05*10^{-6}$	0.180	-0.926
**22**	38	4.64	$1.02*10^{-6}$	0.216	1.35
**25**	39	5.20	$1.61*10^{-6}$	0.204	0.593
**29**	38	3.47	$0.500*10^{-6}$	0.227	2.84
**32**	35	3.81	$1.28*10^{-6}$	0.231	2.08

[a] *HA = number of non-hydrogen atoms in the structure.*

MD simulation data

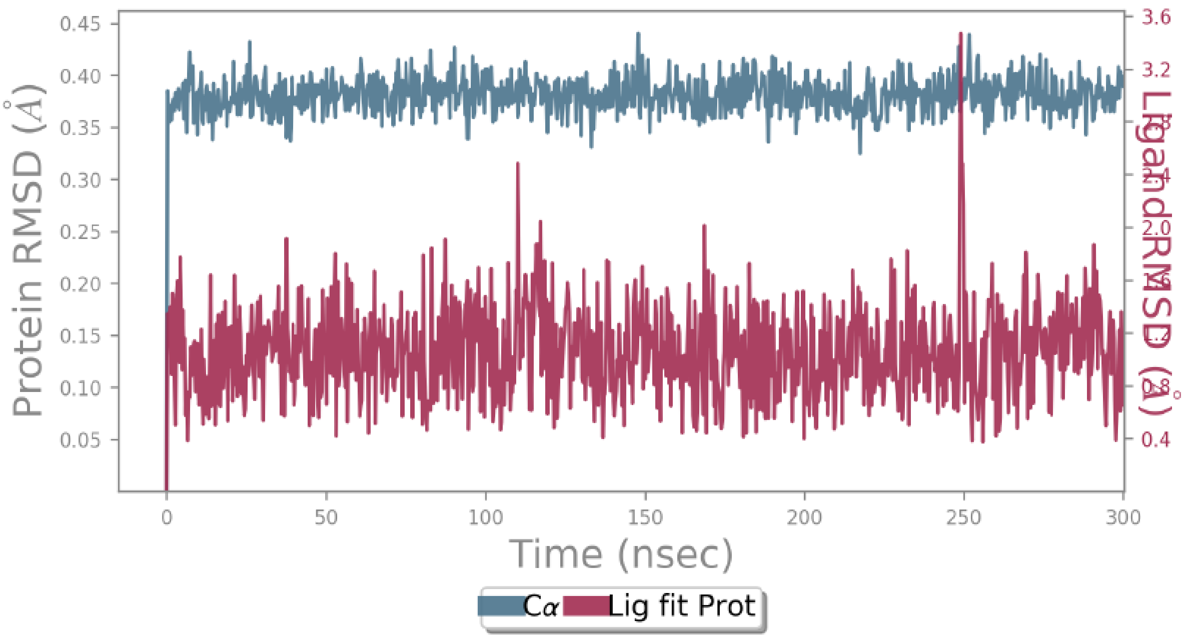

Supplementary Fig. 98. *The protein and ligand root mean square deviation (RMSD) analysis of 300 ns molecular dynamics simulation of compound **11** in the binding site of galectin-8N.*

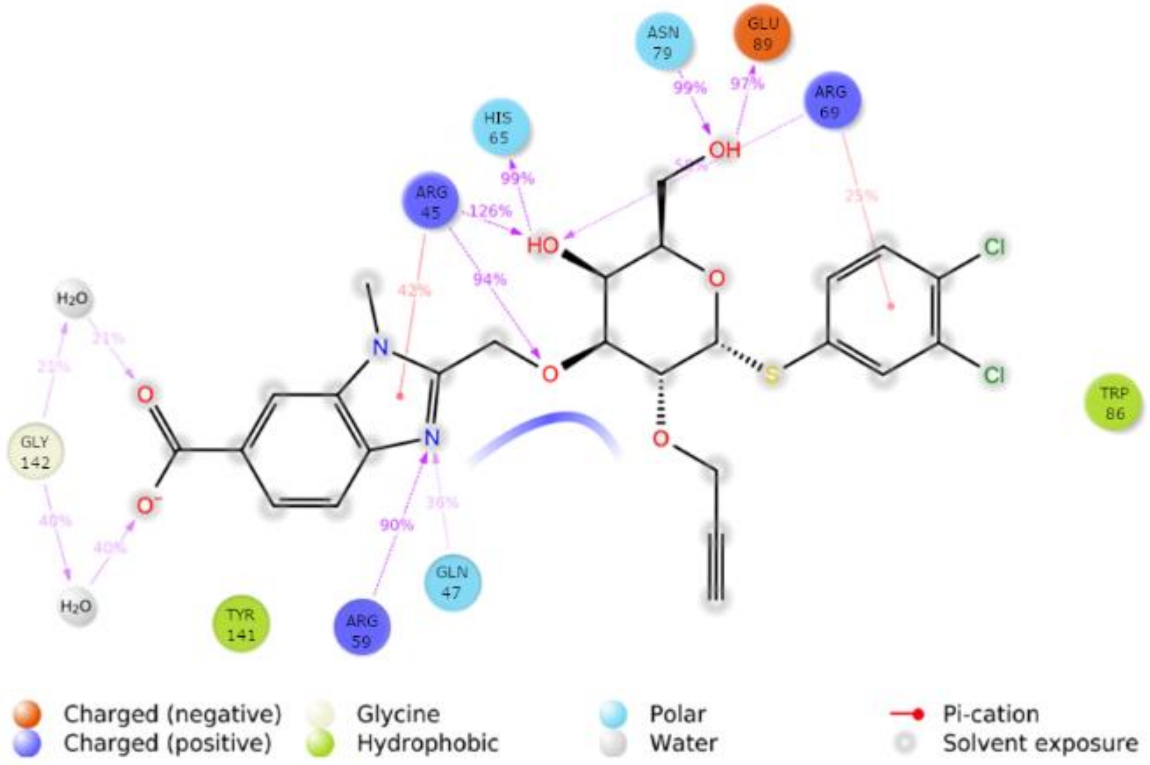

Supplementary Fig. 99. *A schematic representation of ligand atom interactions with the protein residues. Interactions that occur more than 20.0% of the MD simulation time in the trajectory of compound **11** in the binding site of galectin-8N, are shown.*

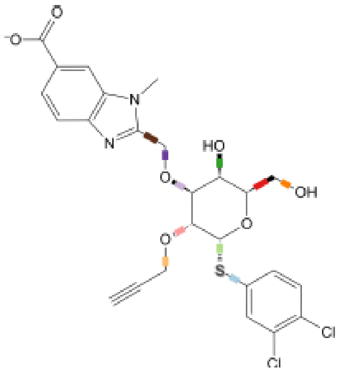
O
-O
N
N
HO
OH
O
O
O
S
Cl
Cl

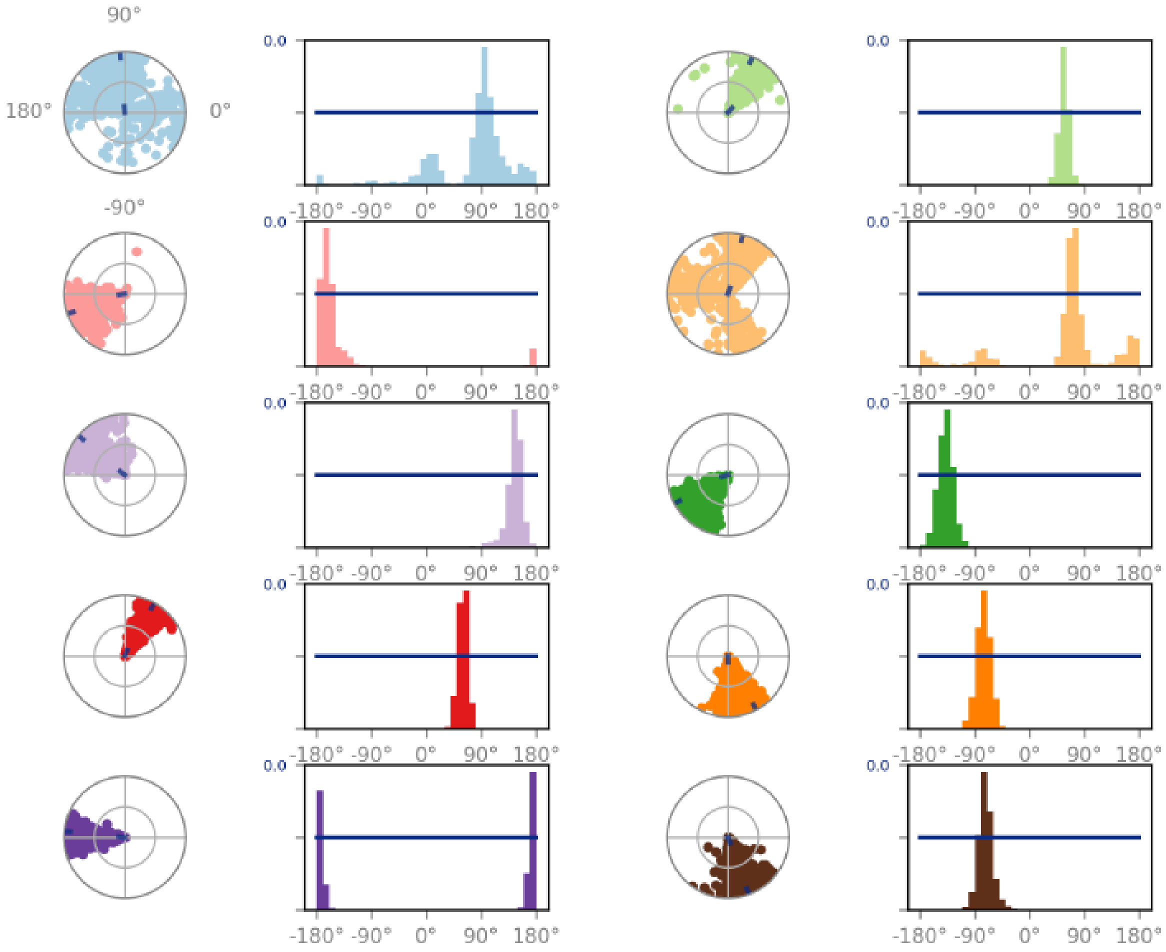
90°
180°
0°
-90°
0.0
-180° -90° 0° 90° 180°

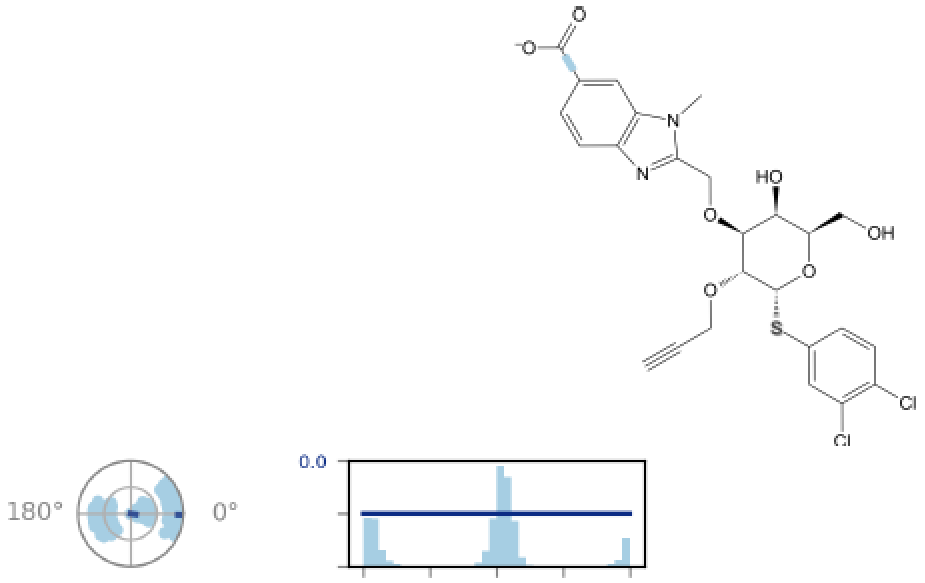

Supplementary Fig. 100. *Ligand torsion profile of **11** in the binding site of galectin-8N, summarizing the conformational evolution of every rotatable bond in the ligand throughout the simulation trajectory. The top panel shows the 2D schematic of a ligand with color-coded rotatable bonds. Each rotatable bond torsion is accompanied by a dial plot and bar plots of the same colour.*

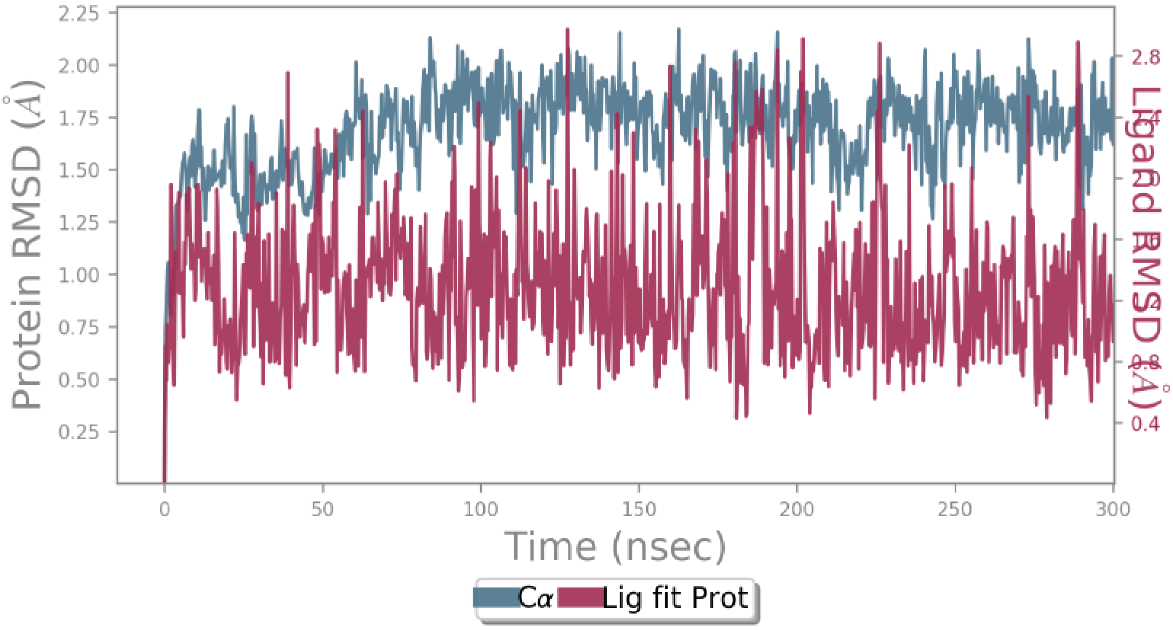

Supplementary Fig. 101. *The protein and ligand root mean square deviation (RMSD) analysis of 300 ns molecular dynamics simulation of compound **29** in the binding site of galectin-8N.*

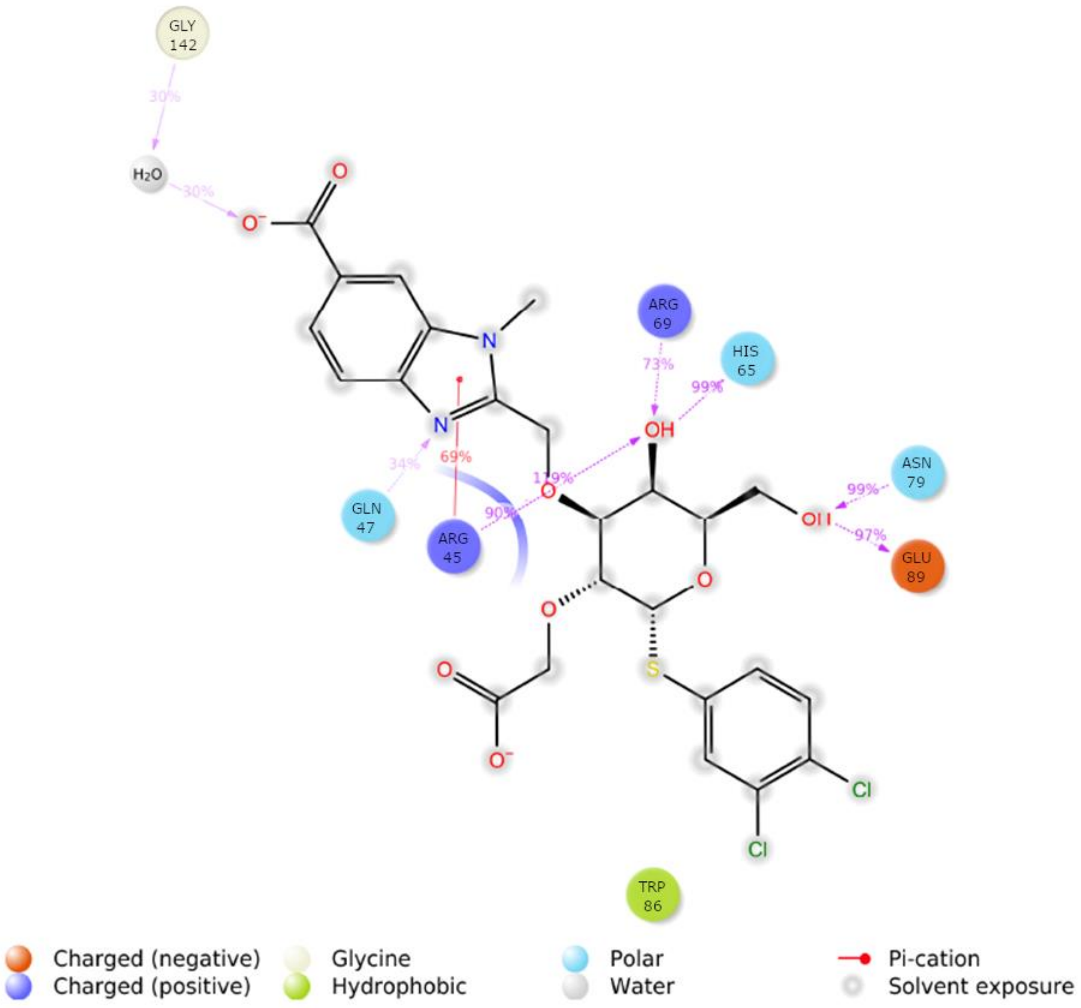

Supplementary Fig. 102. *A schematic representation of ligand atom interactions with the protein residues. Interactions that occur more than 20.0% of the MD simulation time in the trajectory of compound* ***29*** *in the binding site of galectin-8N, are shown.*

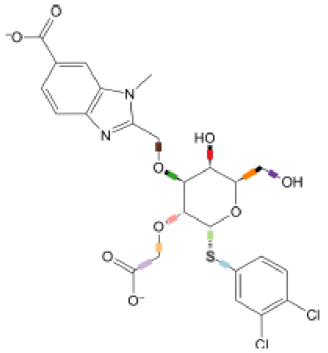
O
⁻O
N
N
HO
O
OH
O
O
O
S
O
O⁻
Cl
Cl

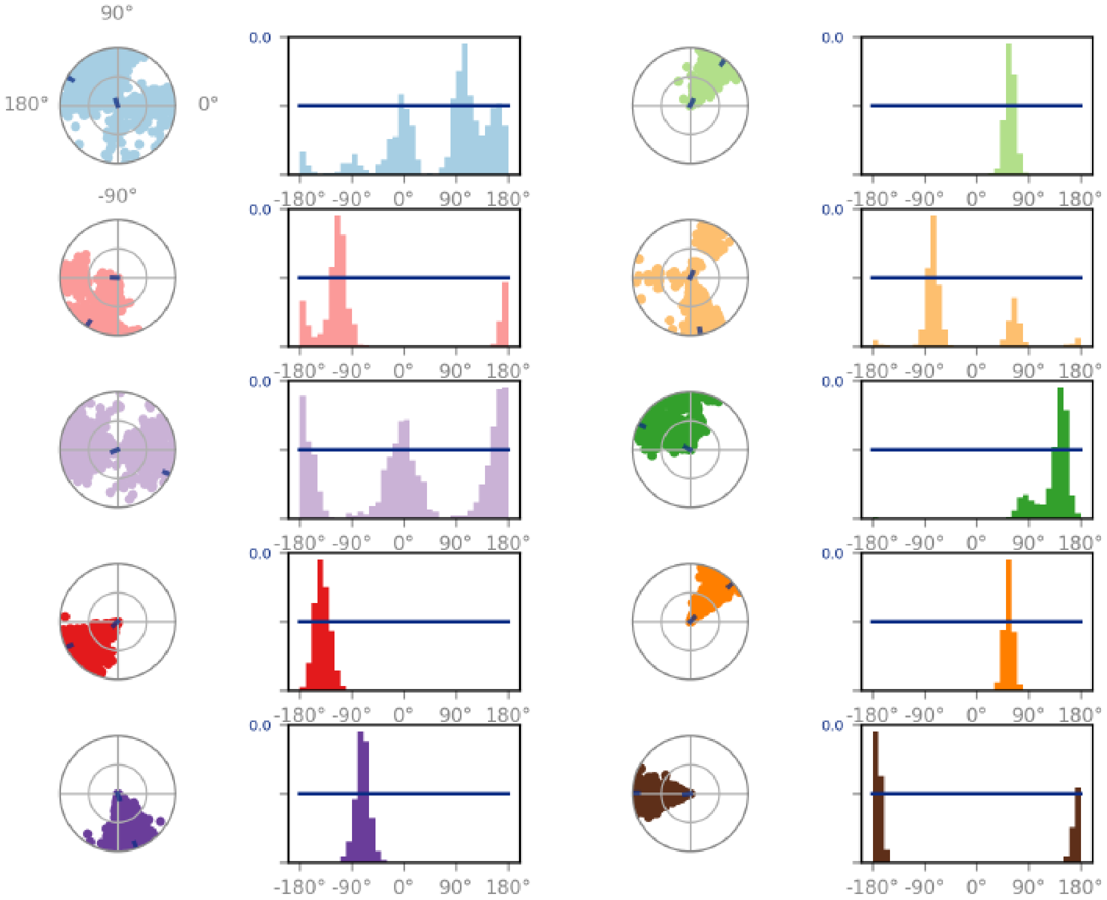
90°
180°
0°
-90°
0.0
-180° -90° 0° 90° 180°

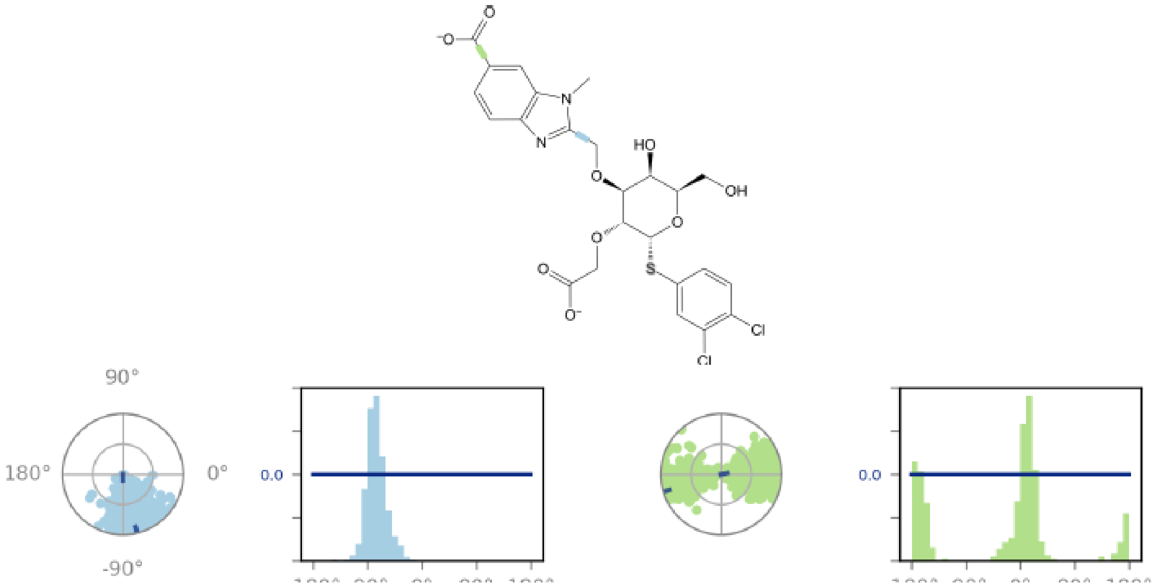

Supplementary Fig. 103. *Ligand torsion profile of **29** in the binding site of galectin-8N, summarizing the conformational evolution of every rotatable bond in the ligand throughout the simulation trajectory. The top panel shows the 2D schematic of a ligand with color-coded rotatable bonds. Each rotatable bond torsion is accompanied by a dial plot and bar plots of the same colour.*

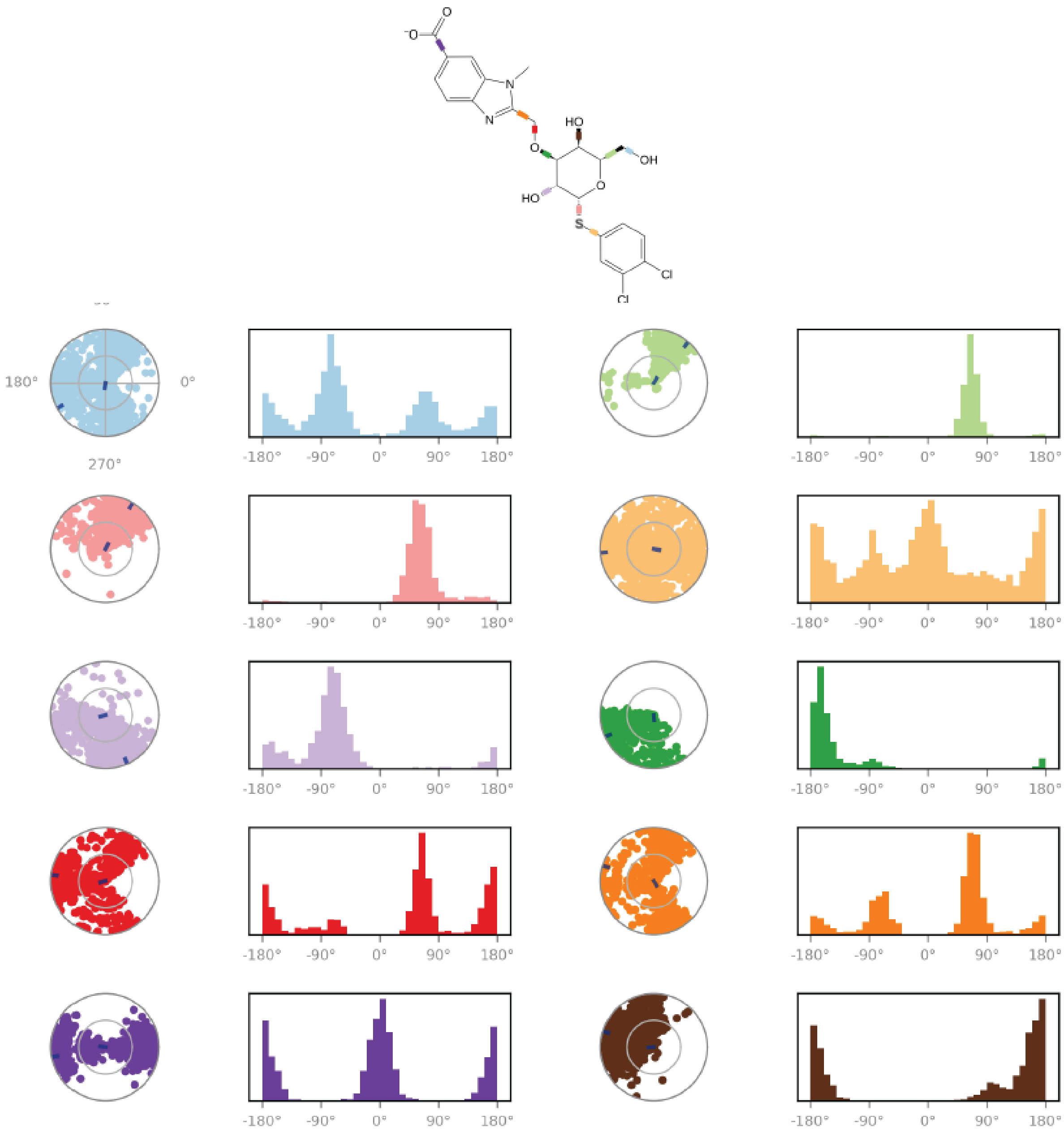

Supplementary Fig. 104. *Ligand torsion profile of unbound* ***1****, summarizing the conformational evolution of every rotatable bond in the ligand throughout the simulation trajectory. The panel shows the 2D schematic of a ligand with color-coded rotatable bonds. Each rotatable bond torsion is accompanied by a dial plot and bar plots of the same colour.*

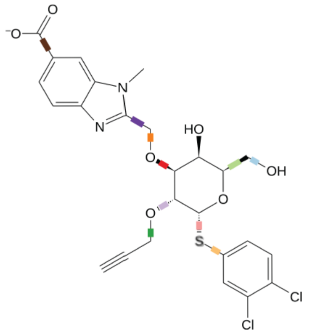
O
⁻O
N
N
HO
O
OH
O
O
S
Cl
Cl

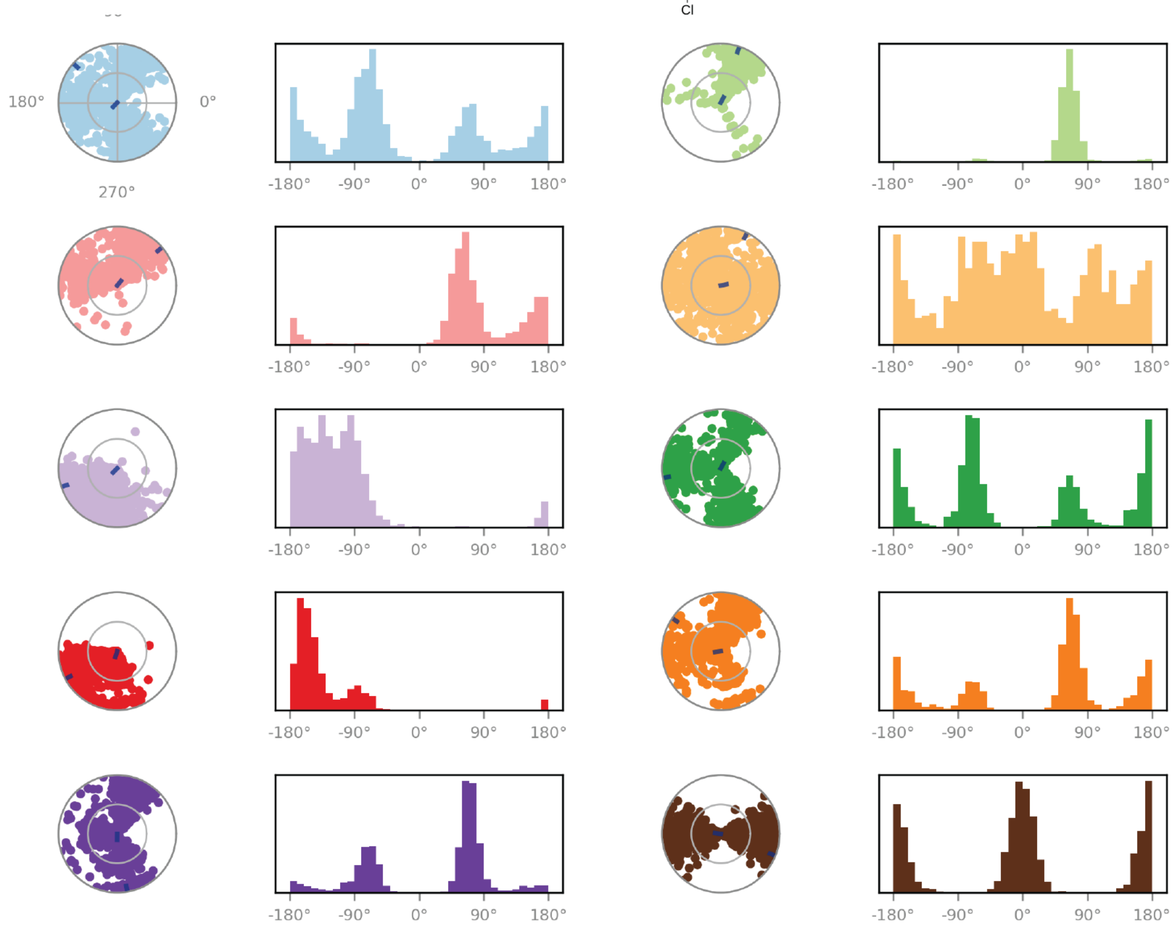
180°
0°
270°
-180°
-90°
0°
90°
180°

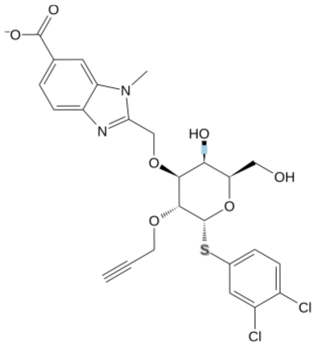
O
⁻O
N
N
HO
O
OH
O
O
S
Cl
Cl

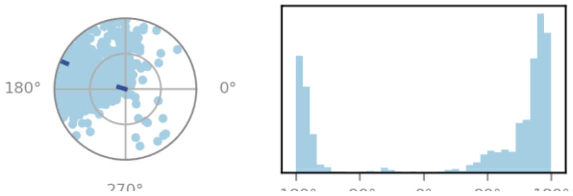

Supplementary Fig. 105. *Ligand torsion profile of unbound **11**, summarizing the conformational evolution of every rotatable bond in the ligand throughout the simulation trajectory. The top panel shows the 2D schematic of a ligand with color-coded rotatable bonds. Each rotatable bond torsion is accompanied by a dial plot and bar plots of the same colour.*

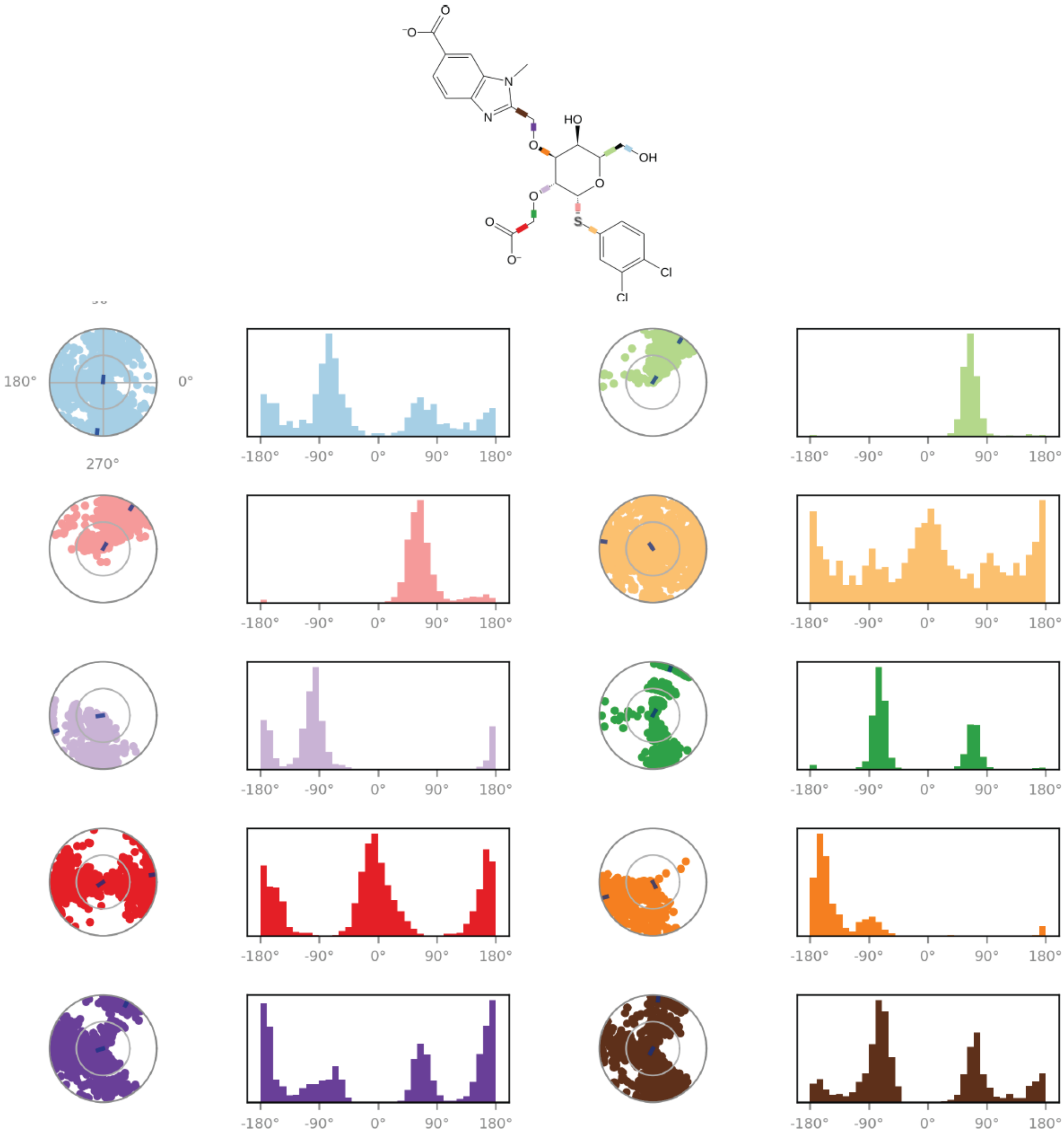

Supplementary Fig. 106. *Ligand torsion profile of unbound **29**, summarizing the conformational evolution of every rotatable bond in the ligand throughout the simulation trajectory. The top panel shows the 2D schematic of a ligand with color-coded rotatable bonds. Each rotatable bond torsion is accompanied by a dial plot and bar plots of the same colour.*

A

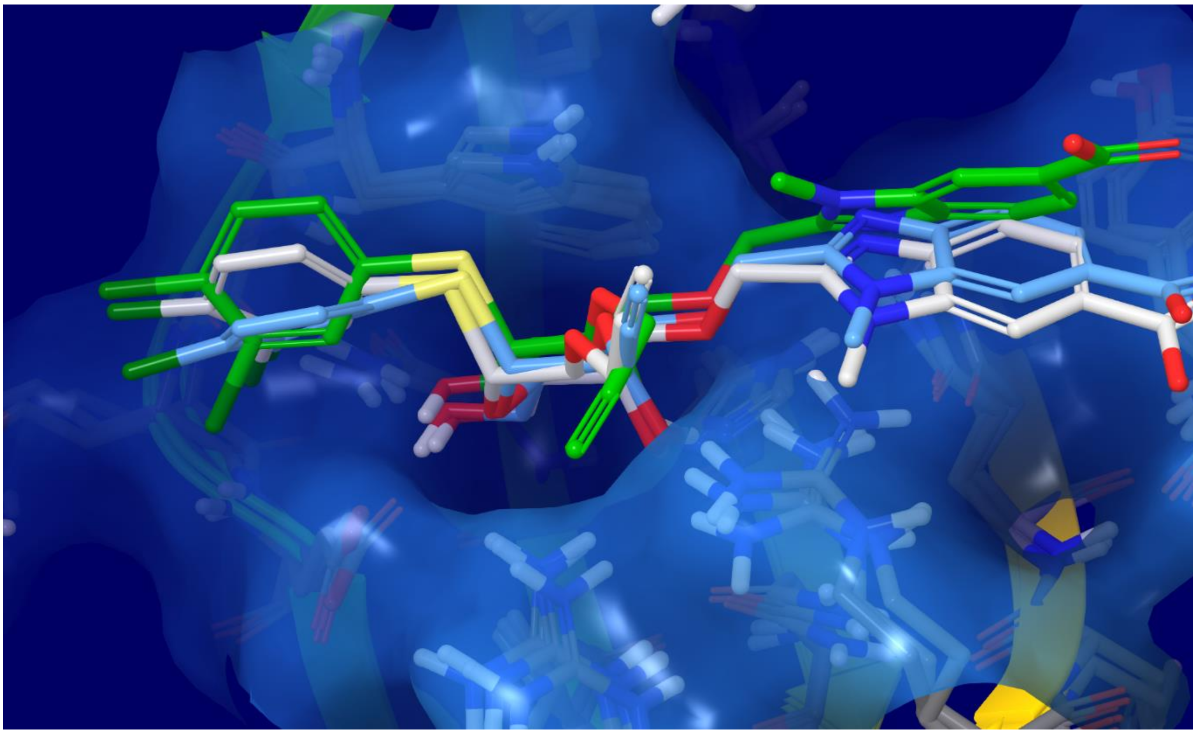

B

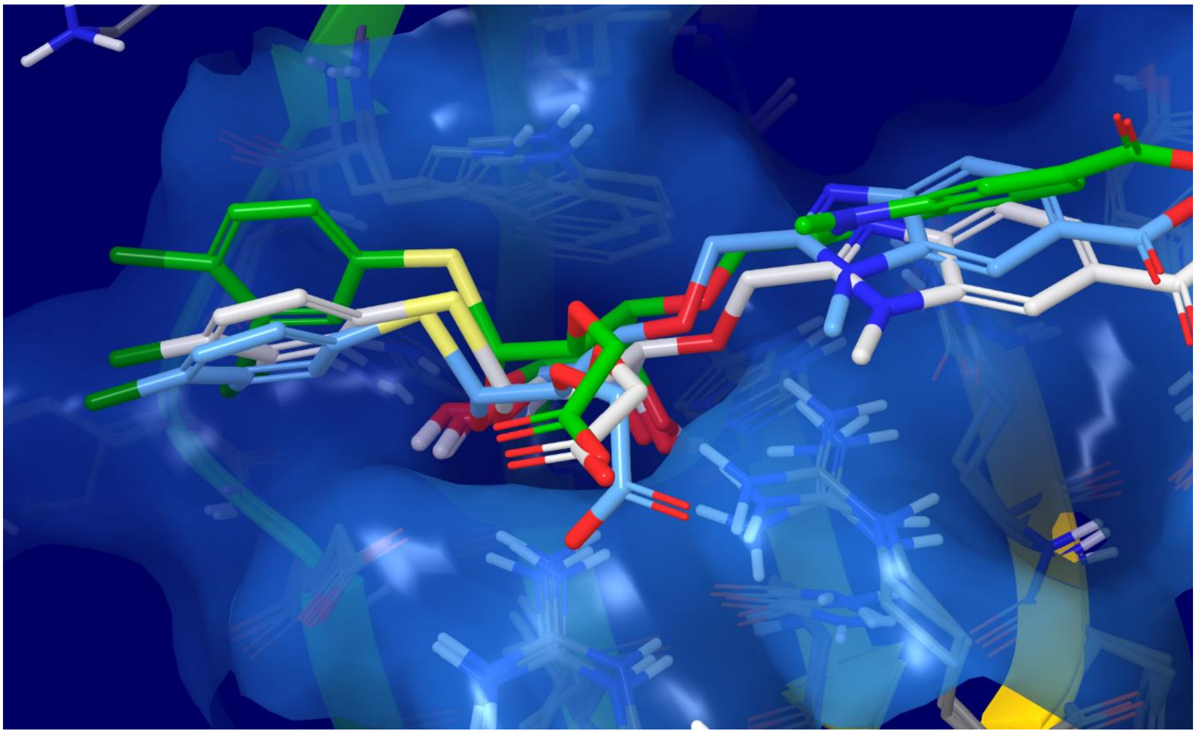

Supplementary Fig. 107. *Clusters of compounds* ***11*** *(A) and* ***29*** *(B) bound to Gal-8 using affinity clustering[4] which finds "exemplars" for each cluster. MD simulations were performed on Gal-8N with bound compound* ***11*** *and of bound compound* ***29****, both structures based on the crystal of compound* ***11*** *in complex with Gal-8N. Clustering of the 200ns trajectories did not feature any direct interactions between the galactose 2-O substituents and the protein. This is probably caused by the lack of parameters for general π-π orbital interactions in molecular mechanics force fields. The clusters of both compound* ***11*** *and of compound* ***29*** *had one thing in common, the methylene group shielded the hydrophobic face of the guanidine moiety of Arg45 from contact with water.*

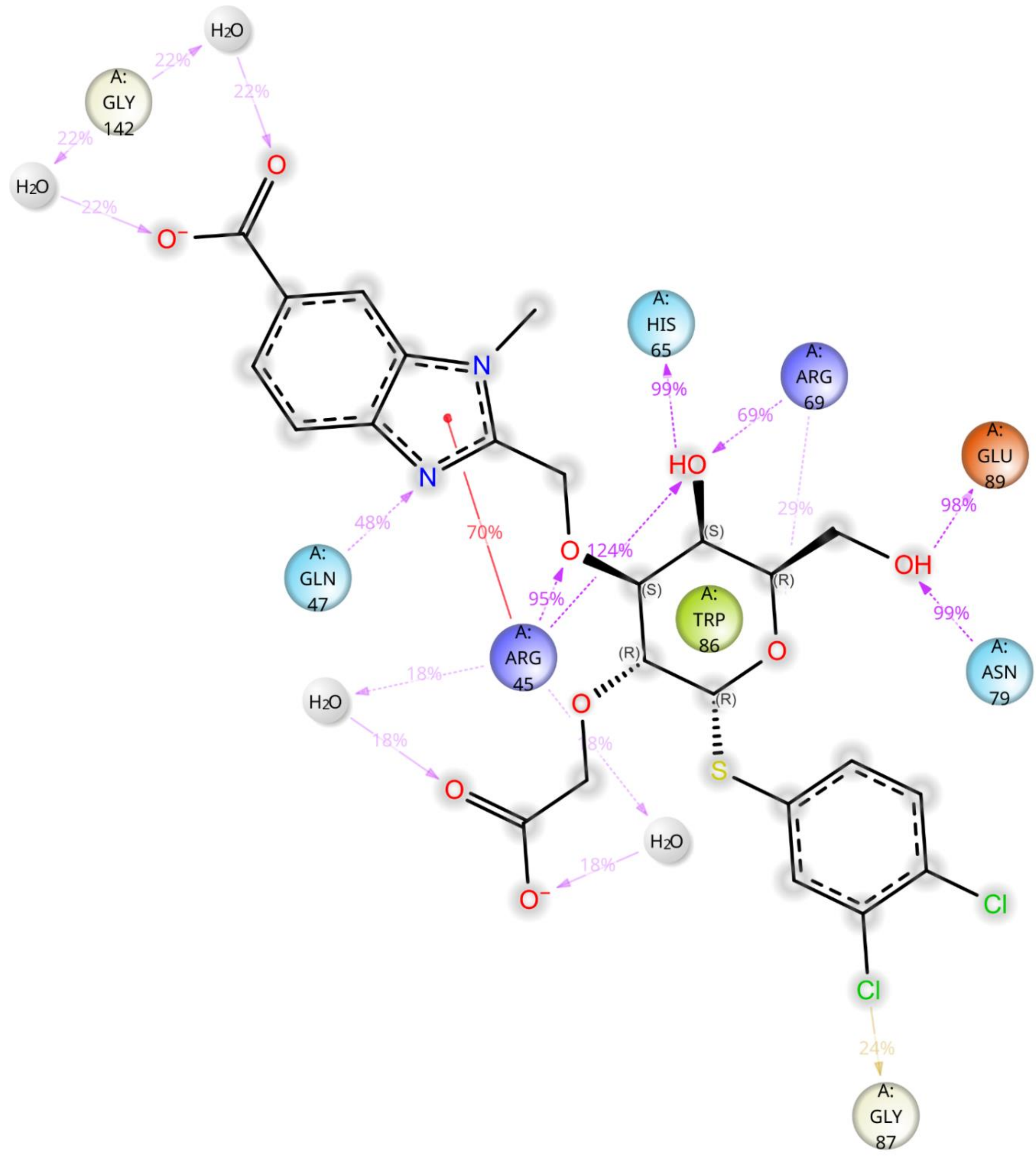

Supplementary Fig. 108. *Ligand interaction diagram for* ***29*** *after affinity clustering. Compound* ***29*** *was found to have the carboxylate stationary in all of the major clusters and analysis of the trajectory featured a single water mediated contact with Arg45 during 36% of the trajectory, but the carboxylate likely also has multiple interactions to both Arg45 and to Arg69 mediated with two water molecules.*

Energy Decomposition Analysis

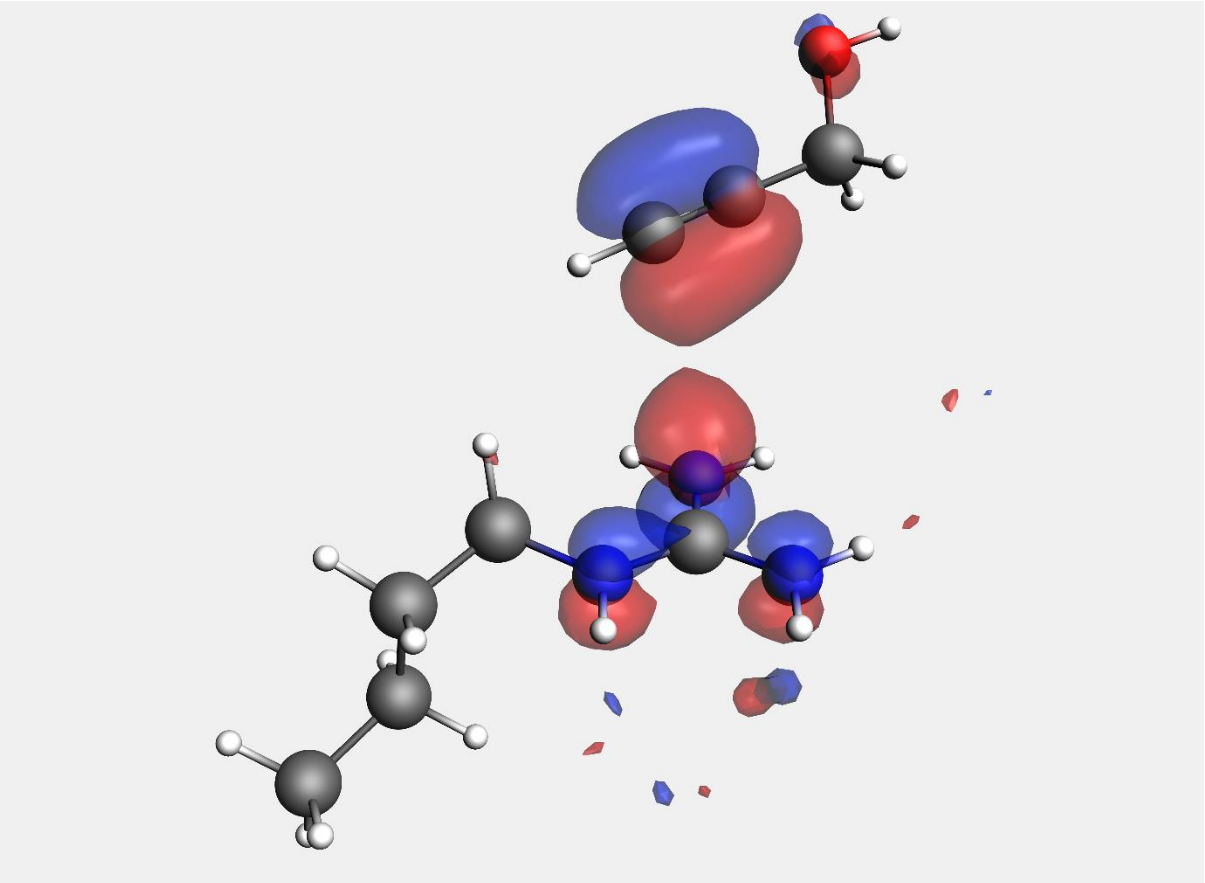

Supplementary Fig. 109. *A plot of the molecular orbitals causing the bonding interaction between the two fragments: the bonding molecular orbital indicates a π-π* interaction between the acetylene of* ***11*** *and guanidinium ion of Arg45.*

Crystal structure

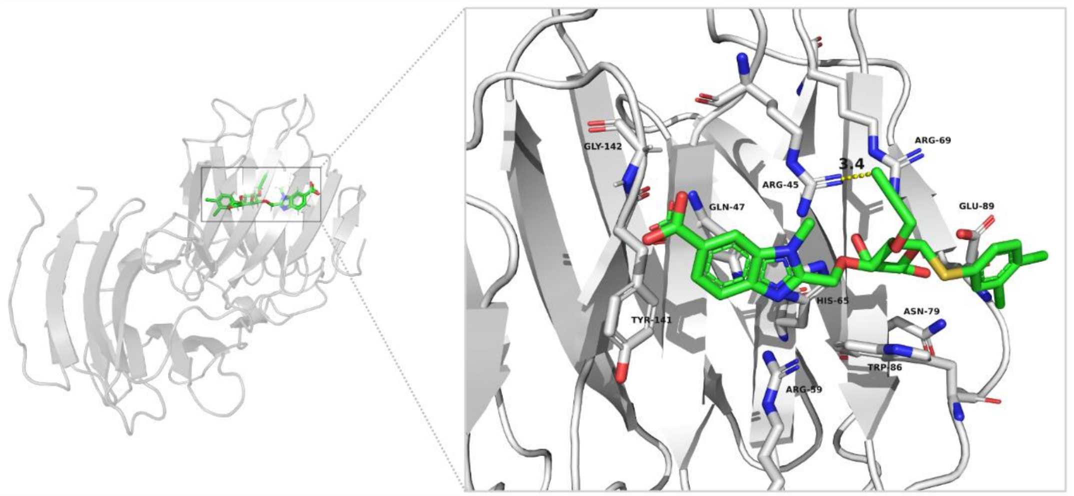

Supplementary Fig. 210*. 3D structure of galectin-8N in complex with* ***11*** *(PDB ID: 9FYJ: grey cartoon representation of galectin-8N,* ***11*** *in sticks: carbon in green, oxygen in red, sulphur in yellow, chlorine in forest green, nitrogen in blue). The distance between the terminal propargyl carbon atom of* ***11*** *and the terminal Arg45 nitrogen atom of galectin-8N is labelled.*

LNnT fluorescent probe binding data

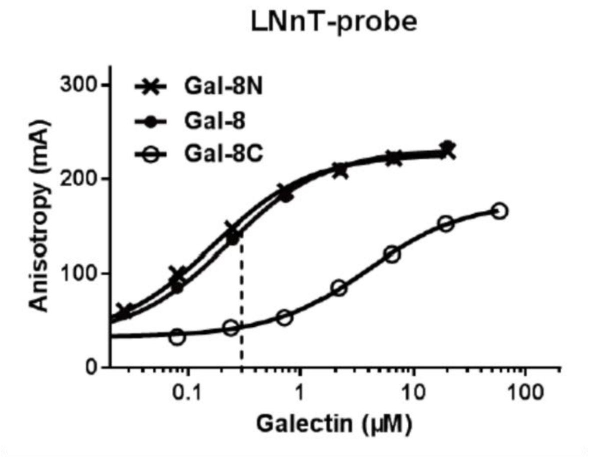

Supplementary Fig. 111. *Binding of galectin-8, galectin-8N and galectin-8C to LNnT fluorescent probe.*

Supplementary References

1. Hassan, M. *et al.* Benzimidazole–galactosides bind selectively to the Galectin-8 N-Terminal domain: Structure-based design and optimisation. *Eur. J. Med. Chem.* **223**, 113664 (2021).

2. Pal, K. B. *et al.* Quinoline–galactose hybrids bind selectively with high affinity to a galectin-8 N-terminal domain. *Org. Biomol. Chem.* **16**, 6295–6305 (2018).

3. Öberg, C. T., Carlsson, S., Fillion, E., Leffler, H. & Nilsson, U. J. Efficient and Expedient Two-Step Pyranose-Retaining Fluorescein Conjugation of Complex Reducing Oligosaccharides: Galectin Oligosaccharide Specificity Studies in a Fluorescence Polarization Assay. *Bioconjug. Chem.* **14**, 1289–1297 (2003).

4. Frey, B. J. & Dueck, D. Clustering by passing messages between data points. *Science.* **315**, 972–976 (2007).
