# Supplementary material for: Nanomolar inhibitor of the galectin-8 N-terminal domain binds via a non-canonical cation-π interaction
Source: Commun Chem. 2025 Feb 24;8:59. doi: 10.1038/s42004-025-01458-6 (PMC11850616; doi:10.1038/s42004-025-01458-6)
Supplement: Supplementary file 3 — Description of Additional Supplementary Files [file 42004_2025_1458_MOESM3_ESM.pdf]

Description of Additional Supplementary Files

File name: Supplementary Data 1

Description: Energy Decomposition Analysis (EDA) with separate specific bonding contributions.
